# Light Lanthanide Metallocenium Cations Exhibiting Weak Equatorial Anion Interactions

**DOI:** 10.1002/chem.201901167

**Published:** 2019-05-13

**Authors:** Jingjing Liu, Daniel Reta, Jake A. Cleghorn, Yu Xuan Yeoh, Fabrizio Ortu, Conrad A. P. Goodwin, Nicholas F. Chilton, David P. Mills

**Affiliations:** ^1^ School of Chemistry The University of Manchester Manchester M13 9PL UK

**Keywords:** cyclopentadienyl ligands, lanthanides, magnetic properties, metallocenes, sandwich complexes

## Abstract

As the dysprosocenium complex [Dy(Cp^ttt^)_2_][B(C_6_F_5_)_4_] (Cp^ttt^=C_5_H_2_ *t*Bu_3_‐1,2,4, **1‐Dy**) exhibits magnetic hysteresis at 60 K, similar lanthanide (Ln) complexes have been targeted to provide insights into this remarkable property. We recently reported homologous [Ln(Cp^ttt^)_2_][B(C_6_F_5_)_4_] (**1‐Ln**) for all the heavier Ln from Gd–Lu; herein, we extend this motif to the early Ln. We find, for the largest Ln^III^ cations, that contact ion pairs [Ln(Cp^ttt^)_2_{(C_6_F_5_‐κ^1^‐*F*)B(C_6_F_5_)_3_}] (**1‐Ln**; La–Nd) are isolated from reactions of parent [Ln(Cp^ttt^)_2_(Cl)] (**2‐Ln**) with [H(SiEt_3_)_2_][B(C_6_F_5_)_4_], where the anion binds weakly to the equatorial sites of [Ln(Cp^ttt^)_2_]^+^ through a single fluorine atom in the solid state. For smaller Sm^III^, [Sm(Cp^ttt^)_2_][B(C_6_F_5_)_4_] (**1‐Sm**) is isolated, which like heavier **1‐Ln** does not exhibit equatorial anion interactions, but the Eu^III^ analogue **1‐Eu** could not be synthesised due to the facile reduction of Eu^III^ precursors to Eu^II^ products. Thus with the exception of Eu and radioactive Pm this work constitutes a structurally similar family of Ln metallocenium complexes, over 50 years after the [M(Cp)_2_]^+^ series was isolated for the 3d metals.

## Introduction

Since the discovery of ferrocene [Fe(Cp)_2_] (Cp=cyclopentadienyl, C_5_H_5_) in the middle of the 20th century,^1^ there has been an explosion of research in organometallic chemistry, leading to applications in some unexpected areas such as optical and redox devices, batteries, sensing, catalysis and medicine.^2^ Following the landmark discovery of ferrocene, derivatized metallocenes [M(Cp^R^)_2_] (Cp^R^=substituted Cp) have been synthesised across the s‐, p‐, d‐ and f‐block elements.^3^ The first examples of lanthanide (Ln) Cp complexes were synthesised in 1954,^4^ and this area has grown to the extent that Ln organometallic chemistry is now dominated by Cp^R^ ligands.^5^

Although isolated d‐block metallocenium cations have been known since ferrocenium, [Fe(Cp)_2_]^+^, was isolated in 1952^6^ and Ln metallocenium cations, [Ln(Cp)_2_]^+^, were first predicted in 1956 by Birmingham and Wilkinson,^7^ it was only recently with the report of the dysprosocenium complex [Dy(Cp^ttt^)_2_][B(C_6_F_5_)_4_] (Cp^ttt^=C_5_H_2_ *t*Bu_3_‐1,2,4, **1**‐**Dy**),^8^ and heavy Ln metallocenium homologues [Ln(Cp^ttt^)_2_][B(C_6_F_5_)_4_] (**1**‐**Ln**, Ln=Y, Gd, Tb, Ho, Er, Tm, Yb, Lu),^8^, ^9^, ^10^ that structurally authenticated Ln metallocenium complexes with no equatorial interactions were achieved. The paucity of isolated Ln metallocenium cations prior to 2017 can be attributed to the propensity for large electropositive Ln^III^ cations to maximise their coordination numbers, coupled with their preference for hard donor ligands not being well‐satisfied by soft Cp^R^ coordination.^5^ Of most relevance here, [Sc(Cp*)_2_{(C_6_F_5_‐κ^2^‐*F*)B(C_6_F_5_)_3_}] (Cp*=C_5_Me_5_)^11^ and [Ln(Cp*)_2_{(μ‐C_6_F_5_‐κ^1^‐*F*)_2_B(C_6_F_5_)_2_}]_2_ (Ln=Pr, Nd)^12^ exhibit equatorial interactions with F atoms of the weakly coordinating anion in the solid state.

Complex **1**‐**Dy** is a single‐molecule magnet (SMM) that exhibits magnetic hysteresis at 60 K;^8^ this was a leap of 46 K over the previous record temperature for molecular magnetic hysteresis (at a comparable sweep rate) of 14 K set by Evans and Long in 2011.^13^ Since the publication of **1**‐**Dy** peralkylated [Dy(Cp^R^)_2_]^+^ cations have been shown to exhibit hysteresis up to 72 K^14^ and 80 K.^15^ The high hysteresis temperature of **1**‐**Dy** and derivatives cannot solely be attributed to their strong axial ligand fields stabilising the most magnetic *m_J_*=±15/2 states,^16^ as the previous record effective barrier to the reversal of magnetization was seen for [Dy(*t*BuO)_2_(pyridine)_5_] (1261 cm^−1^) but this complex does not exhibit hysteresis above 4 K.^17^ State‐of‐the‐art calculations of the spin–phonon coupling in **1**‐**Dy** show that magnetic relaxation in the Orbach (over‐barrier) regime occurs due to localised molecular vibrations, and suggests that the high magnetic hysteresis temperature arises from the constrained metal–ligand vibrational modes intrinsic to the *bis*‐η^5^‐Cp^ttt^ coordination geometry.^8^ We have subsequently shown that isolated [Ln(Cp^ttt^)_2_]^+^ cations exhibit anomalously low Raman exponents, distinct from analogues with equatorial ligands, suggesting the unique vibrational modes of Cp^ttt^ are important across a wide temperature range.^8^, ^9^, ^10^

We previously reported that the bulky *bis*‐Cp^ttt^ framework in combination with the weakly coordinating anion [B(C_6_F_5_)_4_]^−^ provide isolated Ln metallocenium cations for smaller, heavier Ln.^8^, ^9^, ^10^ These studies showed that [H(SiEt_3_)_2_][B(C_6_F_5_)_4_]^18^ is effective at abstracting either fluoride or chloride from [Ln(Cp^ttt^)_2_(X)] precursors to yield **1**‐**Ln**. Herein, we extend these studies to the lighter, larger Ln to define the characteristic features of analogous [Ln(Cp^ttt^)_2_]^+^ cations across the Ln series. We find that for the largest members of the **1**‐**Ln** series one *meta*‐F of the [B(C_6_F_5_)_4_]^−^ anion coordinates to the exposed equatorial site in the solid state, giving [Ln(Cp^ttt^)_2_{(C_6_F_5_‐κ^1^‐F)B(C_6_F_5_)_3_}] (**1**‐**Ln**; Ln=La, Ce, Pr, Nd). However, smaller Sm^III^ yields an isolated cation, [Sm(Cp^ttt^)_2_][B(C_6_F_5_)_4_] (**1**‐**Sm**), which is structurally analogous to the heavier members of the series. We were unable to complete the series with the Eu analogue **1‐Eu** as the stability associated with the +2 oxidation state^5^ precluded the synthesis of [Eu(Cp^ttt^)_2_(X)] (X=F, Cl) precursors.

## Results and Discussion

**Scheme 1:**
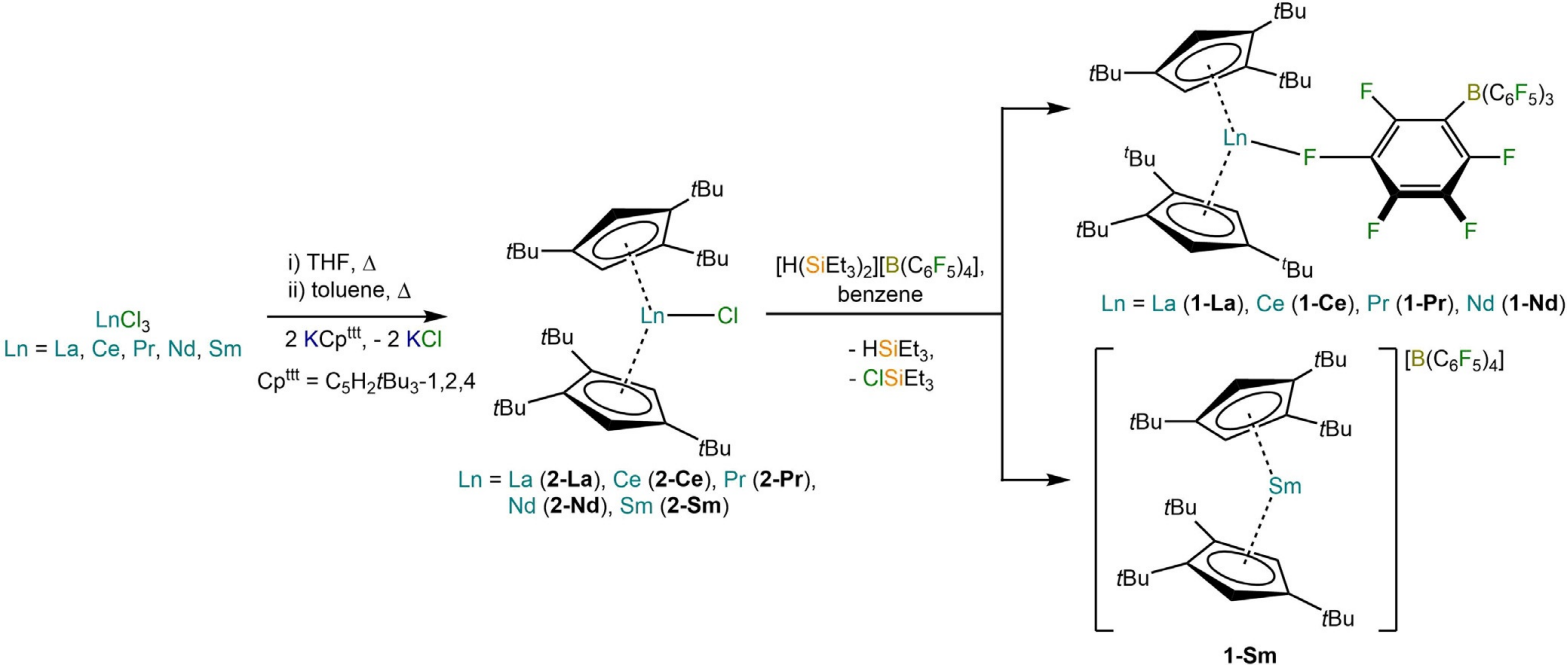
Synthesis of the Ln complexes **1‐Ln** and **2‐Ln**.

According to previously published synthetic methods for the synthesis of **1‐Ln** (Ln=Gd, Tb, Dy, Ho, Er, Tm, Yb, Lu, Y),^8^, ^9^, ^10^ [Ln(Cp^ttt^)_2_{(C_6_F_5_‐κ^1^‐*F*)B(C_6_F_5_)_3_}] (**1‐Ln**; Ln=La, Ce, Pr, Nd) and [Sm(Cp^ttt^)_2_][B(C_6_F_5_)_4_] (**1‐Sm**) were all synthesised by the reactions of [Ln(Cp^ttt^)_2_(Cl)] (**2‐Ln**; Ln=La, Ce, Pr, Nd, Sm) with [H(SiEt_3_)_2_][B(C_6_F_5_)_4_]^18^ in benzene (Scheme 1). [H(SiEt_3_)_2_][B(C_6_F_5_)_4_] was selected as a reagent for Ln metallocenium cation formation as: (i) it is soluble in benzene, hence strongly coordinating O‐donor solvents can be avoided; (ii) there is a significant enthalpic effect due to formation of a strong Si−Cl bond, and (iii) there is an additional entropic effect arising from two reactant species giving three product species. Complexes **1‐Ln** (Ln=Ce, Pr, Nd) were crystallised from dichloromethane (DCM) layered with hexane and became more temperature‐sensitive as soon as DCM was added; thus, these complexes were stored at −25 °C and measurements were performed below −25 °C. Despite repeated efforts we were unable to recrystallise **1‐Ln** (Ln=La, Sm) from DCM/hexane; we later found that these complexes dissolved in hot toluene and could be recrystallised and stored at room temperature.

The precursors **2‐Ln** were synthesized from LnCl_3_ and two equivalents of KCp^ttt^ by modification of the synthesis of **2‐Pr** and **2‐Nd** from the parent LnCl_3_ and NaCp^ttt^ (Scheme 1).^19^ This strategy does not work for Eu as it has previously been shown that the reaction of EuCl_3_ with NaCp^ttt^ gives [Eu(Cp^ttt^)_2_], with elimination of half an equivalent of (Cp^ttt^)_2_ via reductive coupling.^20^ We attempted the synthesis of an alternative precursor [Eu(Cp^ttt^)_2_(F)] by the oxidation of [Eu(Cp^ttt^)_2_]^20^ with [Fe(Cp)_2_][PF_6_]^21^ in toluene, envisaging that this would eliminate gaseous PF_5_ and [Fe(Cp)_2_]. However, this did not occur and instead we obtained [{Eu(Cp^ttt^)(μ‐PF_6_‐κ^4^‐*F*)(THF)}_2_] (**3**) in 60 % yield, along with several crystals of (Cp^ttt^)_2_ (**4**), presumably due to the contamination of the Eu^II^ starting material with THF solvates, such as [Eu(Cp^ttt^)_2_(THF)] or [Eu(Cp^ttt^)(I)(THF)_*x*_]_*n*_, and subsequent ligand scrambling. Therefore, Eu is the only Ln apart from radioactive Pm that we were unable to access via these anion abstraction procedures. Crystalline yields for **2‐Ln** are similar (46–52 %) except for **2‐La**, which was isolated in 31 % yield. For **1‐Ln**, the crystalline yields were dependent upon the solvent system; yields were significantly higher when crystallised from toluene (67 % for **1‐La** and 69 % for **1‐Sm**) compared to those crystallised from DCM layered with hexane (21–59 %).

Elemental analysis results obtained for **1‐Ln** and **2‐Ln** were generally in excellent agreement with expected values, although low carbon values, likely due to carbide formation from incomplete combustion, were consistently obtained in some cases. In spite of this, the bulk purity of **1‐Ln** was indicated by the consistency of other analytical data with their formulations (see below). Complex **1‐Sm** contains the largest Ln^III^ ion in a Ln metallocenium cation with no equatorial interactions to be isolated to date. The most appropriate explanation for the structural change for the largest Ln^III^ cations is a simple argument based on ionic radii, with the two Cp^ttt^ rings offering a sufficient amount of steric protection around the smaller Sm^III^ ion (CN=6, IR=0.958 Å; CN: effective coordination number; IR: ionic radius)^21^ to saturate the coordination sphere and prevent coordination of the counter‐ion, whereas the early Ln^III^ ions are large enough to accommodate a further interaction (e.g. Nd^III^: CN=6, IR=0.983 Å).^22^ This hypothesis can be tested in future by steric and electronic variations of the Cp^R^ ligands and the weakly coordinating anion.

^1^H NMR spectra were recorded from −350 to +350 ppm for **1‐Ln** and **2‐Ln** (Table 1). Significant broadening of signals was seen for all paramagnetic species: the *t*Bu group protons were observed in all cases apart from **1‐Pr**, however, the Cp^ttt^ ring protons were only seen in **1‐La**, **2‐La**, **1‐Sm** and **2‐Sm**. Three signals were observed in the ^1^H NMR spectra of diamagnetic **1‐La** and **2‐La**, and paramagnetic **2‐Sm**, in a ratio of 18:36:4, corresponding to two sets of *t*Bu groups and the Cp^ttt^ ring protons, respectively. The paramagnetism of most **1‐Ln** precluded assignment of their ^13^C{^1^H} NMR spectra. However, for diamagnetic **1‐La** and **2‐La** these could be interpreted: for **1‐La** the expected three Cp^ttt^ ring carbon signals were identified at 135.96, 147.78 and 149.70 ppm, whereas six resonances for the *t*Bu groups were seen between 29.86 and 32.82 ppm. These are assigned as three quaternary and three methyl signals; multiple signals in this region were previously observed for **1‐Lu**.^9^ The [B(C_6_F_5_)_4_]^−^ anions in **1‐Ln** were observed by ^11^B{^1^H} and ^19^F{^1^H} NMR spectroscopy (see Supporting Information). In the case of **1‐La**, we could also employ ^13^C{^19^F} NMR spectroscopy to characterise the [B(C_6_F_5_)_4_]^−^ anion: the expected four aromatic resonances were located at 124.26 (*ipso*‐), 136.91 (*m*‐), 138.79 (*p*‐) and 148.71 ppm (*o*‐); the signal for the *ipso*‐carbon is a quartet from coupling with ^11^B (^1^ *J*_BC_=51.8 Hz). Signals in the ^11^B{^1^H} NMR spectra of **1‐Ln** were observed between −20 and −16 ppm, and three peaks were observed in the ^19^F{^1^H} NMR spectra, corresponding to *meta*‐, *para*‐ and *ortho*‐F. In the ^19^F{^1^H} NMR spectrum of **1‐Sm**, the *para*‐F signal is a triplet from coupling to two adjacent *meta*‐F atoms (*J*_FF_=20.4 Hz), but this is a singlet for **1‐La**, presumably due to broadening by quadrupolar ^139^La (*I*=7/2). The ^11^B and ^19^F NMR spectra of [Sc(Cp*)_2_{(C_6_F_5_‐κ^2^‐*F*)B(C_6_F_5_)_3_}]^11^ and [Ln(Cp*)_2_{(μ‐C_6_F_5_‐κ^1^‐*F*)_2_B(C_6_F_5_)_2_}]_2_ (Ln=Pr, Nd)^12^ have not been reported to the best of our knowledge, but all NMR spectra reported herein are similar to those previously discussed for the heavy **1‐Ln** (Ln=Gd–Lu).^8^, ^9^, ^10^ Together, these data indicate that the [B(C_6_F_5_)_4_]^−^ anions in light **1‐Ln** are only weakly bound in solution, and dissociate to give isolated [Ln(Cp^ttt^)_2_]^+^ cations, as evidenced by the symmetry of ^19^F NMR spectra.^23^ To probe this further, we performed VT ^19^F NMR studies of **1‐La** in C_7_D_8_, but the spectra were unchanged down to 218 K. Finally, the paramagnetism of **3** precluded assignment of its ^1^H, ^13^C, ^19^F and ^31^P NMR spectra.

**Table 1:** ^1^H NMR spectra assignments of **1‐Ln** in [D_2_]DCM and **2‐Ln** in [D_6_]benzene.

Complex	*δ* ^1^H [ppm] {C_5_H_2_C(C*H*_3_)_3_}^−^ (FWHM/Hz)	*δ* ^1^H [ppm] {C_5_ *H*_2_C(CH_3_)_3_}^−^
**1‐La**	1.28, 18 H; 1.46, 36 H	6.26, 4 H
**1‐Ce**	−13.26 (450), 18 H; −7.93 (920), 36 H	–
**1‐Pr**	–	–
**1‐Nd**	−17.88 (310), 18 H; −11.94 (700), 36 H	–
**1‐Sm**	−1.37, 36 H	19.69, 2 H
**2‐La**	1.25, 18 H; 1.52, 36 H	–
**2‐Ce**	−13.06, 18 H; −2.53, 36 H	–
**2‐Pr**	−36.08, 18 H; −7.74 (750), 36 H	–
**2‐Nd**	−18.95, 18 H; −5.58, 36 H	–
**2‐Sm**	−6.01, 18 H; 0.55, 36 H	19.80, 4 H

**Figure 1:**
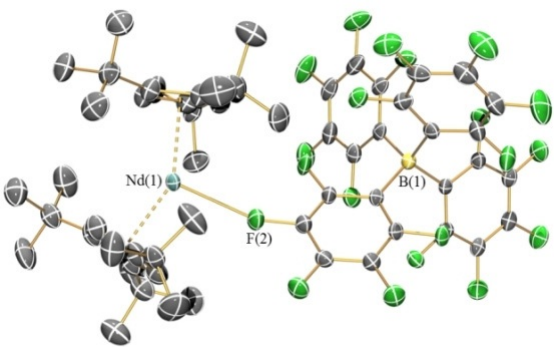
Molecular structure of [Nd(Cp^ttt^)_2_{(C_6_F_5_‐κ^1^‐*F*)B(C_6_F_5_)_3_}] (**1‐Nd**). Displacement ellipsoids set at 30 % probability level and hydrogen atoms are omitted for clarity.

**Figure 2:**
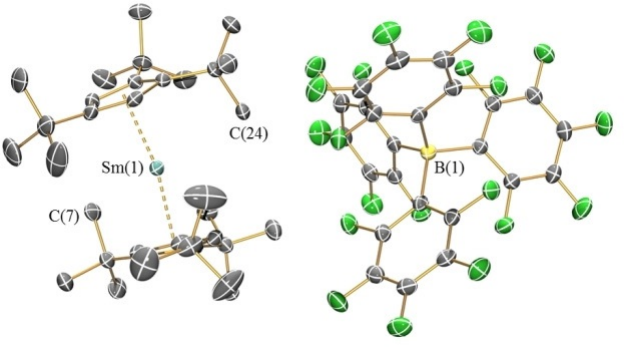
Molecular structure of [Sm(Cp^ttt^)_2_][B(C_6_F_5_)_4_] (**1‐Sm(C_7_H_8_)_1.5_**). Displacement ellipsoids set at 30 % probability level and hydrogen atoms and lattice solvent are omitted for clarity.

**Figure 3:**
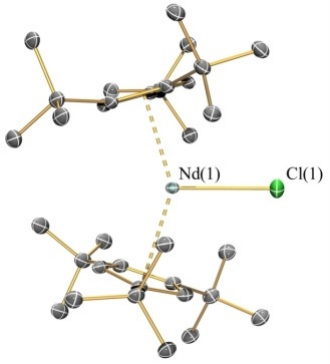
Molecular structure of [Nd(Cp^ttt^)_2_(Cl)] (**2‐Nd**). Displacement ellipsoids set at 30 % probability level and hydrogen atoms are omitted for clarity.

The solid‐state structures of **1‐Ln**, **2‐Ln**, **3** and **4** were determined by single crystal XRD (**1‐Nd** is depicted in Figure 1; **1‐Sm** in Figure 2 and **2‐Nd** in Figure 3; see the Supporting Information for other structures. Selected bond distances and angles of **1‐Ln** are compiled in Table 2). The [Sm(Cp^ttt^)_2_]^+^ cation of **1‐Sm** exhibits identical structural features with **1‐Dy**,^8^ whereas, the early Ln (Ln=La, Ce, Pr, Nd) all exhibit interactions between the Ln centre and a *meta*‐fluorine of one of the C_6_F_5_ rings of the [B(C_6_F_5_)_4_]^−^ anion. In all cases, the [Ln(Cp^ttt^)_2_]^+^ fragments exhibit bent geometries and the Cp_centroid_⋅⋅⋅Ln⋅⋅⋅Cp_centroid_ angles for **1‐Ln** are closer to linearity than the corresponding precursors **2‐Ln**. In the cases of **1‐Sm** and the late Ln metallocenium cations in **1‐Ln** (Ln=Gd, Tb, Dy, Ho, Er, Tm, Yb, Lu),^8^, ^9^, ^10^ the Cp_centroid_⋅⋅⋅Ln⋅⋅⋅Cp_centroid_ angles are significantly more linear than the [Ln(Cp^ttt^)_2_]^+^ fragments in early **1‐Ln** (Ln=La, Ce, Pr, Nd) which have Ln⋅⋅⋅F interactions [range Cp_centroid_⋅⋅⋅Ln⋅⋅⋅Cp_centroid_: early Ln 148.10(13)° to 149.48(11)°; late Ln 150.2(2)° to 155.11(6)°; **1‐Sm**: 153.23(6)°]. Two nearly eclipsed η^5^‐Cp^ttt^ rings coordinate to the Ln^III^ centres in all **1‐Ln**.^8^, ^9^, ^10^ The mean Ln⋅⋅⋅Cp_centroid_ distances decrease regularly across the Ln series [mean La⋅⋅⋅Cp_centroid_=2.503(3) Å; Lu⋅⋅⋅Cp_centroid_=2.246(4) Å].^9^ In the complex with isolated cations **1‐Sm**, two equatorial electrostatic Ln⋅⋅⋅C interactions with carbon atoms from a *t*Bu group were observed [C(7) 2.966(4) Å, C(24) 2.998(4) Å], with a C⋅⋅⋅Ln⋅⋅⋅C angle of 147.56(11)°; this feature has been noted for the heavier Lns previously.^8^, ^9^, ^10^ In the case of **1‐Ln** (Ln=La, Ce, Pr, Nd), Ln⋅⋅⋅F distances were observed between 2.679(9) Å (**1‐La**) to 2.632(4) Å (**1‐Nd**); similar contacts between LnCp^R^_2_ fragments and [B(C_6_F_5_)_4_]^−^ anions have previously been seen for [Sc(Cp*)_2_{(C_6_F_5_‐κ^2^‐*F*)B(C_6_F_5_)_3_}]^11^ and [Ln(Cp*)_2_{(μ‐C_6_F_5_‐κ^1^‐*F*)_2_B(C_6_F_5_)_2_}]_2_ (Ln=Pr, Nd).^12^ In the Pr and Nd examples, the two [Ln(Cp*)_2_]^+^ fragments are bridged by two [B(C_6_F_5_)_4_]^−^ anions to give a total of four *meta*‐F⋅⋅⋅Ln interactions, but in the Sc example a single C_6_F_5_ ring binds to one Sc^III^ centre via both a *meta*‐ and a *para*‐F atom. The shortest Ln⋅⋅⋅F distance in **1‐Sm** is 5.693(2) Å, which indicates that Ln⋅⋅⋅F interactions are only observed for the **1‐Ln** series when the Ln^III^ ionic radius is larger than a critical value, when the *bis*‐Cp^ttt^ framework no longer provides sufficient coverage to prevent equatorial interactions with [B(C_6_F_5_)_4_]^−^ anions.

**Table 2:** Selected bond distances and angles of [Ln(Cp^ttt^)_2_]^+^ fragments in **1‐Ln**.

Complex	Ln⋅⋅⋅Cp_cent_ [Å]	Cp_cent_⋅⋅⋅Ln⋅⋅⋅Cp_cent_ [°]	Ln⋅⋅⋅F [Å]
**1‐La**	2.507(2), 2.499(2)	148.13(2)	2.679(9)
**1‐Ce**	2.507(4), 2.478(5)	149.1(2)	2.677(5)
**1‐Pr**	2.479(3), 2.459(4)	149.48(11)	2.664(3)
**1‐Nd**	2.445(5), 2.464(4)	148.10(13)	2.632(4)
**1‐Sm**	2.384(2), 2.392(2)	153.23(6)	–
**1‐Gd** 9	2.364(5), 2.345(5)	150.2(2)	–
**1‐Tb** 10	2.325(4), 2.339(5)	152.2(2)	–
**1‐Dy** 8	2.318(2), 2.314(2)	152.56(7)	–

**Figure 4:**
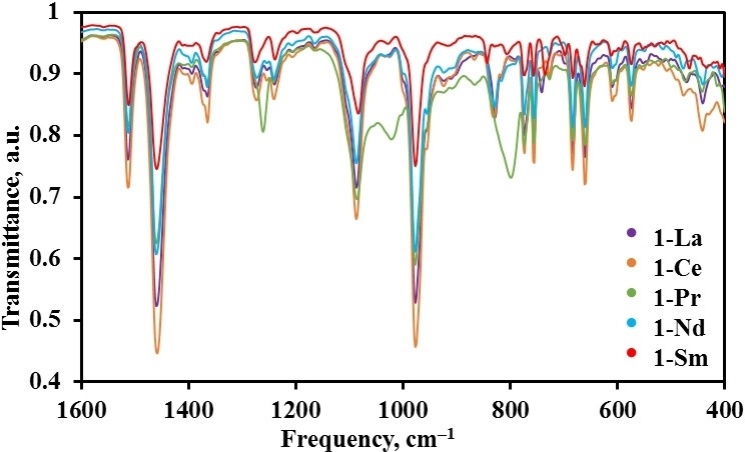
ATR‐IR spectra of **1‐La**, **1‐Ce**, **1‐Pr**, **1‐Nd**, **1‐Sm** in the region 1600–400 cm^−1^ at room temperature.

The ATR‐IR spectra of **1‐Ln** and **2‐Ln** were obtained as microcrystalline powders (spectra of **1‐Ln** compiled in Figure 4; see Supporting Information for full ATR‐IR spectra). As expected, most spectra show broadly similar features, despite the structural differences between **1‐Sm** and other light **1‐Ln**. However, **1‐Pr** is anomalous in exhibiting three additional strong absorptions at 798, 1086 and 1261 cm^−1^; these differences could not be assigned.

**Figure 5:**
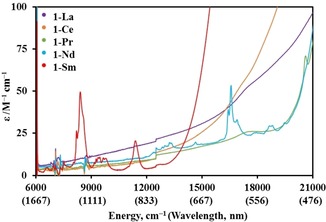
UV/Vis/NIR spectra of **1‐Ln** (ca. 1 mm in CH_2_Cl_2_) from 6000 to 21 000 cm^−1^ at room temperature.

The electronic spectra of **1‐Ln** and **2‐Ln** were obtained at room temperature as 1 mm solutions in DCM (spectra of **1‐Ln** compiled in Figure 5; see Supporting Information for full UV/Vis/NIR spectra). Due to their Laporte‐forbidden nature, f–f transitions are relatively weak,^5^ so charge transfer (CT) bands tailing in from the UV region dominate the spectrum. Dilute solutions of **1‐Nd** and **1‐Sm** are pale green and pale orange, respectively, and some f–f transitions could be clearly observed: **1‐Nd** shows absorptions at $\tilde{\nu }$_max_=16600 cm^−1^ (*ϵ*=46 mol^−1^ dm^3^ cm^−1^), owing to the ^4^I_9/2_→^4^G_5/2_ transitions;^24^ and **1‐Sm** shows absorptions at $\tilde{\nu }$_max_=11 400 cm^−1^ (*ϵ*=20 mol^−1^ dm^3^ cm^−1^) and 8400 cm^−1^ (*ϵ*=50 mol^−1^ dm^−3^ cm^−1^), which likely arise from ^6^H_5/2_→^6^F_11/2_, ^6^F_7/2_ transitions, respectively.^24^ Similar absorptions can be seen in the spectra of the precursors **2‐Nd** and **2‐Sm**.

Complete active space self‐consistent field spin‐orbit (CASSCF‐SO) calculations were performed on unoptimized XRD structures of **1‐Ln** and **2‐Ln** using MOLCAS 8.0^25^ to determine their electronic structures (see Supporting Information for full details). We split our discussion between the Kramers (odd number of unpaired electrons) and non‐Kramers (even number of unpaired electrons) ions for clarity.

### Kramers ions (Ce, Nd and Sm)

In the case of Kramers ions with an odd number of unpaired electrons, there will always be two‐fold electronic degeneracy in zero magnetic field, irrespective of a low‐symmetry CF. Each of these Kramers doublets is a linear combination of *m_J_* projections of the total angular momentum *J*, but can also be defined as an effective *S*=1/2 state. With this definition, the *S*=1/2 states have an effective *g*‐matrix describing the magnetic anisotropy owing to the varying *m_J_* composition of each doublet. The 3×3 *g*‐matrix can always be diagonalised to yield three principal *g*‐values and their corresponding orientations in the molecular frame; here we define the principal *g*‐values such that *g*_1_<*g*_2_<*g*_3_. While the choice of quantisation axis for the CF is irrelevant for any physical observable, it defines how each state is decomposed into linear combinations of *m_J_* projections (*m_J_* is the projection of the total angular momentum *J* along the quantisation axis). In the case of pure *m_J_* states (*m_J_*>1/2), *g*_1_=*g*_2_=0 and *g*_3_=2*g_J_m_J_* where *g_J_* is the Landé *g*‐factor of the *J* multiplet of the Ln^III^ ion. In the general case of mixed *m_J_* states it is useful to define <*J*_z_>, the expectation value of the *J*_z_ operator which measures the projection of *J* on the quantisation axis, to understand the magnetic nature of the states; in the limiting case of a pure *m_J_* state, <*J*_z_>=*m_J_*.

**Figure 6:**
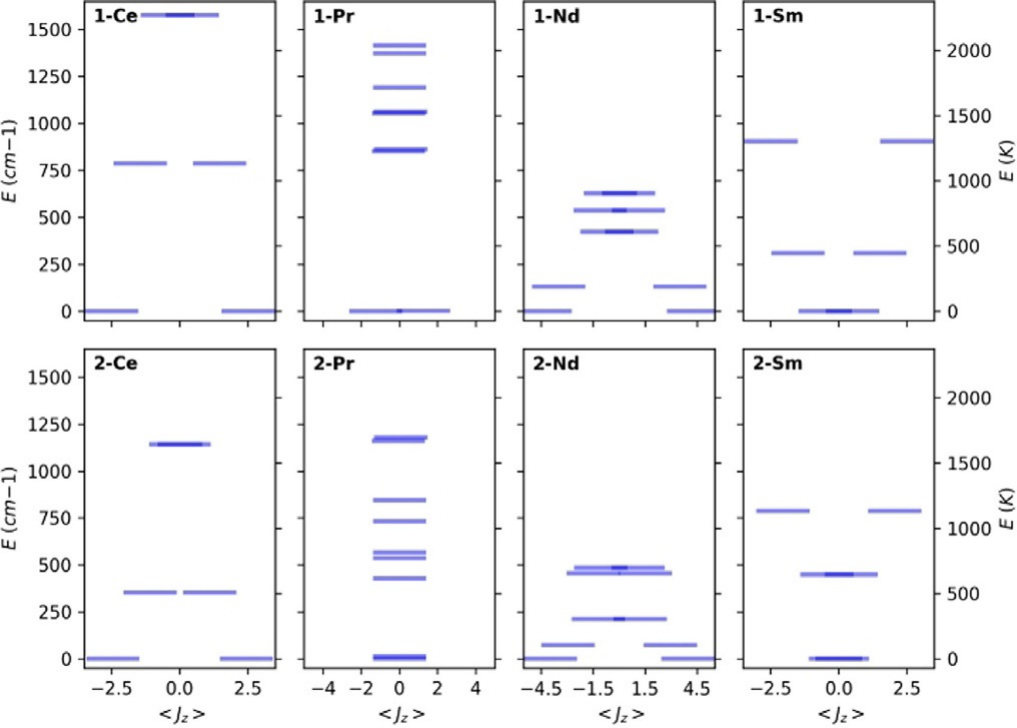
Electronic structure of **1‐Ln** (top) and **2‐Ln** (bottom), calculated with CASSCF‐SO, and shown in the presence of a 0.1 T DC field along the *g_3_* direction of the ground Kramers doublet. <*J*_z_> is the expectation value of *J*_z_, a way to represent mixed *m_J_* states, and is proportional to the magnetic moment.

For **1‐Ce** (Table 3) we observe that the ground Kramers doublet is highly axial (*g*_1_≈*g*_2_<0.1, *g*_3_=4.16), and is thus well‐described as *m_J_*=±5/2 when the CF is quantised along the *g*_3_ direction (Tables 3 and S17). For this choice of quantisation axis, the two excited Kramers doublets are dominated by *m_J_*=±3/2 and ±1/2, respectively. Here, the strong *pseudo*‐linear CF of the *bis*‐Cp^ttt^ ligand set stabilises the oblate spheroid‐shaped electron density of *m_J_*=±5/2 and destabilises the prolate spheroid‐shaped *m_J_*=±1/2 state to give a typical double‐well potential of easy‐axis magnetic anisotropy (Figure 6), as expected from simple electrostatic principles.^16^ This result shows that despite the counter ion binding to the metal ion, its influence on the electronic structure of the Ce^III^ ion is much weaker compared to the *bis*‐Cp^ttt^ CF; this can be verified by repeating the calculations without the [B(C_6_F_5_)_4_]^−^ anion (Table S18), showing only a marginal increase in axiality. For **2‐Ce** however, while the ground doublet is also *m_J_*=±5/2 with the CF defined along the Cp^ttt^–Cp^ttt^ direction (Table S19, Figure S131), the first excited state is almost isotropic and the most excited state also resembles the *m_J_*=±5/2 state when the CF is quantised along the “*pseudo*‐C_3_” direction perpendicular to both the Cp^ttt^–Cp^ttt^ direction and the Ce–Cl vector (Figure S132); this demonstrates that the Cl^−^ anion is competing with the Cp^ttt^ ligands and has a significant influence on the CF.

**Table 3:** Low‐lying electronic structure of **1‐Ce** calculated with CASSCF‐SO in zero‐field. Wavefunction decomposition quantised along the *g*_3_ direction of the ground doublet.

Energy [cm^−1^]	*g*_1_	*g*_2_	*g*_3_	Angle [°]	Wavefunction
0.0	0.05	0.10	4.16	–	100 %$\left|\pm 5/2\right.\rangle$
786.6	0.48	0.52	2.21	2.74	93 %$\left|\pm 3/2\right.\rangle$
1577.1	1.82	3.00	0.84	2.33	94 %$\left|\pm 1/2\right.\rangle$

The electronic structure of **1‐Sm** (Table 4), like **1‐Ce**, can be explained with simple electrostatic principles.^16^ The CF is still dominated by the near‐axial potential of the Cp^ttt^ ligands, but as the electron density distributions of the *m_J_* states of Sm^III^ are in the opposite sense to Ce^III^ (i.e., *m_J_*=±5/2 has a prolate spheroidal electron density and *m_J_*=±1/2 is oblate spheroidal), this makes **1‐Sm** the antithesis of an SMM with the *m_J_*=±1/2, ±3/2 and ±5/2 doublets in ascending energy (Figure 6; Tables 4 and S28). For **2‐Sm**, the *g*_3_ direction of the ground Kramers doublet lies along the “*pseudo*‐C_3_” direction (Figure S140), yet when the CF is quantised along this direction there is strong mixing between *m_J_*=±1/2 and ±3/2 owing to the competition between the Cp^ttt^ and Cl^−^ ligands (Table S29). For both **1‐Sm** and **2‐Sm** the *g*‐values are much reduced from those expected for an isolated ^6^H_5/2_ multiplet; this is due to considerable *J*‐mixing with the low‐lying ^6^H_7/2_ and ^6^H_9/2_ multiplets, rendering a CF model in the basis of a pure *J*=5/2 state an inaccurate representation of the wavefunction.

**Table 4:** Low‐lying electronic structure of **1‐Sm** calculated with CASSCF‐SO in zero‐field. Wavefunction decomposition quantised along the *g*_3_ direction of the ground doublet.

Energy [cm^−1^]	*g*_1_	*g*_2_	*g*_3_	Angle [°]	Wavefunction
0.0	0.18	0.38	1.08	–	100 %$\left|\pm 1/2\right.\rangle$
308.69	0.12	0.13	2.57	2.26	97 %$\left|\pm 3/2\right.\rangle$
905.10	0.06	0.07	4.04	5.11	99 %$\left|\pm 5/2\right.\rangle$

For Nd^III^ the situation is less clear than for Ce^III^ and Sm^III^ because the magnitude of the quadrupolar terms in the expansion of the *m_J_* electron densities are smaller than for the other Ln^III^ ions,^26^ and therefore simple electrostatics do not dominate the electronic structure and mixing of *m_J_* states by the low symmetry CF is more severe. For **1‐Nd** the two lowest lying doublets are 86 % *m_J_*=±9/2 and 86 % *m_J_*=±7/2, respectively, when the CF is quantised along the Cp^ttt^‐Cp^ttt^ direction (Table S25), however the subsequent excited states are quite mixed and the highest‐energy doublet is 86 % *m_J_*=±9/2 when the CF is quantised along the “*pseudo*‐C_3_” direction (based on the nearest F‐atom; Figure S136). Removal of the counterion reduces the axiality of the most excited state (Table S26), however this does not change the overall picture of the electronic structure. This suggests that it is the bent arrangement of the Cp^ttt^ rings, and not the influence of the [B(C_6_F_5_)_4_]^−^ counterion, that forces the magnetic anisotropy of the most excited state to be perpendicular to that of the ground state, further highlighting the minimal electronic influence of the bulky anion. The electronic structure of **2‐Nd** is generally similar to that of **1‐Nd**, however the mixing is more severe (Table S27) and is not discussed further.

### Non‐Kramers ions (Pr)

In the case of non‐Kramers ions, the action of a low‐symmetry CF can completely remove the degeneracy of the electronic states, although it does not have to do so (e.g. in high symmetry environments). In the case of a near‐axial CF, there will be a number of *pseudo*‐doublets that are well described as pure *m_J_* functions and a singlet state corresponding to *m_J_*=0; the departure from axiality determines the degree to which the *pseudo*‐doublets are split into singlets. For *pseudo*‐doublets only a single principal *g*‐value can be defined and the other two are zero (note that this does not necessarily indicate a pure *m_J_* state); thus, <*J*_z_> is a useful indicator for non‐Kramers ions.

The situation for **1‐Pr** (Table 5) is similar to that of **1‐Ce**, where the ground *pseudo*‐doublet is well defined as *m_J_*=±4 when the CF is quantised along the Cp^ttt^‐Cp^ttt^ direction (Tables 5 and S20), however the low‐symmetry CF leads to mixing between the opposing *m_J_* projections and small splittings in the *pseudo*‐doublets at low energies and substantial splittings at the more energetic end of the spectrum. These mixings can be partially lifted by applying a small magnetic field along the *g*_3_ direction of the ground *pseudo*‐doublet (Table S21). Removal of the counterion in the CASSCF‐SO calculations for **1‐Pr** results in larger energy gaps between the CF states and reduces the splitting within the *pseudo*‐doublets at higher energies, but generally does not have a large effect on the electronic structure (Table S22). For **2‐Pr**, with an extra competitive element in the CF (the Cl^−^ ion), the resulting electronic structure is nearly all singlets (Table S23), and application of a small magnetic field is inconsequential (Table S24).

**Table 5:** Low‐lying electronic structure of **1‐Pr** calculated with CASSCF‐SO in zero‐field. Wavefunction decomposition quantised along the *g*_3_ direction of the ground doublet.

Energy [cm^−1^]	*g*_3_	Angle [°]	Wavefunction
0.00	6.18	–	50 %$\left|-4\right.\rangle$ +50 %$\left|+4\right.\rangle$
0.31			50 %$\left|-4\right.\rangle$ +50 %$\left|+4\right.\rangle$
852.05	4.76	10.3	49 %$\left|-3\right.\rangle$ +49 %$\left|+3\right.\rangle$
863.29			48 %$\left|-3\right.\rangle$ +48 %$\left|+3\right.\rangle$
1050.72	3.28	20.8	48 %$\left|-2\right.\rangle$ +48 %$\left|+2\right.\rangle$
1054.69			47 %$\left|-2\right.\rangle$ +47 %$\left|+2\right.\rangle$
1210.68	–	–	49 %$\left|-1\right.\rangle$ +49 %$\left|+1\right.\rangle$
1377.28	–	–	49 %$\left|-1\right.\rangle$ +49 %$\left|+1\right.\rangle$
1403.77	–	–	97 %$\left|0\right.\rangle$

To experimentally probe the nature of the ground states, we have performed cryogenic electron paramagnetic resonance (EPR) spectroscopy on the Kramers ion analogues (Ce^III^, Nd^III^ and Sm^III^) of **1‐Ln** and **2‐Ln**. We note generally that the intensities of the signals are very weak, however that there is excellent agreement between experiment and theory (Table 6; Figures S126–S129). We do not observe a signal for **1‐Nd** or **2‐Sm**: the former is predicted to be EPR silent from CASSCF‐SO (the ground doublet is dominated by *m_J_*=±9/2 and has very small *g*_1_ and *g*_2_, thus there is a vanishing component of Δ*m_J_*=±1 between the two states, which is the EPR selection rule), however the latter is apparently too weak or is broadened beyond detection.

**Table 6:** Calculated and measured *g*‐values for the Kramers analogues of **1‐Ln** and **2‐Ln**.

Complex	CASSCF‐SO	Experiment
**1‐Ce**	4.16	4.22
**2‐Ce**	4.09	4.19
**1‐Nd**	6.32^[a]^	Silent
**2‐Nd**	6.00	6.10
**1‐Sm**	1.08	≈1.5
**2‐Sm**	0.71	Silent

[a] **1‐Nd** is predicted to be EPR silent.

The χ_M_T products for **1‐Ln** and **2‐Ln** show a gradual decrease with temperature for all samples (Figure S109), however the data for **2‐Pr** decrease rapidly at lower temperatures, and those for **1‐Pr** decrease slowly below 20 K. For **2‐Pr** this is due to a singlet ground state, which is confirmed by magnetisation measurements (Figure S111) and CASSCF‐SO calculations (Table S23), but for **1‐Pr** the experimental decrease is much more substantial than predicted by CASSCF‐SO, suggesting that the calculations have underestimated the splitting within the ground pseudo‐doublet on the order of a few cm^−1^ (Table S20). Generally, these magnetic data are in good agreement with CASSCF‐SO‐calculated electronic structures, however we note that due to the small magnetic moments of the light Ln^III^ ions (where the SO coupling gives *J*=|*L*−*S*| ground multiplets) the experimental data are sensitive to small errors in sample masses and diamagnetic corrections. The data for **1‐Sm** and **2‐Sm** are the most susceptible to this, as they feature not only the lowest magnetic moments, but also because they are highly sensitive to *J* mixing (see above) such that the calculations only reveal an approximate electronic structure.

**Figure 7:**
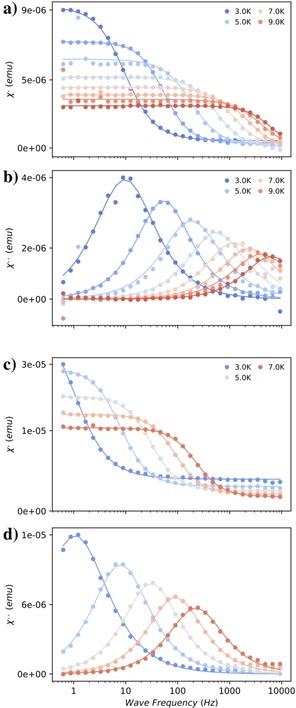
In‐phase and out‐of‐phase AC susceptibilities for **1‐Ce** (a,b) and **1‐Nd** (c,d) in a 0.1 T DC field. Solid lines are fits to the generalized Debye model.

We were curious if the anomalous Raman exponents we observed previously for the heavy [Ln(Cp^ttt^)_2_]^+^ series^8^, ^9^, ^10^ could also be observed for these light Ln^III^ analogues. Therefore, we investigated the Kramers ions with AC susceptibility studies to examine their magnetisation dynamics (Figures 7 and S114–S125). Only in the case of **2‐Ce** was it possible to observe slow magnetic relaxation in zero DC field, and therefore we employed a 0.1 T DC field for all measurements; however, no slow relaxation could be detected for **1‐Sm** and **2‐Sm**. Only for **2‐Nd** is relaxation via an Orbach mechanism plausible (Figure S125), giving an effective barrier of *U*_eff_=51.2 cm^−1^ with *τ*_0_=9.64×10^−8^ s; CASSCF‐SO predicts a first excited state at around 72 cm^−1^, so this is not unreasonable. In all other cases a Raman mechanism is the best model for the data, and indeed is also a possible explanation for **2‐Nd**. For both pairs of **1‐Ce** vs. **2‐Ce** and **1‐Nd** vs. **2‐Nd**, the Raman exponent is smaller for **1‐Ln** than for **2‐Ln** where the Cl^−^ is bound, similar to the trend we have observed previously for the heavy Ln metallocenium cations (Table 7),^9^, ^10^ which supports our previous conclusion that the absence of monodentate ligands in [Ln(Cp^ttt^)_2_]^+^ is responsible for lowered Raman exponents. However, the exponents for **1‐Ce** and **1‐Nd** are considerably larger than for **1‐Tb**, **1‐Dy** and **1‐Ho**, which suggests that there could be an influence of the proximate [B(C_6_F_5_)_4_]^−^ anion on the relaxation dynamics of these species.

**Table 7:** Raman relaxation parameters from AC magnetometry.

Complex	*H* [T]	*C* [s^−1^ K^−*n*^]	*n*
**1‐Ce**	0.1	0.0308	5.37
**2‐Ce**	0.1	0.00475	6.48
**1‐Nd**	0.1	0.00117	6.29
**2‐Nd**	0.1	0.000300	8.74
**1‐Tb** 10	0	24	1.2
**[Tb(Cp^ttt^)_2_(BH_4_)]** 10	0.1	0.26	4.6
**1‐Dy** 8	0	0.000001664	2.151
**2‐Dy** 9	0.1	0.0023	5.3
**1‐Ho** 9	0.1	3.4	2.9
**2‐Sm** 9	0.1	0.015	8.5
**2‐Yb** 9	0.1	0.017	7.1

## Conclusions

We have completed a series of analogous Ln metallocenium cations [Ln(Cp^ttt^)_2_]^+^, with the exception of Eu and Pm. In the case of the early Ln (Ln=La‐Nd) the [B(C_6_F_5_)_4_]^−^ counter‐ions weakly coordinate to the metal centre through a *meta*‐fluorine atom in the solid state, as the metals are large enough to incorporate this additional interaction; these equatorial interactions are absent in the solid state for the later Ln (Ln=Sm, Gd–Lu) analogues which contain smaller Ln centres. However, in the solution phase these interactions could not be detected by VT ^19^F NMR spectroscopy, indicating that isolated Ln metallocenium cations are present in fluid solution.^23^ Analysis of the electronic structures of [Ln(Cp^ttt^)_2_]^+^ for the early Ln suggests that the weak equatorial *meta*‐fluorine interaction has little effect on the axiality of these systems. Measurement of the relaxation dynamics shows a consistent picture across the [Ln(Cp^ttt^)_2_]^+^ series, where the absence of monodentate ligands leads to lower Raman exponents than when they are present, indicating that this effect is a hallmark of all Ln metallocenium cations. We cannot rule out the influence of [B(C_6_F_5_)_4_]^−^ on the relaxation dynamics of **1‐Ce** and **1‐Nd**, and suggest that the effect of weak equatorial donors on magnetic relaxation mechanisms may be explored in the future by using different Cp^R^ ligands and a range of weakly coordinating anions.

## Experimental Section

**Materials and methods**. All manipulations were carried out using standard Schlenk line and glove box techniques under dry argon. Solvents were passed through columns containing alumina or were dried by refluxing over K or CaH_2_ (DCM), and were stored over K mirrors or 4 Å molecular sieves (THF, DCM) and degassed before use. For NMR spectroscopy, [D_6_]benzene and [D_8_]toluene were dried by refluxing over K, and [D_2_]DCM was dried by refluxing over CaH_2_. NMR solvents were degassed by three freeze‐pump‐thaw cycles, and vacuum‐transferred before use. Anhydrous LnCl_3_ were purchased from Alfa Aesar and were used as received. KCp^ttt^,^27^ [H(SiEt_3_)_2_][B(C_6_F_5_)_4_]^18^ and [Eu(Cp^ttt^)_2_]^20^ were prepared according to literature methods, whilst **2‐Ln** (Ln=Pr, Nd) were made by a modification of published procedures.^19^ ^1^H (400 and 500 MHz), ^13^C{^1^H} (100 and 125 MHz), ^13^C{^19^F} (125 MHz),^11^B{^1^H} (128 and 160 MHz), and ^19^F{^1^H} (376 MHz) NMR spectra were obtained on Avance III 400 or 500 MHz spectrometers at 298 K. UV/Vis/NIR spectroscopy was performed on samples in Youngs tap‐appended 10 mm path length quartz cuvettes on an Agilent Technologies Cary Series UV/Vis/NIR spectrophotometer at 175–3300 nm. ATR‐Fourier transform infrared (ATR‐FTIR) spectra were recorded as microcrystalline powders using a Bruker Tensor 27 spectrometer. Elemental analyses were performed by Mrs Anne Davies and Mr Martin Jennings at The University of Manchester School of Chemistry Microanalysis Service, Manchester, UK. General synthetic procedures for **1‐Ln** and **2‐Ln** are given below; full details are in the Supporting Information.

**[Ln(Cp^ttt^)_2_{(C_6_F_5_‐κ^1^‐*F*)B(C_6_F_5_)_3_}] (La=La‐Nd), [Sm(Cp^ttt^)_2_] [B(C_6_F_5_)_4_] (1‐Ln)**: Benzene (15 mL) was added to a mixture of [H(SiEt_3_)_2_][B(C_6_F_5_)_4_] (0.5–0.8 mmol) and **2‐Ln** (0.5–0.8 mmol) at room temperature. The mixture was stirred for 16 hours and a precipitate formed. The volatiles were removed in vacuo to give a powder, which was washed with hexane (10–15 mL) and benzene (10–15 mL). In some cases, the crude material was dissolved in DCM (typically 1.4–3 mL) at −78 °C, and layered with 1–1.5 equiv of hexane. Storage at −25 °C overnight gave crystals of **1‐Ln** (Ln=Ce, Pr, Nd). In other cases, the crude material was dissolved in hot toluene (5 mL). Storage at room temperature gave **1‐Ln** (Ln=La, Sm).

**1‐La**: Colourless crystals (0.432 g, 67 %). Anal. Calcd (%) for C_58_H_58_BF_20_La: C, 54.22; H, 4.55; Found: C, 52.22; H, 4.15. ^1^H NMR ([D_2_]DCM, 400 MHz, 298 K): *δ*=1.38 (s, 18 H, Cp‐C(C*H*_3_)_3_), 1.46 (s, 36 H, Cp‐C(C*H*_3_)_3_), 6.26 ppm (s, 4 H, Cp‐C*H*). ^11^B{^1^H} NMR ([D_2_]DCM, 128 MHz, 298 K): *δ*=−16.63 ppm (s). ^13^C{^1^H} NMR ([D_2_]DCM, 125 MHz, 298 K): *δ*=29.86 and 30.00 (C(*C*H_3_)_3_), 30.53 (C(*C*H_3_)_3_), 31.03 and 31.18 (*C*(CH_3_)_3_), 32.82 (*C*(CH_3_)_3_), 135.96 (Cp‐*C*H), 147.78 (Cp‐*C*), 149.70 ppm (Cp‐*C*). ^13^C{^19^F} NMR ([D_2_]DCM, 125 MHz, 298 K): *δ*=124.26 (q, B‐C_*ipso*_, ^1^ *J*_BC_=51.8 Hz), 136.91 (s, *m*‐*C*F), 138.79 (s, *p*‐CF), 148.71 ppm (s, *o*‐*C*F). ^19^F{^1^H} NMR ([D_2_]DCM, 376 MHz, 298 K): *δ*=−166.98 (s, *m*‐*F*), −162.89 (s, *p*‐*F*), −132.67 ppm (s, *o*‐*F*). The low solubility of **1‐La** in [D_6_]benzene precluded assignment of ^1^H and ^13^C{^1^H} NMR spectra in this solvent. ^11^B{^1^H} NMR ([D_6_]benzene, 160 MHz, 298 K): *δ*=−16.00 ppm (s). ^19^F{^1^H} NMR ([D_6_]benzene, 376 MHz, 298 K): *δ*=−165.32 (s, *m*‐*F*), −160.90 (s, *p*‐*F*), −131.66 ppm (s, *o*‐*F*). FTIR (ATR, microcrystalline): $\tilde{\nu }$ =2967 (br, m), 2874 (w), 2819 (w), 1643 (m), 1513 (s), 1459 (s), 1365 (m), 1273 (m), 1240 (m), 1087 (s), 977 (s), 924 (w), 828 (s), 773 (s), 756 (s), 683 (s), 660 (s), 609 (m), 574 (m), 470 (w), 440 cm^−1^ (w).

**1‐Ce**: Yellow crystals (0.137 g, 21 %). Anal. Calcd (%) for C_58_H_58_BF_20_Ce⋅1.5CH_2_Cl_2_: C, 50.56; H, 4.35; Found: C, 50.90; H, 4.18. *μ*_eff_ (Evans method, 298 K, [D_2_]DCM): 2.12 μ_B_. ^1^H NMR ([D_2_]DCM, 500 MHz, 298 K): *δ*=−13.26 (br, 18 H, *ν*_1/2_∼450 Hz, Cp‐C(C*H*_3_)_3_), −7.93 ppm (br, 36 H, *ν*_1/2_∼920 Hz, Cp‐C(C*H*_3_)_3_); no other signals observed. ^11^B{^1^H} NMR ([D_2_]DCM, 160 MHz, 298 K): *δ*=−18.02 ppm (s). The paramagnetism of **1‐Ce** precluded assignment of its ^13^C{^1^H} NMR spectrum. ^19^F{^1^H} NMR ([D_2_]DCM, 376 MHz, 298 K): *δ*=−170.26 (s, *m*‐*F*), −164.33 (s, *p*‐*F*), −134.61 ppm (s, *o*‐*F*). FTIR (ATR, microcrystalline): $\tilde{\nu }$ =2963 (br, m), 2871 (w), 2821 (w), 1643 (m), 1512 (s), 1459 (s), 1365 (m), 1273 (w), 1241 (w), 1087 (s), 976 (s), 924 (w), 867 (w), 830 (m), 773 (s), 756 (s), 683 (s), 660 (s), 609 (m), 574 (m), 441 cm^−1^ (w).

**1‐Pr**: Yellow crystals (0.255 g, 40 %). Anal. Calcd (%) for C_58_H_58_BF_20_Pr⋅2 CH_2_Cl_2_: C, 49.47; H, 4.29; Found: C, 47.68; H, 4.27. *μ*_eff_ (Evans method, 298 K, [D_2_]DCM): 2.70 μ_B_. The paramagnetism of **1‐Pr** precluded assignment of its ^1^H and ^13^C{^1^H} NMR spectra. ^11^B{^1^H} NMR ([D_2_]DCM, 128 MHz, 298 K): *δ*=−19.42 ppm (s). ^19^F{^1^H} NMR ([D_2_]DCM, 376 MHz, 298 K): *δ*=−172.86 (s, *m*‐*F*), −166.16 (s, *p*‐*F*), −136.51 ppm (s, *o*‐*F*). FTIR (ATR, microcrystalline): $\tilde{\nu }$ =2963 (m), 2909 (w), 2871 (w), 1643 (m), 1460 (s), 1365 (m), 1261 (s), 1242 (w), 1086 (s), 1021 (m), 977 (s), 798 (s), 774 (m), 755 (m), 683 (s), 660 (s), 609 (w), 573 (m), 473 (w), 441 cm^−1^ (w).

**1‐Nd**: Green crystals (0.381 g, 59 %). Anal. Calcd (%) for C_58_H_58_BF_20_Nd⋅2 CH_2_Cl_2_: C, 49.36; H, 4.28; Found: C, 49.51; H, 4.17. *μ*_eff_ (Evans method, 298 K, [D_2_]DCM): 3.42 μ_B_. ^1^H NMR ([D_2_]DCM, 400 MHz, 298 K): *δ*=−17.88 (br, 18 H, *ν*_1/2_∼310 Hz, Cp‐C(C*H*_3_)_3_), −11.94 ppm (br, 36 H, *ν*_1/2_∼700 Hz, Cp‐C(C*H*_3_)_3_); no other signals observed. ^11^B{^1^H} NMR ([D_2_]DCM, 128 MHz, 298 K): *δ*=−18.55 ppm (s). The paramagnetism of **1‐Nd** precluded assignment of its ^13^C{^1^H} NMR spectrum. ^19^F{^1^H} NMR ([D_2_]DCM, 376 MHz, 298 K): *δ*=−170.31 (s, *m*‐*F*), −164.92 (s, *p*‐*F*), −135.17 ppm (s, *o*‐*F*). FTIR (ATR, microcrystalline): $\tilde{\nu }$ =2967 (br, m), 2875 (w), 2820 (w), 1643 (m), 1512 (s), 1460 (s), 1394 (w), 1365 (m), 1254 (w), 1241 (w), 1087 (s), 977 (s), 955 (w), 924 (w), 829 (m), 773 (s), 756 (s), 683 (s), 660 (s), 609 (w), 574 (m), 477 (w), 440 cm^−1^ (w).

**1‐Sm**: Red crystals (0.790 g, 69 %). Anal. Calcd (%) for C_58_H_58_BF_20_Sm: C, 53.74; H, 4.51; Found: C, 53.88; H, 4.49. *μ*_eff_ (Evans method, 298 K, [D_2_]DCM): 1.90 μ_B_. The paramagnetism of **1‐Sm** precluded assignment of its ^13^C{^1^H} NMR spectrum. ^1^H NMR ([D_2_]DCM, 400 MHz, 298 K): *δ*=−1.37 (s, 36 H, Cp‐C(C*H*_3_)_3_), 19.69 ppm (s, 2 H, Cp‐C*H*); no other signals observed. ^11^B{^1^H} NMR ([D_2_]DCM, 128 MHz, 298 K): *δ*=−16.76 ppm (s). ^19^F{^1^H} NMR ([D_2_]DCM, 376 MHz, 298 K): *δ*=−167.89 (s, *m*‐*F*), −163.76 (t, *J*_FF_=20.4 Hz, *p*‐*F*), −133.13 ppm (s, *o*‐*F*). ^1^H NMR ([D_6_]benzene, 400 MHz, 298 K): *δ*=−2.58 (s, 18 H, Cp‐C(C*H*_3_)_3_), −0.90 (s, 36 H, Cp‐C(C*H*_3_)_3_), 18.80 ppm (s, 4 H, Cp‐C*H*). ^11^B{^1^H} NMR ([D_6_]benzene, 128 MHz, 298 K): *δ*=−16.32 ppm (s). ^19^F{^1^H} NMR ([D_6_]benzene, 376 MHz, 298 K): *δ*=−168.96 (s, *m*‐*F*), −162.02 (s, *p*‐*F*), −131.93 ppm (s, *o*‐*F*). FTIR (ATR, microcrystalline): $\tilde{\nu }$ =2964 (br, m), 2872 (w), 2796 (w), 1642 (m), 1512 (s), 1459 (s), 1367 (m), 1276 (m), 1239 (m), 1083 (s), 977 (s), 843 (w), 806 (w), 774 (m), 756 (m), 735 (m), 683 (m), 661 (s), 611 (w), 574 (w), 466 (w), 449 cm^−1^ (w).

**[Ln(Cp^ttt^)_2_(Cl)] (Ln=La, Ce, Pr, Nd, Sm) (2‐Ln)**: THF (30 mL) was added to a Teflon tap‐appended ampoule containing a pre‐cooled (−78 °C) mixture of LnCl_3_ (2 mmol) and KCp^ttt^ (4 mmol). The reaction mixture was allowed to warm to room temperature and heated in an oil bath at 80 °C for 16 hours. The solvent was removed in vacuo and toluene (30 mL) was added. The reaction mixture was heated in an oil bath at 120 °C for 16 hours. The resultant suspension was allowed to settle for 3 hours and filtered. The solution was concentrated to 2 mL and stored at 8 °C to afford crystals of **2‐Ln**.

**2‐La**: Colourless crystals (0.395 g, 31 %). Anal. Calcd (%) for C_34_H_58_LaCl: C, 63.69; H, 9.12; Found: C, 63.57; H, 9.30. ^1^H NMR ([D_6_]benzene, 400 MHz, 298 K): *δ*=1.25 (s, 18 H, C(C*H*_3_)_3_), 1.52 (s, 36 H, C(C*H*_3_)_3_), 6.51 ppm (s, 4 H, Cp‐*H*). ^13^C{^1^H} NMR ([D_6_]benzene, 100 MHz, 298 K): *δ*=31.19 (C(*C*H_3_)_3_), 32.86 (*C*(CH_3_)_3_), 34.50 (*C*(CH_3_)_3_), 34.68 (C(*C*H_3_)_3_), 114.78 (Cp‐*C*H), 138.17 (Cp‐*C*), 139.58 ppm (Cp‐*C*). FTIR (ATR, microcrystalline): $\tilde{\nu }$ =2956 (s), 2904 (w), 2869 (w), 1461 (m), 1389 (m), 1361 (s), 1260 (s), 1241 (m), 1091 (br, w), 1016 (s), 866 (w), 797 (s), 678 (s), 590 (w), 566 (w), 551 (w), 436 cm^−1^ (w).

**2‐Ce**: Orange crystals (0.651 g, 51 %). Anal. Calcd (%) for C_34_H_58_CeCl: C, 63.57; H, 9.10. Found: C, 63.60; H, 9.22. *μ*_eff_ (Evans method, 298 K, [D_6_]benzene): 2.34 μ_B_. The paramagnetism of **2‐Ce** precluded assignment of its ^13^C{^1^H} NMR spectrum. ^1^H NMR ([D_6_]benzene, 400 MHz, 298 K): *δ*=−13.06 (s, 18 H, C(C*H*_3_)_3_), −2.53 ppm (s, 36 H, C(C*H*_3_)_3_); no other signals observed. FTIR (ATR, microcrystalline): $\tilde{\nu }$ =2954 (s), 2904 (m), 2868 (w), 1460 (s), 1389 (s), 1358 (s), 1241 (s), 1165 (m), 1001 (s), 958 (m), 816 (s), 774 (s), 678 (s), 566 (w), 436 cm^−1^ (s).

**2‐Pr**: Pale green crystals (0.672 g, 52 %). Anal. Calcd (%) for C_34_H_58_PrCl: C, 63.49; H, 9.09; Found: C, 63.37; H, 9.22. *μ*_eff_ (Evans method, 298 K, [D_6_]benzene): 3.35 μ_B_. The paramagnetism of **2‐Pr** precluded assignment of its ^13^C{^1^H} NMR spectrum. ^1^H NMR ([D_6_]benzene, 400 MHz, 298 K): *δ*=−36.08 (s, 18 H, C(C*H*_3_)_3_), −7.74 ppm (br, 36 H, *ν*_1/2_∼750 Hz, C(C*H*_3_)_3_); no other signals observed. FTIR (ATR, microcrystalline): $\tilde{\nu }$ =2955 (s), 2905 (m), 2869 (w), 1460 (s), 1389 (s), 1359 (s), 1241 (s), 1166 (m), 1001 (s), 959 (m), 832 (m), 818 (s), 775 (s), 679 (s), 567 (m), 437 cm^−1^ (s).

**2‐Nd**: Blue crystals (0.593 g, 46 %). Anal. Calcd (%) for C_34_H_58_NdCl: C, 63.16; H, 9.04; Found: C, 61.22; H, 9.02. The paramagnetism of **2‐Nd** precluded assignment of its ^13^C{^1^H} NMR spectrum. *μ*_eff_ (Evans method, 298 K, [D_6_]benzene): 3.55 μ_B_. ^1^H NMR ([D_6_]benzene, 400 MHz, 298 K): *δ*=−18.95 (s, 18 H, C(C*H*_3_)_3_), −5.58 ppm (s, 36 H, C(C*H*_3_)_3_); no other signals observed FTIR (ATR, microcrystalline): $\tilde{\nu }$ =2955 (s), 2907 (m), 2870 (w), 1460 (s), 1389 (s), 1358 (s), 1241 (s), 1166 (m), 1001 (s), 833 (s), 820 (s), 775 (s), 679 (s), 439 cm^−1^ (s).

**2‐Sm**: Yellow crystals (0.656 g, 50 %). Anal. Calcd (%) for C_34_H_58_SmCl: C, 62.57; H, 8.96; Found: C, 60.93; H, 9.19. *μ*_eff_ (Evans method, 298 K, [D_6_]benzene): 1.69 μ_B_. The paramagnetism of **2‐Sm** precluded assignment of its ^13^C{^1^H} NMR spectrum. ^1^H NMR ([D_6_]benzene, 400 MHz, 298 K): *δ*=−6.01 (s, 18 H, C(C*H*_3_)_3_), 0.55 (s, 36 H, C(C*H*_3_)_3_), 19.80 ppm (s, 4 H, Cp‐*H*). FTIR (ATR, microcrystalline): $\tilde{\nu }$ =2956 (s), 2905 (m), 2870 (w), 1460 (s), 1389 (s), 1356 (s), 1241 (s), 1221 (w), 1166 (s), 1000 (s), 959 (m), 836 (w), 824 (s), 776 (s), 679 (s), 591 (w), 438 cm^−1^ (s).

## Conflict of interest

The authors declare no conflict of interest.

## Supporting information

As a service to our authors and readers, this journal provides supporting information supplied by the authors. Such materials are peer reviewed and may be re‐organized for online delivery, but are not copy‐edited or typeset. Technical support issues arising from supporting information (other than missing files) should be addressed to the authors.

Supplementary
