# Supplementary material for: Light Lanthanide Metallocenium Cations Exhibiting Weak Equatorial Anion Interactions
Source: Chemistry. 2019 May 13;25(32):7749–58. doi: 10.1002/chem.201901167 (PMC6637382; doi:10.1002/chem.201901167)
Supplement: Supplementary file 1 — Supplementary [file CHEM-25-7749-s001.pdf]

CHEMISTRY
A European Journal

Supporting Information

Light Lanthanide Metallocenium Cations Exhibiting Weak Equatorial Anion Interactions

Jingjing Liu, Daniel Reta, Jake A. Cleghorn, Yu Xuan Yeoh, Fabrizio Ortu, Conrad A. P. Goodwin, Nicholas F. Chilton,* and David P. Mills*[a]

chem_201901167_sm_miscellaneous_information.pdf

Contents

1. Synthesis of 1-Ln, 2-Ln, 3 and 4 S2

2. Crystallography S8

3. Molecular structures of complexes 1-La, 1-Ce, 1-Pr, 1-Nd and 1-Sm•(C_7H_8)$_{1.5}$ S14

4. Molecular structures of complexes 2-La, 2-Ce, 2-Pr, 2-Nd and 2-Sm S17

5. Molecular structures of complexes 3 and 4 S20

6. NMR spectroscopy S21

7. ATR-IR spectroscopy S57

8. UV-vis-NIR spectroscopy S63

9. Magnetic measurements S70

10. EPR spectroscopy S86

11. CASSCF-SO electronic structure S89

12. References S102

1. Synthesis of 1-Ln, 2-Ln, 3 and 4

[La(Cp^{ttt})$_2${(C$_6$F$_5$-κ^1-*F*)B(C$_6$F$_5$)$_3$}] (1-La). Benzene (15 mL) was added to a mixture of [H(SiEt$_3$)$_2$][B(C$_6$F$_5$)$_4$] (0.455 g, 0.5 mmol) and [La(Cp^{ttt})$_2$(Cl)l] (0.321 g, 0.5 mmol) at room temperature to give a pale-yellow reaction mixture. The mixture was stirred for 16 hours, forming a pale-yellow precipitate. The volatiles were removed under vacuum to give a pale-yellow powder, which was washed with hexane (10 mL) and benzene (10 mL). The crude material was dissolved in hot toluene (5 mL). Storage at room temperature afforded **1-La** as colourless crystals (0.432 g, 67%). Anal. Calcd (%) for $C_{58}H_{58}BF_{20}La$: C, 54.22; H, 4.55; Found: C, 52.22; H, 4.15. ^{1}H NMR ([D$_2$]DCM, 400 MHz, 298 K): δ = 1.38 (s, 18H, Cp-C(C*H*$_3$)$_3$), 1.46 (s, 36H, Cp-C(C*H*$_3$)$_3$), 6.26 (s, 4H, Cp-C*H*). ^{11}B{^{1}H} NMR ([D$_2$]DCM, 128 MHz, 298 K): δ = –16.63 (s). ^{13}C{^{1}H} NMR ([D$_2$]DCM, 125 MHz, 298 K): δ = 29.86 and 30.00 (C(*C*H$_3$)$_3$), 30.53 (C(*C*H$_3$)$_3$), 31.03 and 31.18 (*C*(CH$_3$)$_3$), 32.82 (*C*(CH$_3$)$_3$), 135.96 (Cp-*C*H), 147.78 (Cp-*C*), 149.70 (Cp-*C*). ^{13}C{^{19}F} NMR ([D$_2$]DCM, 125 MHz, 298 K): δ = 124.26 (q, B-C$_{ipso}$, $^1J_{BC}$ = 51.77 Hz), 136.91 (s, *m*-*C*F), 138.79 (s, *p*-CF), 148.71 (s, *o*-*C*F). ^{19}F{^{1}H} NMR ([D$_2$]DCM, 376 MHz, 298 K): δ = −166.98 (s, *m-F*), −162.89 (s, *p-F*), −132.67 (s, *o-F*). The low solubility of **1-La** in [D$_6$]benzene precluded assignment of ^{1}H and ^{13}C{^{1}H} NMR spectra in this solvent. ^{11}B{^{1}H} NMR ([D$_6$]benzene, 160 MHz, 298 K): δ = –16.00 (s). ^{19}F{^{1}H} NMR ([D$_6$]benzene, 376 MHz, 298 K): δ = −165.32 (s, *m-F*), −160.90 (s, *p-F*), −131.66 (s, *o-F*). FTIR (ATR, microcrystalline): $\tilde{\nu}$ = 2967 (br, m), 2874 (w), 2819 (w), 1643 (m), 1513 (s), 1459 (s), 1365 (m), 1273 (m), 1240 (m), 1087 (s), 977 (s), 924 (w), 828 (s), 773 (s), 756 (s), 683 (s), 660 (s), 609 (m), 574 (m), 470 (w), 440 (w) cm^{-1}.

[Ce(Cp^{ttt})$_2${(C$_6$F$_5$-κ^1-*F*)B(C$_6$F$_5$)$_3$}] (1-Ce). Benzene (15 mL) was added to a mixture of [H(SiEt$_3$)$_2$][B(C$_6$F$_5$)$_4$] (0.455 g, 0.5 mmol) and [Ce(Cp^{ttt})$_2$(Cl)] (0.321 g, 0.5 mmol) at room temperature to give a reddish brown reaction mixture. The mixture was stirred for 16 hours, forming an orange precipitate. The volatiles were removed under vacuum to give an orange powder, which was washed with hexane (10 mL) and benzene (10 mL). The crude material was dissolved in DCM (3 mL) at –78 °C, filtered, and layered with hexane (3 mL). Storage at –25 °C afforded **1-Ce** as yellow crystals (0.137 g, 21%). Anal. Calcd (%) for $C_{58}H_{58}BF_{20}Ce\cdot1.5CH_2Cl_2$: C, 50.56; H, 4.35; Found: C, 50.90; H, 4.18. μ_{eff} (Evans method, [D$_2$]DCM, 298 K): 2.12 μ_B. ^{1}H NMR ([D$_2$]DCM, 500 MHz, 298 K): δ = –13.26 (br, 18H, $\nu_{1/2}$ ~ 450 Hz, Cp-C(C*H*$_3$)$_3$), –7.93 (br, 36H, $\nu_{1/2}$ ~ 920 Hz, Cp-C(C*H*$_3$)$_3$); no other signals observed. ^{11}B{^{1}H} NMR ([D$_2$]DCM, 160 MHz, 298 K): δ = –18.02 (s). The paramagnetism of **1-Ce** precluded assignment of its ^{13}C{^{1}H} NMR spectrum. ^{19}F{^{1}H} NMR

([D$_2$]DCM, 376 MHz, 298 K): δ = −170.26 (s, *m-F*), −164.33 (s, *p-F*), −134.61 (s, *o-F*). FTIR (ATR, microcrystalline): $\tilde{\nu}$ = 2963 (br, m), 2871 (w), 2821 (w), 1643 (m), 1512 (s), 1459 (s), 1365 (m), 1273 (w), 1241 (w), 1087 (s), 976 (s), 924 (w), 867 (w), 830 (m), 773 (s), 756 (s), 683 (s), 660 (s), 609 (m), 574 (m), 441 (w) cm^{-1}.

[Pr(Cp^{ttt})$_2${(C$_6$F$_5$-κ^1-*F*)B(C$_6$F$_5$)$_3$}] (1-Pr). Benzene (15 mL) was added to a mixture of [H(SiEt$_3$)$_2$][B(C$_6$F$_5$)$_4$] (0.455 g, 0.5 mmol) and [Pr(Cp^{ttt})$_2$(Cl)] (0.321 g, 0.5 mmol) at room temperature to give a bright yellow reaction mixture. The mixture was stirred for 16 hours, forming a bright yellow precipitate. The volatiles were removed under vacuum to give a bright yellow powder, which was washed with hexane (10 mL) and benzene (10 mL). The crude material was dissolved in DCM (1.4 mL) at −78 °C, and layered with hexane (3 mL). Storage at –25 °C afforded **1-Pr** as yellow crystals (0.255 g, 40%). Anal. Calcd (%) for $C_{58}H_{58}BF_{20}Pr\cdot 2CH_2Cl_2$: C, 49.47; H, 4.29; Found: C, 47.68; H, 4.27. μ_{eff} (Evans method, [D$_2$]DCM, 298 K): 2.70 μ_B. The paramagnetism of **1-Pr** precluded assignment of its ^{1}H and ^{13}C{^{1}H} NMR spectra. ^{11}B{^{1}H} NMR ([D$_2$]DCM, 128 MHz, 298 K): δ = –19.42 (s). ^{19}F{^{1}H} NMR ([D$_2$]DCM, 376 MHz, 298 K): δ = −172.86 (s, *m-F*), −166.16 (s, *p-F*), −136.51 (s, *o-F*). FTIR (ATR, microcrystalline): $\tilde{\nu}$ = 2963 (m), 2909 (w), 2871 (w), 1643 (m), 1460 (s), 1365 (m), 1261 (s), 1242 (w), 1086 (s), 1021 (m), 977 (s), 798 (s), 774 (m), 755 (m), 683 (s), 660 (s), 609 (w), 573 (m), 473 (w), 441 (w) cm^{-1}.

[Nd(Cp^{ttt})$_2${(C$_6$F$_5$-κ^1-*F*)B(C$_6$F$_5$)$_3$}] (1-Nd). Benzene (15 mL) was added to a mixture of [H(SiEt$_3$)$_2$][B(C$_6$F$_5$)$_4$] (0.455 g, 0.5 mmol) and [Nd(Cp^{ttt})$_2$(Cl)] (0.323 g, 0.5 mmol) at room temperature to give a greenish blue reaction mixture. The mixture was stirred for 16 hours, forming a green precipitate. The volatiles were removed under vacuum to give a green powder, which was washed with hexane (10 mL) and benzene (10 mL). The crude material was dissolved in DCM (2 mL) at –78 °C, and layered with hexane (2 mL). Storage at –25 °C afforded **1-Nd** as green crystals (0.381 g, 59%). Anal. Calcd (%) for $C_{58}H_{58}BF_{20}Nd\cdot 2CH_2Cl_2$: C, 49.36; H, 4.28; Found: C, 49.51; H, 4.17. μ_{eff} (Evans method, [D$_2$]DCM, 298 K): 3.42 μ_B. ^{1}H NMR ([D$_2$]DCM, 400 MHz, 298 K): δ = –17.88 (br, 18H, $\nu_{1/2}$ ~ 310 Hz, Cp-C(C*H*$_3$)$_3$), –11.94 (br, 36H, $\nu_{1/2}$ ~ 700 Hz, Cp-C(C*H*$_3$)$_3$); no other signals observed. ^{11}B{^{1}H} NMR ([D$_2$]DCM, 128 MHz, 298 K): δ = –18.55 (s). The paramagnetism of **1-Nd** precluded assignment of its ^{13}C{^{1}H} NMR spectrum. ^{19}F{^{1}H} NMR ([D$_2$]DCM, 376 MHz, 298 K): δ = −170.31(s, *m-F*), −164.92 (s, *p-F*), −135.17 (s, *o-F*). FTIR (ATR, microcrystalline): $\tilde{\nu}$ = 2967 (br, m), 2875

(w), 2820 (w), 1643 (m), 1512 (s), 1460 (s), 1394 (w), 1365 (m), 1254 (w), 1241 (w), 1087 (s), 977 (s), 955 (w), 924 (w), 829 (m), 773 (s), 756 (s), 683 (s), 660 (s), 609 (w), 574 (m), 477 (w), 440 (w) cm^{-1}.

[Sm(Cp^{ttt})$_2$][B(C_6F_5)$_4$]·(C_7H_8)$_{1.5}$ (1-Sm·(C_7H_8)$_{1.5}$). Benzene (20 mL) was added to a mixture of [H($SiEt_3$)$_2$][B(C_6F_5)$_4$] (0.728 g, 0.8 mmol) and [Sm(Cp^{ttt})$_2$(Cl)] (0.522 g, 0.8 mmol) at room temperature to give a dark brown reaction mixture. The mixture was stirred for 16 hours, forming a dark brown precipitate. The volatiles were removed under vacuum to give a reddish brown powder, which was washed with hexane (15 mL) and benzene (15 mL). The crude material was dissolved in hot toluene (5 mL). Storage at room temperature afforded **1-Sm·(C_7H_8)$_{1.5}$** as red crystals (0.790 g, 69%). Anal. Calcd (%) for $C_{58}H_{58}BF_{20}Sm$: C, 53.74; H, 4.51; Found: C, 53.88; H, 4.49. μ_{eff} (Evans method, [D_2]DCM, 298 K): 1.90 μ_B. The paramagnetism of **1-Sm·(C_7H_8)$_{1.5}$** precluded assignment of its $^{13}C\{^1H\}$ NMR spectrum. ^1H NMR ([D_2]DCM, 400 MHz, 298 K): δ = −1.37 (s, 36H, Cp-C(C*H*$_3$)$_3$), 19.69 (s, 2H, Cp-C*H*); no other signals observed. $^{11}B\{^1H\}$ NMR ([D_2]DCM, 128 MHz, 298 K): δ = –16.76 (s). $^{19}F\{^1H\}$ NMR ([D_2]DCM, 376 MHz, 298 K): δ = −167.89 (s, *m-F*), −163.76 (t, J_{FF} = 20.44 Hz, *p-F*), −133.13 (s, *o-F*). ^1H NMR ([D_6]benzene, 400 MHz, 298 K): δ = −2.58 (s, 18H, Cp-C(C*H*$_3$)$_3$), −0.90 (s, 36H, Cp-C(C*H*$_3$)$_3$), 18.80 (s, 4H, Cp-C*H*). $^{11}B\{^1H\}$ NMR ([D_6]benzene, 128 MHz, 298 K): δ = –16.32 (s). $^{19}F\{^1H\}$ NMR ([D_6]benzene, 376 MHz, 298 K): δ = −168.96 (s, *m-F*), −162.02 (s, *p-F*), −131.93 (s, *o-F*). FTIR (ATR, microcrystalline): $\tilde{\upsilon}$ = 2964 (br, m), 2872 (w), 2796 (w), 1642 (m), 1512 (s), 1459 (s), 1367 (m), 1276 (m), 1239 (m), 1083 (s), 977 (s), 843 (w), 806 (w), 774 (m), 756 (m), 735 (m), 683 (m), 661 (s), 611 (w), 574 (w), 466 (w), 449 (w) cm^{-1}.

[La(Cp^{ttt})$_2$(Cl)] (2-La). THF (30 mL) was added to a pre-cooled (−78 °C) ampoule containing $LaCl_3$ (0.491 g, 2 mmol) and KCp^{ttt} (1.089 g, 4 mmol). The reaction mixture was allowed to reflux for 48 hours. The solvent was removed *in vacuo* and toluene (30 mL) was added. The reaction mixture was allowed to reflux for 48 hours. The resultant white suspension was allowed to settle for 3 hours and filtered. The pale yellow solution was concentrated to 2 mL and stored at 8 °C to afford **2-La** as colourless crystals (0.395 g, 31%). Anal. Calcd (%) for $C_{34}H_{58}LaCl$: C, 63.69; H, 9.12; Found: C, 63.57; H, 9.30. ^1H NMR ([D_6]benzene, 400 MHz, 298 K): δ = 1.25 (s, 18H, C(C*H*$_3$)$_3$), 1.52 (s, 36H, C(C*H*$_3$)$_3$), 6.51 (s, 4H, Cp-*H*). $^{13}C\{^1H\}$ NMR ([D_6]benzene, 100 MHz, 298 K): δ = 31.19 (C(*C*H$_3$)$_3$), 32.86 (*C*(CH$_3$)$_3$), 34.50 (*C*(CH$_3$)$_3$), 34.68 (C(*C*H$_3$)$_3$), 114.78 (Cp-*C*H), 138.17 (Cp-*C*), 139.58 (Cp-*C*). FTIR (ATR, microcrystalline): $\tilde{\upsilon}$ = 2956 (s), 2904 (w), 2869 (w), 1461 (m), 1389 (m),

1361 (s), 1260 (s), 1241 (m), 1091 (br, w), 1016 (s), 866 (w), 797 (s), 678 (s), 590 (w), 566 (w), 551 (w), 436 (w) cm^{-1}.

[Ce(Cp^{ttt})$_2$(Cl)] (2-Ce). THF (30 mL) was added to a pre-cooled (−78 °C) ampoule containing $CeCl_3$ (0.493 g, 2 mmol) and KCp^{ttt} (1.089 g, 4 mmol). The reaction mixture was allowed to reflux for 48 hours. The solvent was removed *in vacuo* and toluene (30 mL) was added. The reaction mixture was allowed to reflux for 48 hours. The resultant suspension was allowed to settle for 3 hours and filtered. The orange solution was concentrated to 2 mL and stored at 8 °C to afford **2-Ce** as orange crystals (0.651g, 51%). Anal. Calcd (%) for $C_{34}H_{58}CeCl$: C, 63.57; H, 9.10; Found: C, 63.60; H, 9.22. μ_{eff} (Evans method, [D$_6$]benzene, 298 K): 2.34 μ_B. The paramagnetism of **2-Ce** precluded assignment of its $^{13}C\{^1H\}$ NMR spectrum. ^1H NMR ([D$_6$]benzene, 400 MHz, 298 K): δ = −13.06 (s, 18H, C(CH_3)$_3$), −2.53 (s, 36H, C(CH_3)$_3$); no other signals observed. FTIR (ATR, microcrystalline): $\tilde{\upsilon}$ = 2954 (s), 2904 (m), 2868 (w), 1460 (s), 1389 (s), 1358 (s), 1241 (s), 1165 (m), 1001 (s), 958 (m), 816 (s), 774 (s), 678 (s), 566 (w), 436 (s) cm^{-1}.

[Pr(Cp^{ttt})$_2$(Cl)] (2-Pr). THF (30 mL) was added to a pre-cooled (−78 °C) ampoule containing $PrCl_3$ (0.495 g, 2 mmol) and KCp^{ttt} (1.089 g, 4 mmol). The reaction mixture was allowed to reflux for 48 hours. The solvent was removed *in vacuo* and toluene (30 mL) was added. The reaction mixture was allowed to reflux for 48 hours. The resultant suspension was allowed to settle for 3 hours and filtered. The pale green solution was concentrated to 2 mL and stored at 8 °C to afford **2-Pr** as light green crystals (0.672, 52%). Anal. Calcd (%) for $C_{34}H_{58}PrCl$: C, 63.49; H, 9.09; Found: C, 63.37; H, 9.22. μ_{eff} (Evans method, [D$_6$]benzene, 298 K): 3.35 μ_B. The paramagnetism of **2-Pr** precluded assignment of its $^{13}C\{^1H\}$ NMR spectrum. ^1H NMR ([D$_6$]benzene, 400 MHz, 298 K): δ = –36.08 (s, 18H, C(CH_3)$_3$), –7.74 (br, 36H, $v_{1/2}$ ~ 750 Hz, C(CH_3)$_3$); no other signals observed. FTIR (ATR, microcrystalline): $\tilde{\upsilon}$ = 2955 (s), 2905 (m), 2869 (w), 1460 (s), 1389 (s), 1359 (s), 1241 (s), 1166 (m), 1001 (s), 959 (m), 832 (m), 818 (s), 775 (s), 679 (s), 567 (m), 437 (s) cm^{-1}.

[Nd(Cp^{ttt})$_2$(Cl)] (2-Nd). THF (30 mL) was added to a pre-cooled (–78 °C) ampoule containing $NdCl_3$ (0.501 g, 2 mmol) and KCp^{ttt} (1.089 g, 4 mmol). The reaction mixture was allowed to reflux for 48 hours. The light blue solvent was removed *in vacuo* and toluene (30 mL) was added. The reaction mixture was allowed to reflux for 48 hours. The resultant suspension was allowed to settle for 3 hours and filtered. The green solution

was concentrated to 2 mL and stored at 8 °C to afford **2-Nd** as blue crystals (0.593 g, 46%). Anal. Calcd (%) for $C_{34}H_{58}NdCl$: C, 63.16; H, 9.04; Found: C, 61.22; H, 9.02. μ_{eff} (Evans method, [D$_6$]benzene, 298 K): 3.55 μ_B. The paramagnetism of **2-Nd** precluded assignment of its $^{13}C\{^1H\}$ NMR spectrum. ^1H NMR ([D$_6$]benzene, 400 MHz, 298 K): δ = –18.95 (s, 18H, C(CH_3)$_3$), –5.58 (s, 36H, C(CH_3)$_3$); no other signals observed. FTIR (ATR, microcrystalline): $\tilde{\upsilon}$ = 2955 (s), 2907 (m), 2870 (w), 1460 (s), 1389 (s), 1358 (s), 1241 (s), 1166 (m), 1001 (s), 833 (s), 820 (s), 775 (s), 679 (s), 439 (s) cm^{-1}.

[Sm(Cp^{ttt})$_2$(Cl)] (2-Sm). THF (30 mL) was added to a pre-cooled (–78 °C) ampoule containing $SmCl_3$ (0.513 g, 2 mmol) and KCp^{ttt} (1.089 g, 4 mmol). The reaction mixture was allowed to reflux for 24 hours. The solvent was removed *in vacuo* and toluene (30 mL) was added. The reaction mixture was allowed to reflux for 24 hours. The resultant suspension was allowed to settle for 3 hours and filtered. The orange solution was concentrated to 2.5 mL and stored at 8 °C to afford yellow crystals (0.656 g, 50%). Anal. Calcd (%) for $C_{34}H_{58}PrCl$: C, 62.57; H, 8.96; Found: C, 60.93; H, 9.19. μ_{eff} (Evans method, [D$_6$]benzene, 298 K): 1.69 μ_B. The paramagnetism of **2-Sm** precluded assignment of its $^{13}C\{^1H\}$ NMR spectrum. ^1H NMR ([D$_6$]benzene, 400 MHz, 298 K): δ = –6.01 (s, 18H, C(CH_3)$_3$), 0.55 (s, 36H, C(CH_3)$_3$), 19.80 (s, 4H, Cp-*H*). FTIR (ATR, microcrystalline): $\tilde{\upsilon}$ = 2956 (s), 2905 (m), 2870 (w), 1460 (s), 1389 (s), 1356 (s), 1241 (s), 1221 (w), 1166 (s), 1000 (s), 959 (m), 836 (w), 824 (s), 776 (s), 679 (s), 591 (w), 438 (s) cm^{-1}.

[{Eu(Cp^{ttt})(μ-PF$_6$-κ^4-*F*)(THF)}$_2$] (3) and (Cp^{ttt})$_2$ (4). THF (30 mL) was added to a pre-cooled (–78 °C) mixture of [Eu(I)$_2$(THF)$_2$] (2.200 g, 4 mmol) and KCp^{ttt} (2.178 g, 8 mmol) with stirring. The orange solution was warmed to room temperature and was stirred for 16 hours. The solvent was removed to afford an orange powder, which was extracted with hexane (100 mL). The orange solution was concentrated to 10 mL. Some orange crystals (2.012 g)* were formed at 8 °C, isolated and dried for 4 hours. Toluene (40 mL) was added in the pre-cooled (–78 °C) mixture of the orange crystals and [Fe(Cp)$_2$][PF$_6$] (1.059 g, 3.2 mmol). The orange mixture was warmed to room temperature, stirred for 16 hours and settled for 3 hours. The solvent was removed *in vacuo* to afford an orange powder. The orange powder was sublimed at 40 °C for 4 hours and toluene (7 mL) was added. Storage at 8 °C to afford the orange crystals of **3** (0.992 g, 51%). Filtration and further concentration of the solution to 2 mL afforded several colourless crystals of **4**. *The orange crystals were assumed to be [Eu(Cp^{ttt})$_2$] but the formation of **3** indicates that either [Eu(Cp^{ttt})$_2$(THF)] or [Eu(Cp^{ttt})(I)(THF)$_x$]$_n$

was present. Date for **3**: Anal. Calcd (%) for $C_{42}H_{74}O_2F_{12}P_2Eu_2$: C, 41.87; H, 6.19; Found: C, 40.42; H, 6.22. The paramagnetism of **3** precluded assignment of all NMR spectra. FTIR (ATR, microcrystalline): $\tilde{\upsilon}$ = 2960 (s), 2904 (w), 2867 (w), 1466 (m), 1389 (w), 1239 (s), 1202 (w), 1160 (w), 1095 (br, w), 1024 (s), 962 (w), 870 (s), 830 (s), 795 (s), 729 (s), 679 (s), 554 (s), 477 (w), 457 (w) cm^{-1}.

2. Crystallography

The crystal data for complexes **1-Ln** (Ln = La, **1-La**; Ce, **1-Ce**; Pr, **1-Pr**; Nd, **1-Nd**; Sm, **1-Sm·(C_7H_8)$_{1.5}$**), **2-Ln** (Ln = La, **2-La**; Ce, **2-Ce**; Pr, **2-Pr**; Nd, **2-Nd**; Sm, **2-Sm**), **3** and **4** are compiled in **Tables S1-S5**. Crystals of **1-La**, **1-Sm**, **2-La** and **2-Nd** were examined using an Oxford Diffraction Supernova diffractometer with a CCD area detector and a mirror-monochromated Mo Kα radiation (λ = 0.71073 Å). Crystals of **1-Ce**, **1-Pr**, **1-Nd**, **2-Sm**, **3** and **4** were examined using a Rigaku XtalLAB AFC11 diffractometer with a CCD area detector and a graphite-monochromated Mo Kα radiation (λ = 0.71073 Å). Crystals of **2-Ce** and **2-Pr** were examined using a Bruker Apex II diffractometer with a CCD area detector and a graphite-monochromated Cu Kα radiation (λ = 1.54178 Å). Intensities were integrated from data recorded on 0.5° (**1-Ce**, **1-Pr**, **2-Sm**, **4**), 0.9° (**1-Nd**, **2-La**) or 1° (**1-La**, **1-Sm•(C_7H_8)$_{1.5}$**, **2-Ce**, **2-Pr**, **2-Nd**, **3**) frames by ω rotation. Cell parameters were refined from the observed positions of all strong reflections in each data set. A Gaussian grid face-indexed (**1-La**, **1-Ce**, **1-Pr**, **1-Nd**, **1-Sm**, **2-La**, **2-Nd**, **2-Sm**), multi-scan (**2-Ce**, **2-Pr**, **4**) or analytical (**3**) absorption correction with a beam profile was applied.[1] The structures were solved using SHELXS;[2] the datasets were refined by full-matrix least-squares on all unique F^2 values,[3] with anisotropic displacement parameters for all non-hydrogen atoms, and with constrained riding hydrogen geometries; U_{iso}(H) was set at 1.2 (1.5 for methyl groups) times U_{eq} of the parent atom. The largest features in final difference syntheses were close to heavy atoms and were of no chemical significance. CrysAlisPro[1] was used for control and integration, and SHELX[2,3] was employed through OLEX2[4] for structure solution and refinement. ORTEP-3[5] and POV-Ray[6] were employed for molecular graphics. CCDC 1867384–1867395 contain the supplementary crystal data for this article. These data can be obtained free of charge from the Cambridge Crystallographic Data Centre via www.ccdc.cam.ac.uk/data_request/cif.

Table S1. Crystallographic data for **1-Ln**.

[a]Conventional $R = \Sigma||Fo| - |Fc||/\Sigma|Fo|$; $Rw = [\Sigma w(Fo^2 - Fc^2)^2/\Sigma w(Fo^2)^2]^{1/2}$; $S = [\Sigma w(Fo^2 - Fc^2)^2/\text{no. data} - \text{no. params})]^{1/2}$ for all data.

	**1-La**	**1-Ce**	**1-Pr**
Formula	$C_{58}H_{58}BF_{20}La$	$C_{58}H_{58}BF_{20}Ce$	$C_{58}H_{58}BF_{20}Pr$
Formula weight	1226.55	1285.97	1286.76
Crystal size, mm	0.484 × 0.355 × 0.164	0.418 × 0.153 × 0.134	0.134 × 0.108 × 0.091
Crystal system	Monoclinic	Monoclinic	Monoclinic
Space group	$P2_1/n$	$P2_1/n$	$P2_1/n$
a, Å	15.3148(8)	14.9311(4)	14.9336(5)
b, Å	16.7296(7)	16.5403(4)	16.4988(6)
c, Å	23.1479(10)	23.2432(7)	23.1845(8)
α, °	90	90	90
β, °	104.808(5)	105.543(3)	105.567(4)
γ, °	90	90	90
V, Å^3	5733.8(5)	5530.3(3)	5502.8(3)
Z	1	4	4
ρ_{calc}, g cm^3	1.421	1.545	1.553
μ, mm^{-1}	0.844	0.929	0.991
F(*000*)	2361	2596	2600
No. of reflections (unique)	19481 (10429)	37385 (10099)	40961 (11004)
S[a]	0.932	1.046	0.948
$R_1(wR_2)$ ($F^2 > 2\sigma(F^2)$)	0.0788 (0.2062)	0.0809 (0.2243)	0.0587 (0.1463)
R_{int}	0.044	0.079	0.069
Min./max. diff map, Å^{-3}	−1.90, 1.68	−1.12, 2.23	−0.77, 1.53

Table S2. Crystallographic data for **1-Ln**.

[a]Conventional $R = \Sigma||Fo| - |Fc||/\Sigma|Fo|$; $Rw = [\Sigma w(Fo^2 - Fc^2)^2/\Sigma w(Fo^2)^2]^{1/2}$; $S = [\Sigma w(Fo^2 - Fc^2)^2/\text{no. data} - \text{no. params})]^{1/2}$ for all data.

	**1-Nd**	**1-Sm·$(C_7H_8)_{1.5}$**
Formula	$C_{58}H_{58}BF_{20}Nd$	$C_{68.5}H_{70}BF_{20}Sm$
Formula weight	1290.09	1434.4
Crystal size, mm	0.114 × 0.076 × 0.053	0.268 × 0.124 × 0.063
Crystal system	Monoclinic	triclinic
Space group	$P2_1/n$	P-1
a, Å	14.9319(7)	11.9059(5)
b, Å	16.4823(8)	12.4295(5)
c, Å	23.1975(12)	21.9307(8)
α, °	90	93.574(3)
β, °	105.408(5)	91.290(3)
γ, °	90	99.963(3)
V, Å^3	5504.0(5)	3188.4(2)
Z	4	1
ρ_{calc}, g cm^3	1.557	1.494
μ, mm^{-1}	1.049	1.020
F(*000*)	2604	1456
No. of reflections (unique)	27882 (9983)	17090 (11615)
S[a]	1.043	1.068
$R_1(wR_2)$ ($F^2 > 2\sigma(F^2)$)	0.0637 (0.1459)	0.0396 (0.0867)
R_{int}	0.070	0.027
Min./max. diff map, Å^{-3}	−0.71, 1.29	−0.73, 0.95

Table S3. Crystallographic data for **2-Ln**.

[a]Conventional $R = \Sigma||Fo| - |Fc||/\Sigma|Fo|$; $Rw = [\Sigma w(Fo^2 - Fc^2)^2/\Sigma w(Fo^2)^2]^{1/2}$; $S = [\Sigma w(Fo^2 - Fc^2)^2/\text{no. data} - \text{no. params})]^{1/2}$ for all data.

	**2-La**	**2-Ce**	**2-Pr**
Formula	$C_{34}H_{58}ClLa$	$C_{34}H_{58}ClCe$	$C_{34}H_{58}ClPr$
Formula weight	641.16	642.37	643.16
Crystal size, mm	0.189 × 0.084 × 0.045	0.1 × 0.1 × 0.1	0.2 × 0.1 × 0.1
Crystal system	Monoclinic	Monoclinic	Monoclinic
Space group	$P2_1/c$	$P2_1/c$	$P2_1/c$
a, Å	10.3202(7)	10.32247(10)	10.3273(2)
b, Å	15.4359(10)	15.4139(2)	15.4158(2)
c, Å	20.675(2)	20.6193(2)	20.5592(2)
α, °	90	90	90
β, °	98.635(7)	98.7771(8)	98.8665(12)
γ, °	90	90	90
V, Å^3	3256.2(4)	3242.30(6)	3234.00(7)
Z	4	4	4
ρ_{calc}, g cm^3	1.308	1.316	1.321
μ, mm^{-1}	1.413	11.724	12.435
F(*000*)	1344	1348	1352
No. of reflections (unique)	11028 (5939)	23050 (5867)	21073 (5871)
S[a]	1.028	1.057	1.026
$R_1(wR_2)$ ($F^2 > 2\sigma(F^2)$)	0.0760 (0.1154)	0.0402 (0.1103)	0.0343 (0.0899)
R_{int}	0.094	0.034	0.043
Min./max. diff map, Å^{-3}	−0.94, 0.74	−1.23, 1.75	−0.86, 1.27

Table S4. Crystallographic data for **2-Ln**.

[a]Conventional $R = \Sigma||Fo| - |Fc||/\Sigma|Fo|$; $Rw = [\Sigma w(Fo^2 - Fc^2)^2/\Sigma w(Fo^2)^2]^{1/2}$; $S = [\Sigma w(Fo^2 - Fc^2)^2/\text{no. data} - \text{no. params})]^{1/2}$ for all data.

	**2-Nd**	**2-Sm**
Formula	$C_{34}H_{58}ClNd$	$C_{34}H_{58}ClSm$
Formula weight	646.49	652.60
Crystal size, mm	0.124 × 0.064 × 0.053	0.216 × 0.119 × 0.095
Crystal system	Monoclinic	Monoclinic
Space group	$P2_1/c$	$P2_1/c$
a, Å	10.3426(4)	10.33268(13)
b, Å	15.4145(9)	15.3878(2)
c, Å	20.4955(9)	20.4435(3)
α, °	90	90
β, °	98.962(4)	99.1012(12)
γ, °	90	90
V, Å^3	3227.6(3)	3209.54(7)
Z	4	4
ρ_{calc}, g cm^3	1.330	1.351
μ, mm^{-1}	1.711	14.623
F(*000*)	1356	1364
No. of reflections (unique)	22469 (5912)	25394 (5859)
S[a]	0.935	1.041
$R_1(wR_2)$ ($F^2 > 2\sigma(F^2)$)	0.0571 (0.0969)	0.0394 (0.1090)
R_{int}	0.103	0.038
Min./max. diff map, Å^{-3}	−0.68, 1.45	−0.95, 2.42

Table S5. Crystallographic data for **3** and **4**.

[a]Conventional $R = \Sigma||Fo| - |Fc||/\Sigma|Fo|$; $Rw = [\Sigma w(Fo^2 - Fc^2)^2/\Sigma w(Fo^2)^2]^{1/2}$; $S = [\Sigma w(Fo^2 - Fc^2)^2/\text{no. data} - \text{no. params})]^{1/2}$ for all data.

	**3**	**4**
Formula	$C_{56}H_{90}Eu_2F_{12}O_2P_2$	$C_{34}H_{58}$
Formula weight	1389.13	466.80
Crystal size, mm	0.098 × 0.062 × 0.059	0.163 × 0.103 × 0.062
Crystal system	triclinic	monoclinic
Space group	*P*-1	$P2_1/c$
a, Å	9.0959(8)	11.1064(18)
b, Å	9.3505(7)	15.247(2)
c, Å	17.8572(13)	18.213(4)
α, °	87.572(6)	90
β, °	88.959(6)	95.254(16)
γ, °	89.725(7)	90
V, Å^3	1517.2(2)	3071.4(9)
Z	1	4
ρ_{calc}, g cm^3	1.520	1.010
μ, mm^{-1}	2.175	0.056
F(*000*)	706	1048
No. of reflections (unique)	9542	9919 (5571)
S[a]	1.009	0.902
$R_1(wR_2)$ ($F^2 > 2\sigma(F^2)$)	0.0472 (0.1206)	
R_{int}		
Min./max. diff map, Å^{-3}	−1.51, 1.55	

3. Molecular structures of complexes 1-La, 1-Ce, 1-Pr, 1-Nd and 1-Sm·$(C_7H_8)_{1.5}$

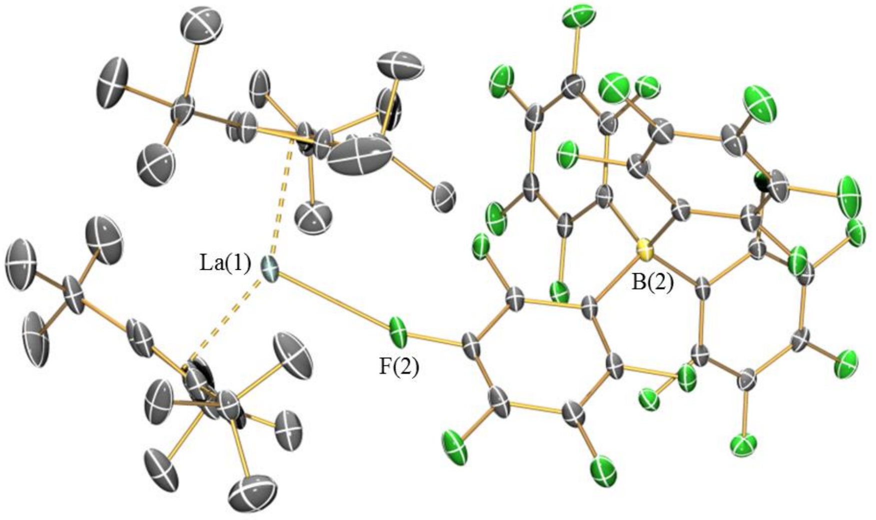

Figure S1. Molecular structure of [La$(Cp^{ttt})_2${$(C_6F_5$-κ^1-*F*)B$(C_6F_5)_3$}] (**1-La**) with selected labelling. Displacement ellipsoids set at 30 % probability level and hydrogen atoms are omitted for clarity. Selected distances and angles: La1···$Cp_{centroid1}$, 2.50646(11) Å; La1···$Cp_{centroid2}$, 2.49855(9) Å; La1···F2, 2.67944(9) Å; $Cp_{centroid1}$···La1···$Cp_{centroid2}$, 148.1303(15)°.

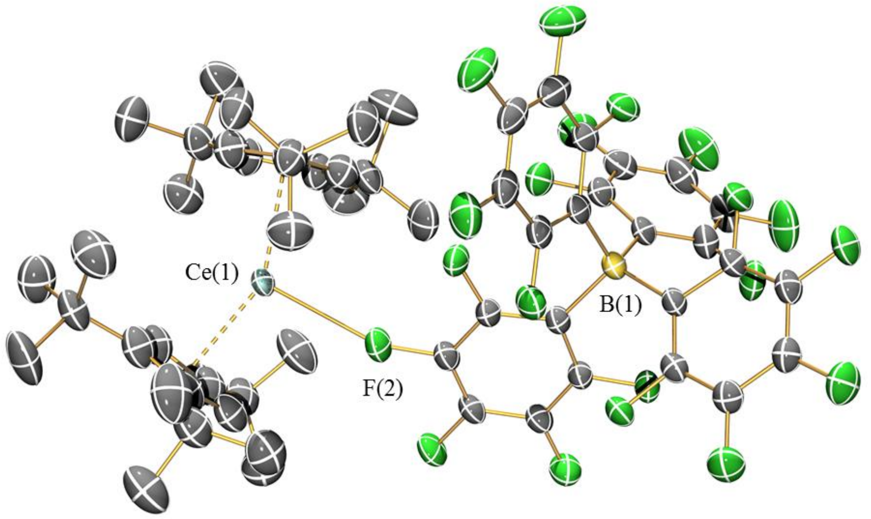

Figure S2. Molecular structure of [Ce$(Cp^{ttt})_2${$(C_6F_5$-κ^1-*F*)B$(C_6F_5)_3$}] (**1-Ce**) with selected labelling. Displacement ellipsoids set at 30 % probability level and hydrogen atoms are omitted for clarity. Selected distances and angles: Ce1···$Cp_{centroid1}$, 2.507(4) Å; Ce1···$Cp_{centroid2}$, 2.478(5) Å; Ce1···F2, 2.677(5) Å; $Cp_{centroid1}$···Ce1···$Cp_{centroid2}$, 149.10(15)°.

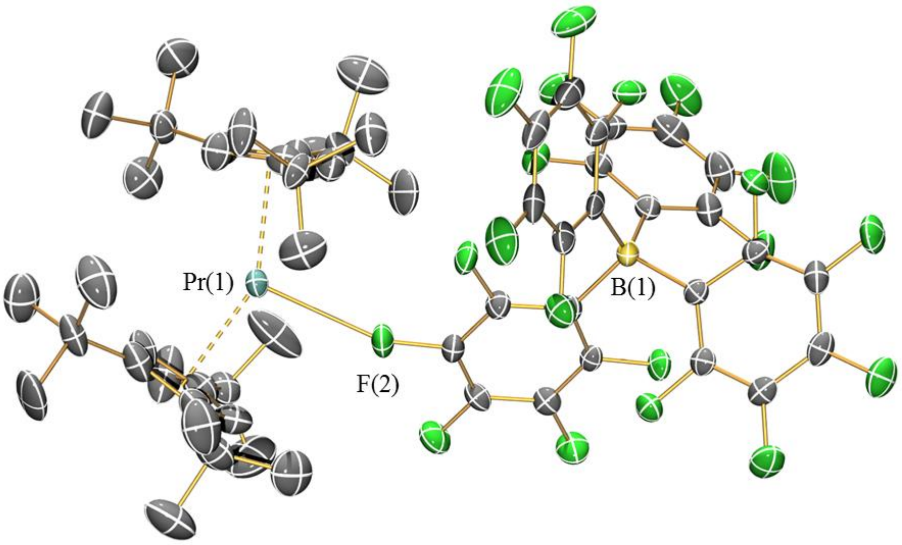

Figure S3. Molecular structure of [Pr(Cp^{ttt})$_2${(C$_6$F$_5$-κ^1-*F*)B(C$_6$F$_5$)$_3$}] (**1-Pr**) with selected labelling. Displacement ellipsoids set at 30 % probability level and hydrogen atoms are omitted for clarity. Selected distances and angles: Pr1···Cp$_{centroid1}$, 2.479(3) Å; Pr1···Cp$_{centroid2}$, 2.459(4) Å; Pr1···F2, 2.664(3) Å; Cp$_{centroid1}$···Pr1···Cp$_{centroid2}$, 149.48(11)°.

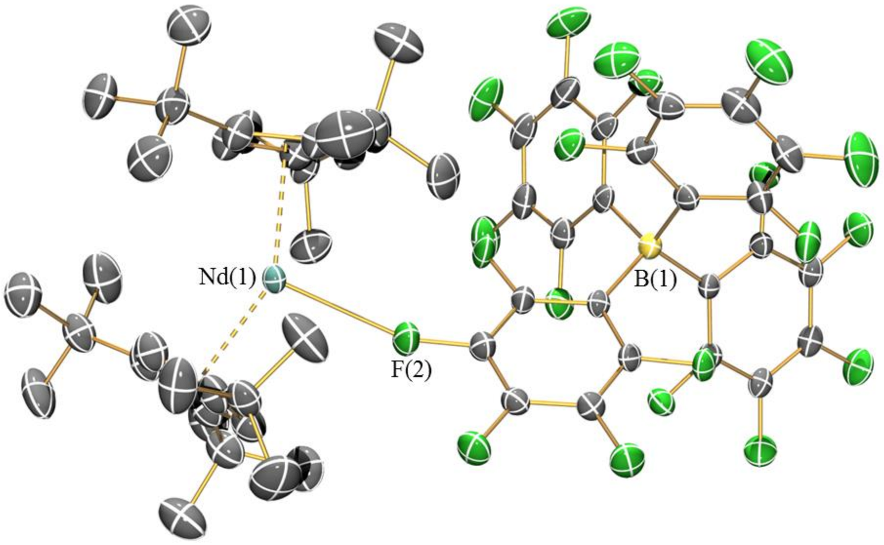

Figure S4. Molecular structure of [Nd(Cp^{ttt})$_2${(C$_6$F$_5$-κ^1-*F*)B(C$_6$F$_5$)$_3$}] (**1-Nd**) with selected labelling. Displacement ellipsoids set at 30 % probability level and hydrogen atoms are omitted for clarity. Selected distances and angles: Nd1···Cp$_{centroid1}$, 2.445(5) Å; Nd1···Cp$_{centroid2}$, 2.464(4) Å; Nd1···F2, 2.632(4) Å; Cp$_{centroid1}$···Nd1···Cp$_{centroid2}$, 148.10(13)°.

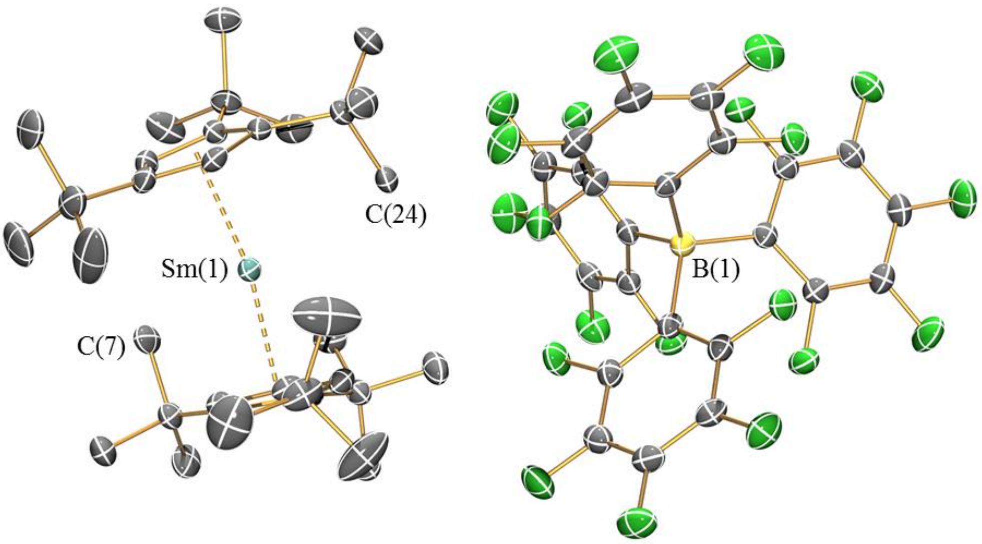

Figure S5. Molecular structure of $[Sm(Cp^{ttt})_2][B(C_6F_5)_4]\cdot(C_7H_8)_{1.5}$ (**1-Sm·$(C_7H_8)_{1.5}$**) with selected labelling. Displacement ellipsoids set at 30 % probability level, lattice solvent and hydrogen atoms are omitted for clarity. Selected distances and angles: Sm1···$Cp_{centroid1}$, 2.3843(17) Å; Sm1···$Cp_{centroid2}$, 2.3923(17) Å; Sm1···C7, 2.966(4) Å; Sm1···C24, 2.998(4) Å; Sm1···H, 2.4623(3) and 2.4258(3) Å; $Cp_{centroid1}$···Sm1···$Cp_{centroid2}$, 153.23(6)°; H···Sm1···H, 171.06°.

4. Molecular structures of complexes 2-La, 2-Ce, 2-Pr, 2-Nd and 2-Sm

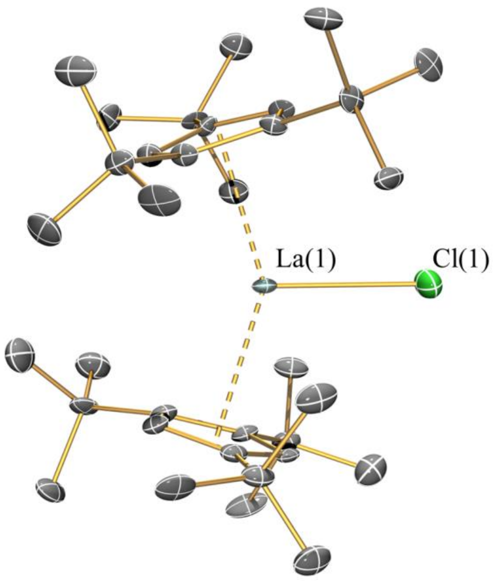

Figure S6. Molecular structure of [La(Cp^{ttt})$_2$(Cl)] (**2-La**) with selected labelling. Displacement ellipsoids set at 30 % probability level and hydrogen atoms are omitted for clarity. Selected distances and angles: La1···Cp$_{centroid1}$, 2.571(4) Å; La1···Cp$_{centroid2}$, 2.576(4) Å; La1···Cl1, 2.687(2) Å; Cp$_{centroid1}$···La1···Cp$_{centroid2}$, 146.13(13)°.

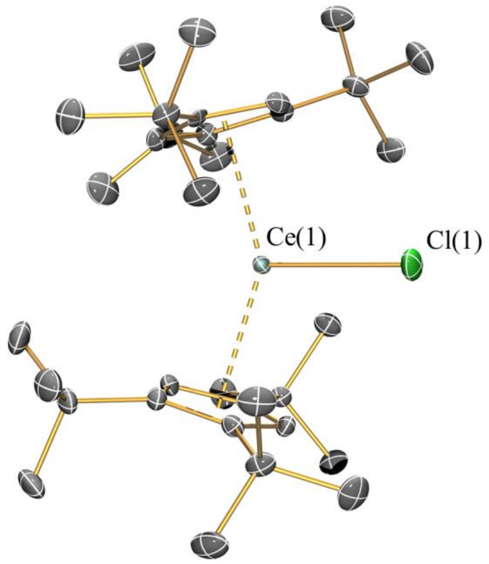

Figure S7. Molecular structure of [Ce(Cp^{ttt})$_2$(Cl)] (**2-Ce**) with selected labelling. Displacement ellipsoids set at 30 % probability level and hydrogen atoms are omitted for clarity. Selected distances and angles: Ce1···Cp$_{centroid1}$, 2.546(2) Å; Ce1···Cp$_{centroid2}$, 2.544(2) Å; Ce(1)···Cl1, 2.6606(10) Å; Cp$_{centroid1}$···Ce1···Cp$_{centroid2}$, 146.46(5)°.

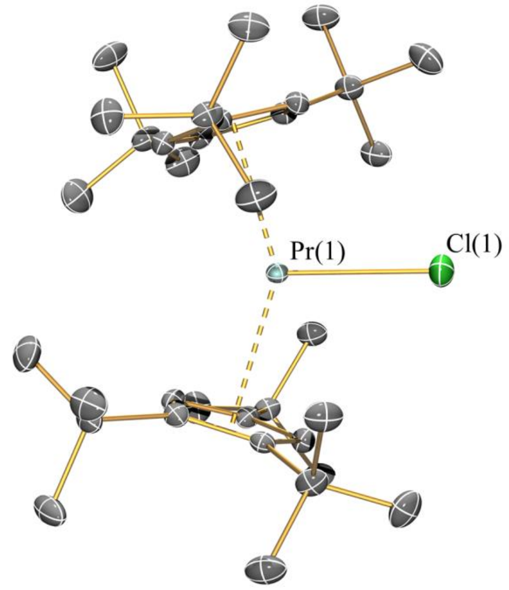

Figure S8. Molecular structure of [Pr(Cp^{ttt})$_2$(Cl)] (**2-Pr**) with selected labelling. Displacement ellipsoids set at 30 % probability level and hydrogen atoms are omitted for clarity. Selected distances and angles: Pr1···Cp$_{centroid1}$, 2.522(2) Å; Pr1···Cp$_{centroid2}$, 2.520(2) Å; Pr1···Cl1, 2.6456(10) Å; Cp$_{centroid1}$···Pr1···Cp$_{centroid2}$, 146.66(5)°.

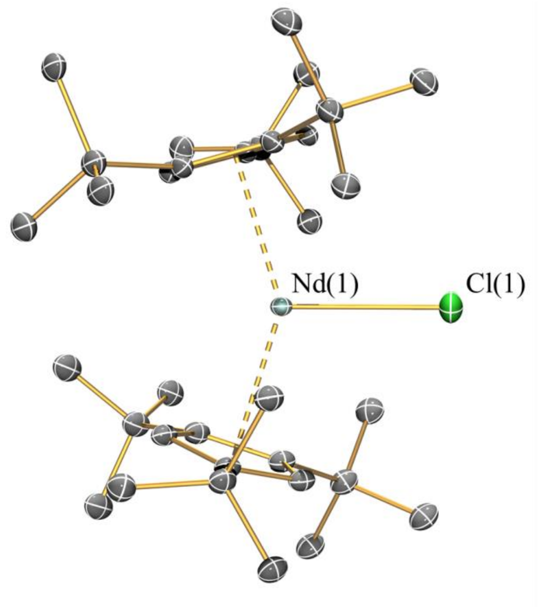

Figure S9. Molecular structure of [Nd(Cp^{ttt})$_2$(Cl)] (**2-Nd**) with selected labelling. Displacement ellipsoids set at 30 % probability level and hydrogen atoms are omitted for clarity. Selected distances and angles: Nd1···Cp$_{centroid1}$, 2.503(3) Å; Nd1···Cp$_{centroid2}$, 2.498(3) Å; Nd1···Cl1, 2.633(2) Å; Cp$_{centroid1}$···Nd1···Cp$_{centroid2}$, 146.07(11)°.

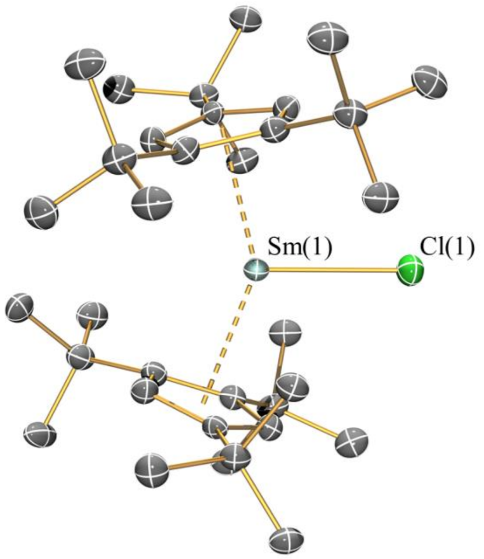

Figure S10. Molecular structure of [Sm(Cp^{ttt})$_2$(Cl)] (**2-Sm**) with selected labelling. Displacement ellipsoids set at 30 % probability level and hydrogen atoms are omitted for clarity. Selected distances and angles: Sm1···$Cp_{centroid1}$, 2.477(2) Å; Sm1···$Cp_{centroid2}$, 2.474(2) Å; Sm1···Cl1, 2.6027(10) Å; $Cp_{centroid1}$···Sm1···$Cp_{centroid2}$, 145.95(5)°.

5. Molecular structures of complexes 3 and 4

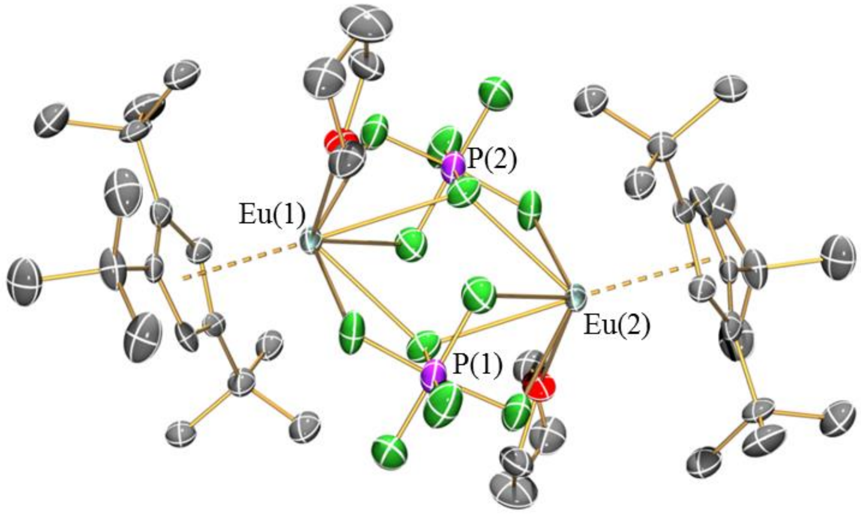

Figure S11. Molecular structure of [{Eu(Cp^{ttt})(μ-PF$_6$-κ^4-F)(THF)}$_2$] (**3**) with selected labelling. Displacement ellipsoids set at 30 % probability level and hydrogen atoms are omitted for clarity.

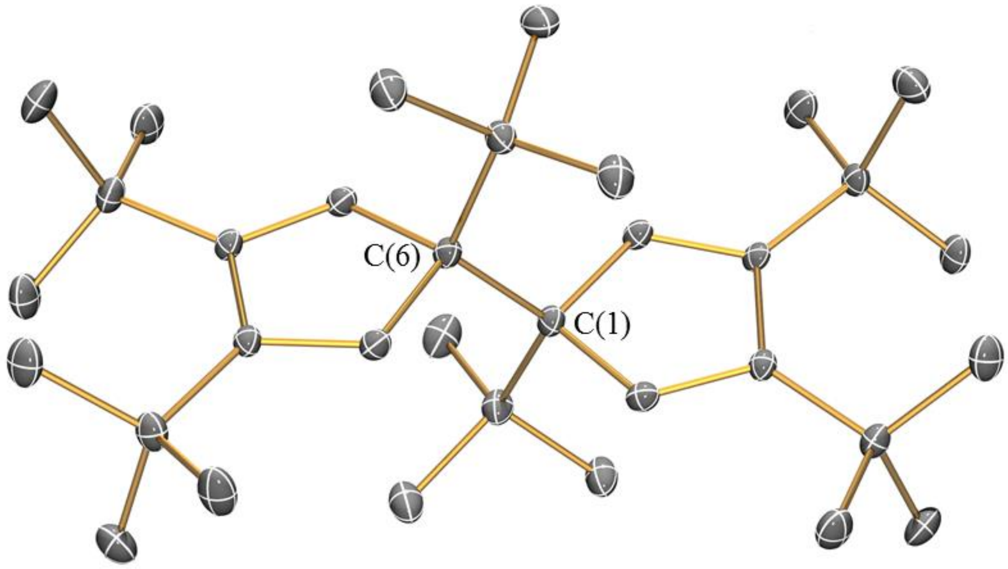

Figure S12. Molecular structure of (Cp^{ttt})$_2$ (**4**) with selected labelling. Displacement ellipsoids set at 30 % probability level and hydrogen atoms are omitted for clarity.

6. NMR spectroscopy

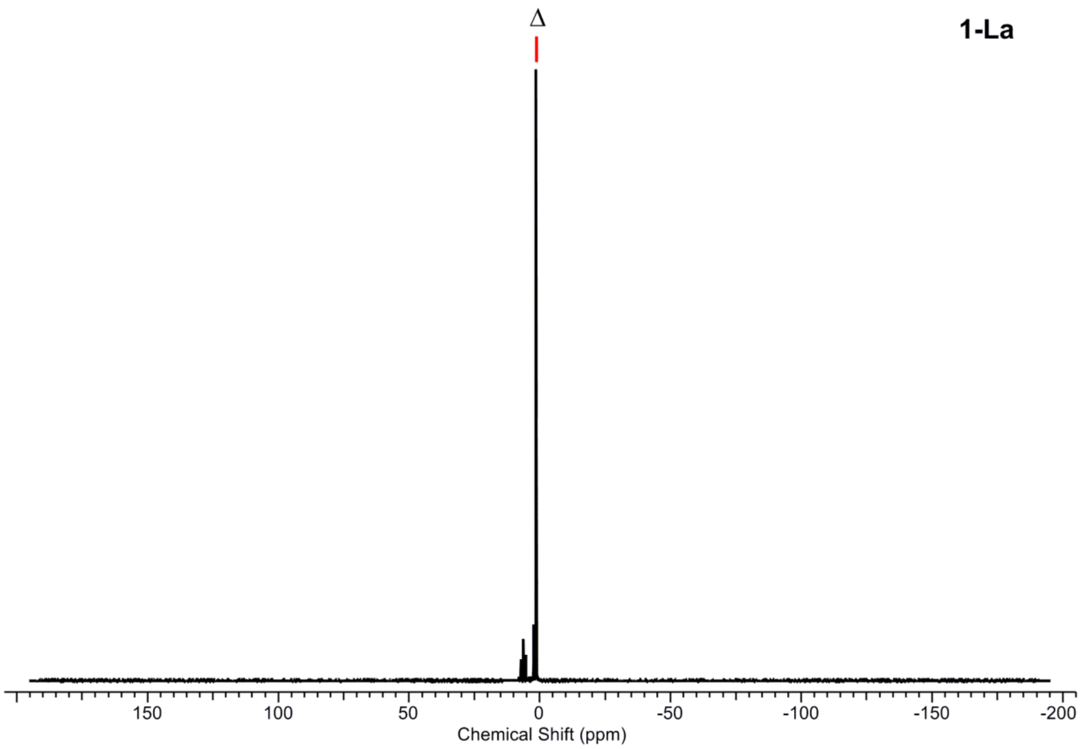

Figure S13. ^{1}H NMR spectrum of complex **1-La** in [D_2]DCM. Δ denotes solvent residual.

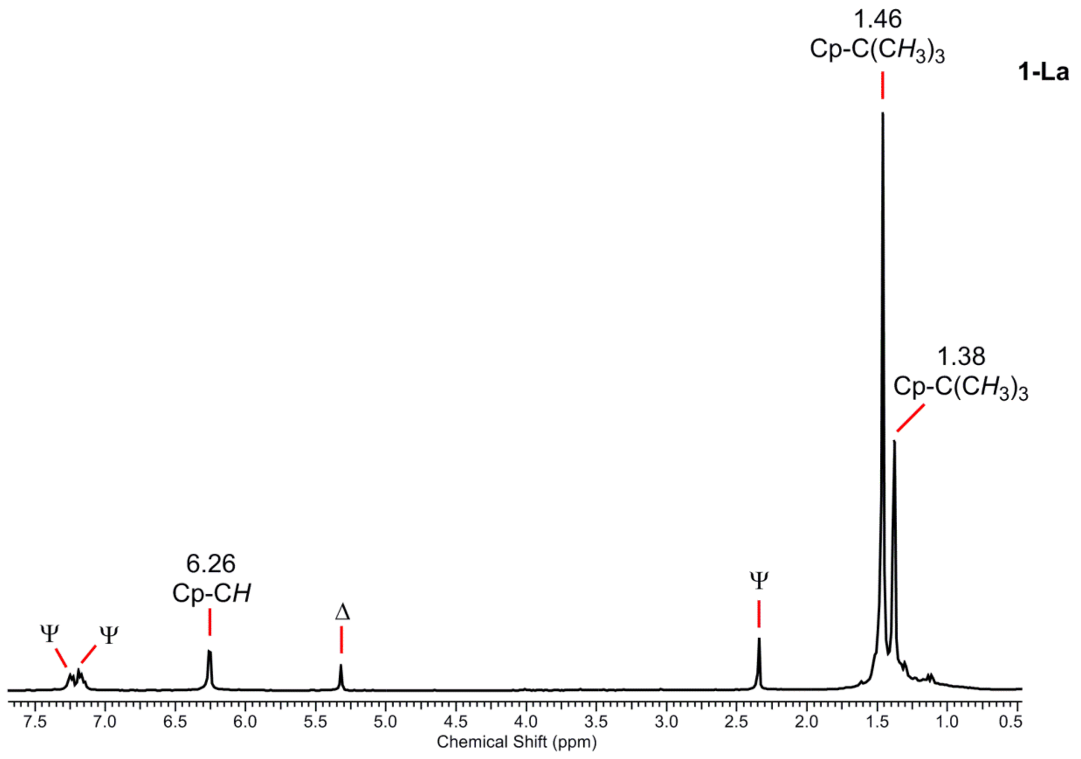

Figure S14. ^{1}H NMR spectrum of complex **1-La** in [D_2]DCM zoomed in the region 0.5 and 7.5 ppm. Δ denotes solvent residual, Ψ denotes toluene of recrystallization.

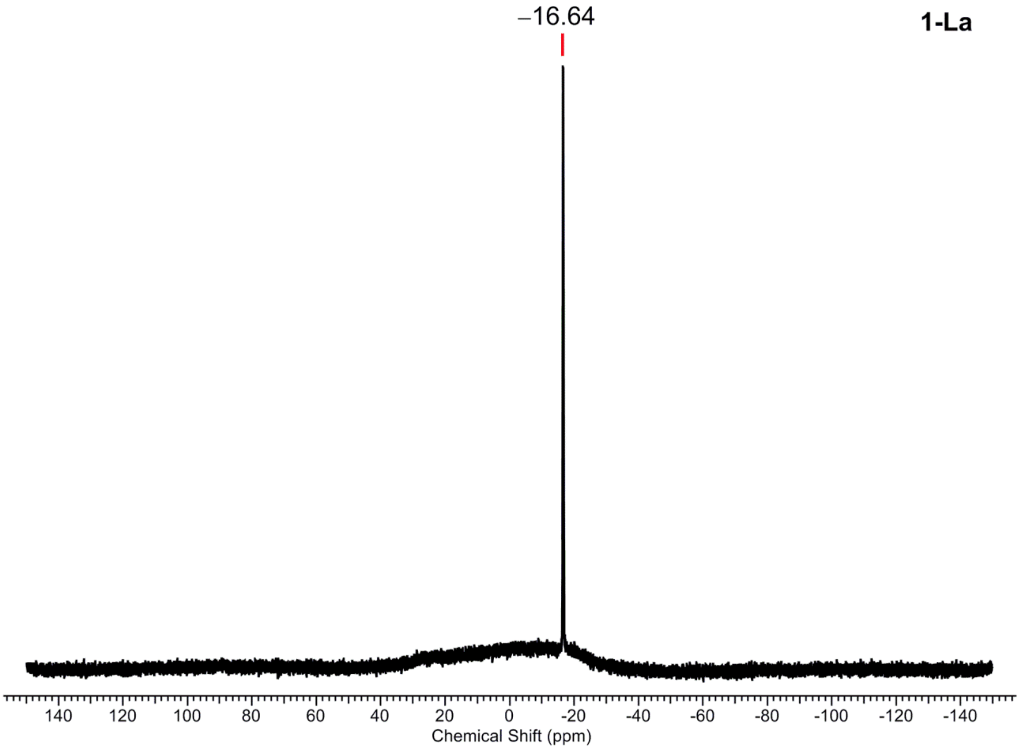

Figure S15. ^{11}B NMR spectrum of complex **1-La** in [D$_2$]DCM.

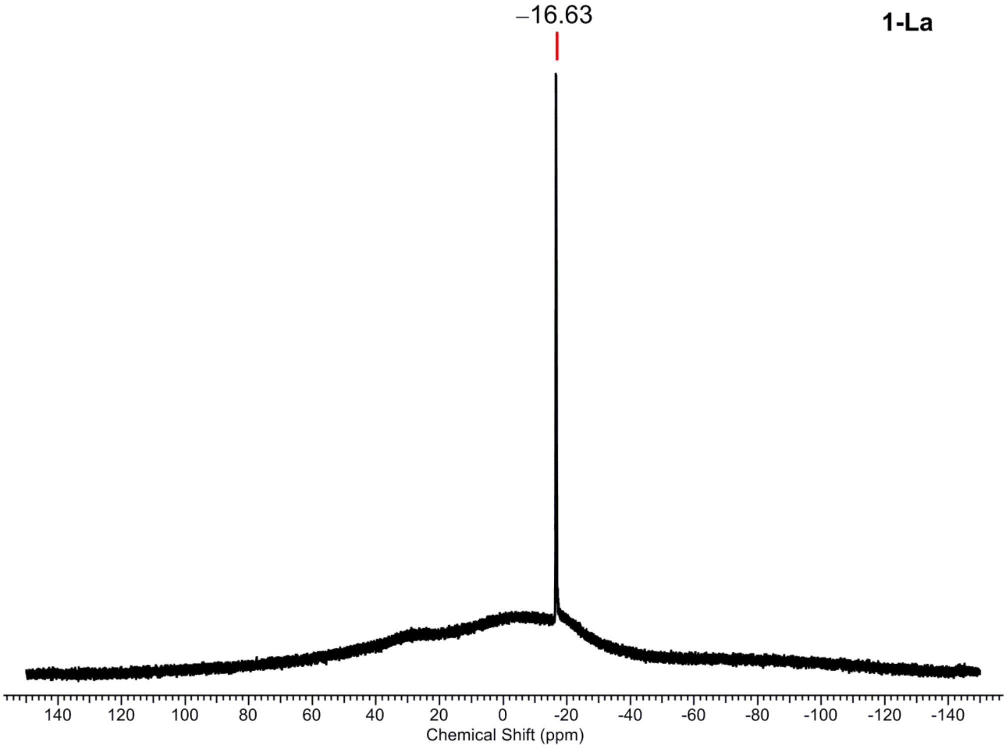

Figure S16. $^{11}B\{^1H\}$ NMR spectrum of complex **1-La** in [D$_2$]DCM.

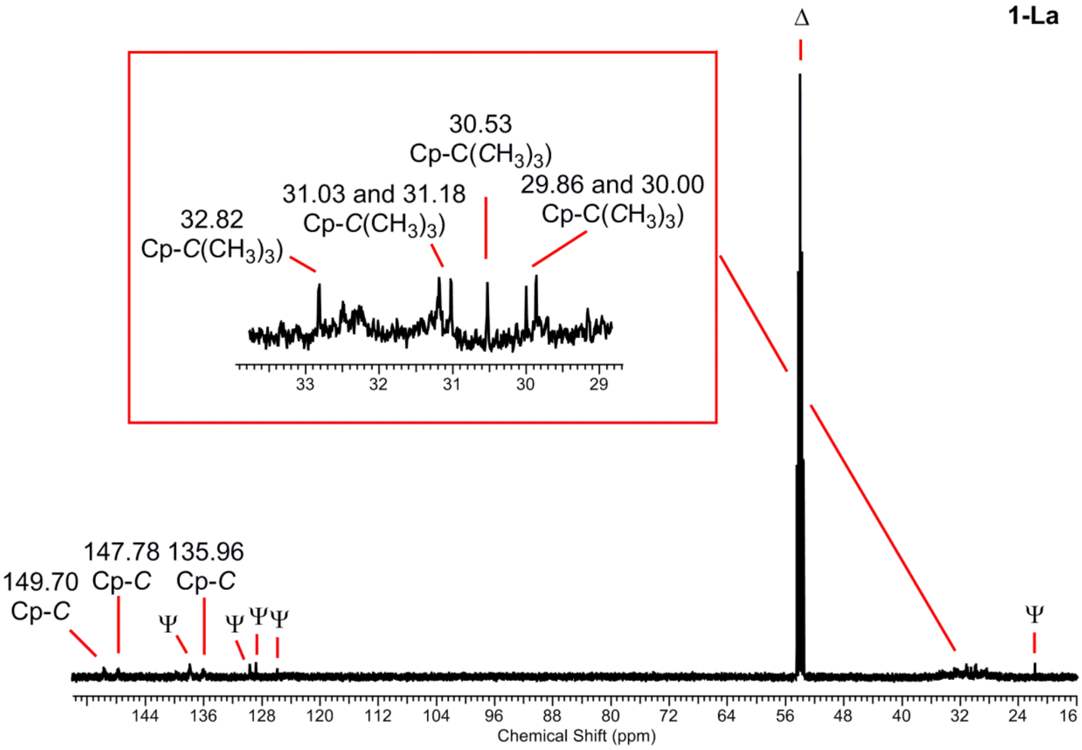

Figure S17. $^{13}C\{^{1}H\}$ NMR spectrum of complex **1-La** in [D_2]DCM. Δ denotes solvent residual, Ψ denotes toluene of recrystallization.

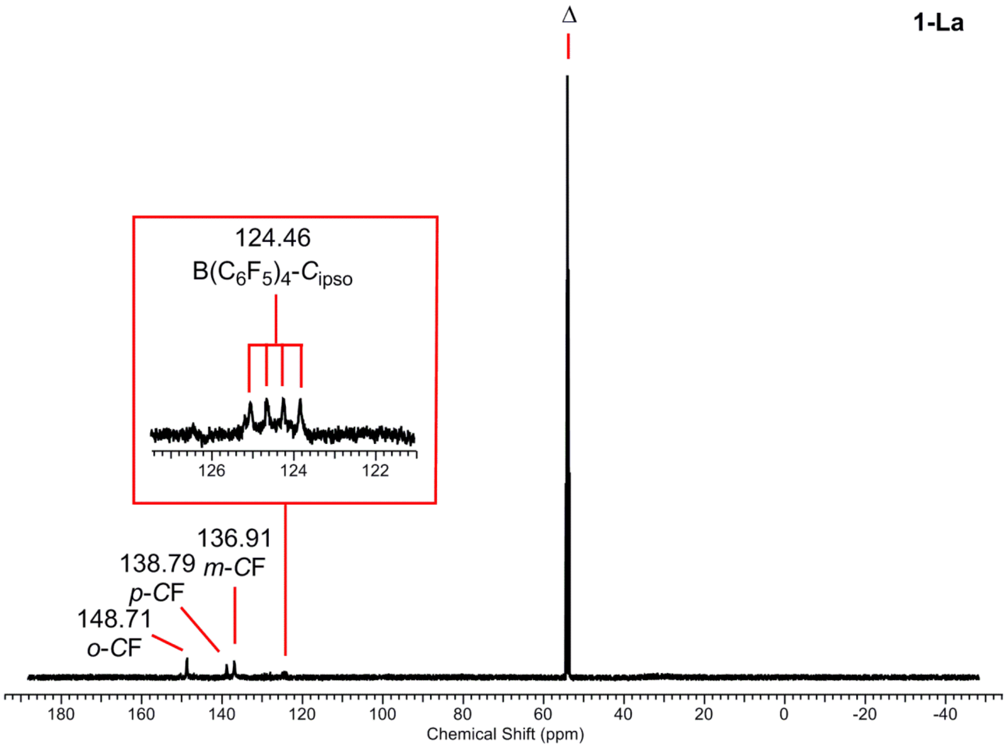

Figure S18. $^{13}C\{^{19}F\}$ NMR spectrum of complex **1-La** in [D_2]DCM.

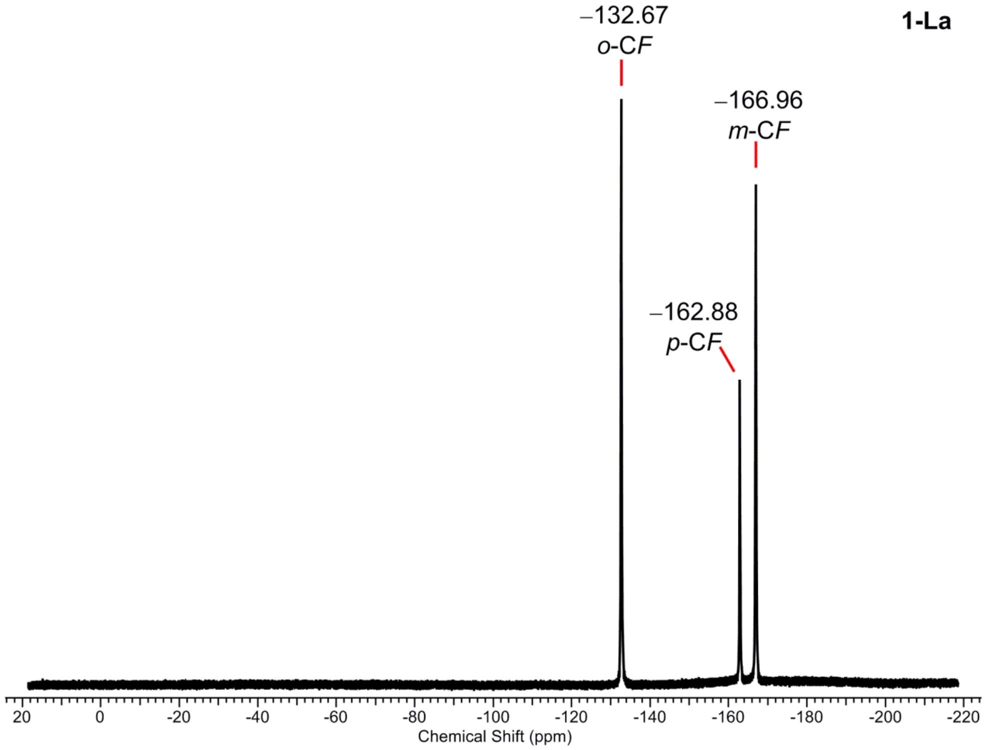

Figure S19. ^{19}F NMR spectrum of complex **1-La** in [D_2]DCM.

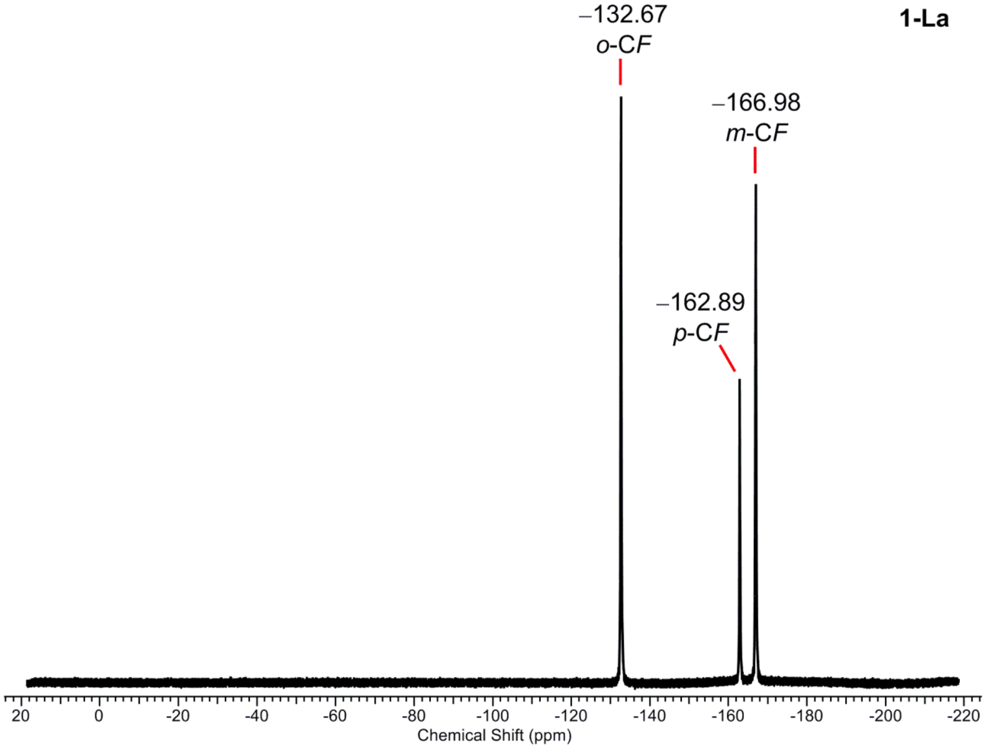

Figure S20. $^{19}F\{^1H\}$ NMR spectrum of complex **1-La** in [D_2]DCM.

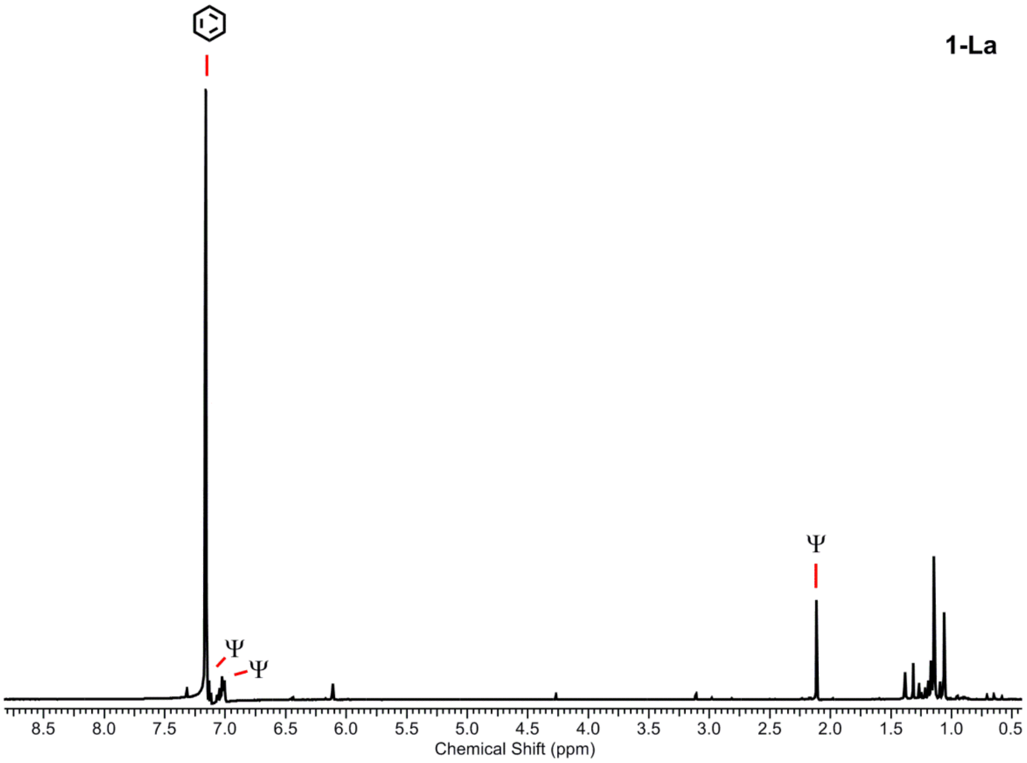

Figure S21. ^{1}H NMR spectrum of complex **1-La** in [D_6]benzene. Solvent residual marked. Ψ denotes toluene of recrystallization.

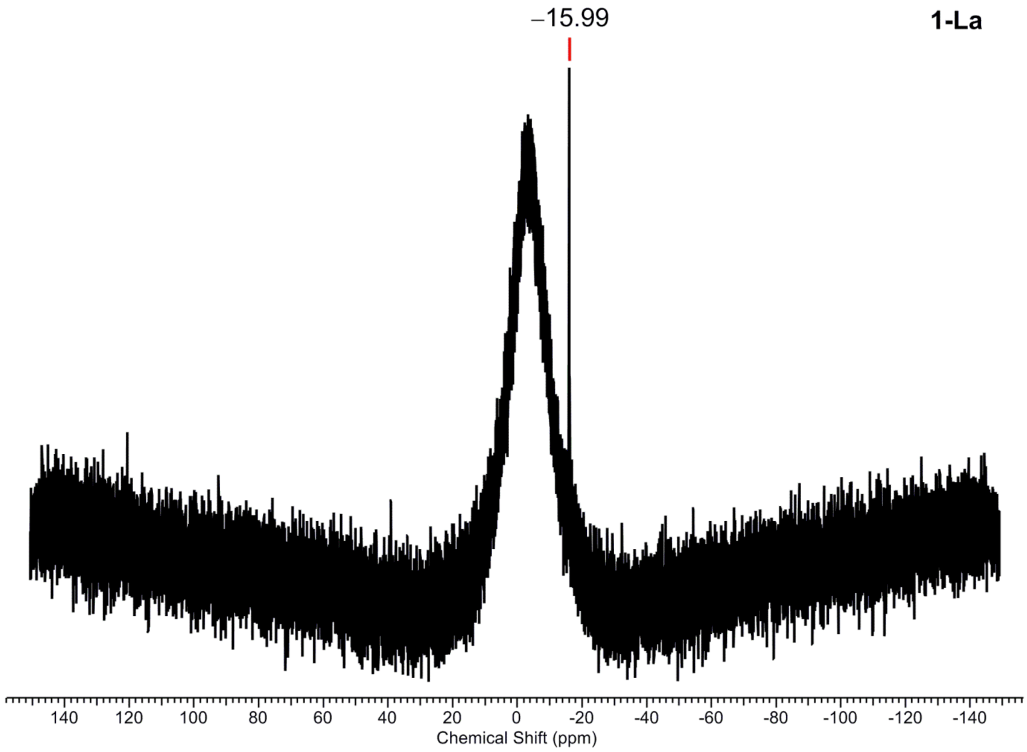

Figure S22. ^{11}B NMR spectrum of complex **1-La** in [D_6]benzene.

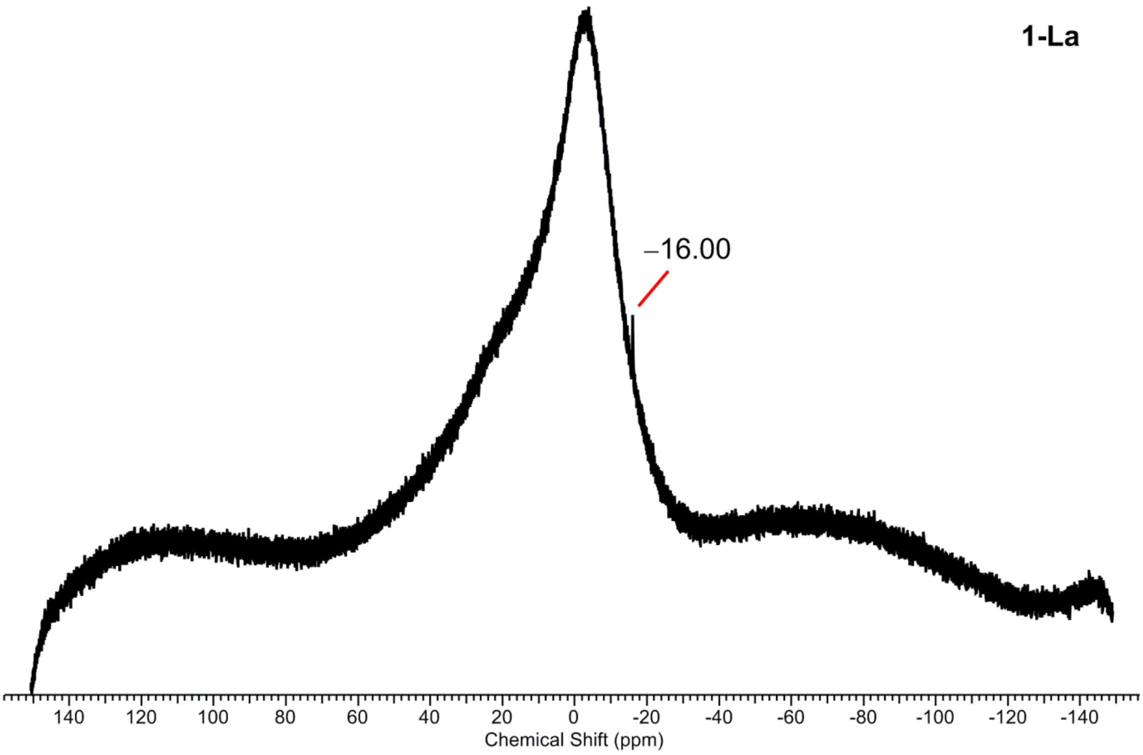

Figure S23. $^{11}B\{^1H\}$ NMR spectrum of complex **1-La** in [D$_6$]benzene.

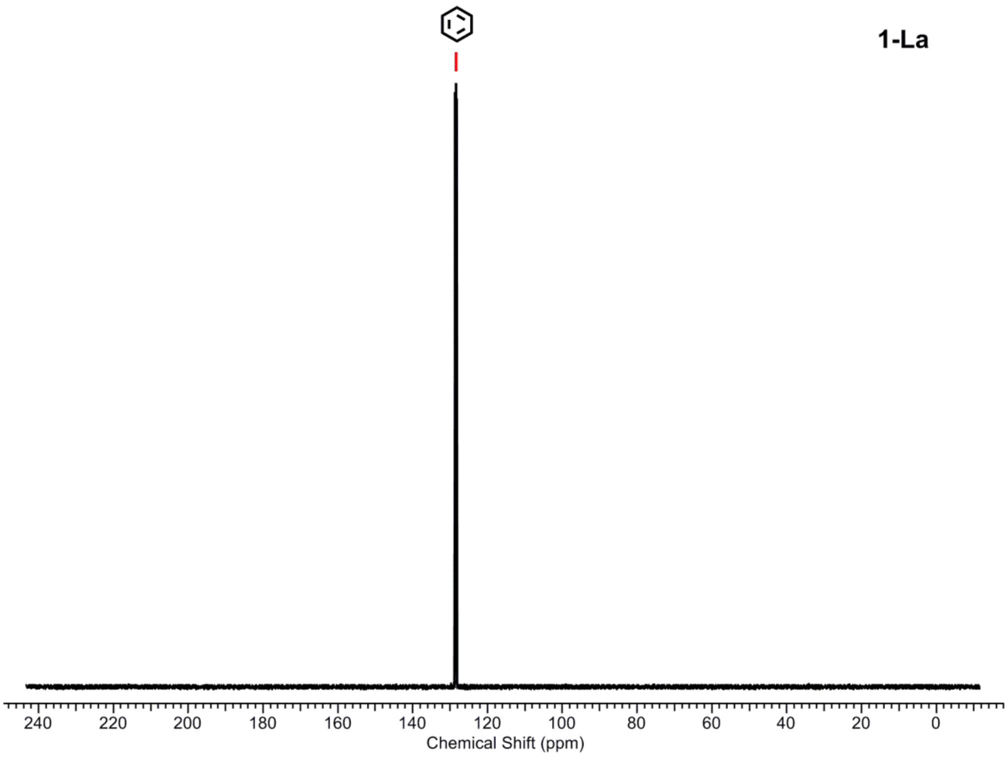

Figure S24. $^{13}C\{^1H\}$ NMR spectrum of complex **1-La** in [D$_6$]benzene. Solvent residual marked.

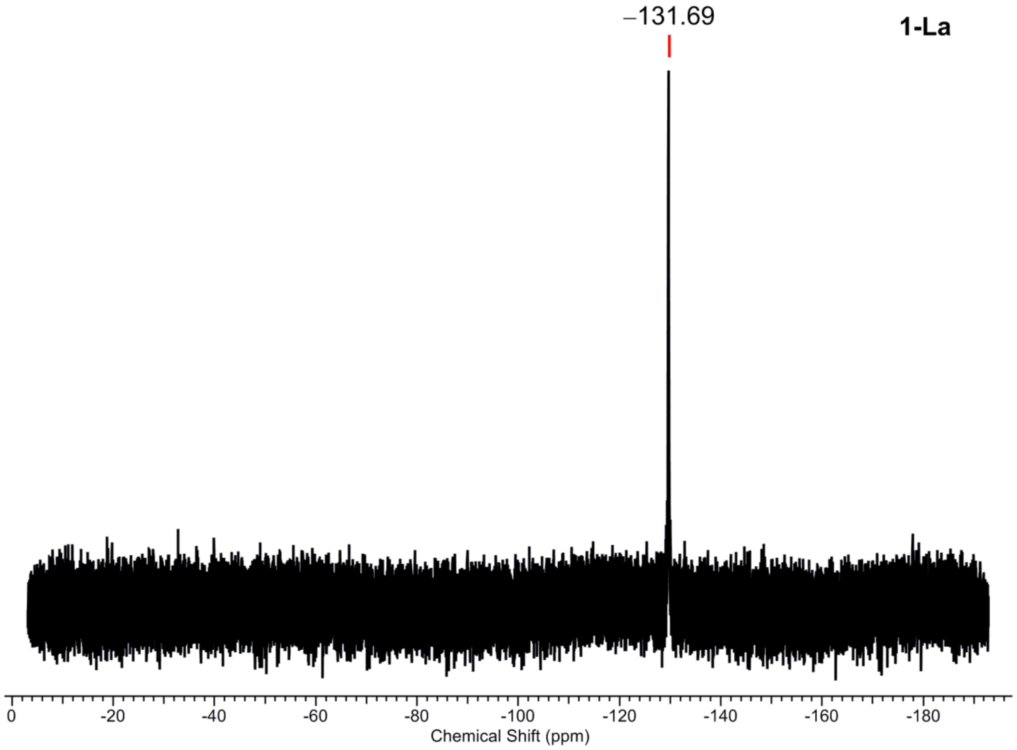

Figure S25. ^{19}F NMR spectrum of complex **1-La** in [D$_6$]benzene.

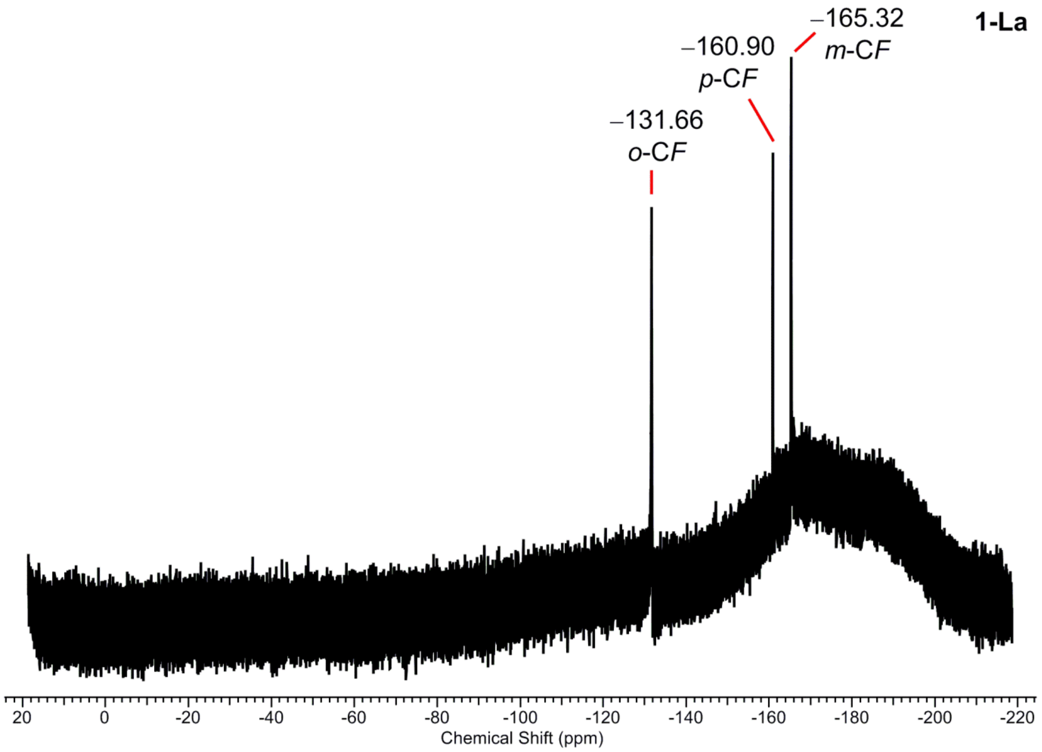

Figure S26. $^{19}F\{^1H\}$ NMR spectrum of complex **1-La** in [D$_6$]benzene.

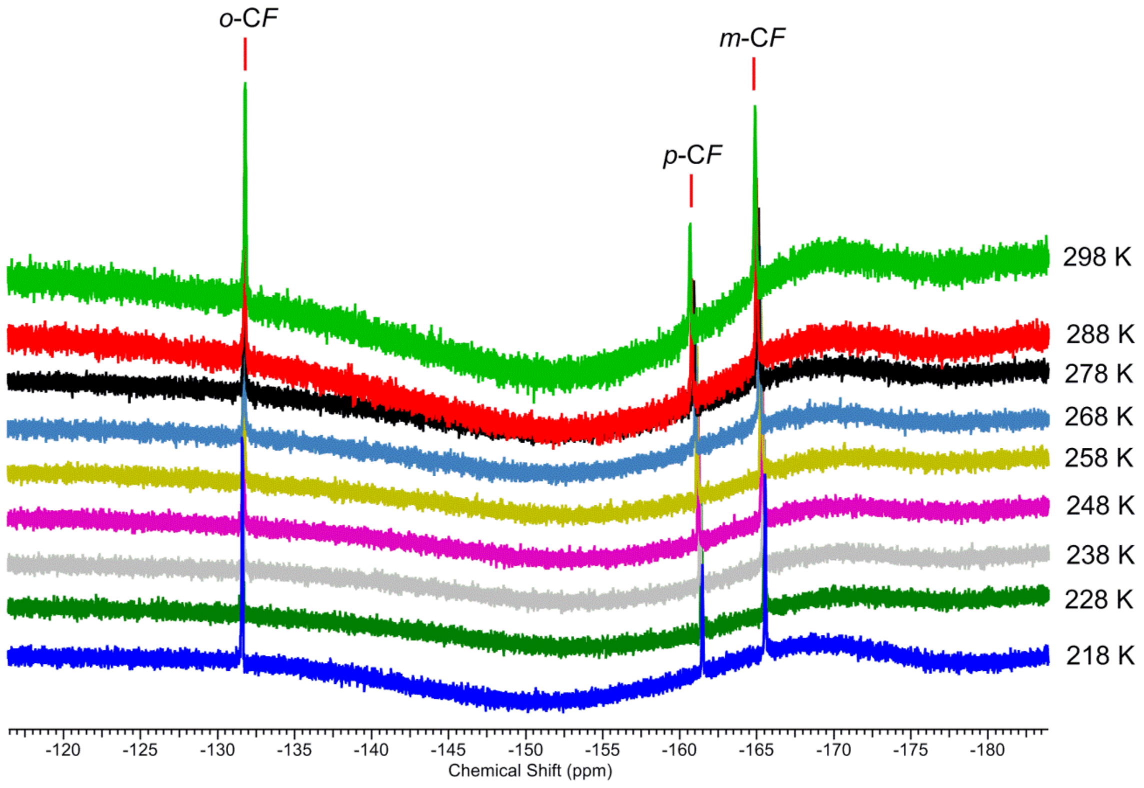

Figure S27. Variable temperature (218 – 298 K) $^{19}F\{^{1}H\}$ NMR spectra of **1-La** in C_7D_8.

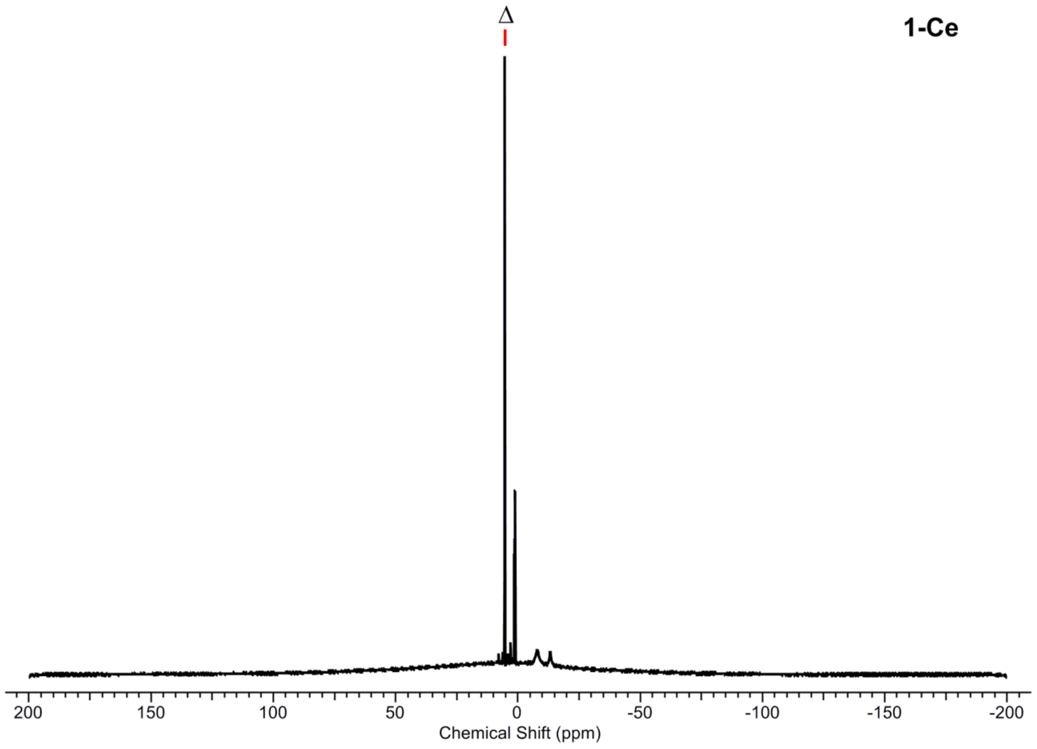

Figure S28. ^{1}H NMR spectrum of complex **1-Ce** in [D_2]DCM. Δ denotes solvent residual.

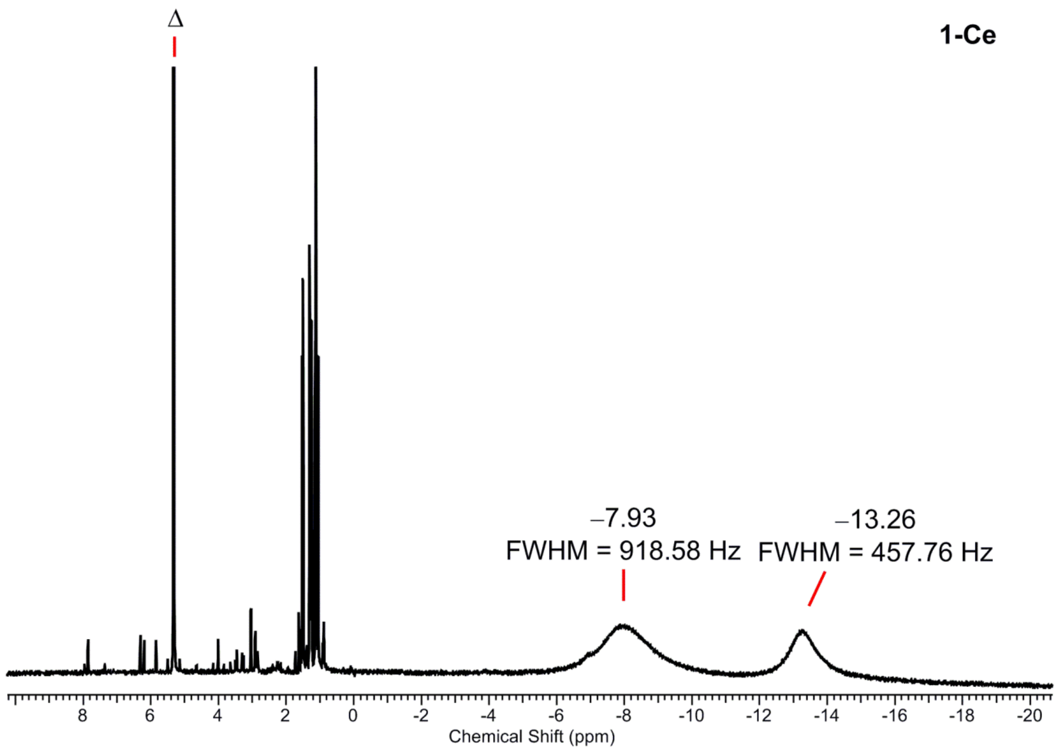

Figure S29. ^{1}H NMR spectrum of complex **1-Ce** in [D$_2$]DCM zoomed in the region –20 to 10 ppm. Δ denotes solvent residual.

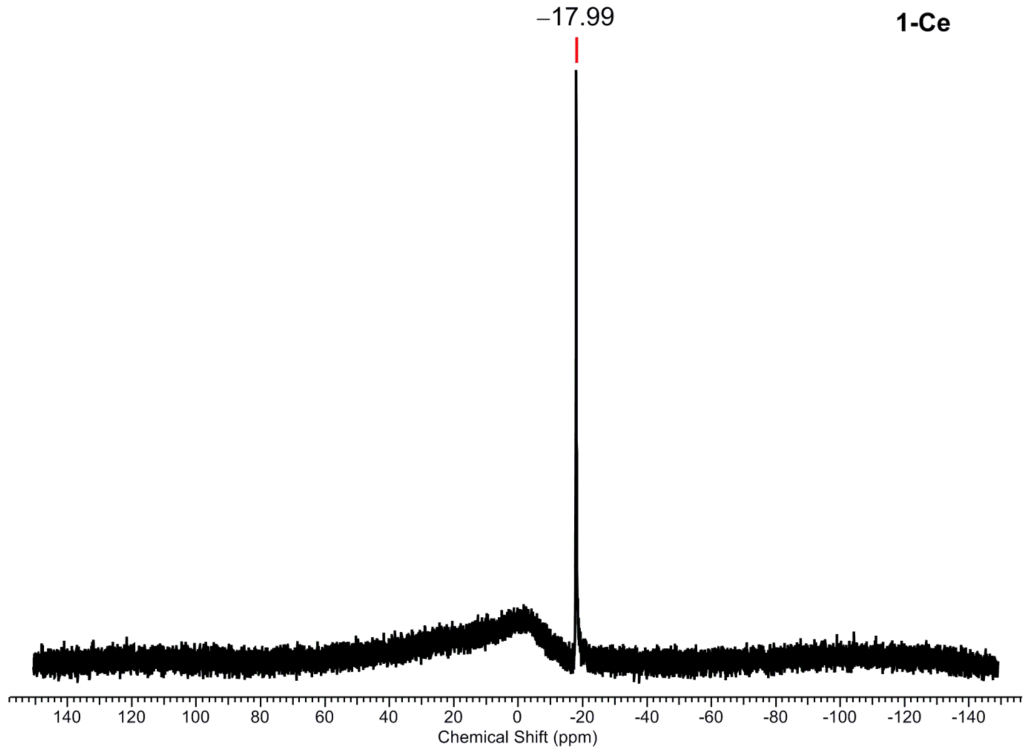

Figure S30. ^{11}B NMR spectrum of complex **1-Ce** in [D$_2$]DCM.

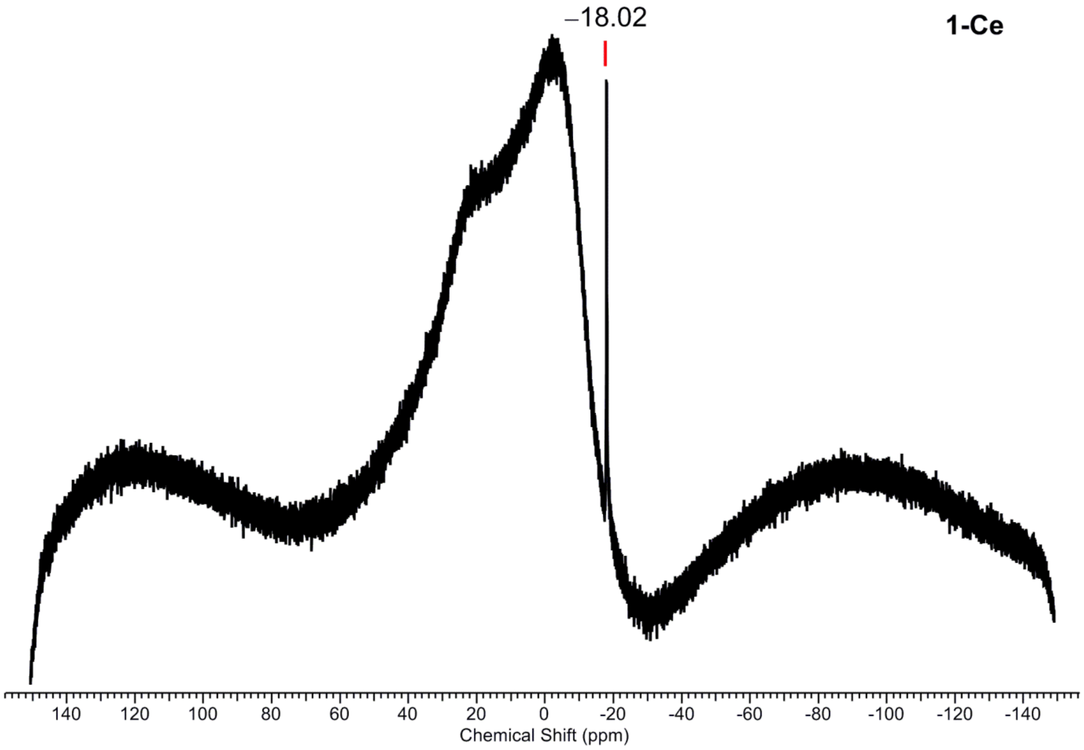

Figure S31. $^{11}B\{^{1}H\}$ NMR spectrum of complex **1-Ce** in [D$_2$]DCM.

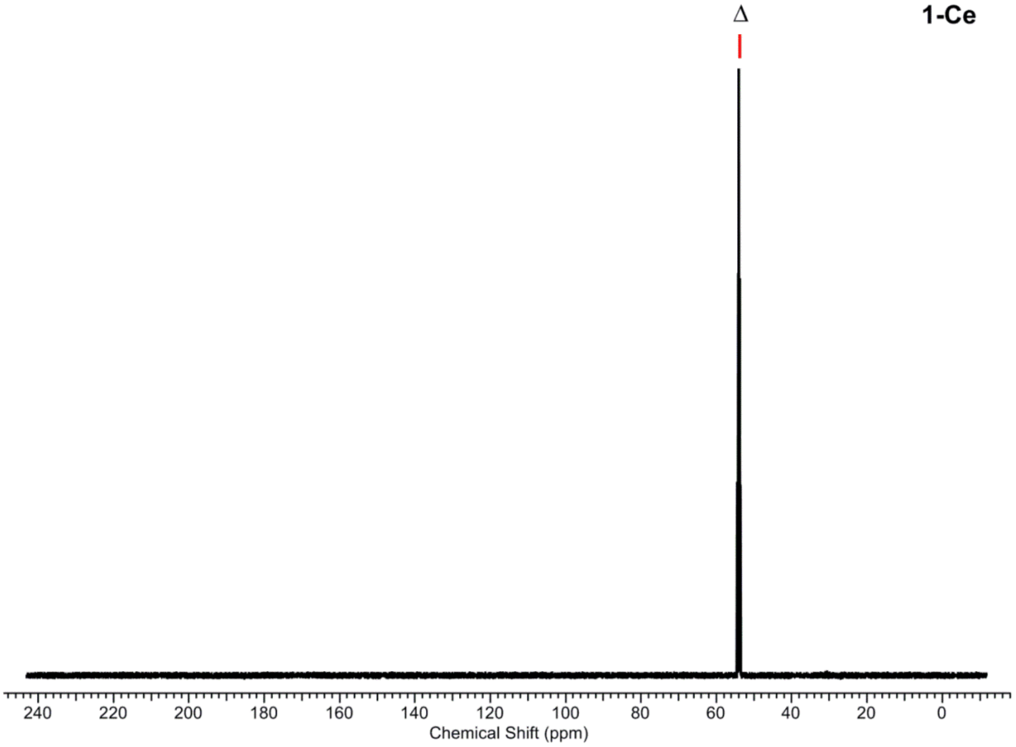

Figure S32. $^{13}C\{^{1}H\}$ NMR spectrum of complex **1-Ce** in [D$_2$]DCM. Δ denotes solvent residual.

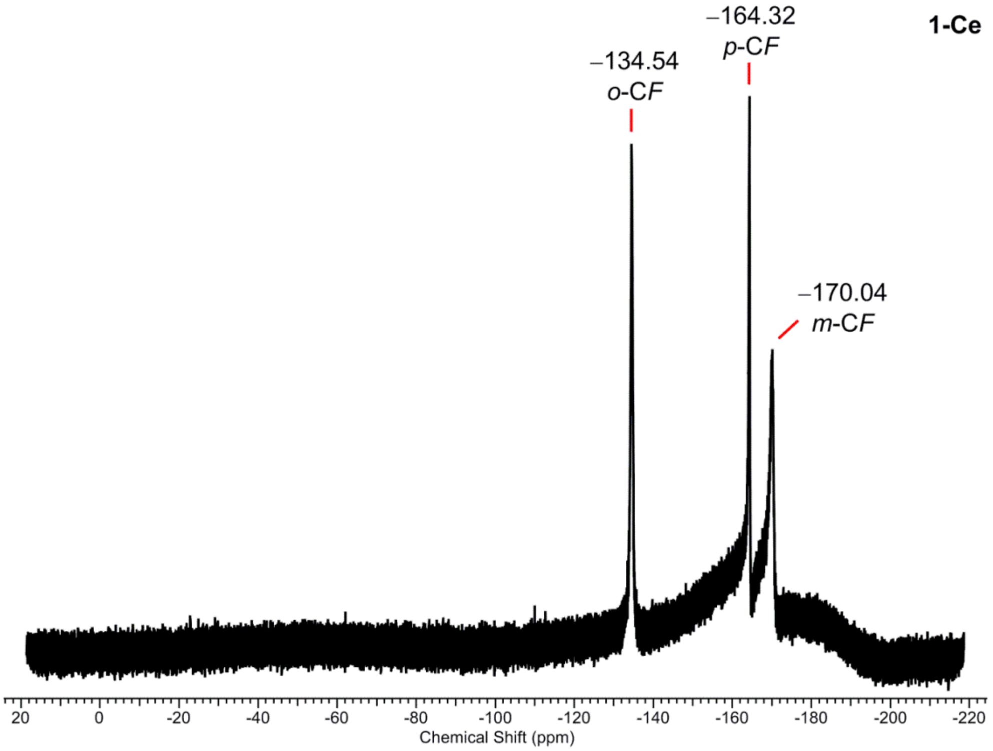

Figure S33. ^{19}F NMR spectrum of complex **1-Ce** in [D_2]DCM.

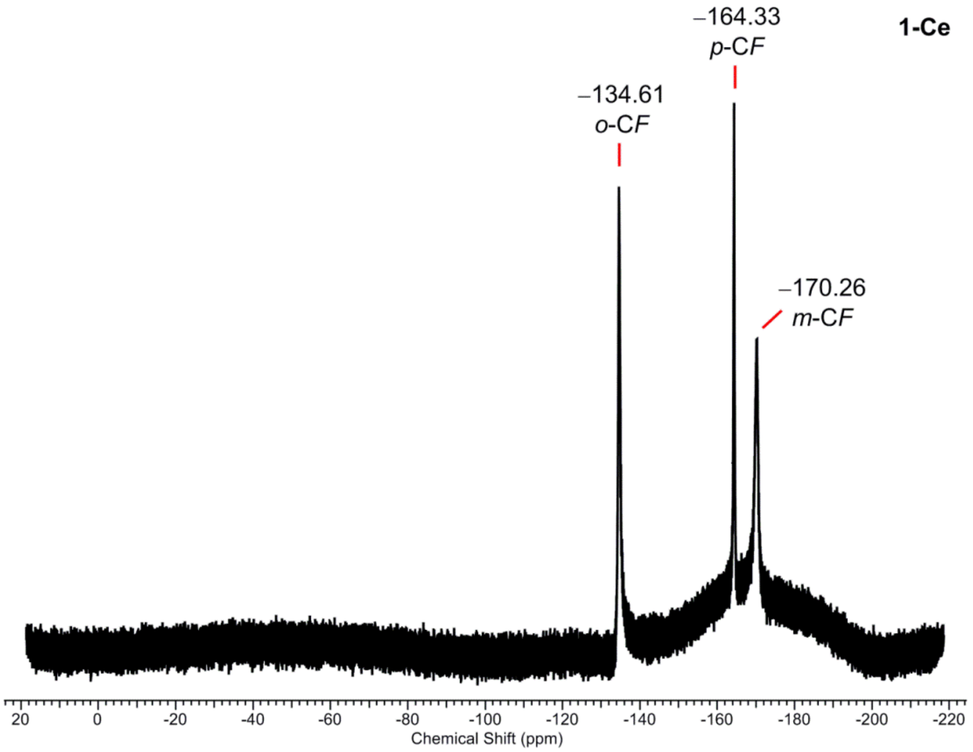

Figure S34. $^{19}F\{^1H\}$ NMR spectrum of complex **1-Ce** in [D_2]DCM.

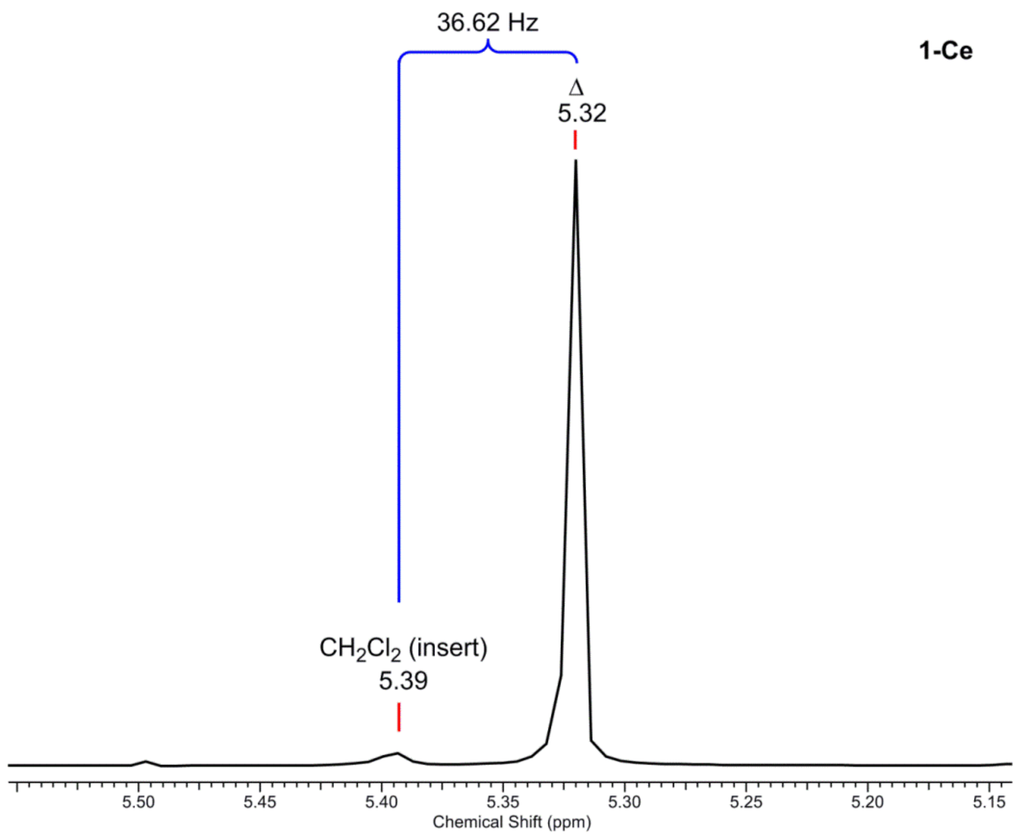

Figure S35. ^{1}H NMR spectrum of complex **1-Ce** in [D_2]DCM with a CH_2Cl_2/[D_2]DCM insert. Δ denotes solvent residual.

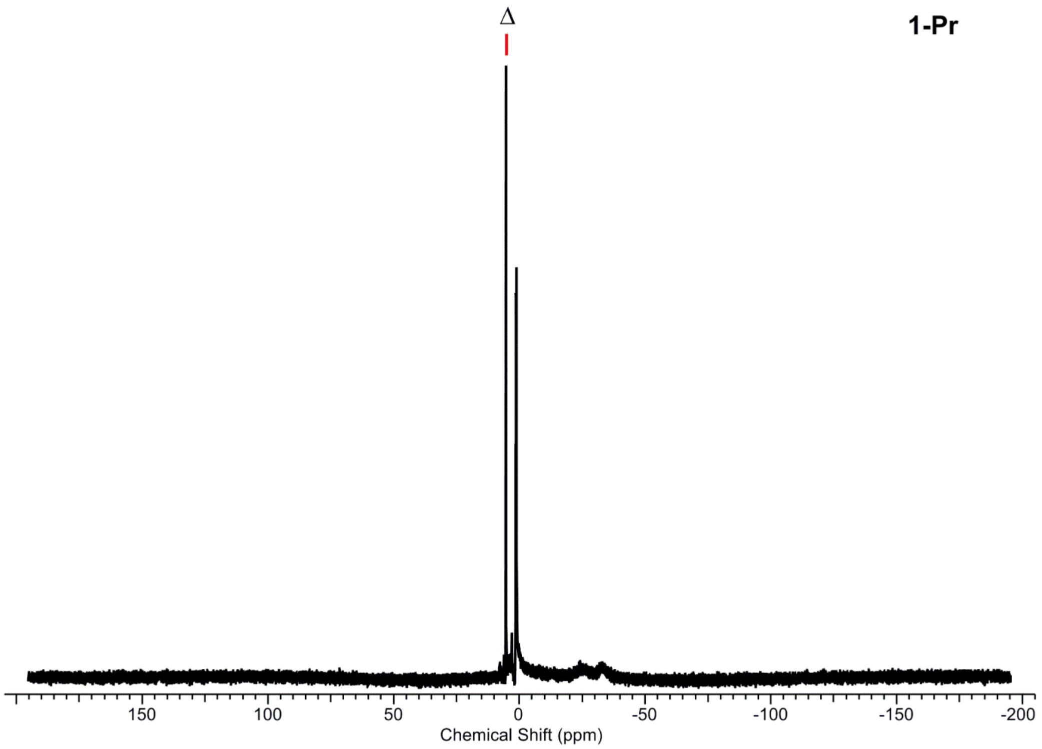

Figure S36. ^{1}H NMR spectrum of complex **1-Pr** in [D_2]DCM. Δ denotes solvent residual.

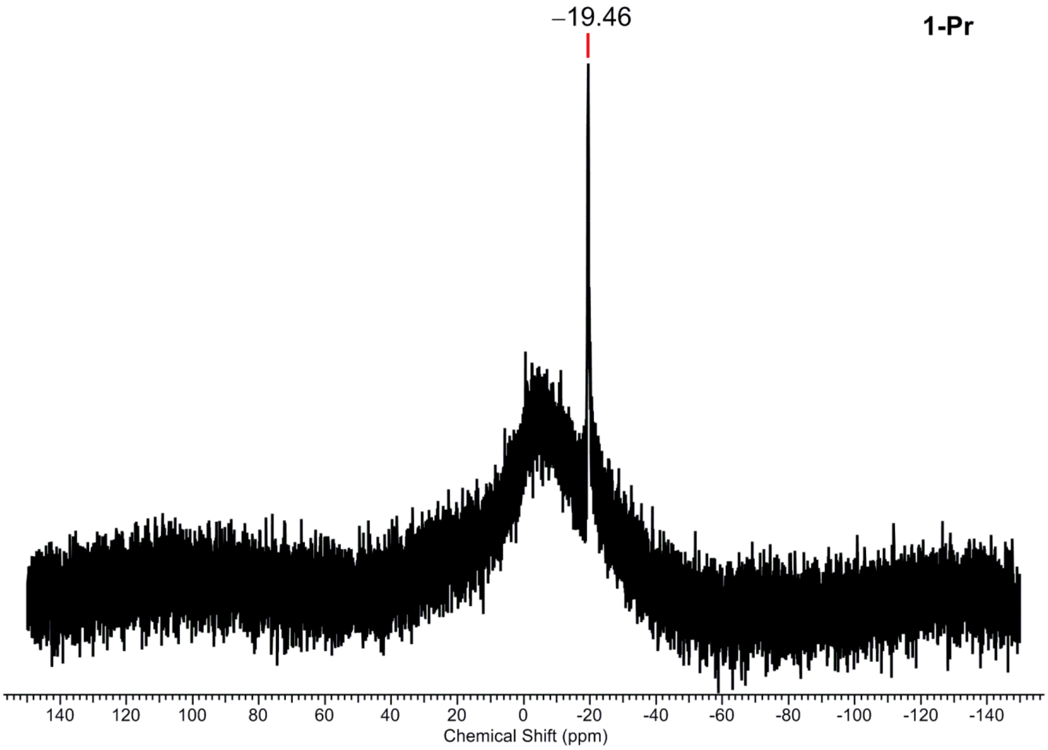

Figure S37. ^{11}B NMR spectrum of complex **1-Pr** in [D_2]DCM.

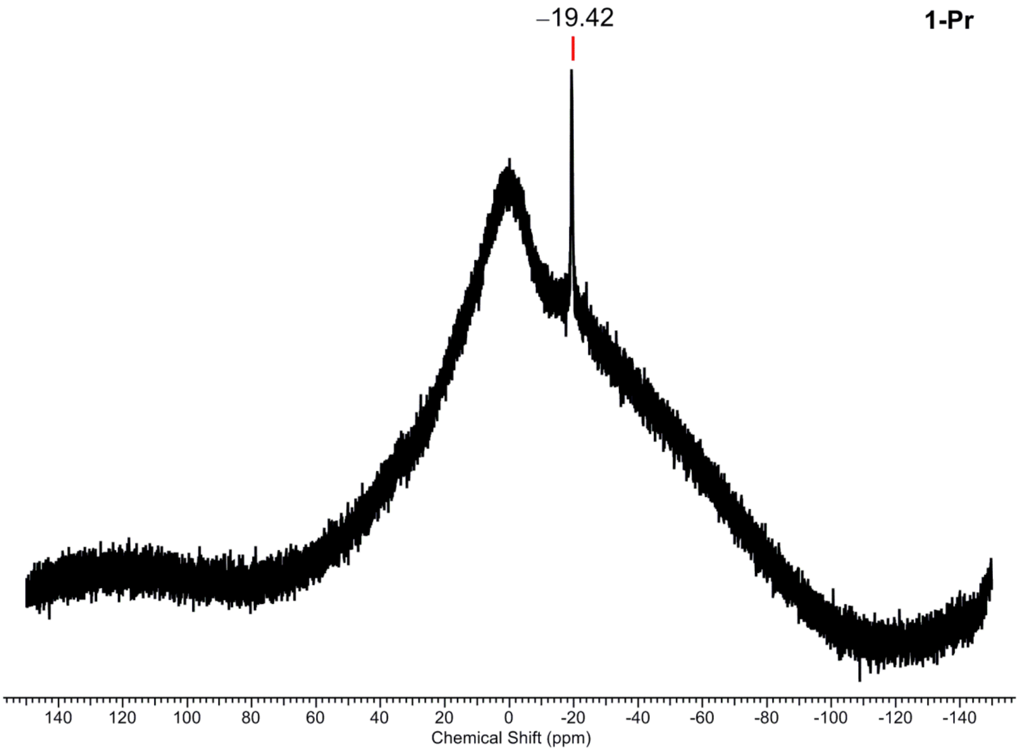

Figure S38. $^{11}B\{^{1}H\}$ NMR spectrum of complex **1-Pr** in [D_2]DCM.

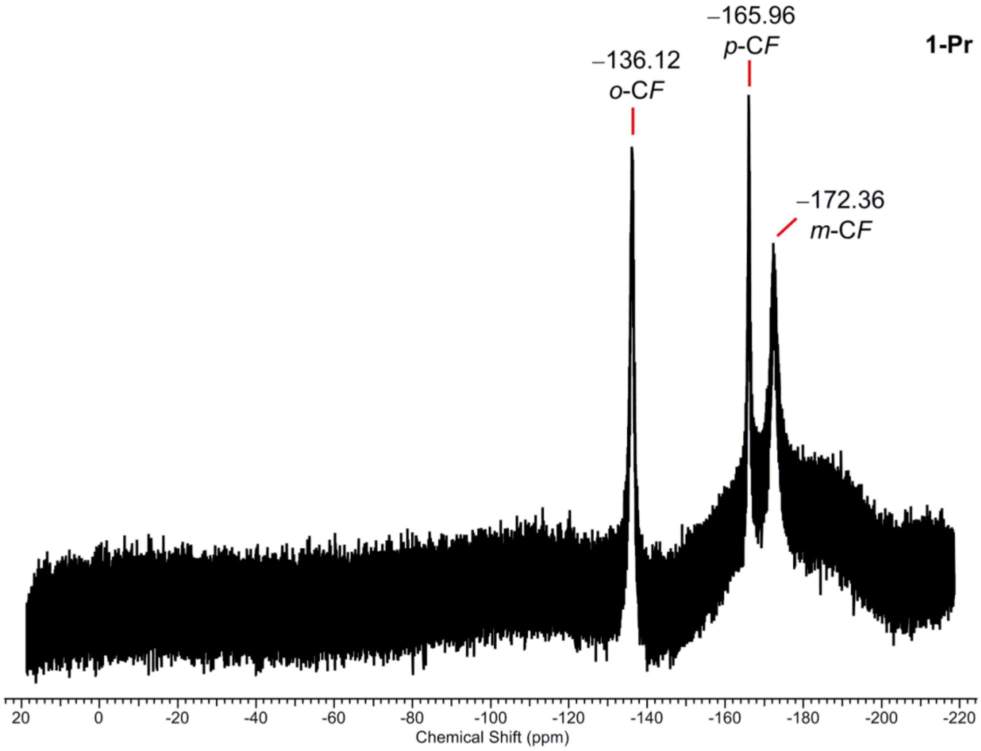

Figure S39. ^{19}F NMR spectrum of complex **1-Pr** in [D$_2$]DCM.

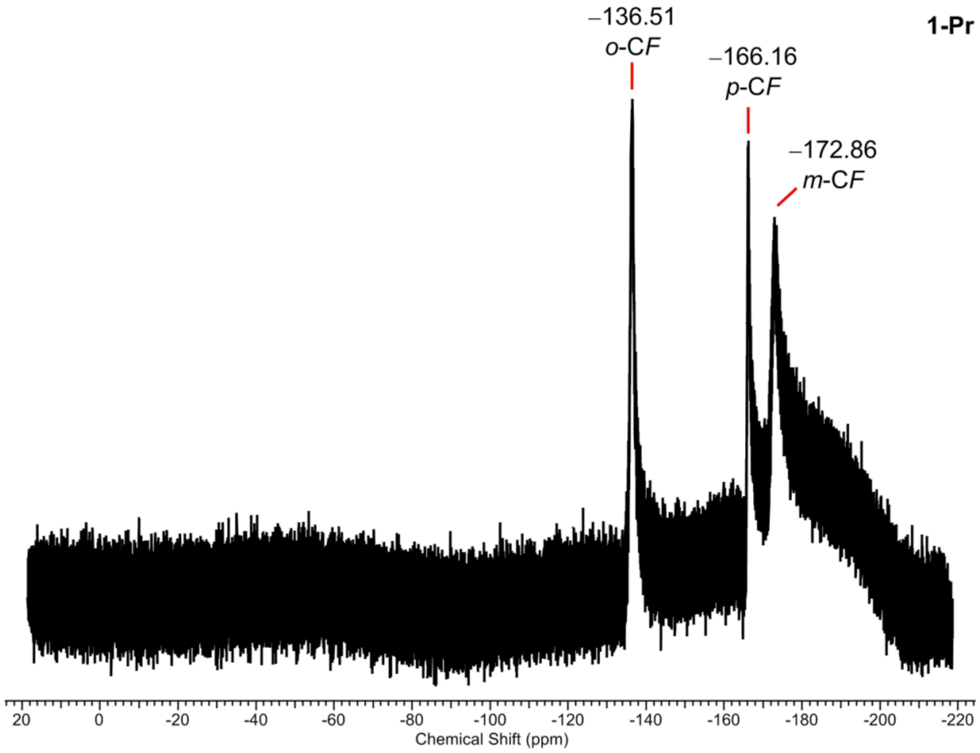

Figure S40. $^{19}F\{^1H\}$ NMR spectrum of complex **1-Pr** in [D$_2$]DCM.

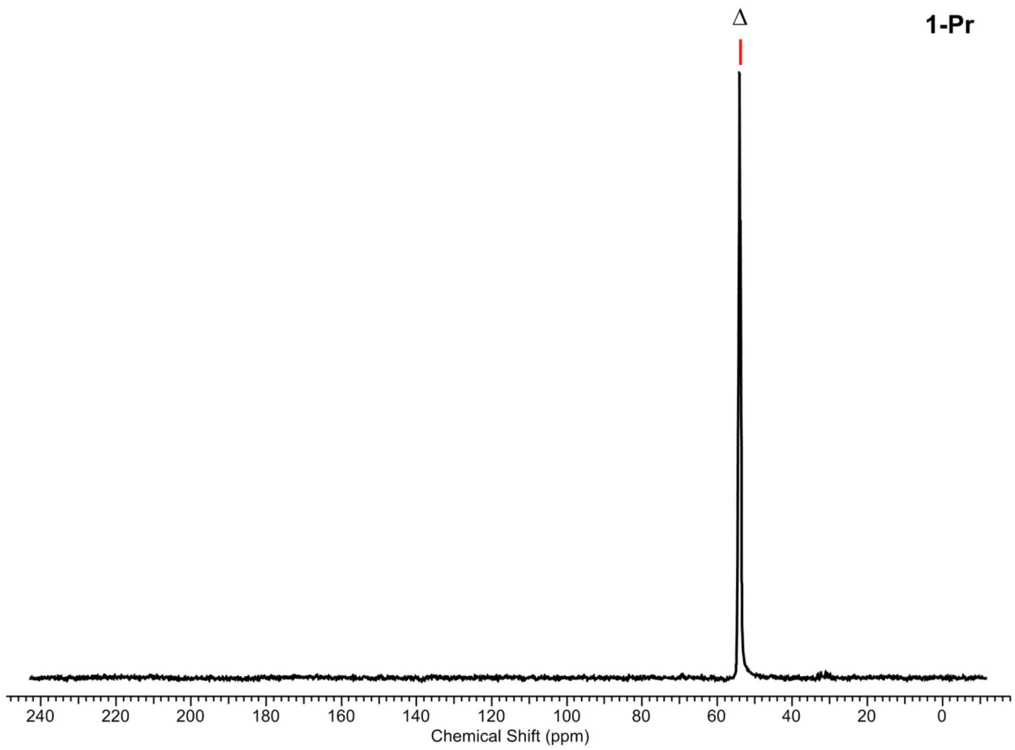

Figure S41. $^{13}C\{^{1}H\}$ NMR spectrum of complex **1-Pr** in $[D_2]DCM$. Δ denotes solvent residual.

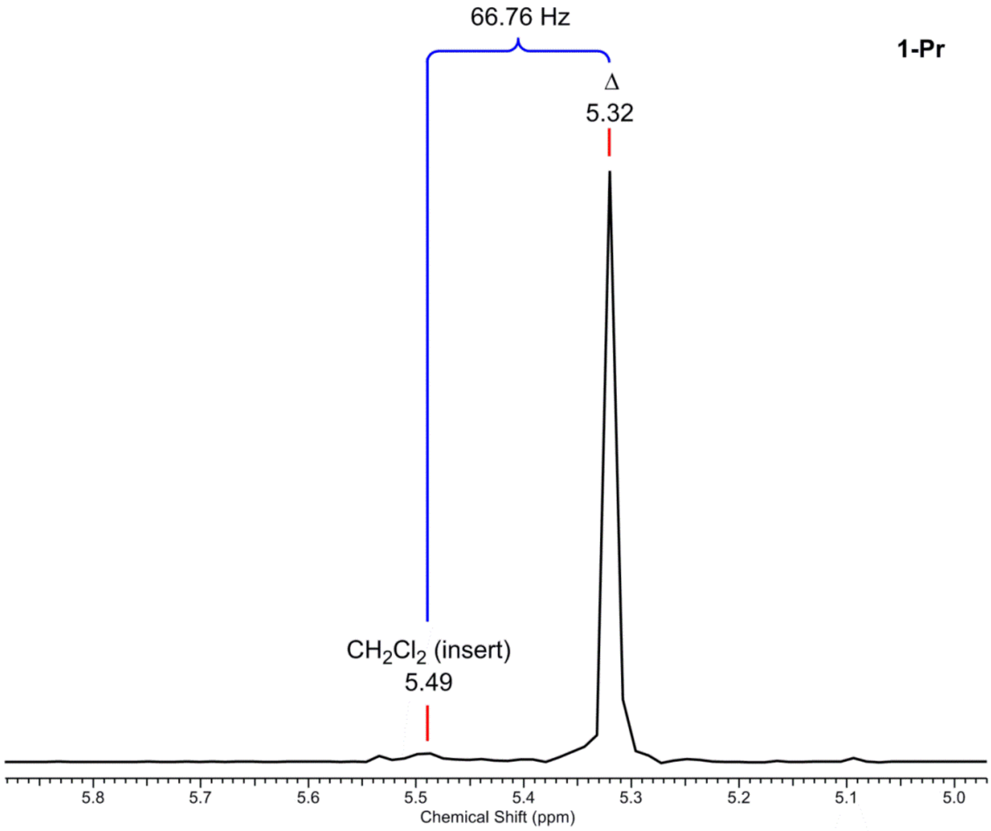

Figure S42. ^{1}H NMR spectrum of complex **1-Pr** in $[D_2]DCM$ with a $CH_2Cl_2/[D_2]DCM$ insert. Δ denotes solvent residual.

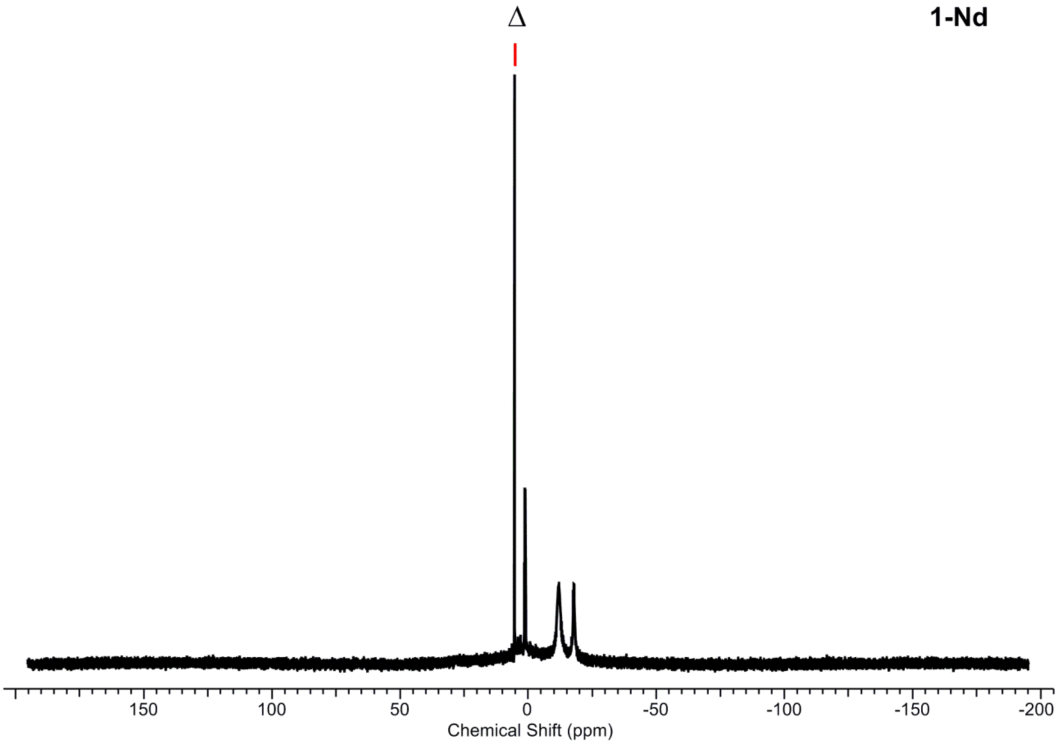

Figure S43. ^{1}H NMR spectrum of complex **1-Nd** in [D$_2$]DCM. Δ denotes solvent residual.

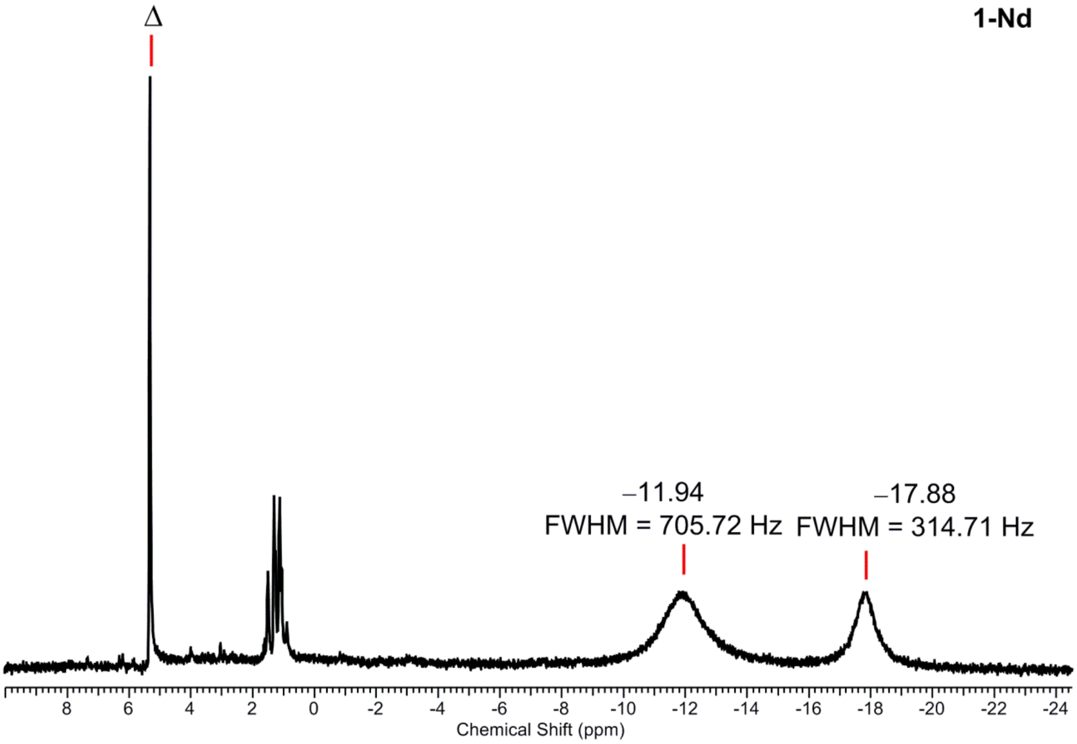

Figure S44. ^{1}H NMR spectrum of complex **1-Nd** in [D$_2$]DCM zoomed in the region –24 and 10 ppm. Δ denotes solvent residual.

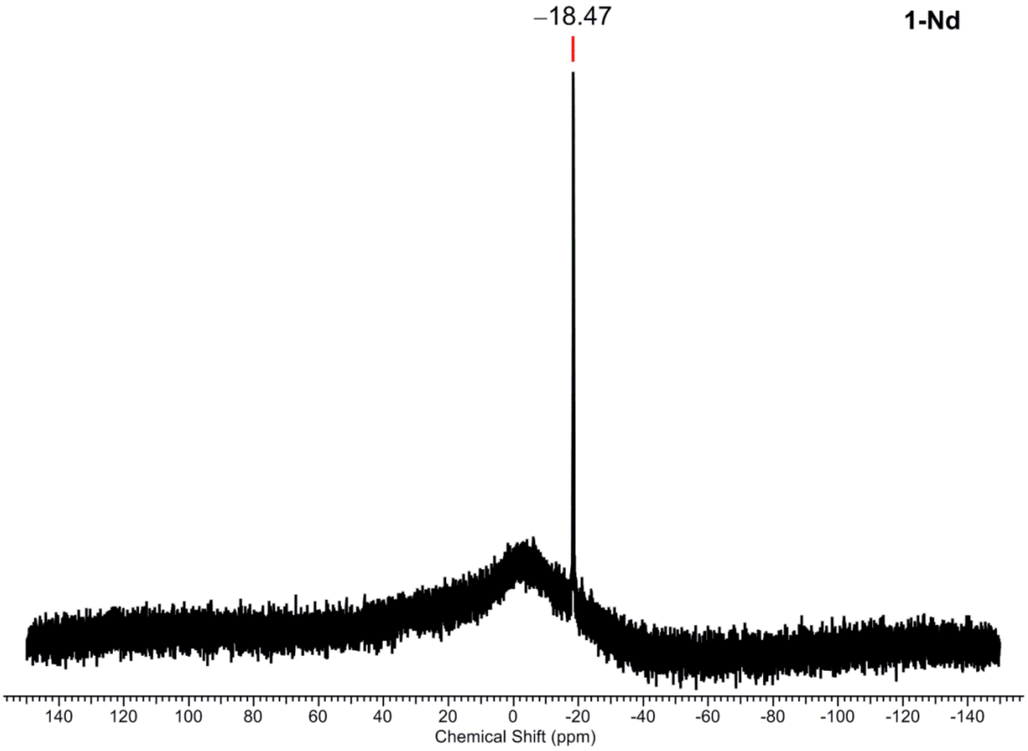

Figure S45. ^{11}B NMR spectrum of complex **1-Nd** in [D_2]DCM.

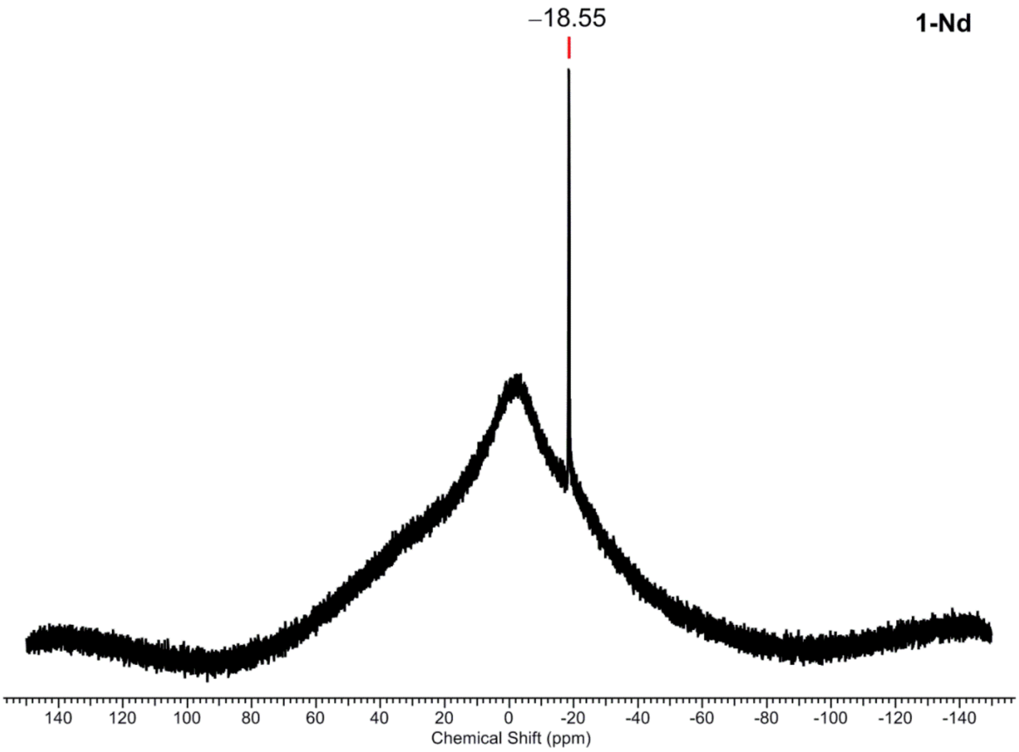

Figure S46. $^{11}B\{^{1}H\}$ NMR spectrum of complex **1-Nd** in [D_2]DCM.

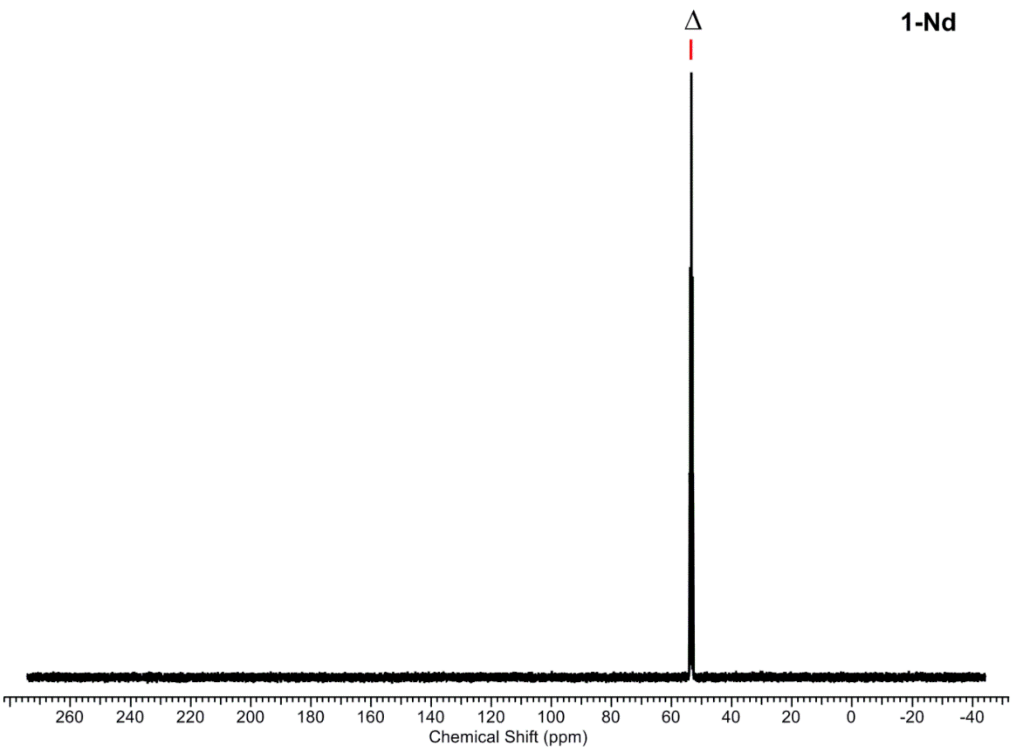

Figure S47. $^{13}C\{^{1}H\}$ NMR spectrum of complex **1-Nd** in [D$_2$]DCM. Δ denotes solvent residual.

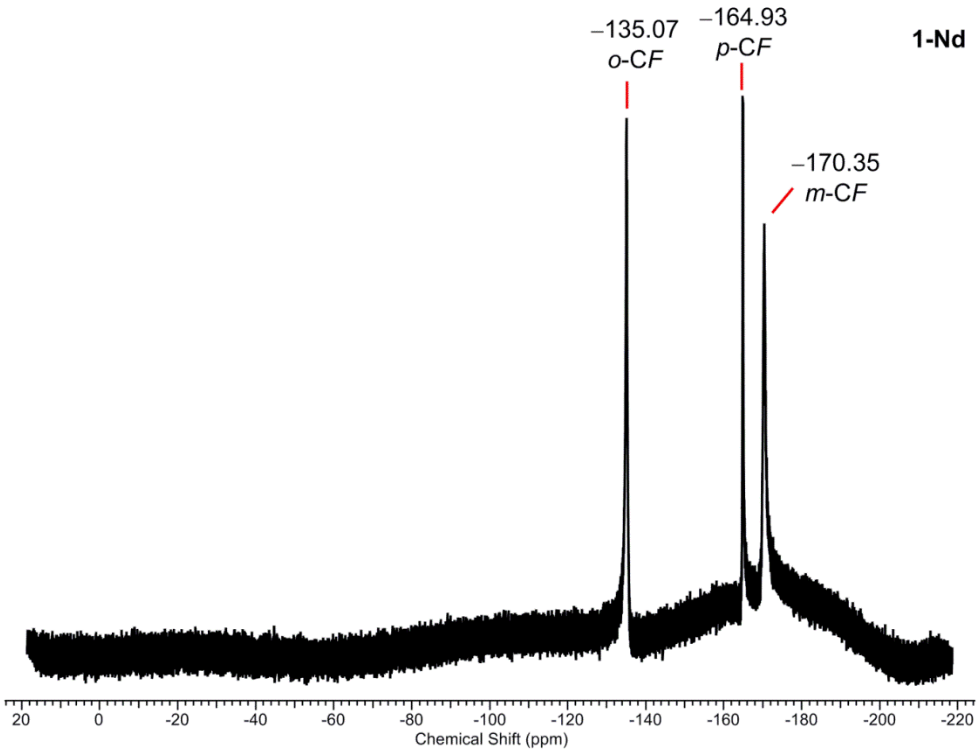

Figure S48. ^{19}F NMR spectrum of complex **1-Nd** in [D$_2$]DCM.

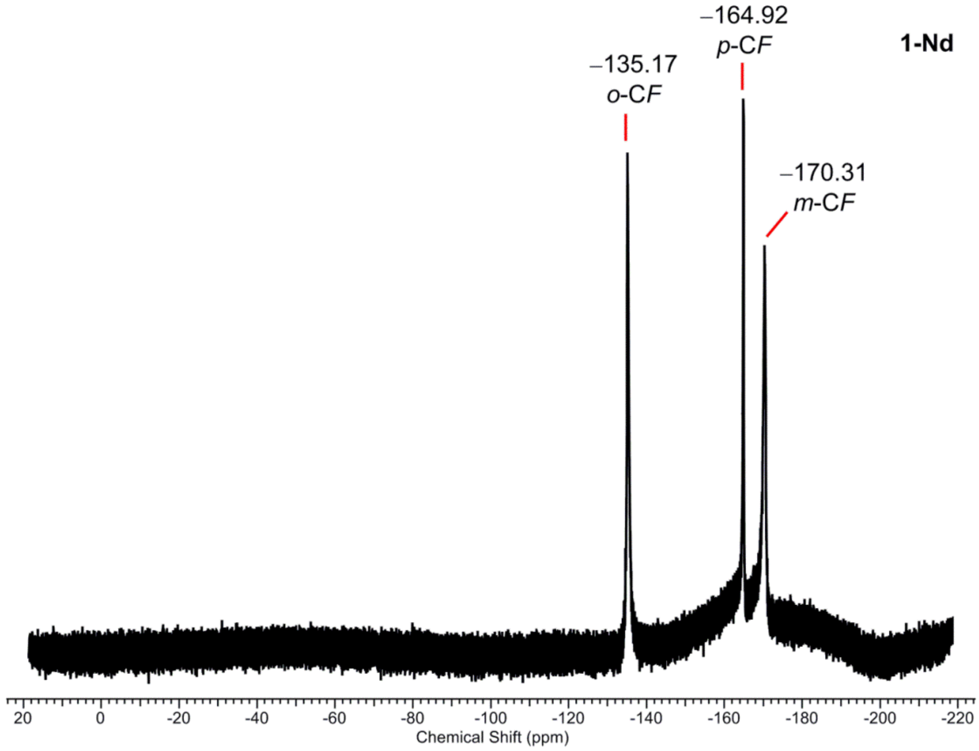

Figure S49. $^{19}F\{^{1}H\}$ NMR spectrum of complex **1-Nd** in $[D_2]DCM$.

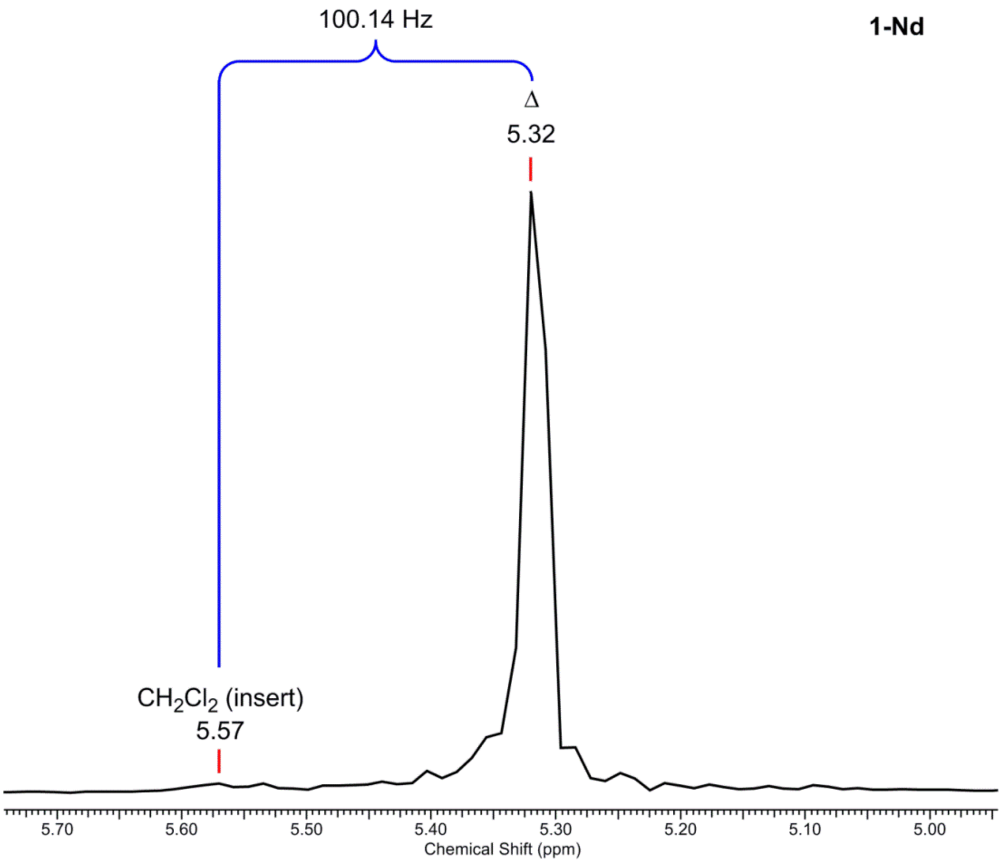

Figure S50. ^{1}H NMR spectrum of complex **1-Nd** in $[D_2]DCM$ with a $CH_2Cl_2/[D_2]DCM$ insert. Δ denotes solvent residual.

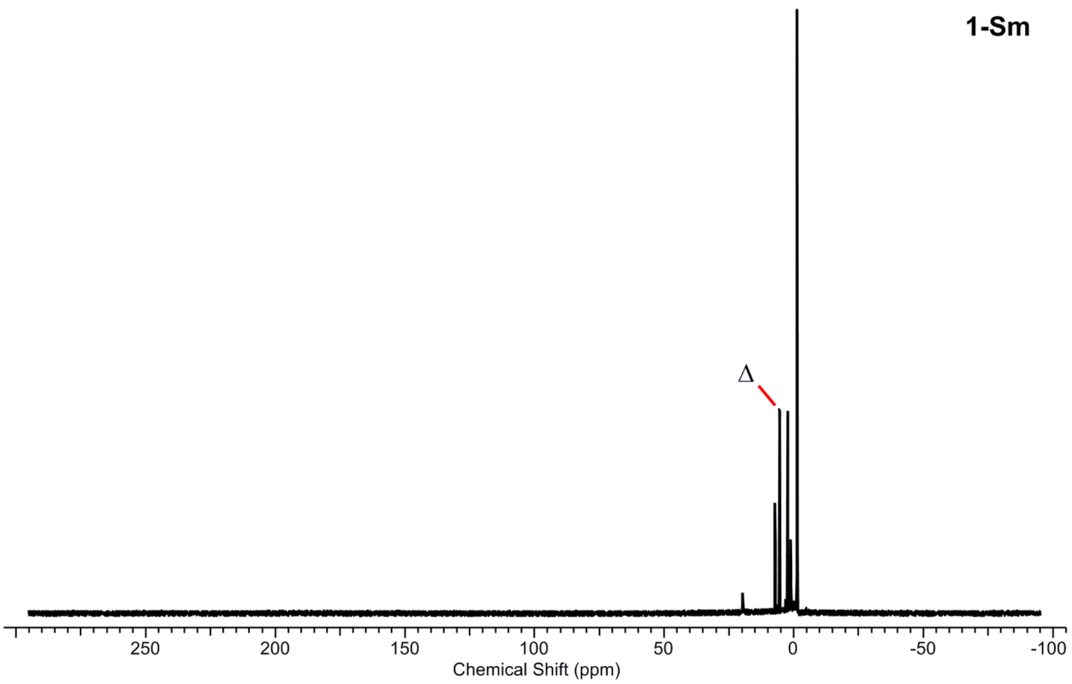

Figure S51. ^{1}H NMR spectrum of complex **1-Sm** in [D_2]DCM. Δ denotes solvent residual.

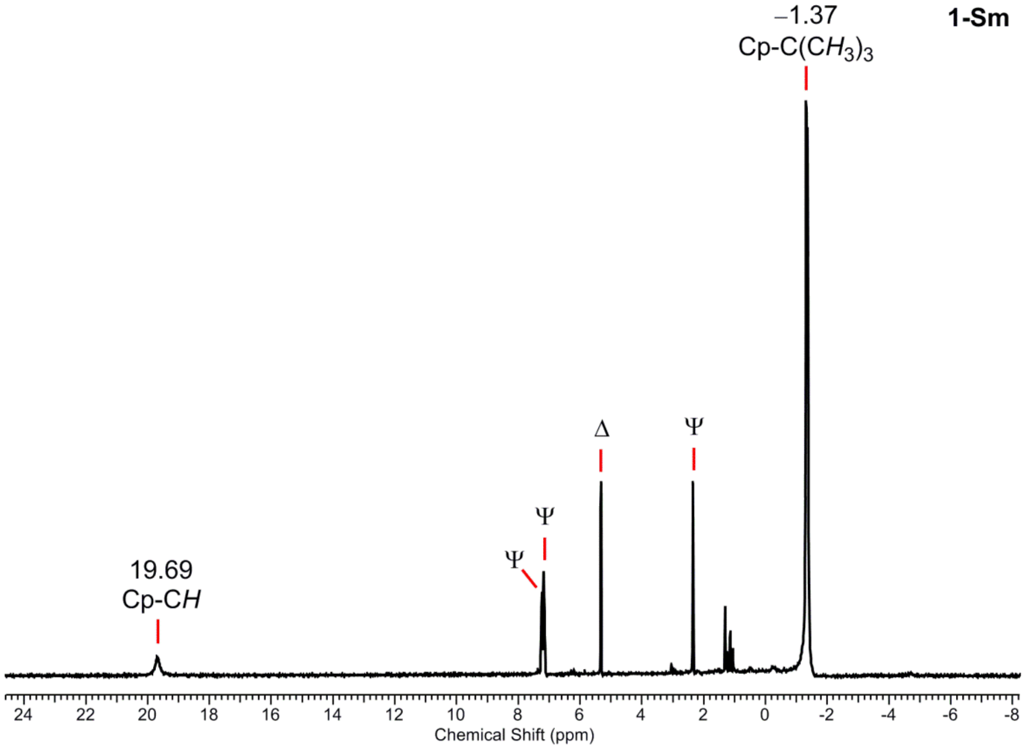

Figure S52. ^{1}H NMR spectrum of complex **1-Sm** in [D_2]DCM zoomed in the region –8 and 24 ppm. Δ denotes solvent residual. Ψ denotes toluene of recrystallization.

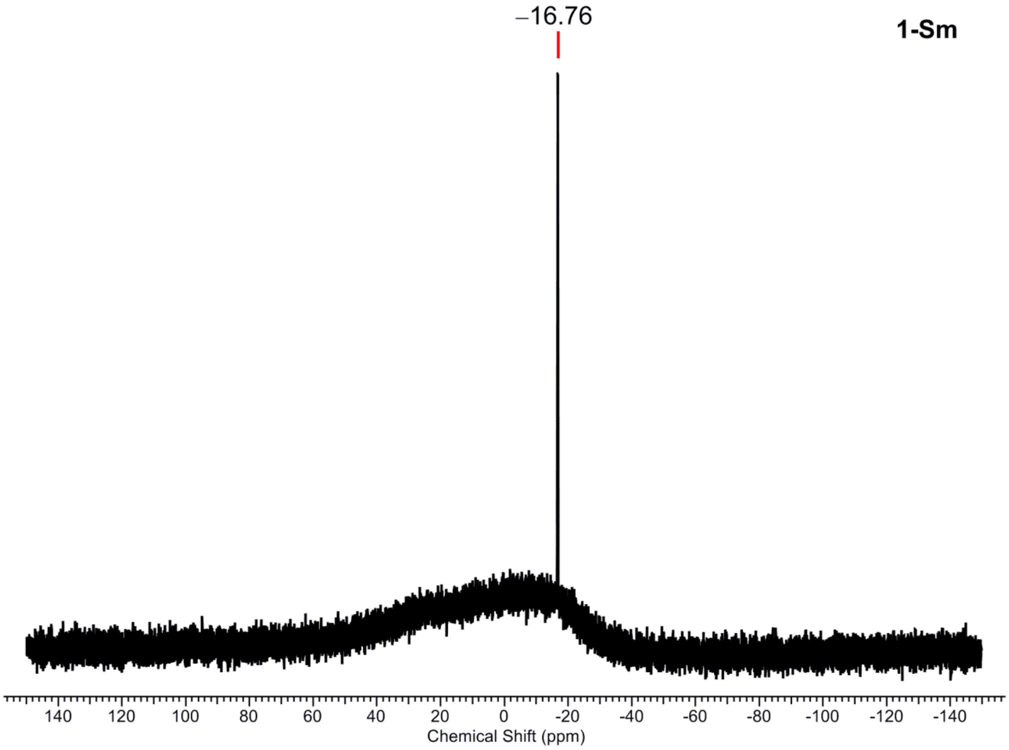

Figure S53. ^{11}B NMR spectrum of complex **1-Sm** in [D_2]DCM.

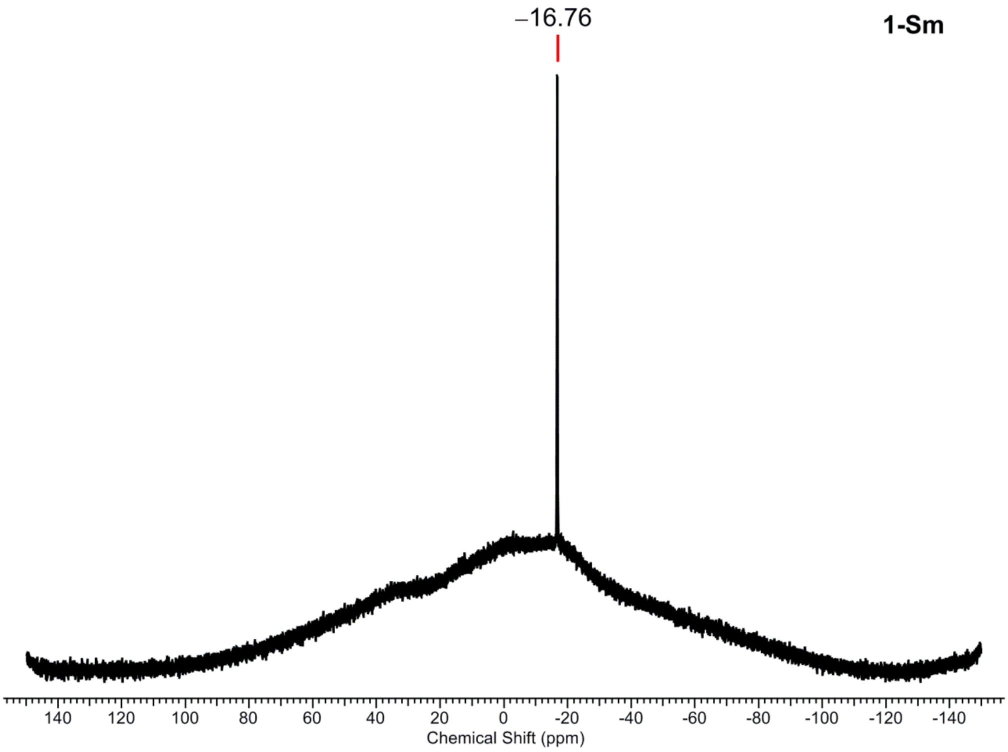

Figure S54. $^{11}B\{^{1}H\}$ NMR spectrum of complex **1-Sm** in [D_2]DCM.

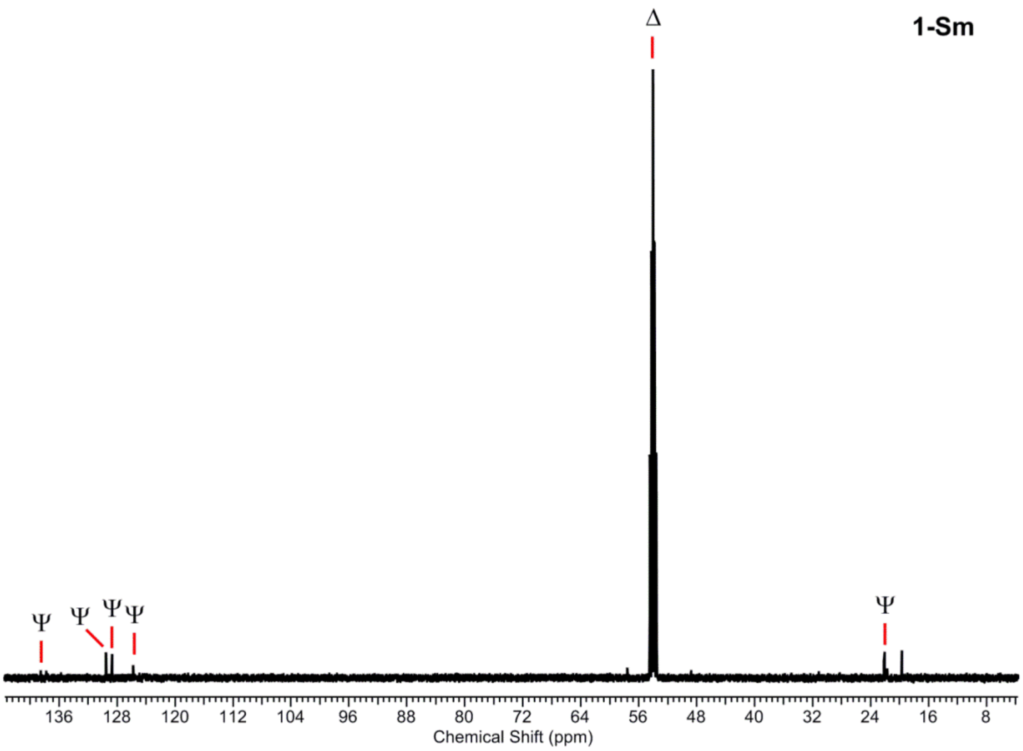

Figure S55. $^{13}C\{^{1}H\}$ NMR spectrum of complex **1-Sm** in [D$_2$]DCM. Δ denotes solvent residual, Ψ denotes toluene of crystallization.

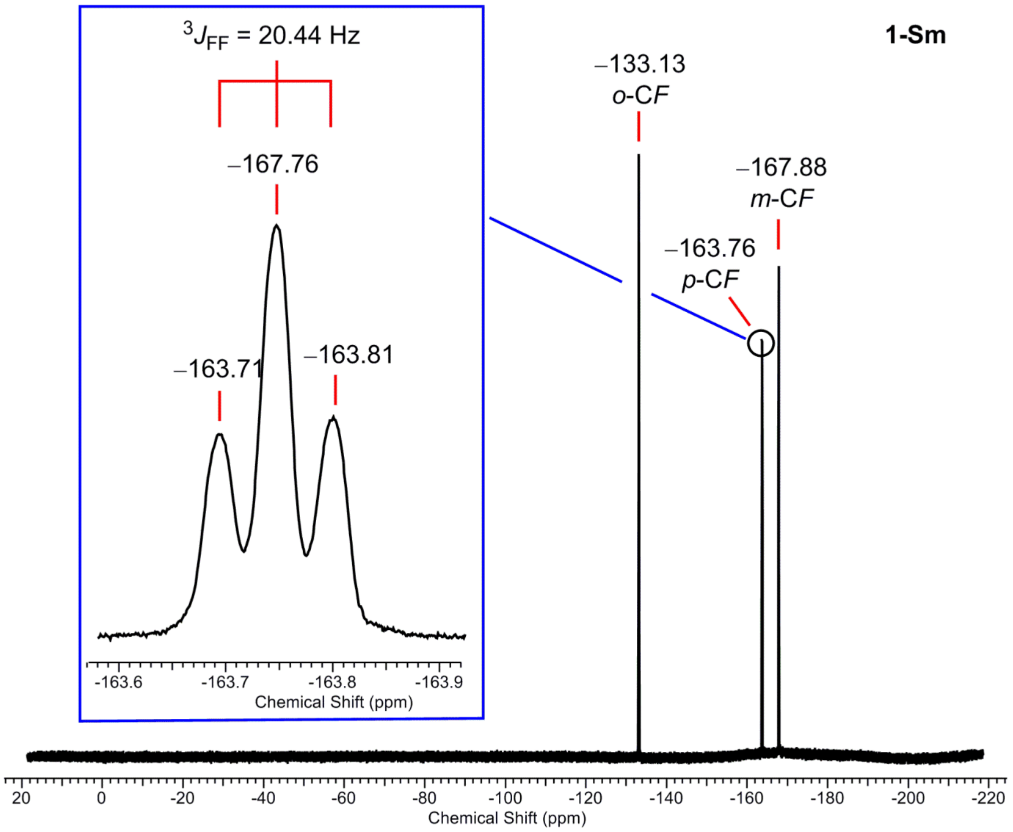

Figure S56. ^{19}F NMR spectrum of complex **1-Sm** in [D$_2$]DCM.

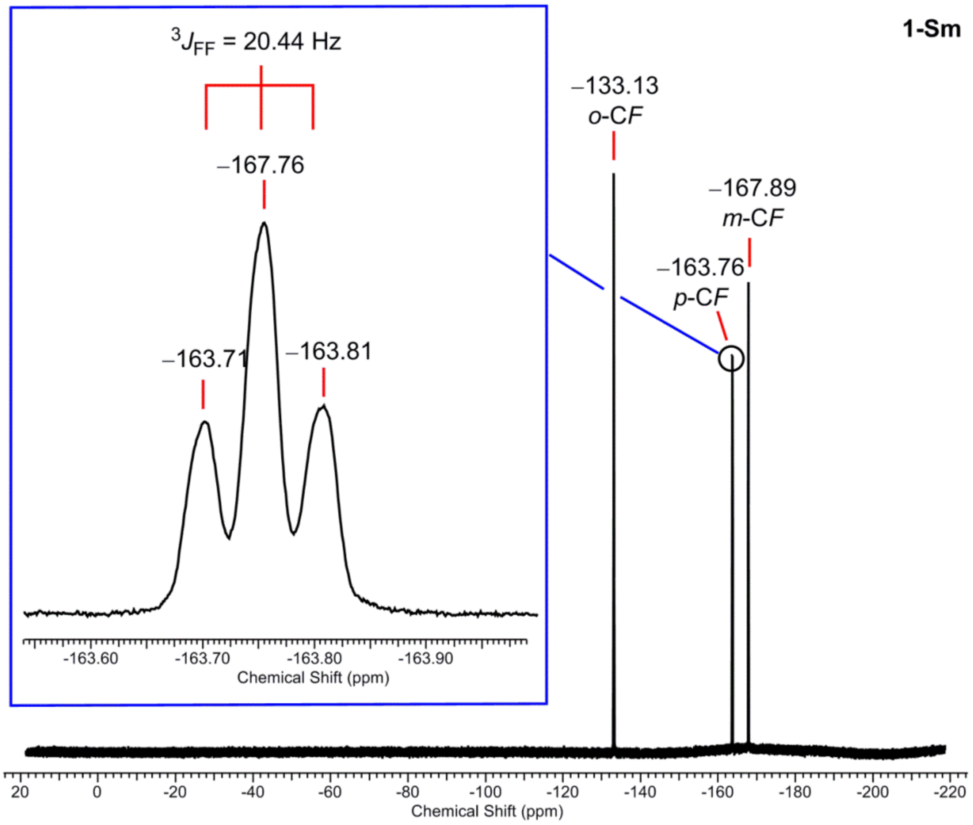

Figure S57. $^{19}F\{^1H\}$ NMR spectrum of complex **1-Sm** in [D$_2$]DCM.

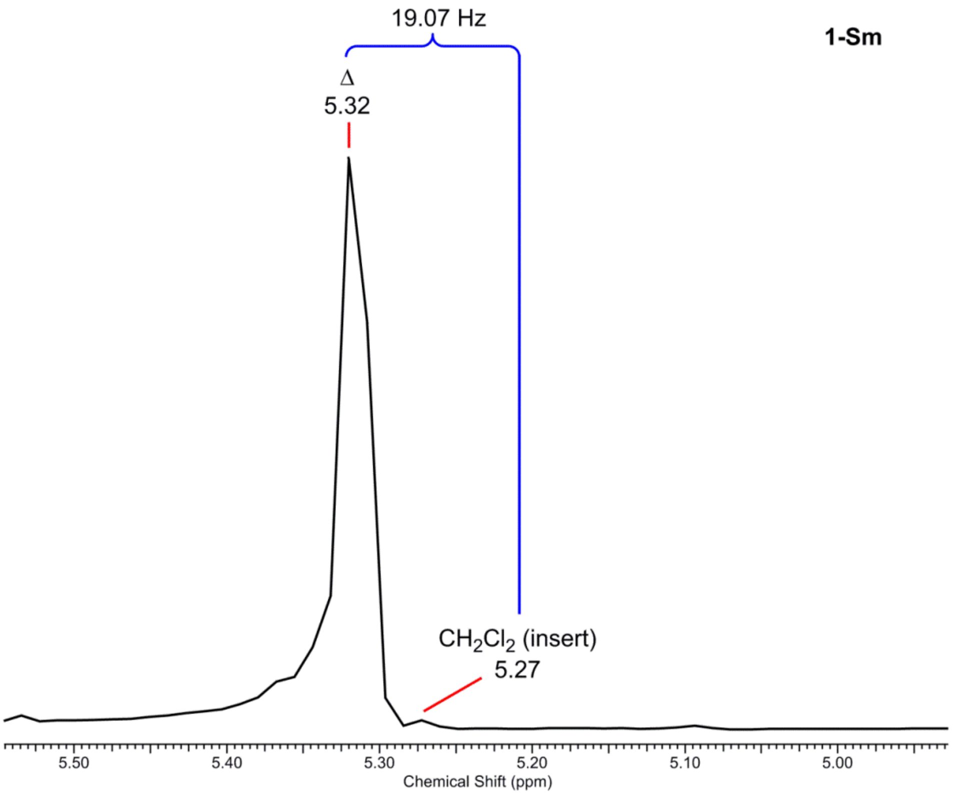

Figure S58. ^1H NMR spectrum of complex **1-Sm** in [D$_2$]DCM with a CH_2Cl_2/[D$_2$]DCM insert. Δ denotes solvent residual.

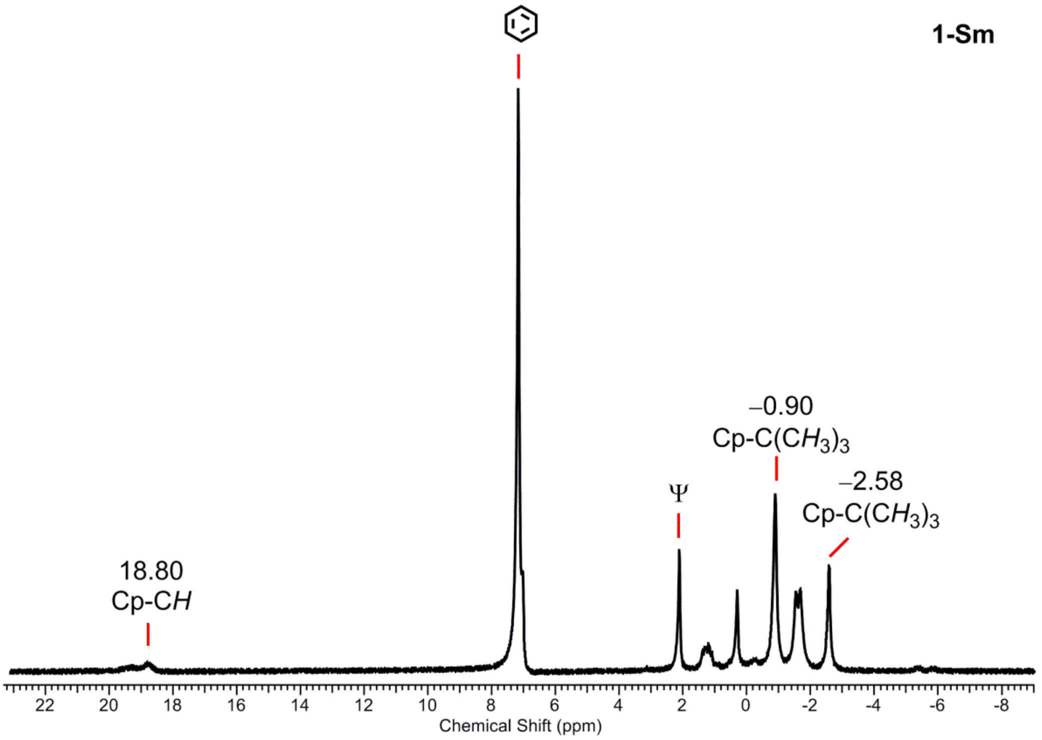

Figure S59. ^{1}H NMR spectrum of complex **1-Sm** in [D_6]benzene zoomed in the region –9 and 23 ppm. Ψ denotes toluene of crystallization.

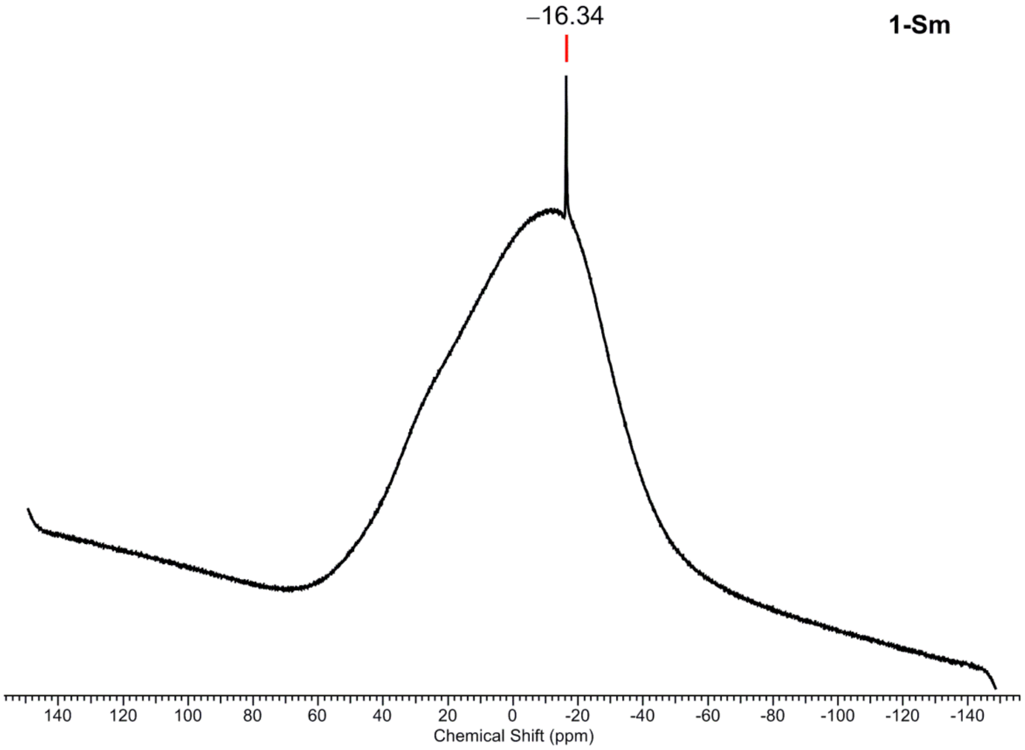

Figure S60. ^{11}B NMR spectrum of complex **1-Sm** in [D_6]benzene.

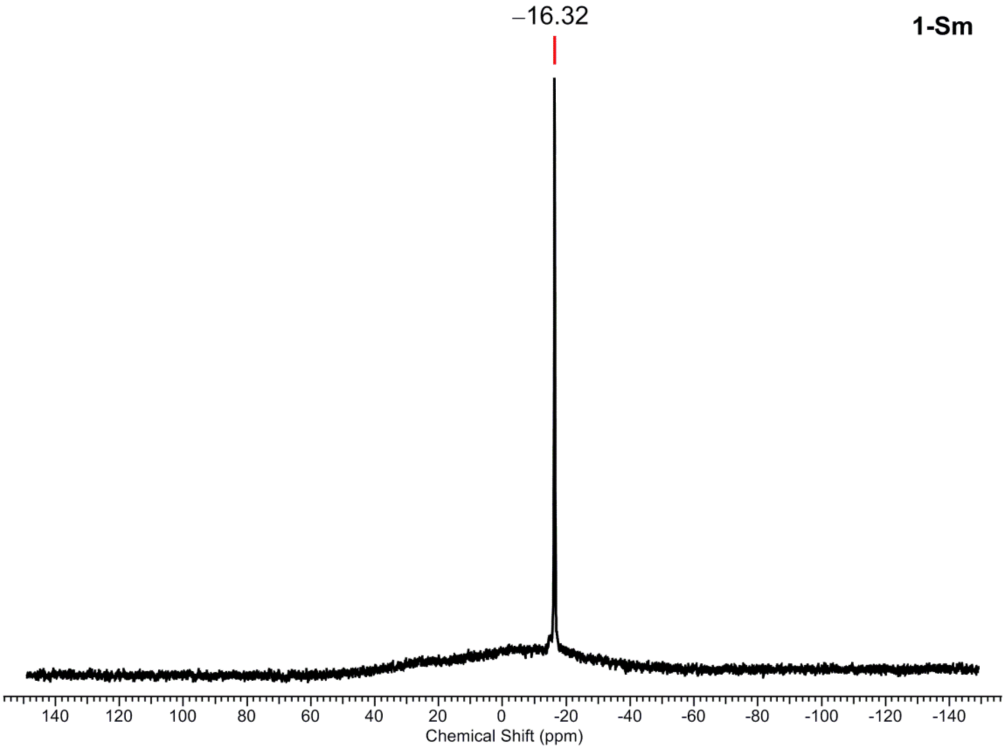

Figure S61. $^{11}B\{^{1}H\}$ NMR spectrum of complex **1-Sm** in [D_6]benzene.

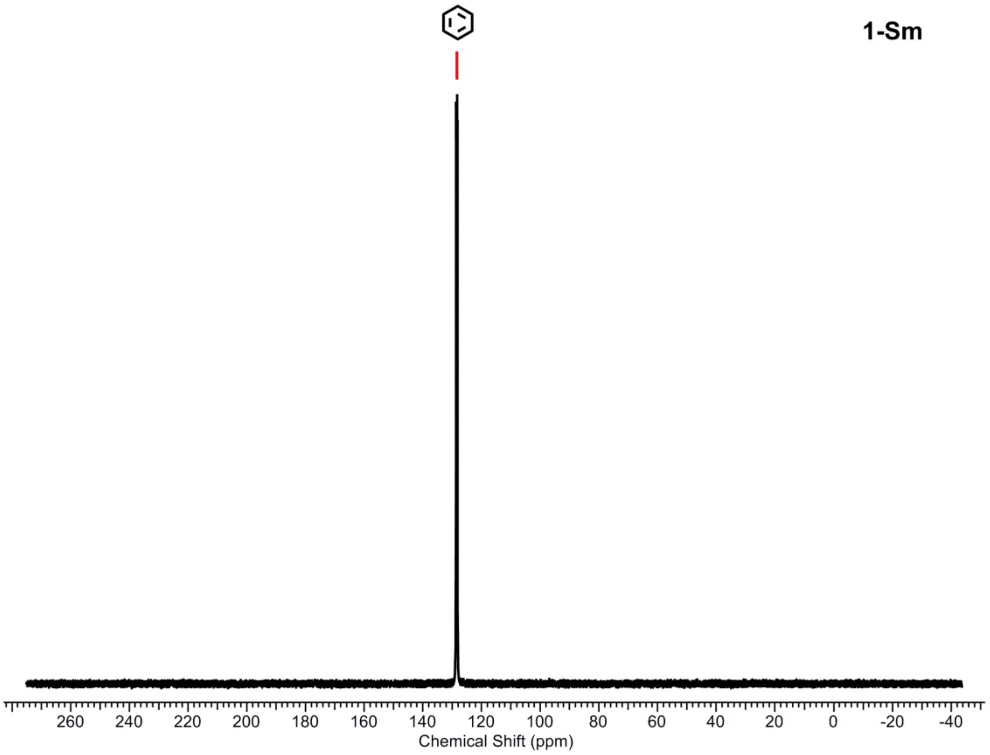

Figure S62. $^{13}C\{^{1}H\}$ NMR spectrum of complex **1-Sm** in [D_6]benzene.

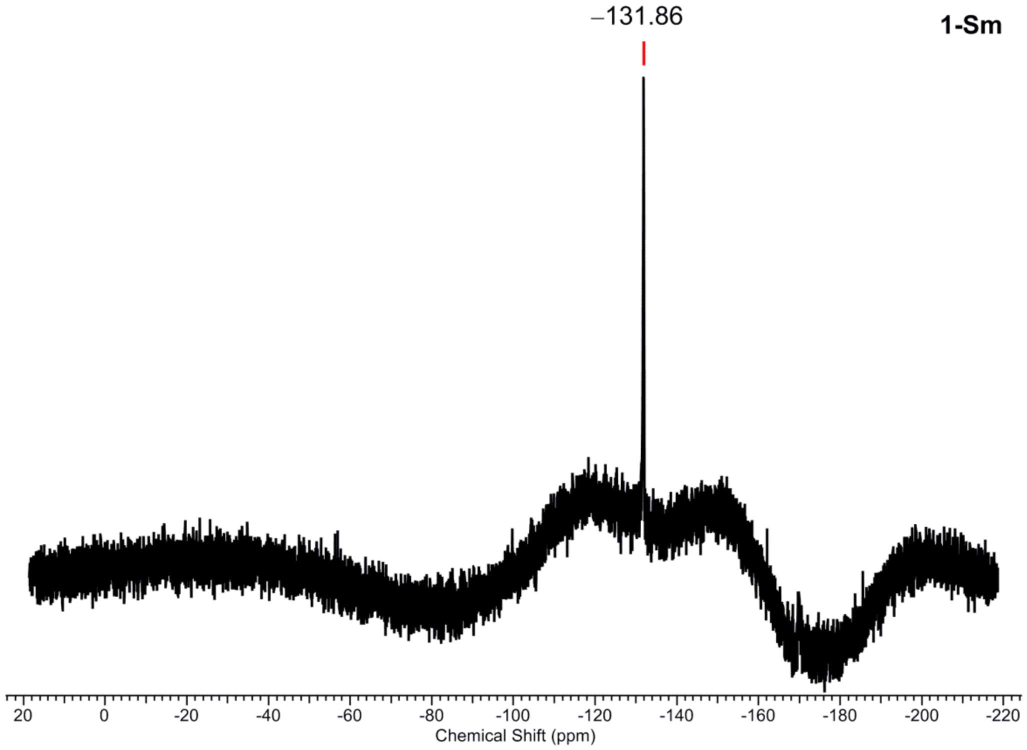

Figure S63. ^{19}F NMR spectrum of complex **1-Sm** in [D$_6$]benzene.

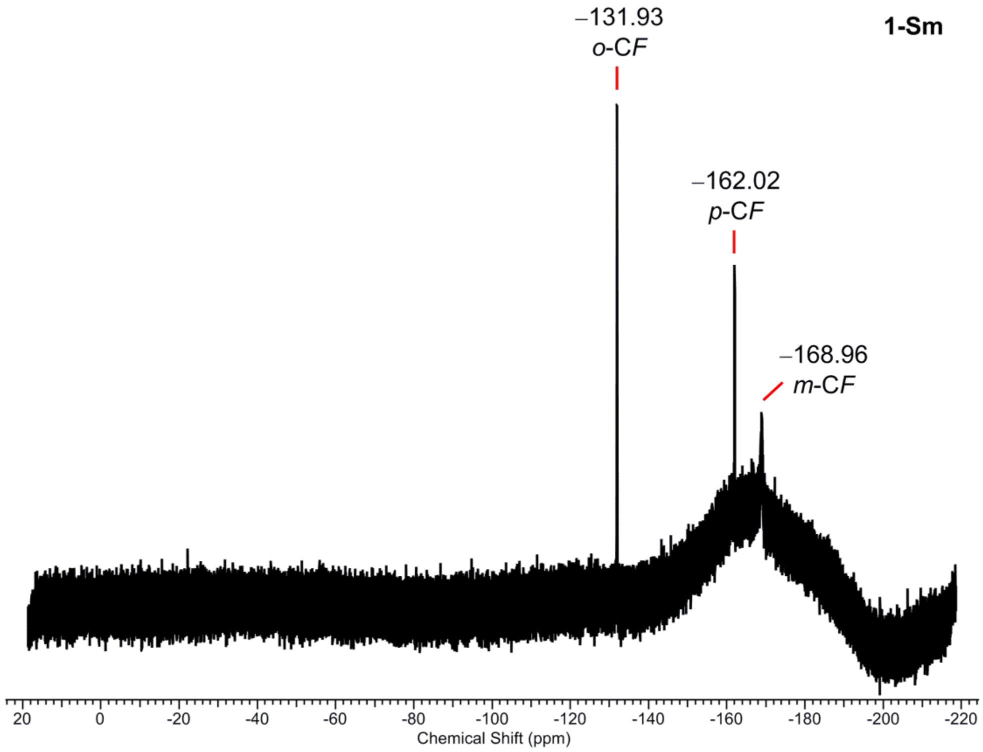

Figure S64. $^{19}F\{^1H\}$ NMR spectrum of complex **1-Sm** in [D$_6$]benzene.

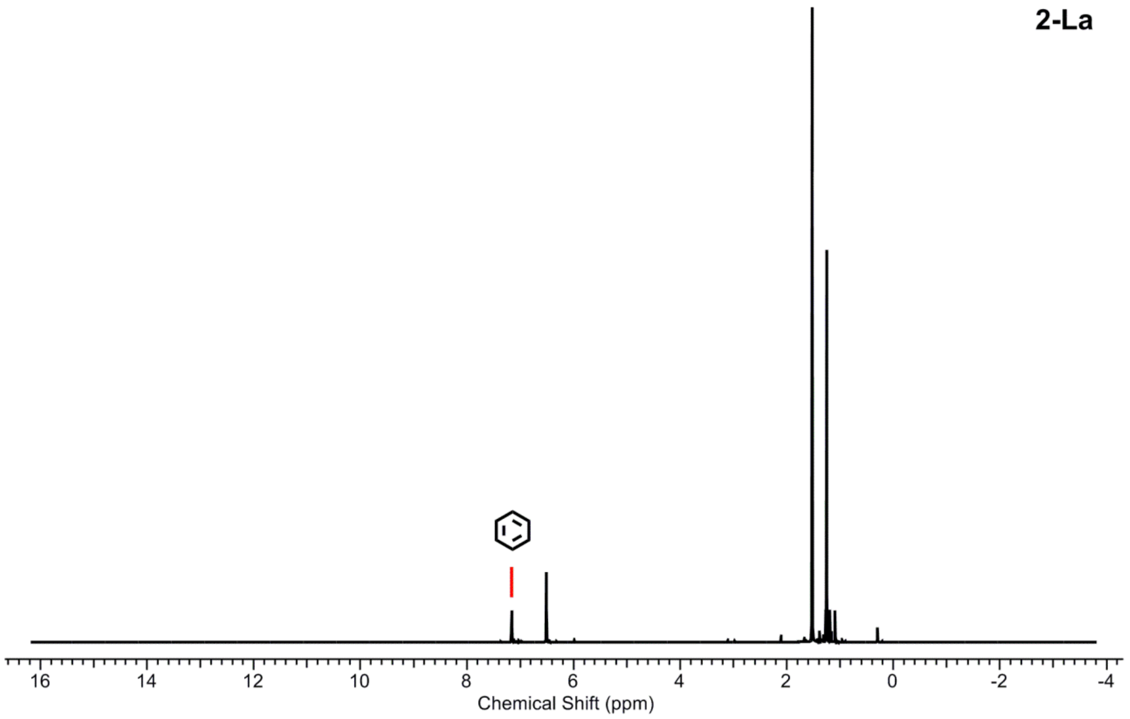

Figure S65. ^{1}H NMR spectrum of complex **2-La** in [D$_6$]benzene. Solvent residual marked.

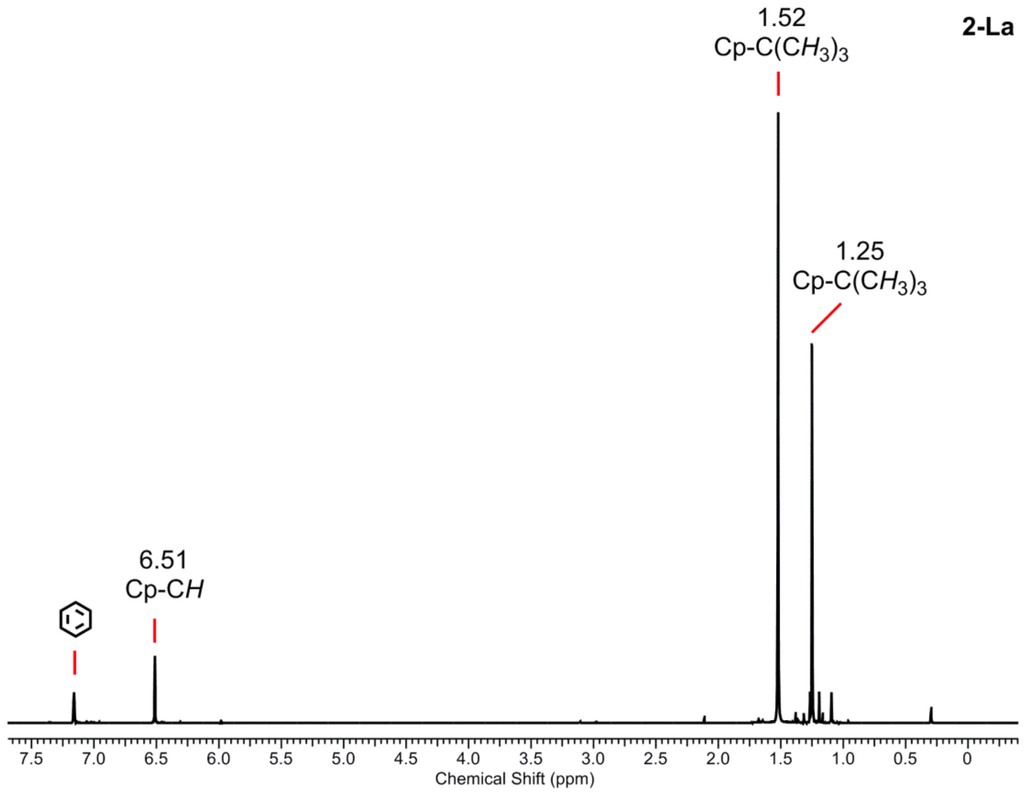

Figure S66. ^{1}H NMR spectrum of complex **2-La** in [D$_6$]benzene zoomed in the region –0.5 and 7.5 ppm. Solvent residual marked.

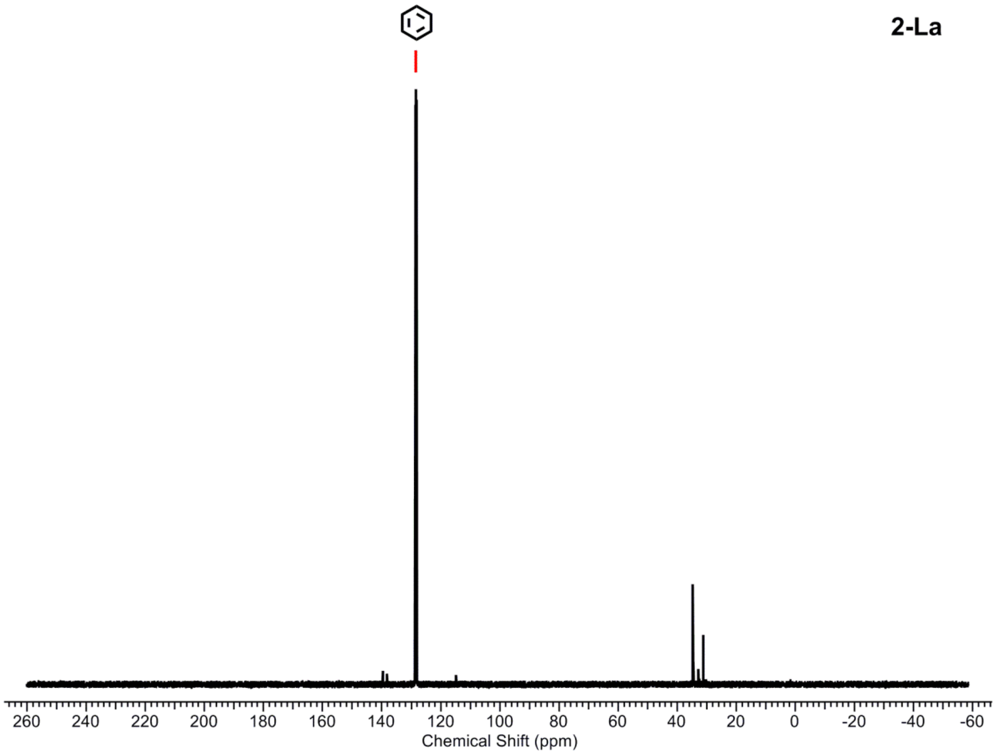

Figure S67. $^{13}C\{^{1}H\}$ NMR spectrum of complex **2-La** in [D$_6$]benzene. Solvent residual marked.

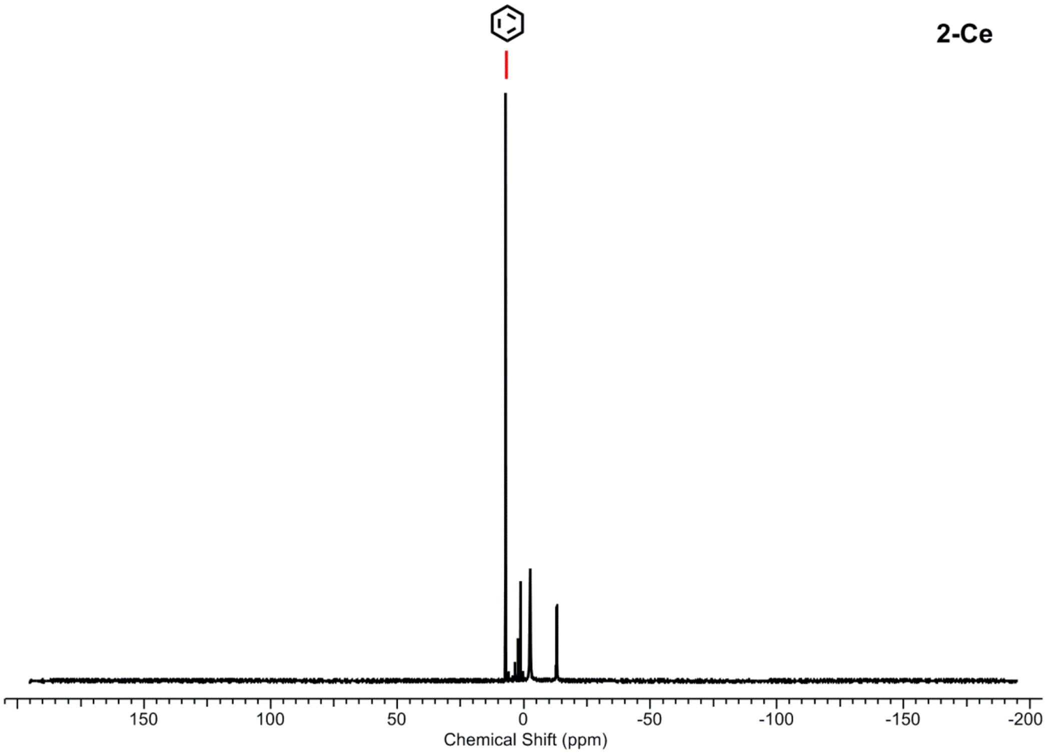

Figure S68. ^{1}H NMR spectrum of complex **2-Ce** in [D$_6$]benzene. Solvent residual marked.

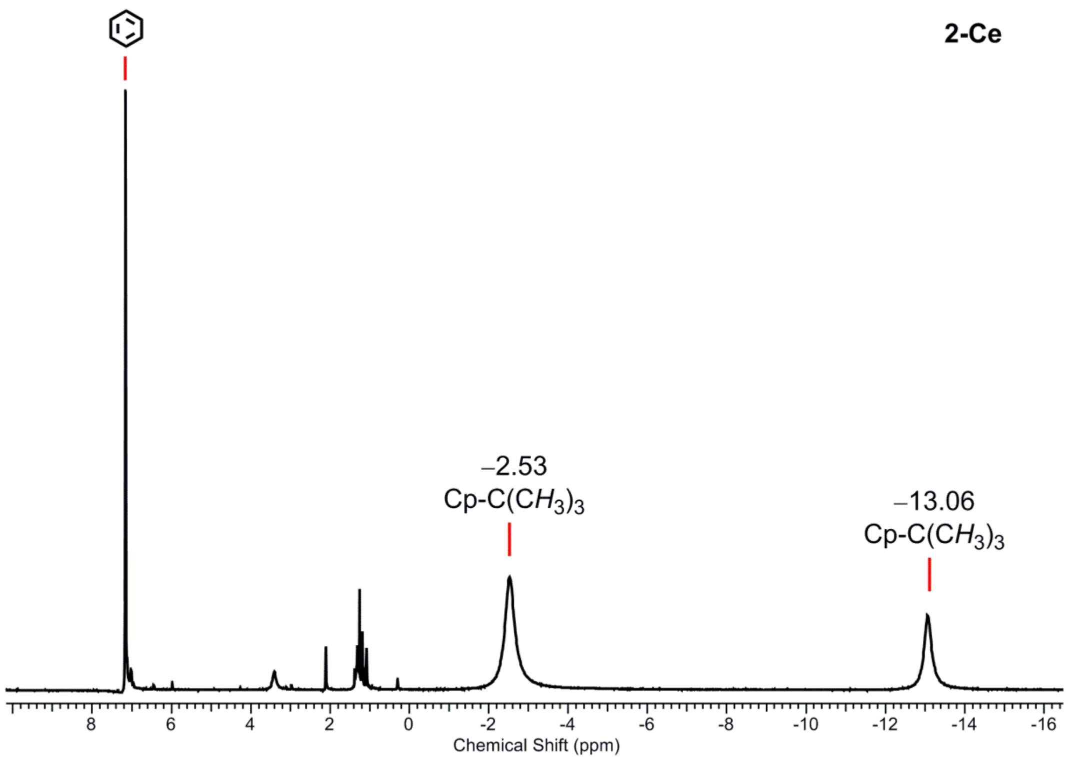

Figure S69. ^{1}H NMR spectrum of complex **2-Ce** in [D$_6$]benzene zoomed in the region –16 and 10 ppm. Solvent residual marked.

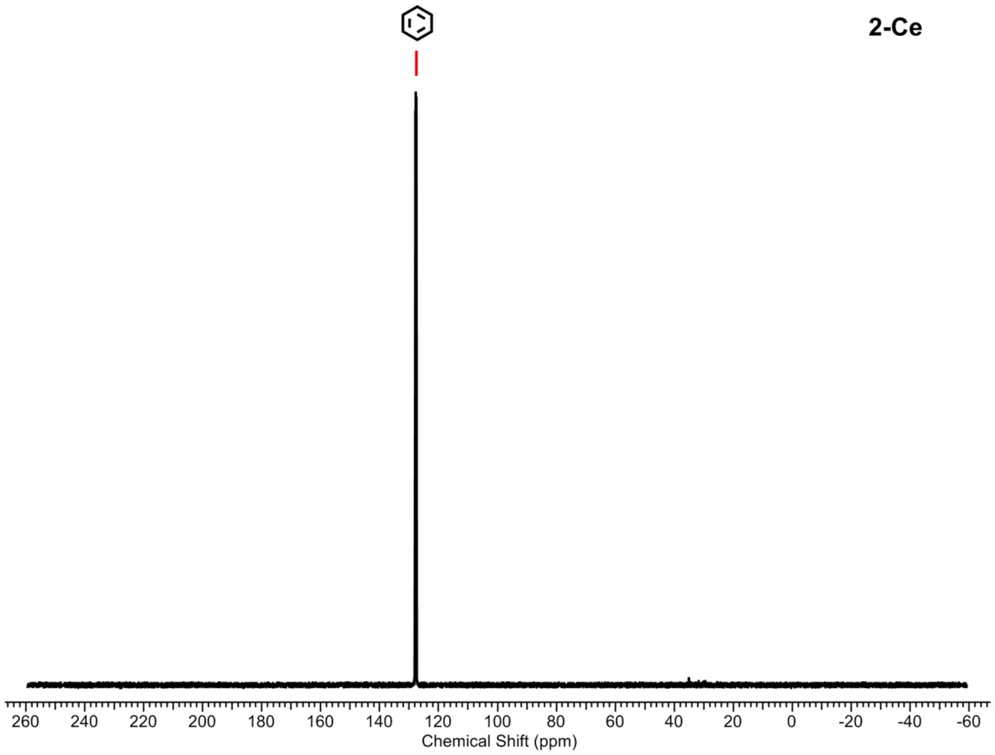

Figure S70. $^{13}C\{^1H\}$ NMR spectrum of complex **2-Ce** in [D$_6$]benzene. Solvent residual marked.

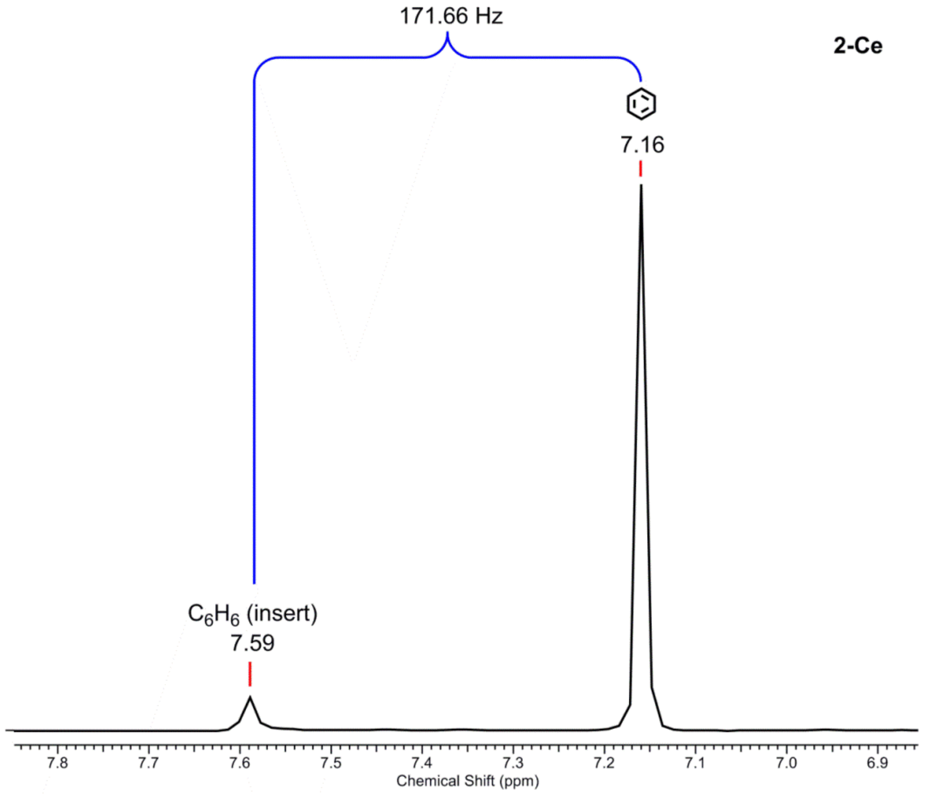

Figure S71. ^{1}H NMR spectrum of complex **2-Ce** in [D$_6$]benzene with a C_6H_6/[D$_6$]benzene insert. Solvent residual marked.

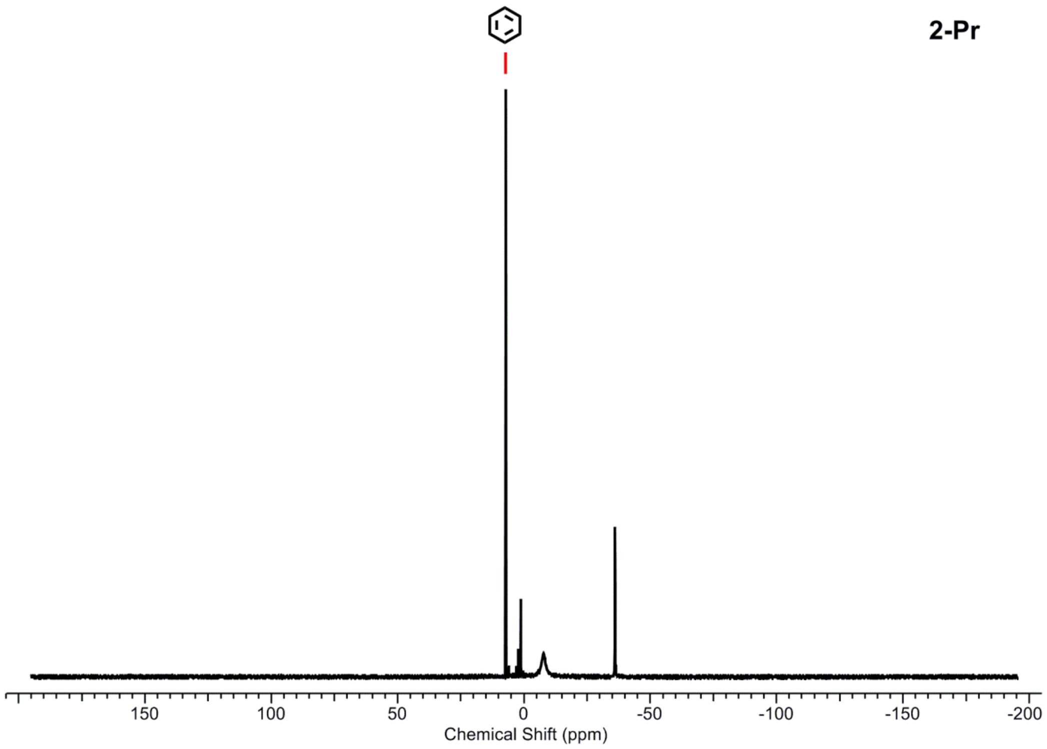

Figure S72. ^{1}H NMR spectrum of complex **2-Pr** in [D$_6$]benzene. Solvent residual marked.

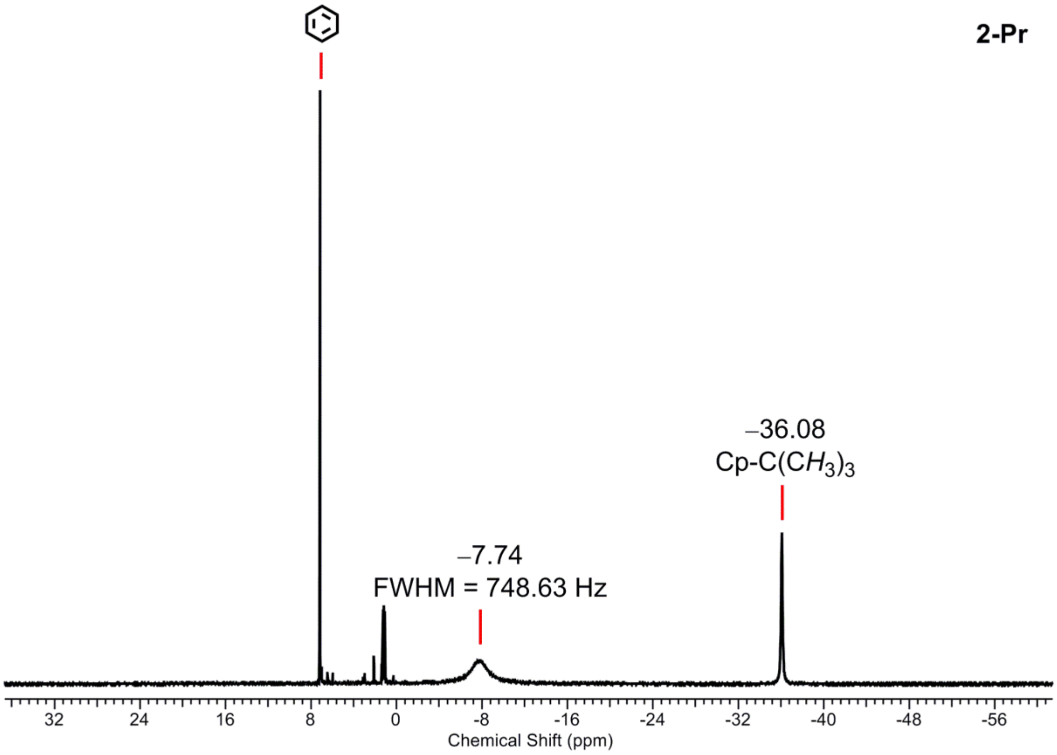

Figure S73. ^{1}H NMR spectrum of complex **2-Pr** in [D$_6$]benzene zoomed in the region –60 and 36 ppm. Solvent residual marked.

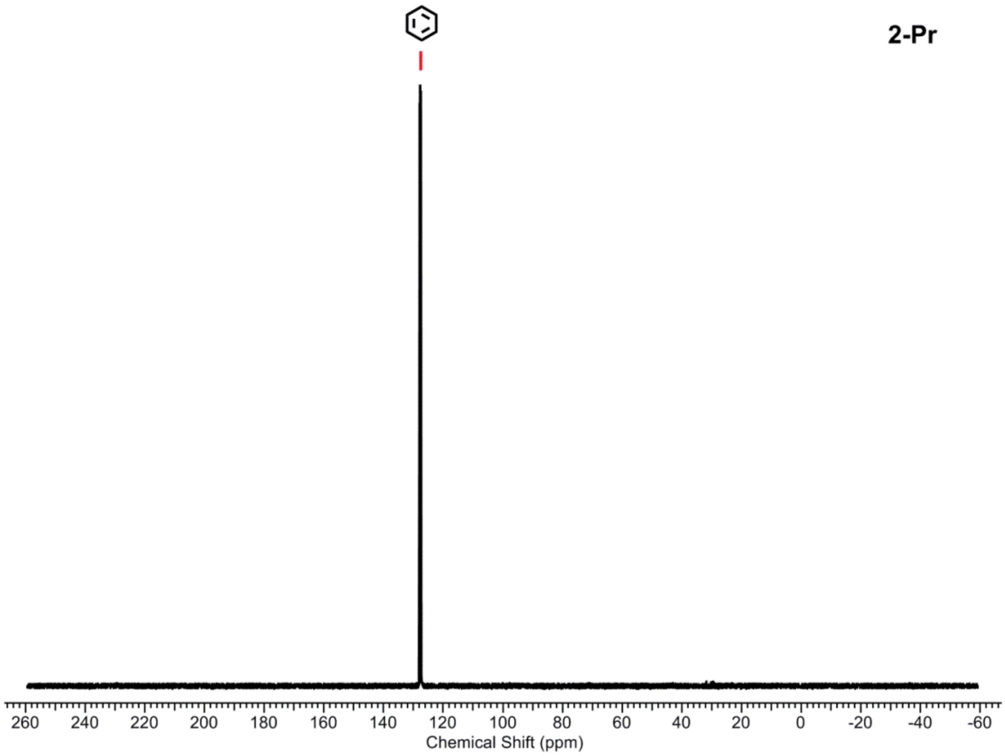

Figure S74. $^{13}C\{^{1}H\}$ NMR spectrum of complex **2-Pr** in [D$_6$]benzene. Solvent residual marked.

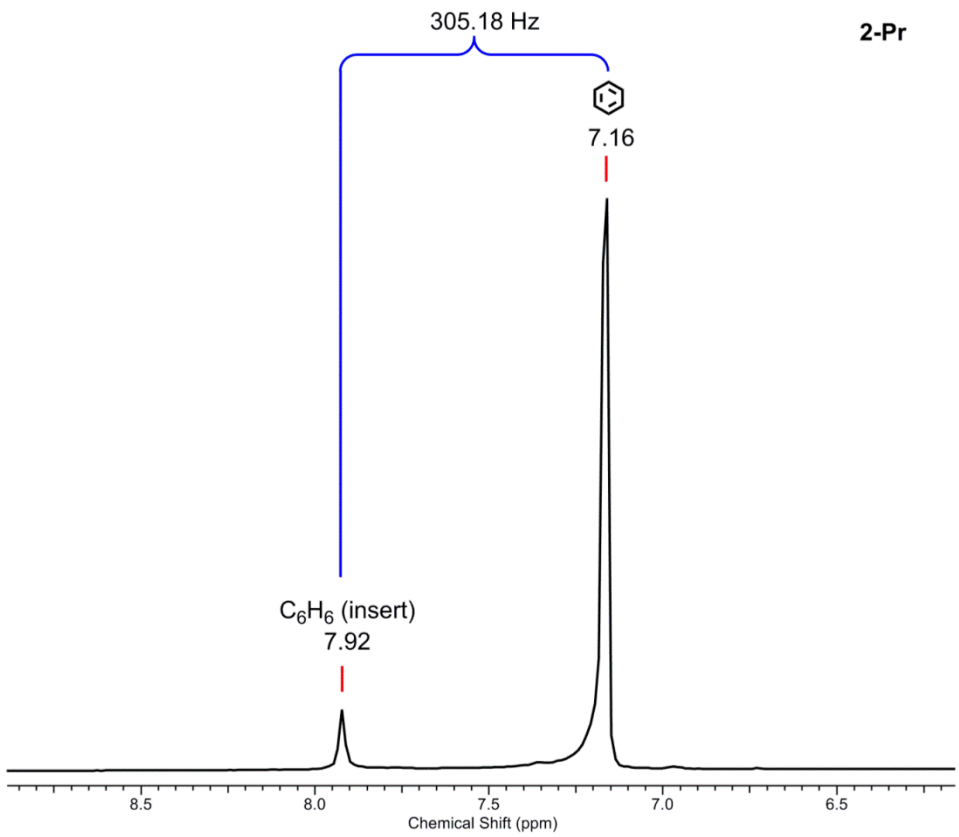

Figure S75. ^{1}H NMR spectrum of complex **2-Pr** in [D$_6$]benzene with a C_6H_6/[D$_6$]benzene insert. Solvent residual marked.

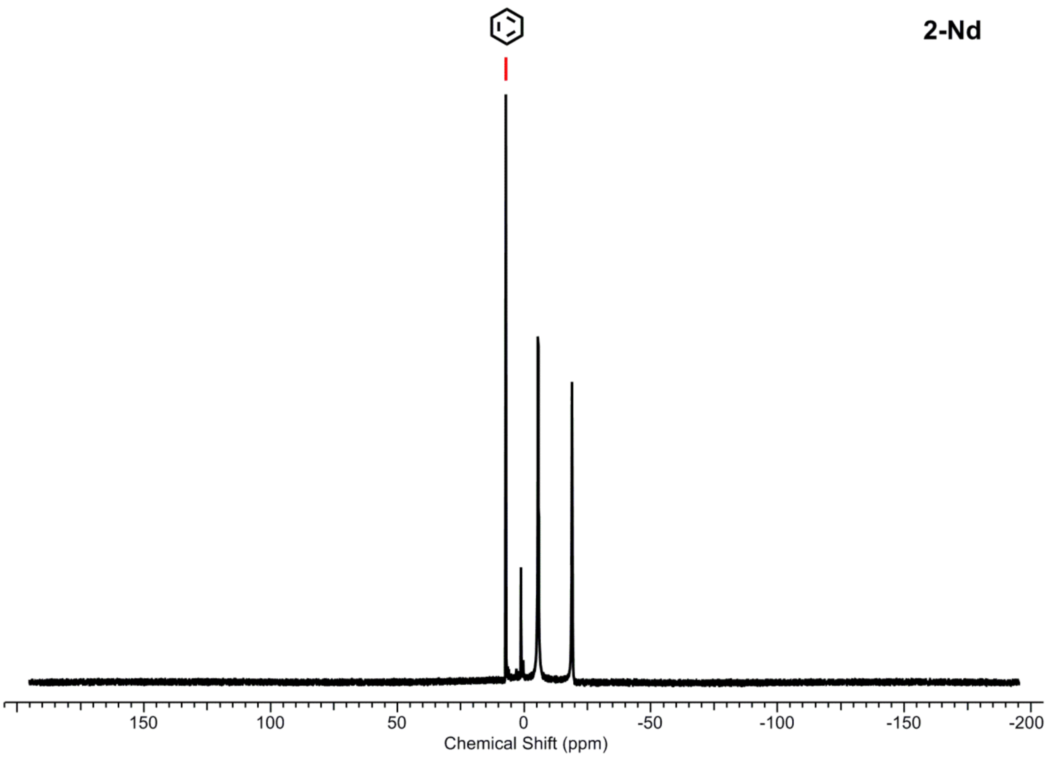

Figure S76. ^{1}H NMR spectrum of complex **2-Nd** in [D$_6$]benzene. Solvent residual marked.

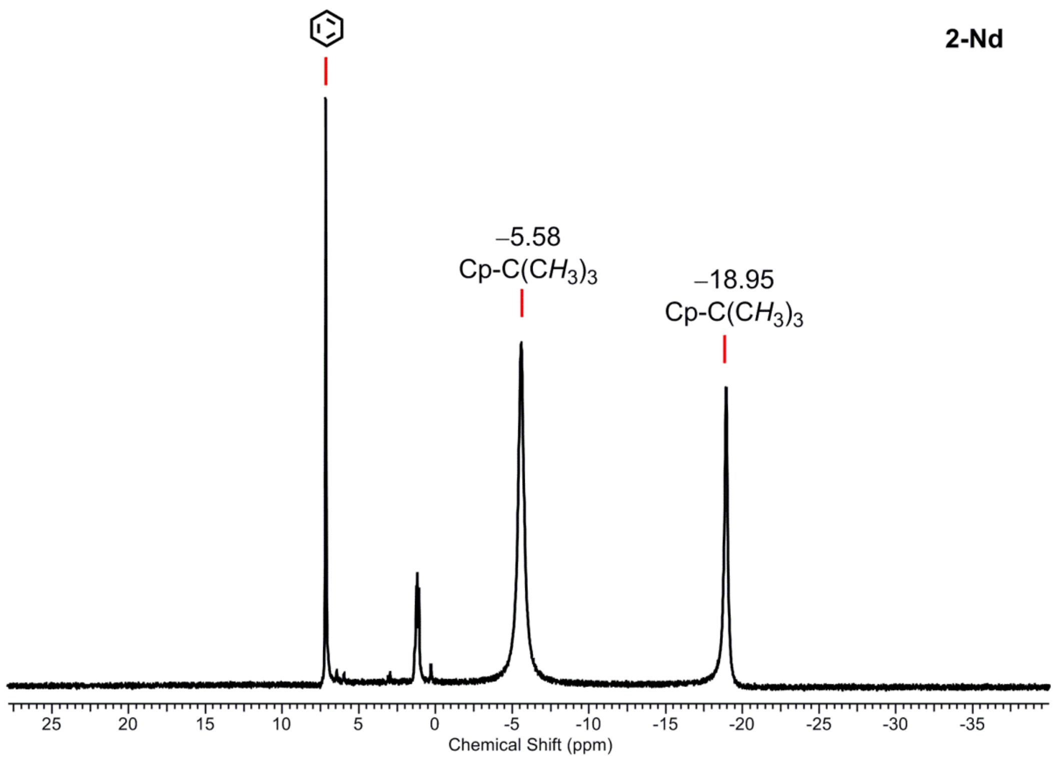

Figure S77. ^{1}H NMR spectrum of complex **2-Nd** in [D$_6$]benzene zoomed in the region –40 and 27 ppm. Solvent residual marked.

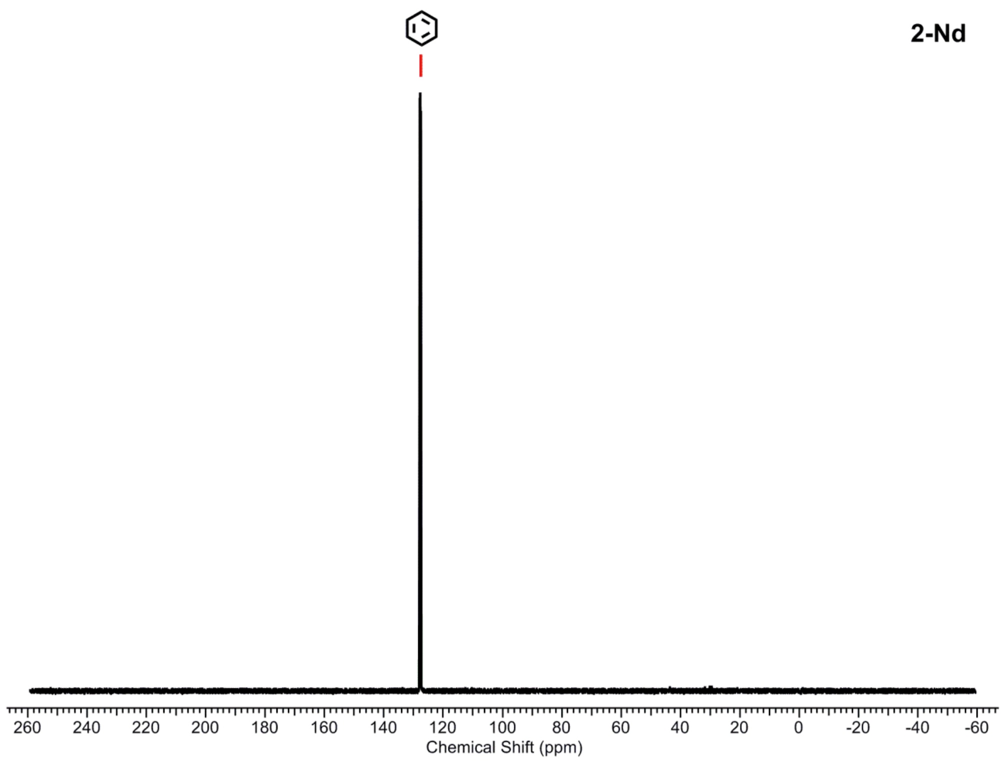

Figure S78. $^{13}C\{^{1}H\}$ NMR spectrum of complex **2-Nd** in [D$_6$]benzene. Solvent residual marked.

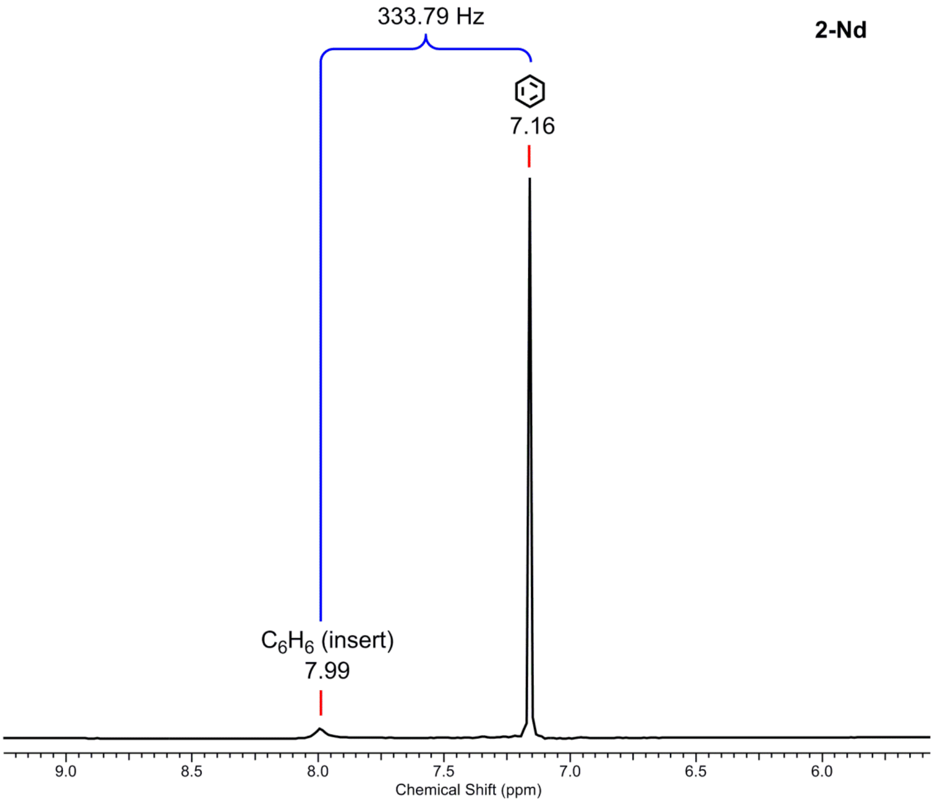

Figure S79. ^{1}H NMR spectrum of complex **2-Nd** in [D$_6$]benzene with a C_6H_6/[D$_6$]benzene insert. Solvent residual marked.

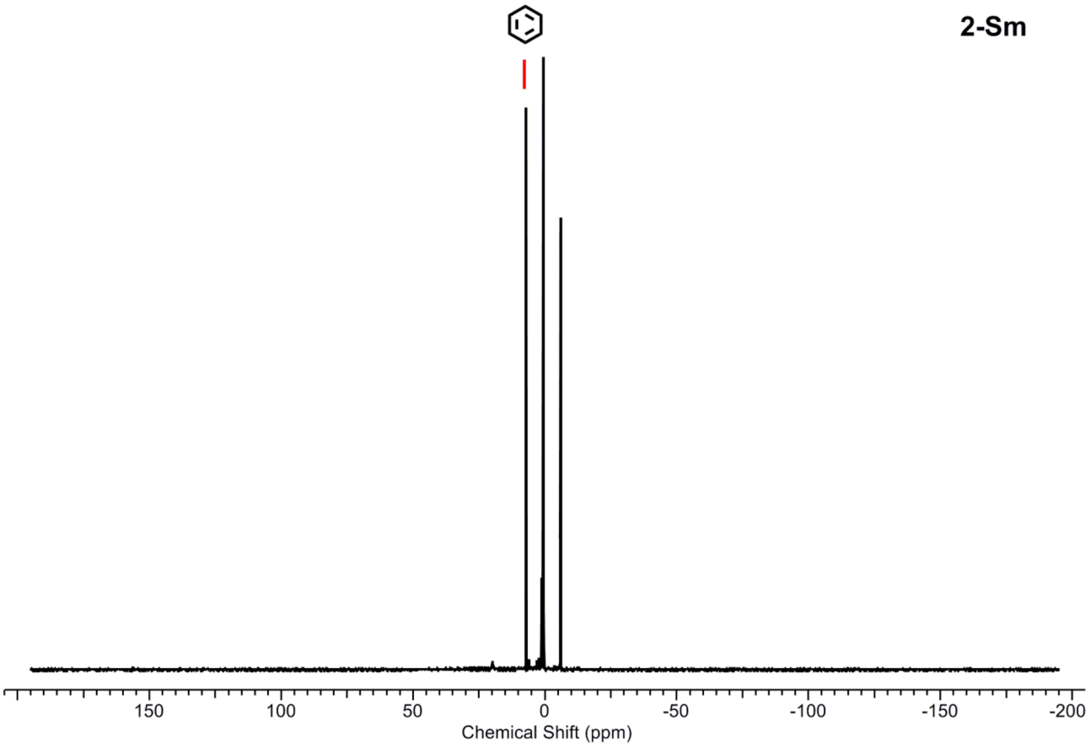

Figure S80. ^{1}H NMR spectrum of complex **2-Sm** in [D$_6$]benzene. Solvent residual marked.

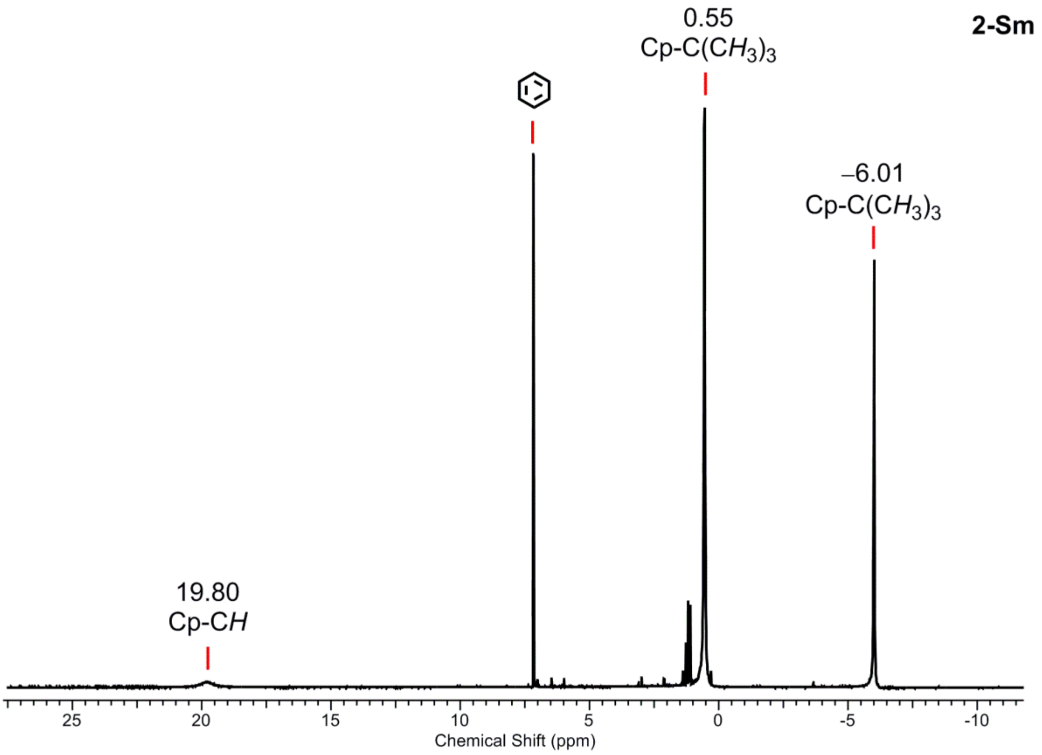

Figure S81. ^{1}H NMR spectrum of complex **2-Sm** in [D$_6$]benzene zoomed in the region –12 and 27 ppm. Solvent residual marked.

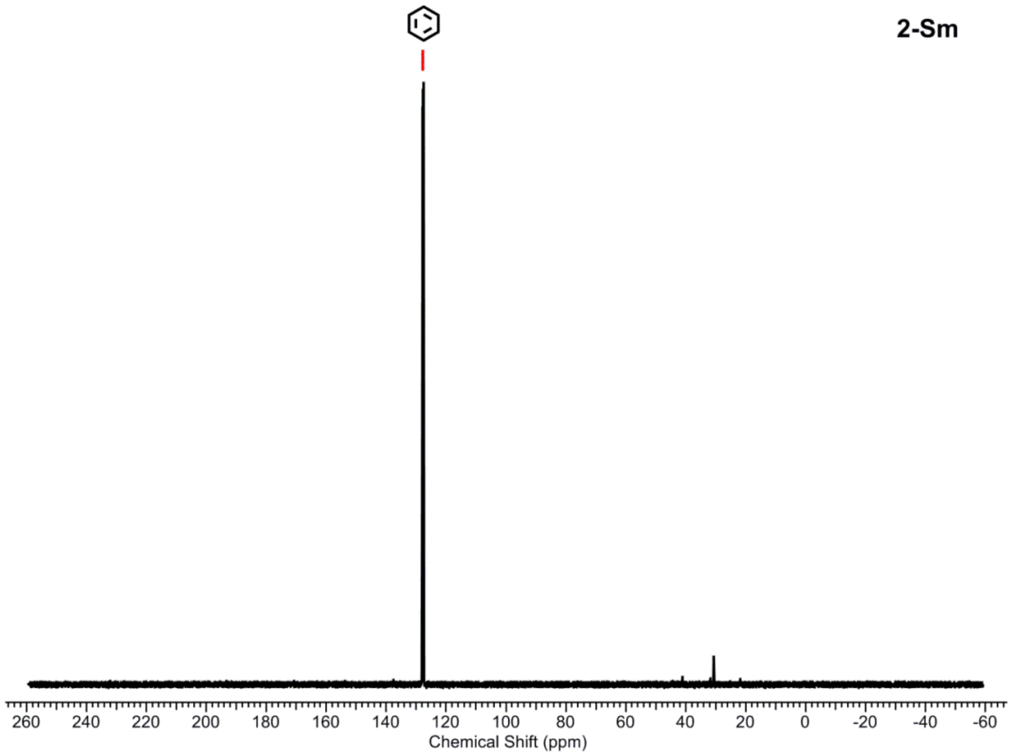

Figure S82. $^{13}C\{^{1}H\}$ NMR spectrum of complex **2-Sm** in [D$_6$]benzene. Solvent residual marked.

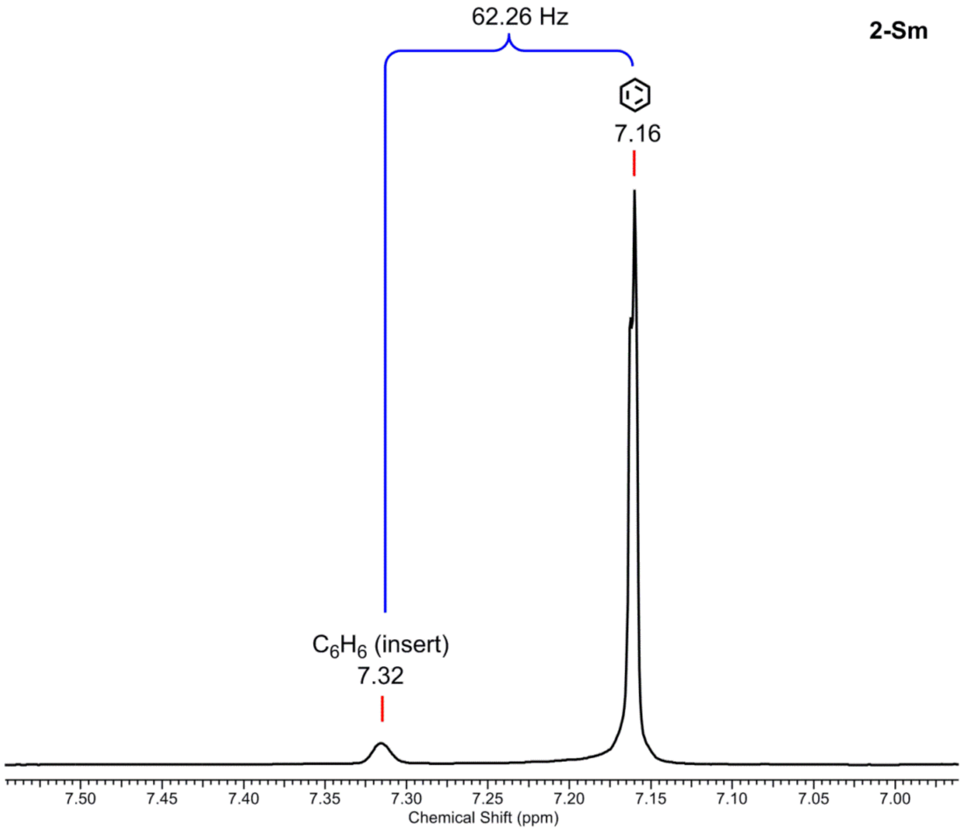

Figure S83. ^{1}H NMR spectrum of complex **2-Sm** in [D$_6$]benzene with a C_6H_6/[D$_6$]benzene insert. Solvent residual marked.

7. ATR-IR spectroscopy

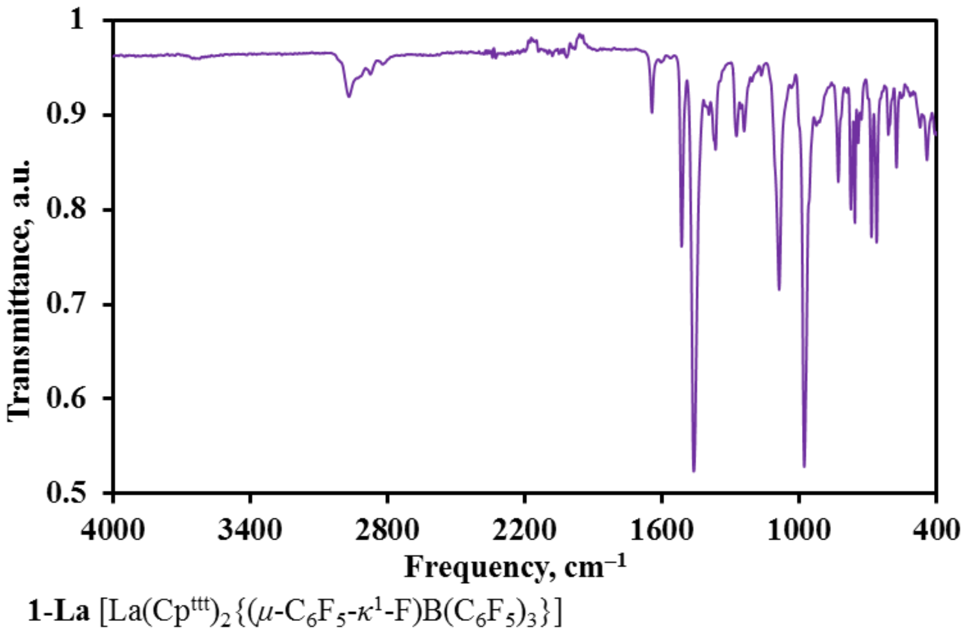

Figure S84. ATR-IR spectrum of **1-La** recorded as a microcrystalline powder.

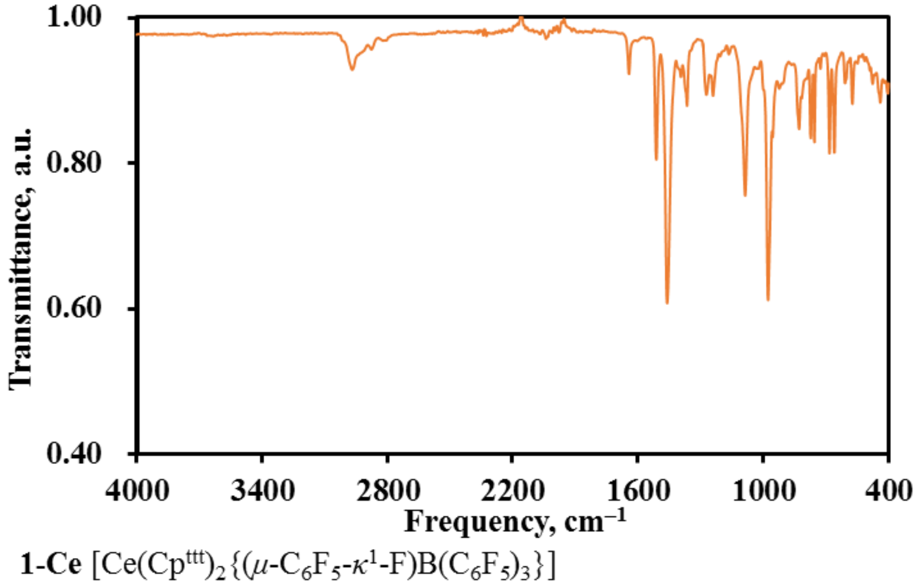

Figure S85. ATR-IR spectrum of **1-Ce** recorded as a microcrystalline powder.

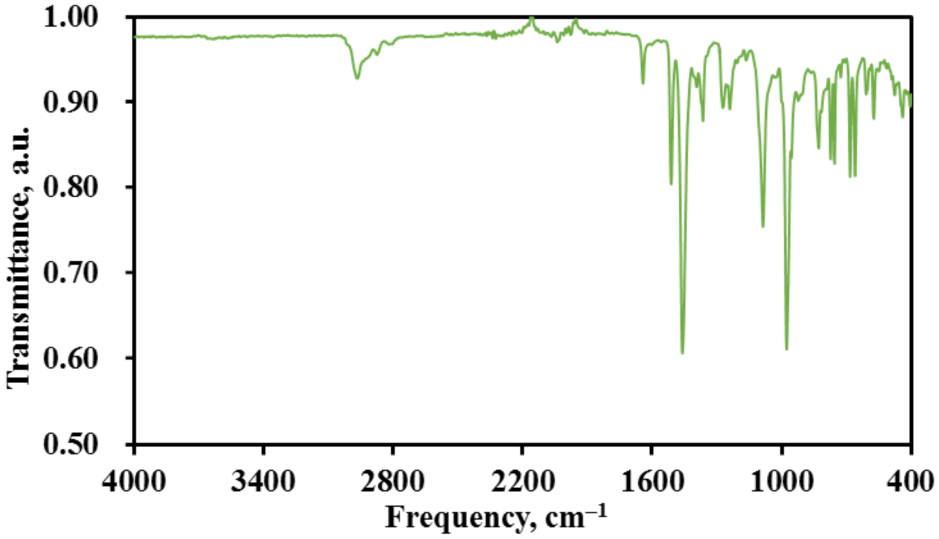

1-Pr [Pr(Cp^{ttt})$_2${(μ-C$_6$F$_5$-κ^1-F)B(C$_6$F$_5$)$_3$}]

Figure S86. ATR-IR spectrum of **1-Pr** recorded as a microcrystalline powder.

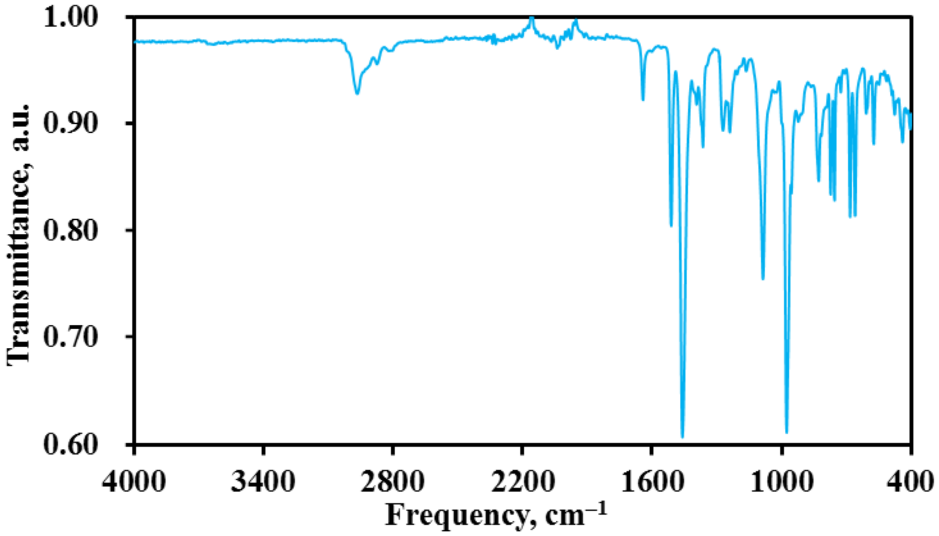

1-Nd [Nd(Cp^{ttt})$_2${(μ-C$_6$F$_5$-κ^1-F)B(C$_6$F$_5$)$_3$}]

Figure S87. ATR-IR spectrum of **1-Nd** recorded as a microcrystalline powder.

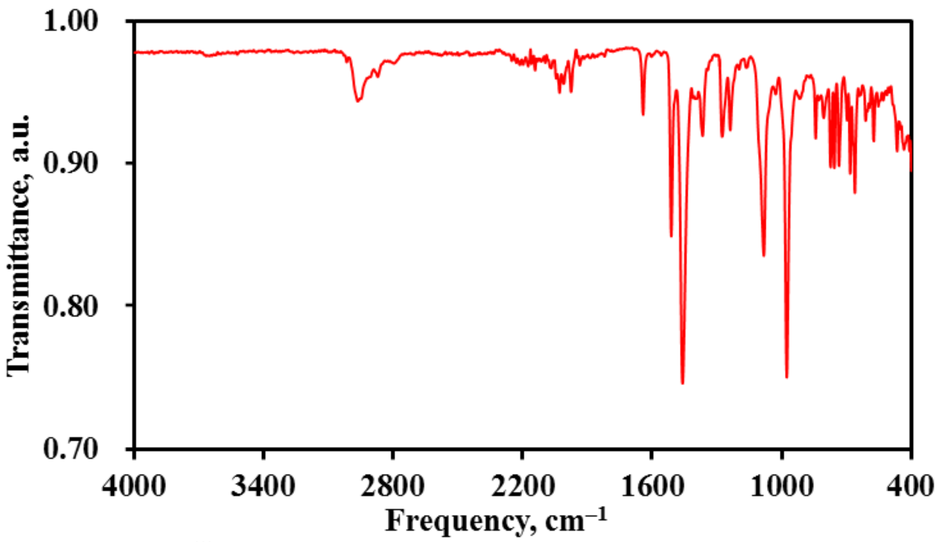

1-Sm $[Sm(Cp^{ttt})_2][B(C_6F_5)_4]$

Figure S88. ATR-IR spectrum of **1-Sm** recorded as a microcrystalline powder.

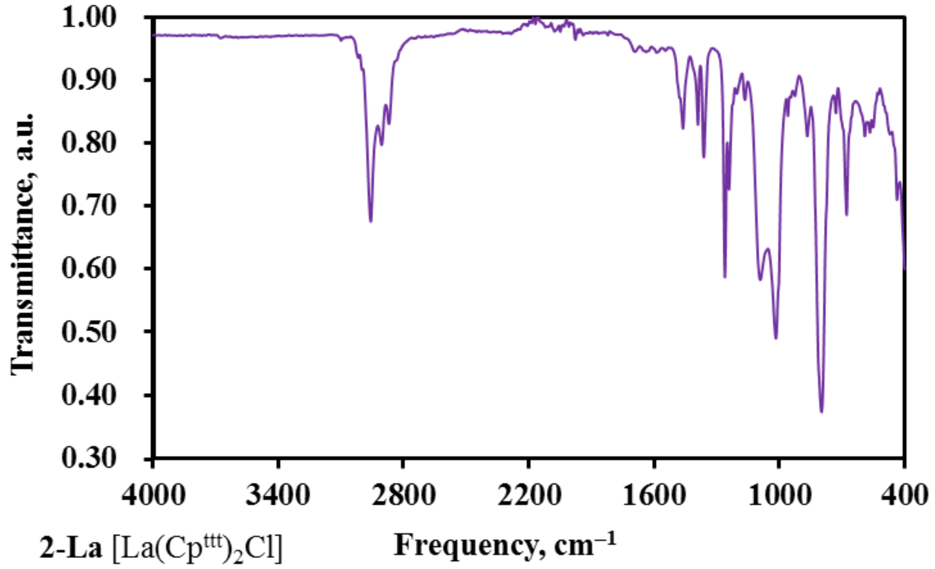

2-La $[La(Cp^{ttt})_2Cl]$

Figure S89. ATR-IR spectrum of **2-La** recorded as a microcrystalline powder.

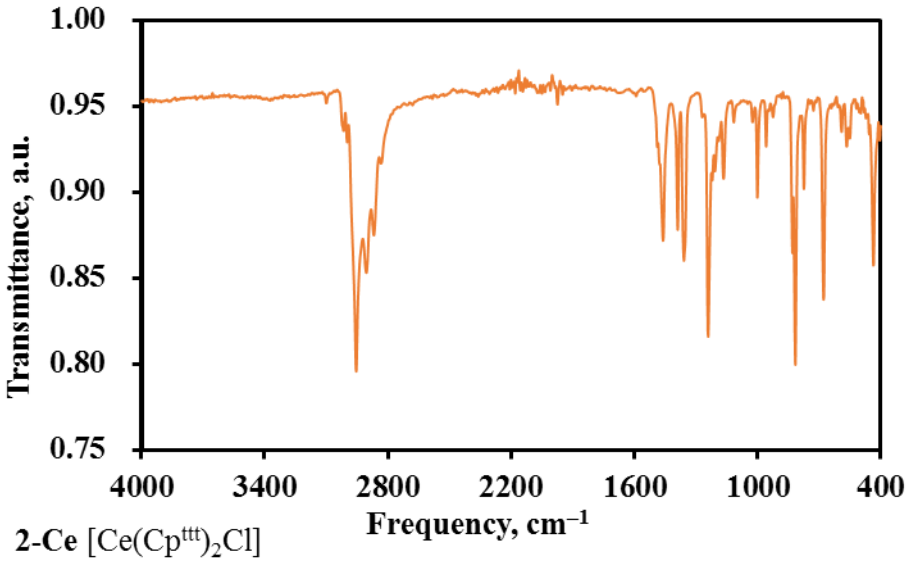

Figure S90. ATR-IR spectrum of **2-Ce** recorded as a microcrystalline powder.

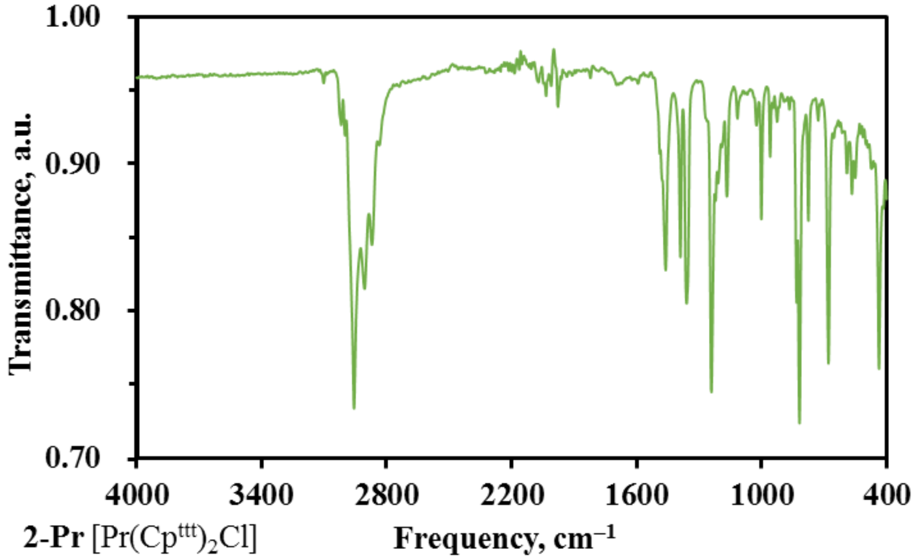

Figure S91. ATR-IR spectrum of **2-Pr** recorded as a microcrystalline powder.

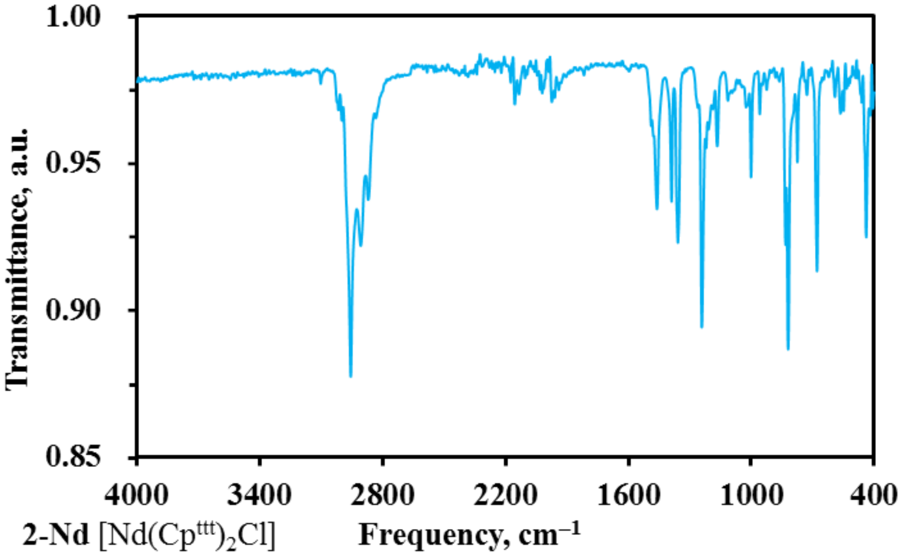

Figure S92. ATR-IR spectrum of **2-Nd** recorded as a microcrystalline powder.

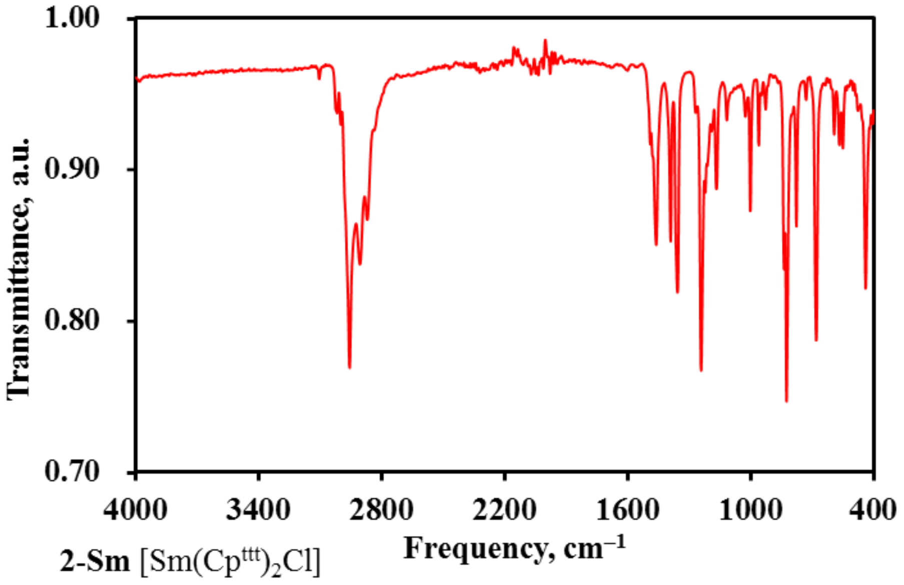

Figure S93. ATR-IR spectrum of **2-Sm** recorded as a microcrystalline powder.

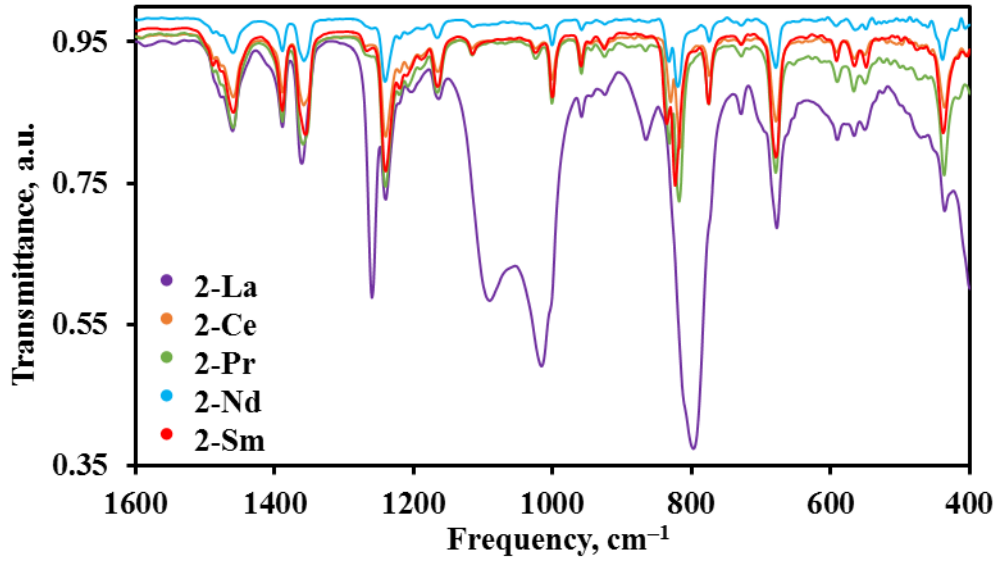

Figure S94. ATR-IR spectra of **2-La**, **2-Ce**, **2-Pr**, **2-Nd**, **2-Sm**, in the region 1600–400 cm^{-1} intended to show the similarities between all spectra.

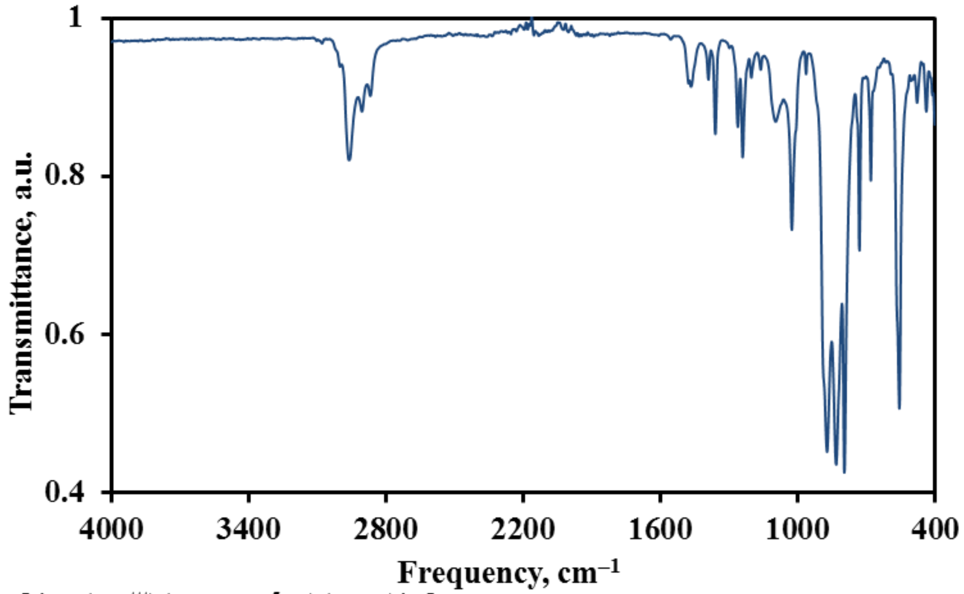

3 [{Eu(Cp^{ttt})(μ-PF_6-κ^5-F)(THF)$\}_2$]

Figure S95. ATR-IR spectrum of **3** recorded as a microcrystalline powder.

8. UV-vis-NIR spectroscopy

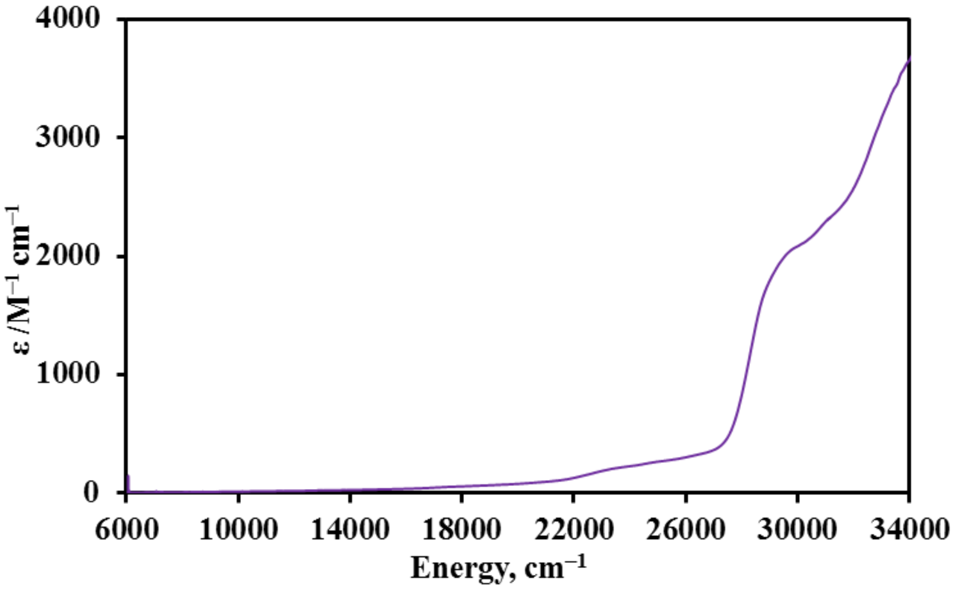

1-La [La(Cp^{ttt})$_2${(μ-C$_6$F$_5$-κ^1-F)B(C$_6$F$_5$)$_3$}]

Figure S96. UV-vis-NIR spectrum of **1-La** between 34000–6000 cm^{-1} (295–1650 nm) recorded as a 1.136 mM solution in DCM.

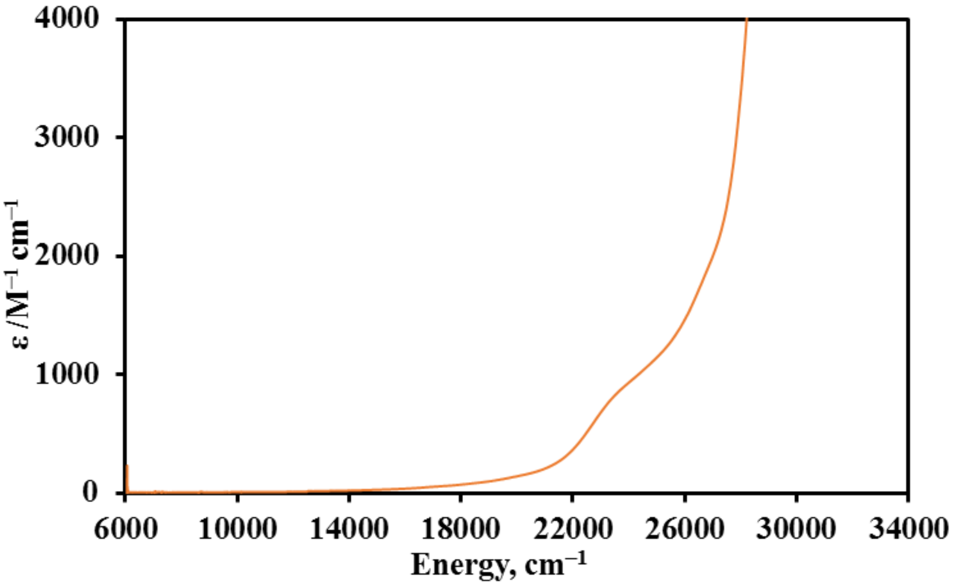

1-Ce [Ce(Cp^{ttt})$_2${(μ-C$_6$F$_5$-κ^1-F)B(C$_6$F$_5$)$_3$}]

Figure S97. UV-vis-NIR spectrum of **1-Ce** between 34000–6000 cm^{-1} (295–1650 nm) recorded as a 1.032 mM solution in DCM.

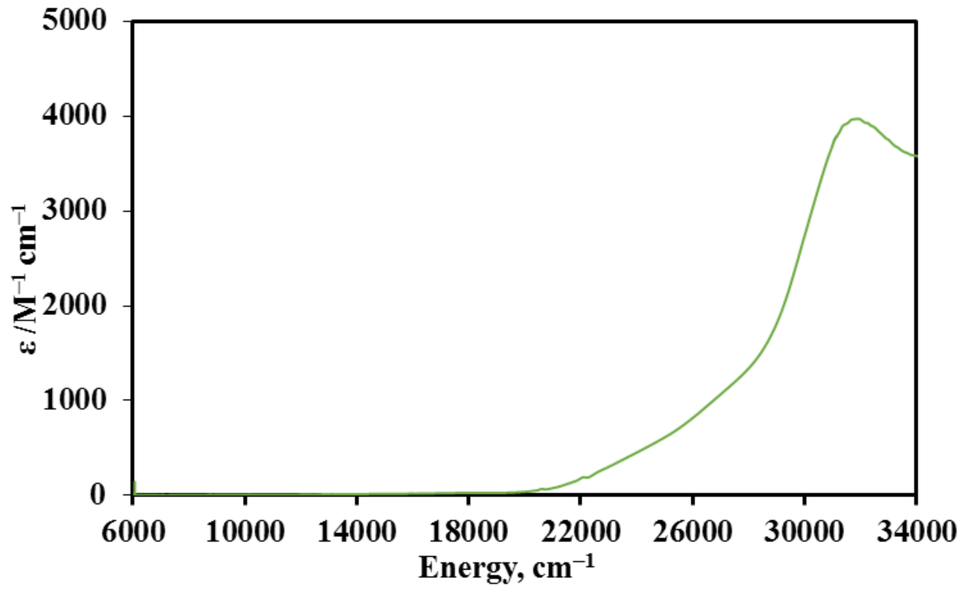

1-Pr [Pr(Cp^{ttt})$_2${(μ-C_6F_5-κ^1-F)B(C_6F_5)$_3$}]

Figure S98. UV-vis-NIR spectrum of **1-Pr** between 34000–6000 cm^{-1} (295–1650 nm) recorded as a 0.974 mM solution in DCM.

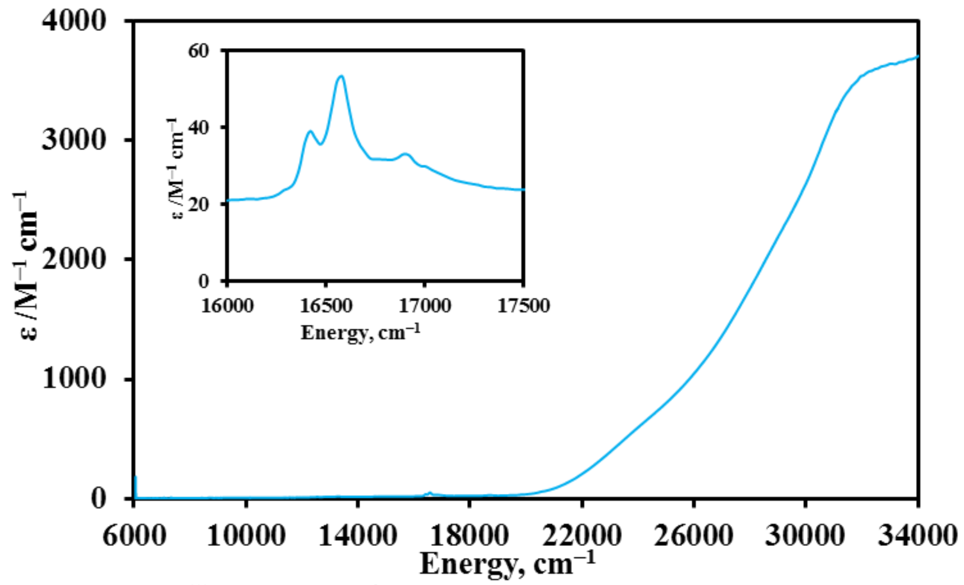

1-Nd [Nd(Cp^{ttt})$_2${(μ-C_6F_5-κ^1-F)B(C_6F_5)$_3$}]

Figure S99. UV-vis-NIR spectrum of **1-Nd** between 34000–6000 cm^{-1} (295–1650 nm) recorded as a 1.024 mM solution in DCM. Inset shows the region 17500–16000 cm^{-1} (570–625 nm).

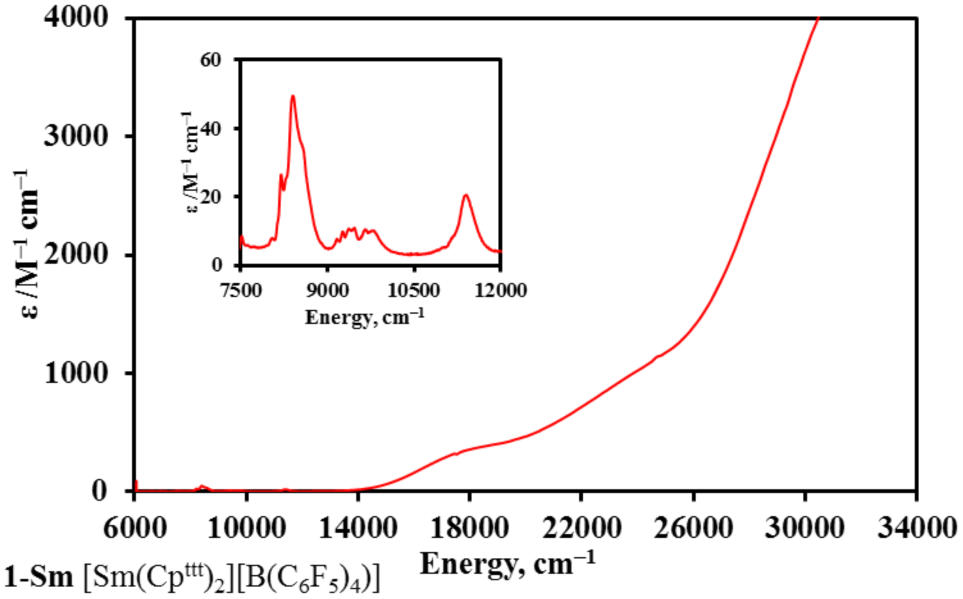

Figure S100. UV-vis-NIR spectrum of **1-Sm** between 34000–6000 cm^{-1} (295–1650 nm) recorded as a 0.949 mM solution in DCM. Inset shows the region 12000–7500 cm^{-1} (830–1330 nm).

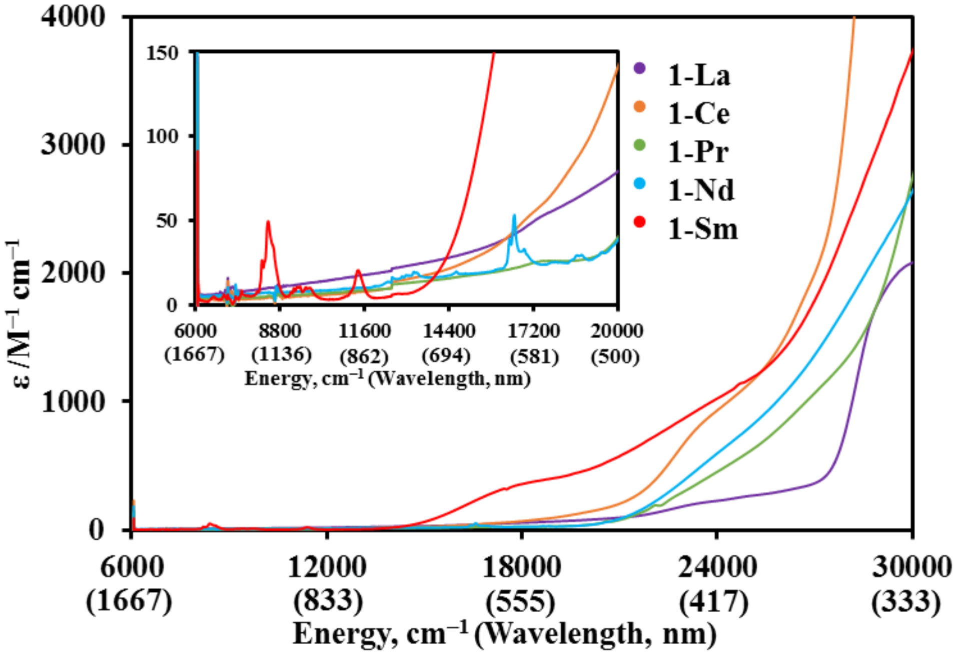

Figure S101. UV-vis-NIR spectrum of **1-La**, **1-Ce**, **1-Pr**, **1-Nd**, **1-Sm** in the region 6000–30000 cm^{-1} (inset 6000–20000 showing main region) recorded in DCM at room temperature, concentrations listed above individually.

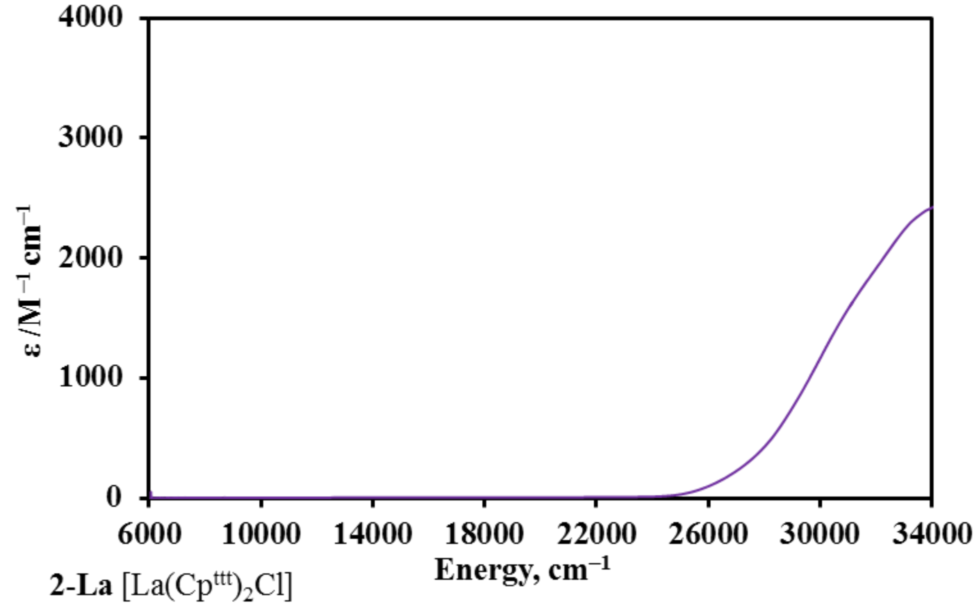

Figure S102. UV-vis-NIR spectrum of **2-La** between 34000–6000 cm^{-1} (295–1650 nm) recorded as a 1.134 mM solution in DCM.

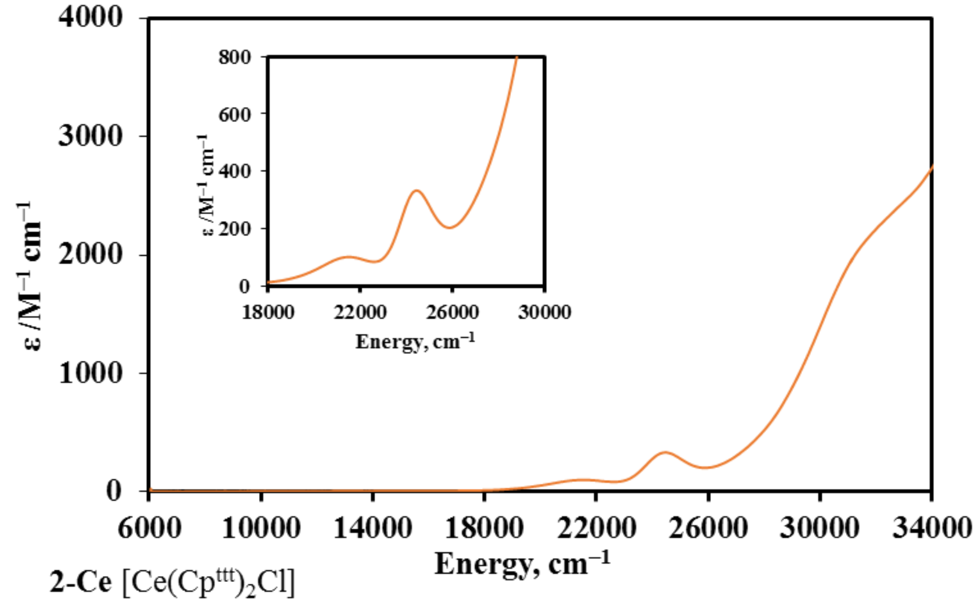

Figure S103. UV-vis-NIR spectrum of **2-Ce** between 34000–6000 cm^{-1} (295–1650 nm) recorded as a 1.005 mM solution in DCM. Inset shows the region 30000–18000 cm^{-1} (330–555 nm).

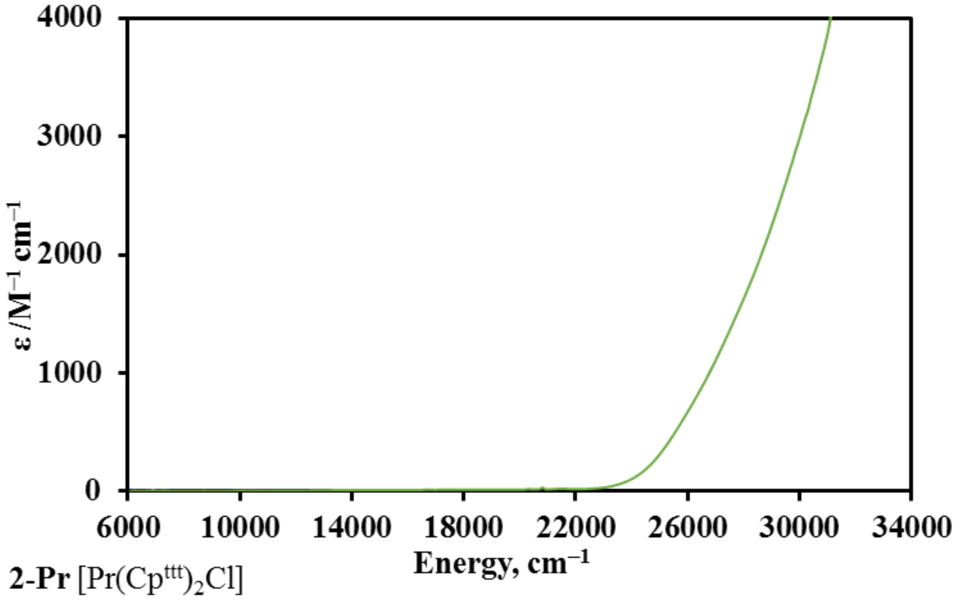

Figure S104. UV-vis-NIR spectrum of **2-Pr** between 34000–6000 cm^{-1} (295–1650 nm) recorded as a 1.125 mM solution in DCM.

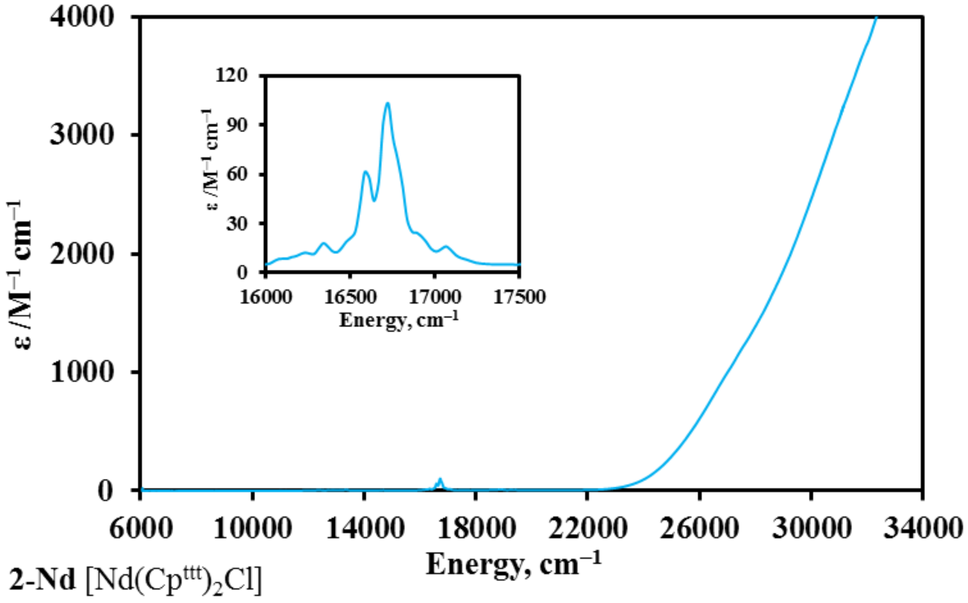

Figure S105. UV-vis-NIR spectrum of **2-Nd** between 34000–6000 cm^{-1} (295–1650 nm) recorded as a 1.062 mM solution in DCM. Inset shows the region 17500–16000 cm^{-1} (570–625 nm).

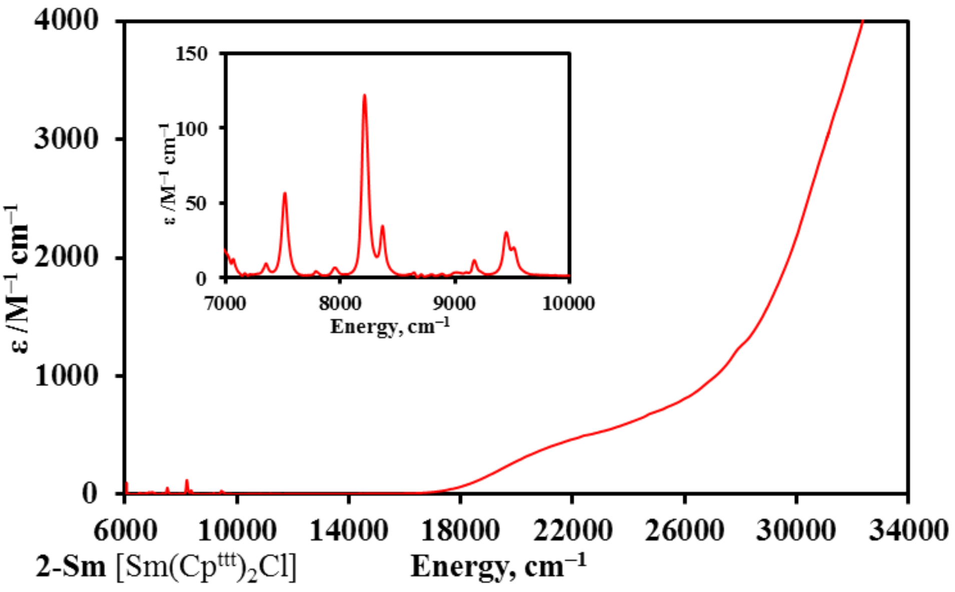

Figure S106. UV-vis-NIR spectrum of **2-Sm** between 34000–6000 cm^{-1} (295–1650 nm) recorded as a 1.103 mM solution in DCM. Inset shows the region 10000–7000 cm^{-1} (1000–1430 nm).

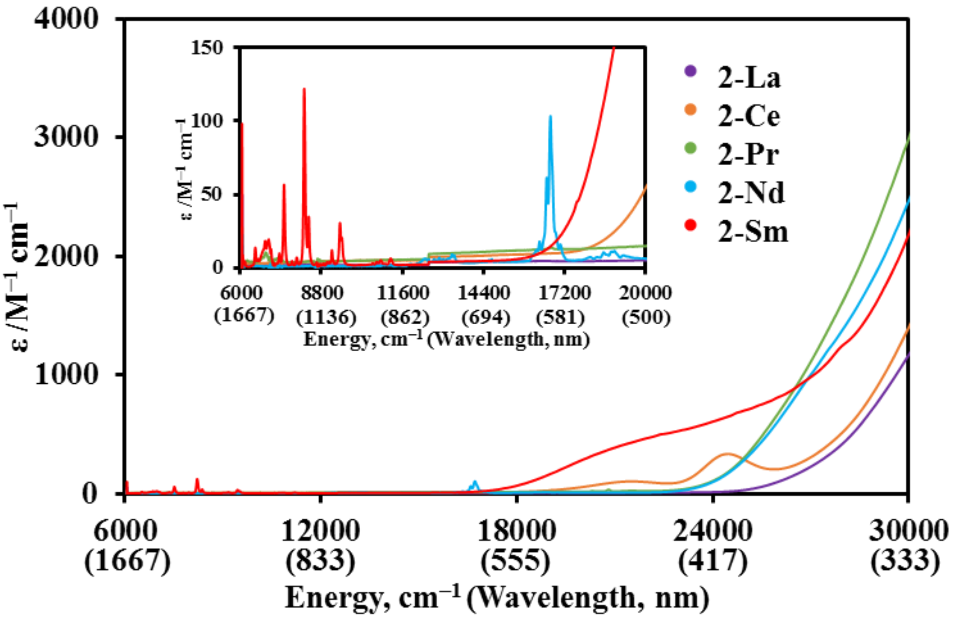

Figure S107. UV-vis-NIR spectrum of **2-La**, **2-Ce**, **2-Pr**, **2-Nd**, **2-Sm**, in the region 6000–30000 cm^{-1} (inset 6000–20000 showing main region) recorded in DCM at room temperature, concentrations listed above individually.

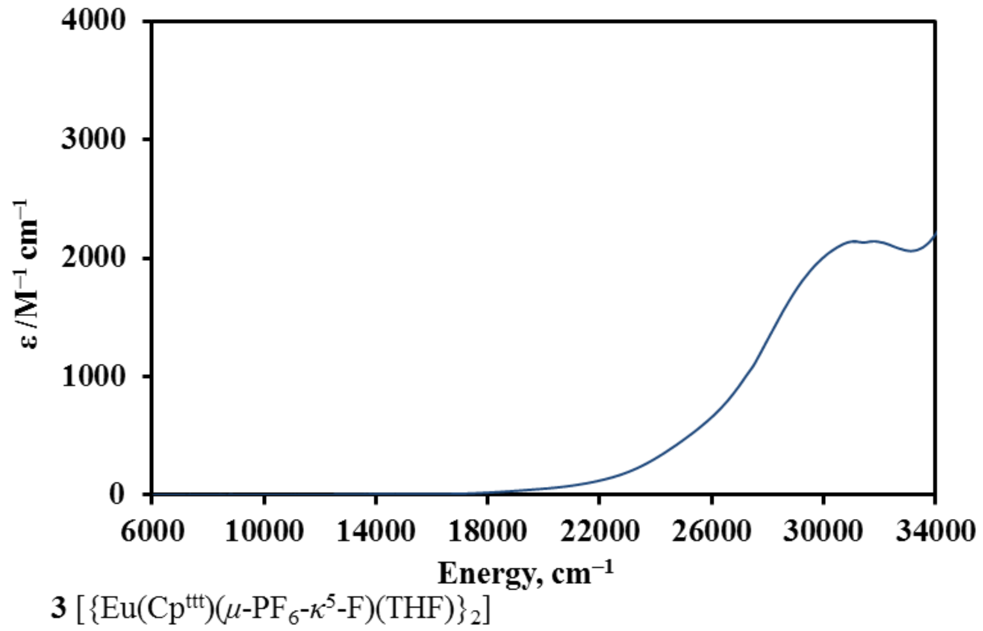

Figure S108. UV-vis-NIR spectrum of **3** between 34000–6000 cm^{-1} (295–1650 nm) recorded as a 1.013 mM solution in toluene.

9. Magnetic measurements

Magnetic measurements were performed using a Quantum Design MPMS-XL7 superconducting quantum interference device (SQUID) magnetometer. Crystalline samples with mass ranging between 15 and 40 mg were crushed with a mortar and pestle under an inert atmosphere, and then loaded into a borosilicate glass NMR tube along with *ca.* 5 - 20 mg powdered eicosane, which was then evacuated and flame-sealed to a length of *ca.* 5 cm. The eicosane was melted by heating the tube gently with a low-power heat gun in order to immobilize the crystallites. The NMR tube was then mounted in the centre of a drinking straw using friction by wrapping it with Kapton tape, and the straw was then fixed to the end of the sample rod. *N.B.* **1-Ln** samples recrystallized from DCM/hexane are temperature sensitive and therefore the samples were maintained below 250 K during measurement. The measurements were corrected for the diamagnetism of the straw, borosilicate tube and eicosane using calibrated blanks, and the intrinsic diamagnetism of the sample using Pascals constants.[7]

Table S6. Room temperature $\chi_M T$ products (cm^3 mol^{-1} K) for **1-Ln** and **2-Ln**. Evans method measured on an NMR spectrometer operating at 298 K, SQUID values measured in a 0.5 T field at 250 and 300 K for **1-Ln** and **2-Ln**, respectively.

Method	1-Ce	2-Ce	1-Pr	2-Pr	1-Nd	2-Nd	1-Sm	2-Sm
Evans	0.56	0.68	0.91	1.40	1.46	1.58	0.45	0.36
SQUID	0.79	0.76	1.80	1.35	1.80	1.57	0.46	0.29
CASSCF	0.66	0.70	1.37	1.40	1.54	1.55	0.25	0.25
Free Ion	0.81		1.60		1.69		0.09	

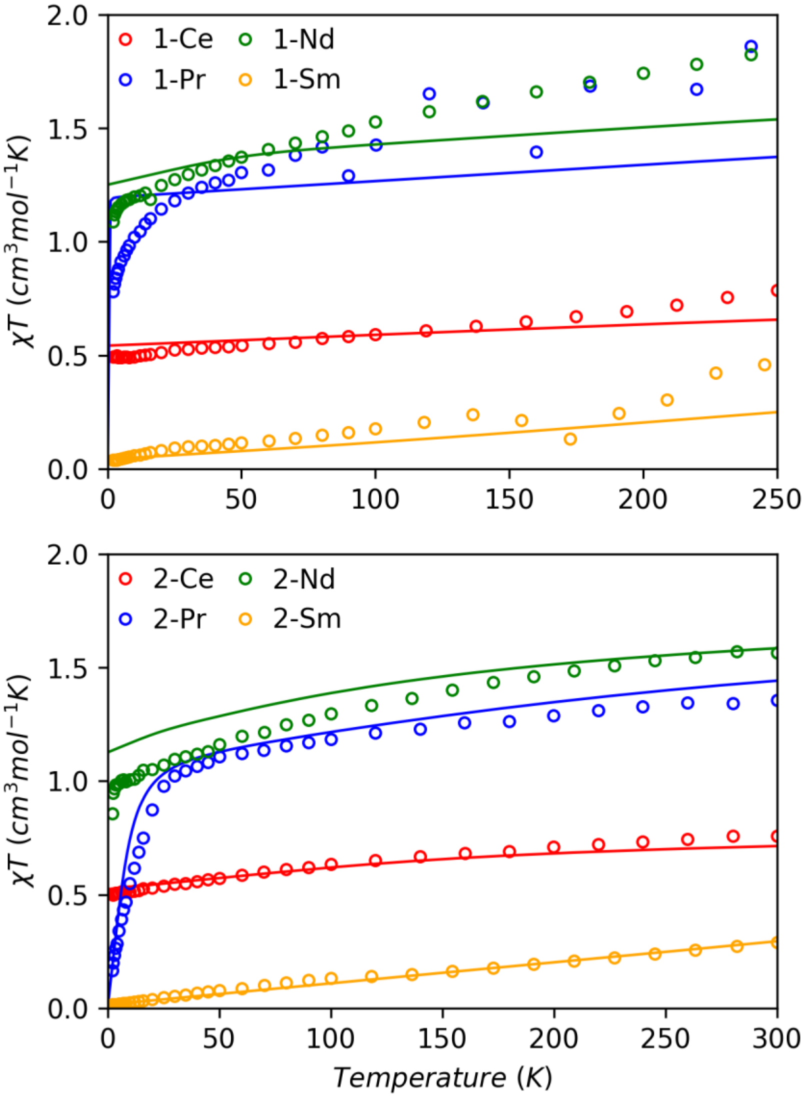

Figure S109. Temperature dependence of the molar magnetic susceptibility $\chi_M T$ products measured under a 0.1 T DC field for a) **1-Ln** and b) **2-Ln** complexes. Circles and solid lines are the experimental and CASSCF-SO calculated values, respectively.

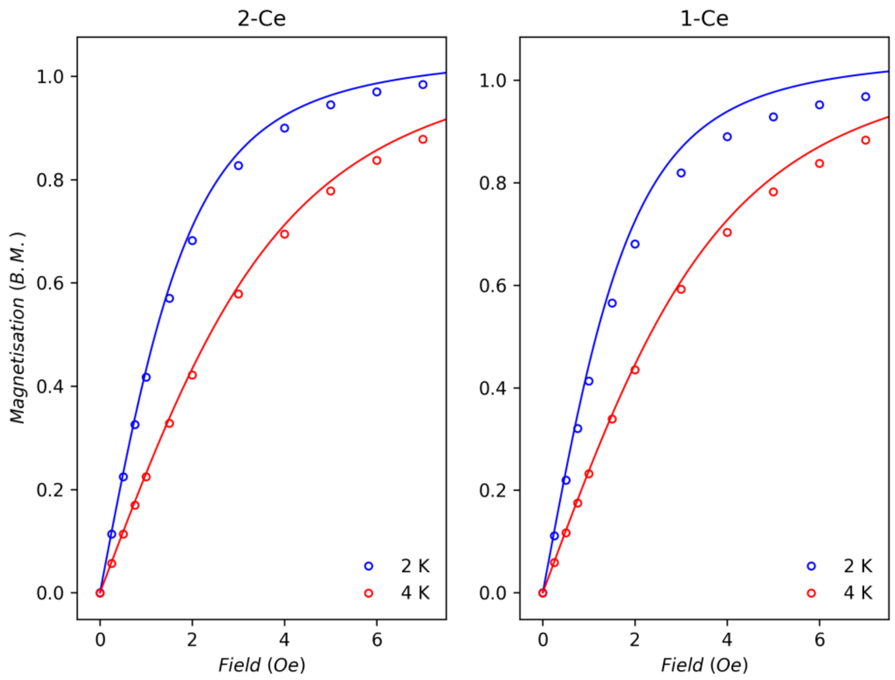

Figure S110. Field dependence of magnetization at 2 and 4 K for **2-Ce** (left) and **1-Ce** (right). Circles and solid lines are the experimental and CASSCF-SO calculated values, respectively.

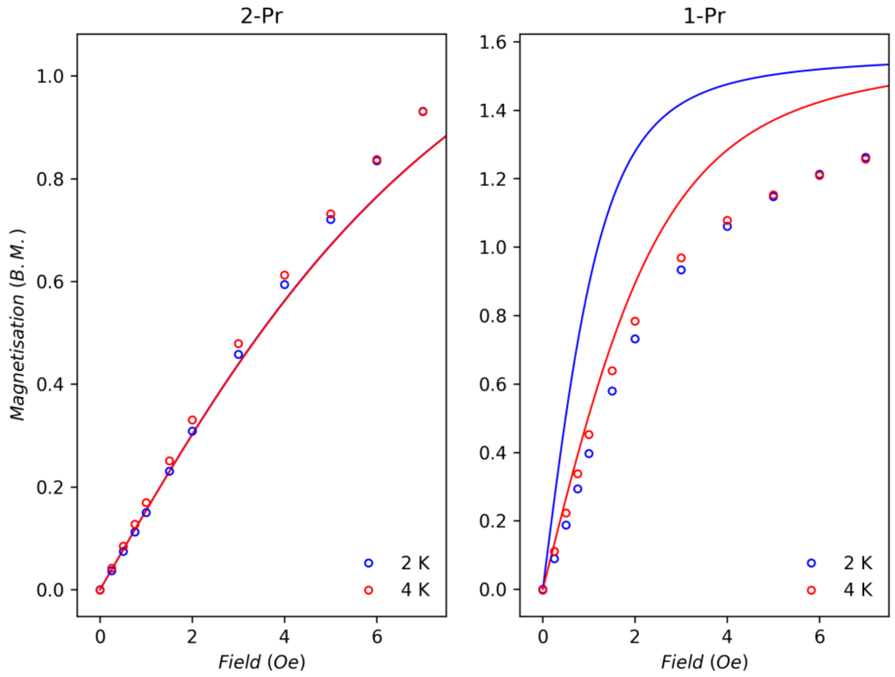

Figure S111. Field dependence of magnetization at 2 and 4 K for **2-Pr** (left) and **1-Pr** (right). Circles and solid lines are the experimental and CASSCF-SO calculated values, respectively.

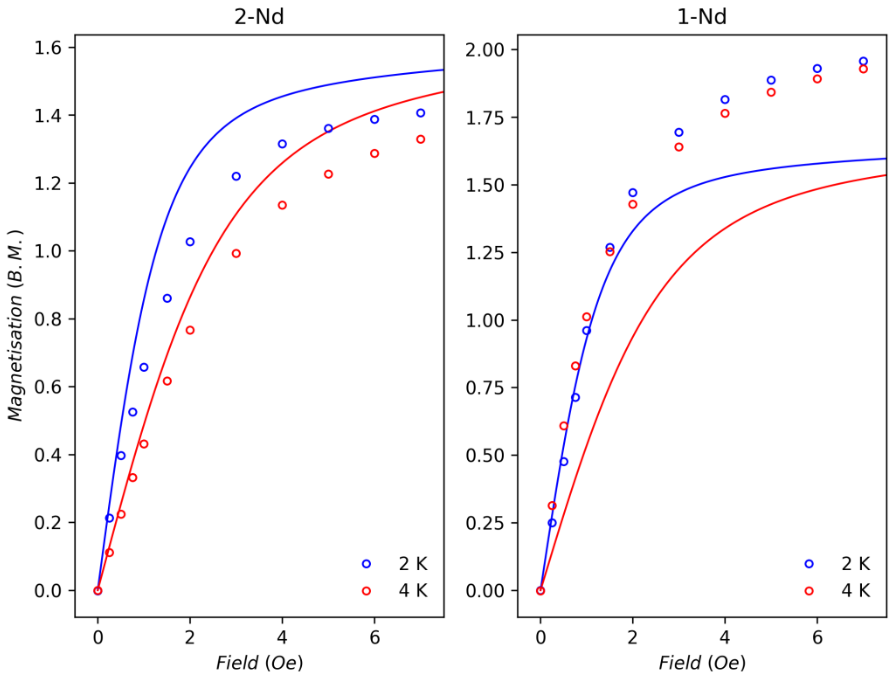

Figure S112. Field dependence of magnetization at 2 and 4 K for **2-Nd** (left) and **1-Nd** (right). Circles and solid lines are the experimental and CASSCF-SO calculated values, respectively.

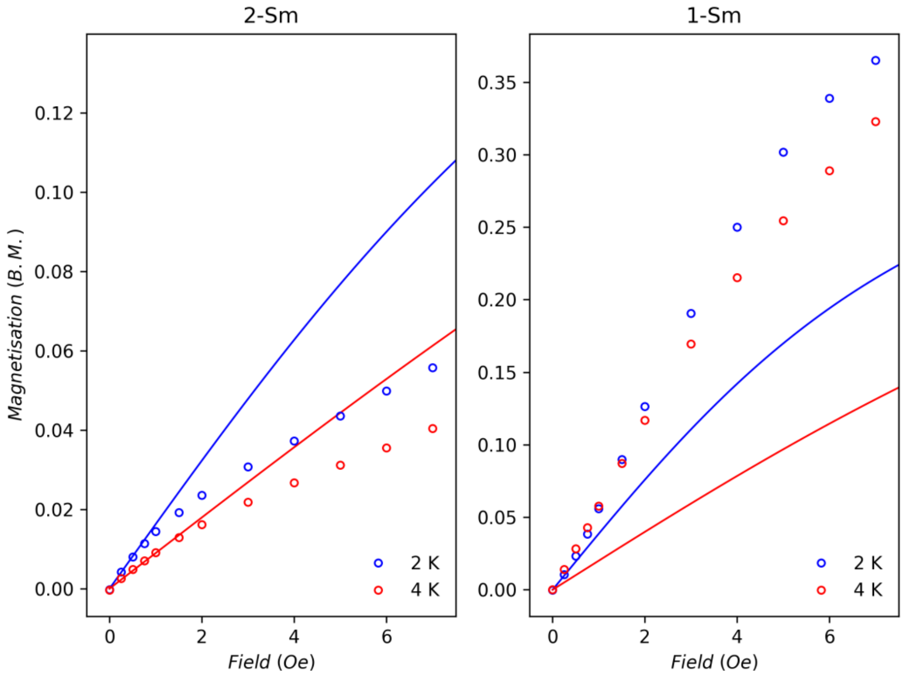

Figure S113. Field dependence of magnetization at 2 and 4 K for **2-Sm** (left) and **1-Sm** (right). Circles and solid lines are the experimental and CASSCF-SO calculated values, respectively.

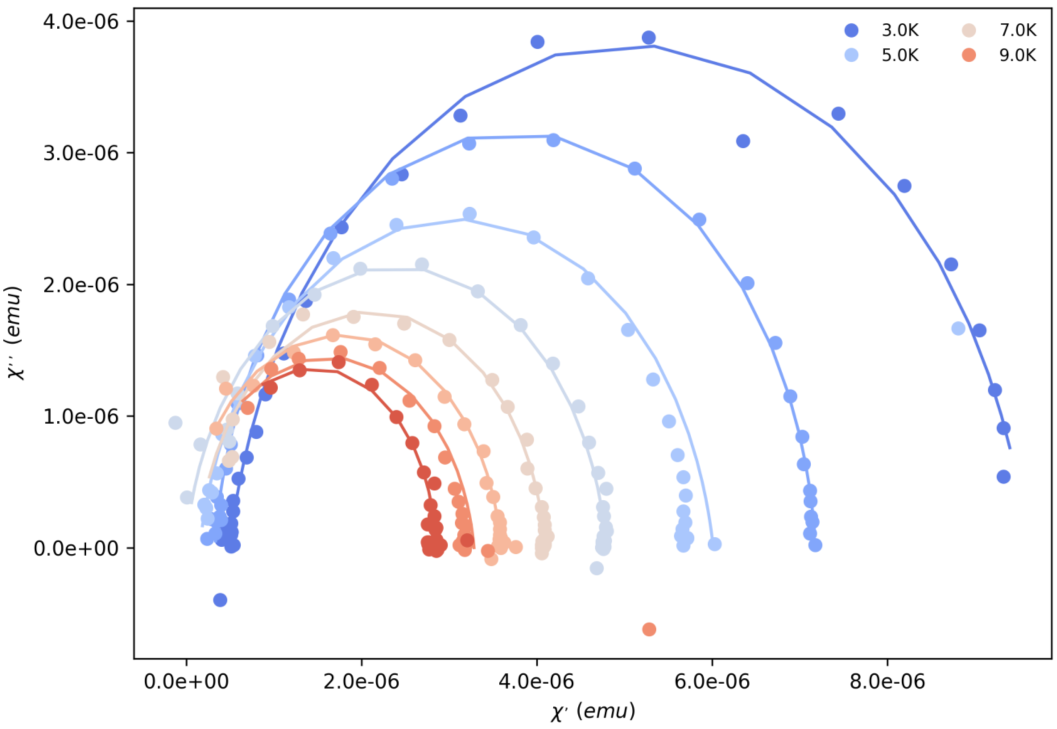

Figure S114. Cole-Cole plot for **1-Ce** in a 0.1 T DC field. Solid lines are fits to the generalized Debye model, giving $0.11 \leq \alpha \leq 0.08$.

Table S7. Best fit parameters to the generalized Debye model for **1-Ce** in a 0.1 T DC field.

**Temperature**	τ	χ_S	χ_T	α
3.0	0.10312	4.90E-07	9.60E-06	0.11155
4.0	0.01849	3.30E-07	7.18E-06	0.05229
5.0	0.00547	1.50E-07	6.01E-06	0.10193
6.0	0.00180	0.00E+00	4.79E-06	0.07473
7.0	0.00085	1.30E-07	4.09E-06	0.06309
8.0	0.00043	-4.00E-08	3.59E-06	0.07266
9.0	0.00025	-1.00E-08	3.28E-06	0.08242
10.0	0.00017	6.00E-08	2.84E-06	0.01400

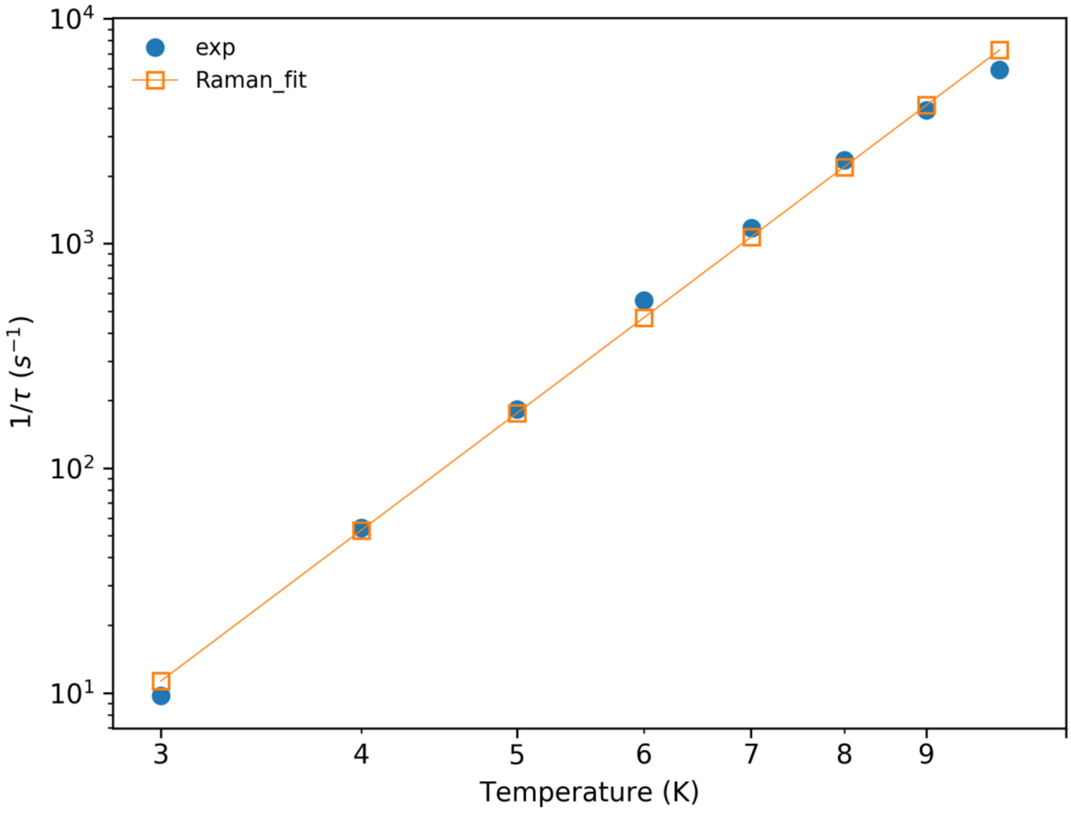

Figure S115. Fitting of relaxation rates for **1-Ce** in a 0.1 T DC field.

Table S8. Best fit parameters to the relaxation rates for **1-Ce** in a 0.1 T DC field.

**Model**	***C*** **($s^{-1}K^{-n}$)**	***n***
$\tau^{-1} = CT^n$	3.08E-2	5.37

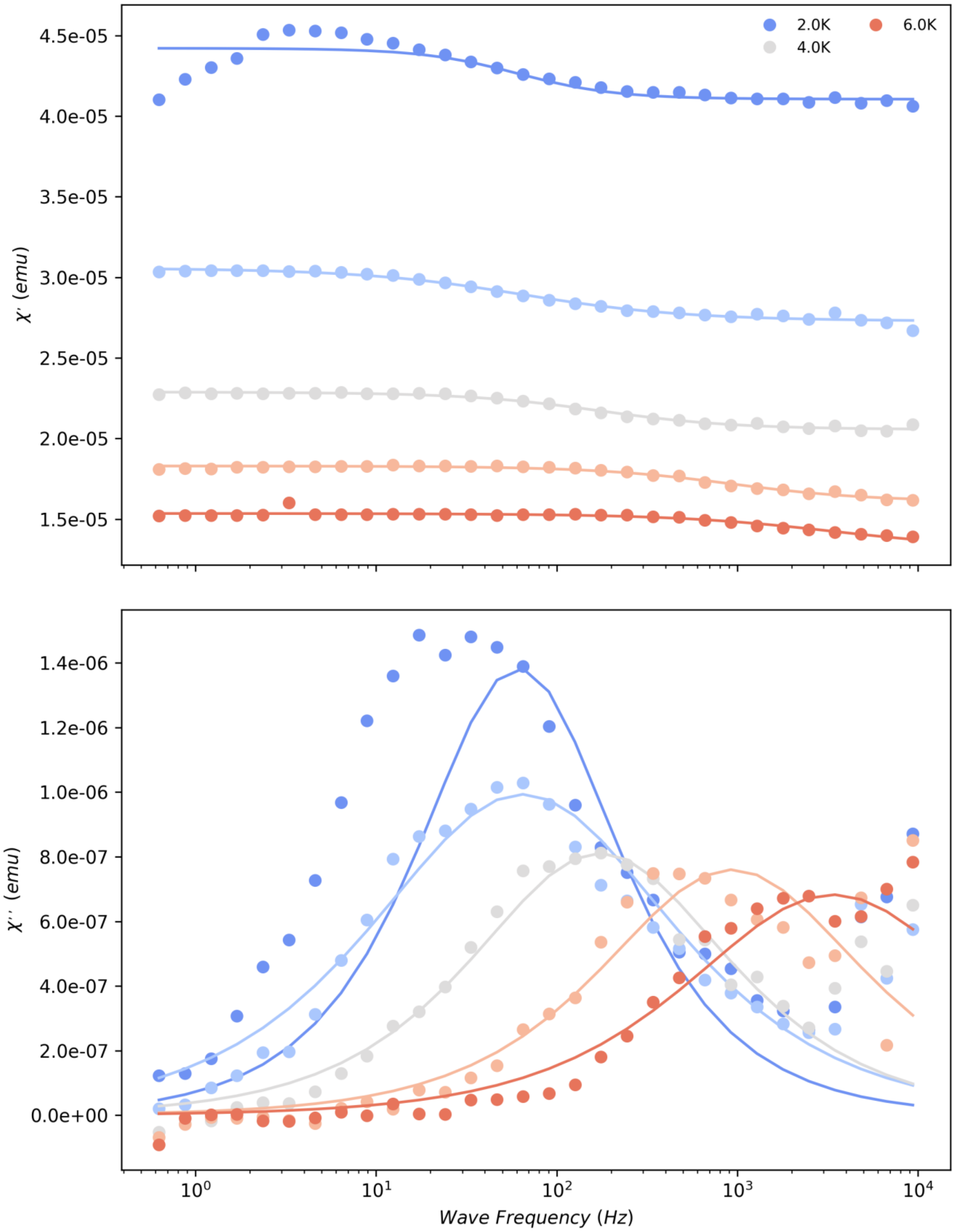

Figure S116. In-phase (top) and out-of-phase (bottom) ac susceptibilities for **2-Ce** in a zero field. Solid lines are fits to the generalized Debye model, giving $0.09 \leq \alpha \leq 0.30$.

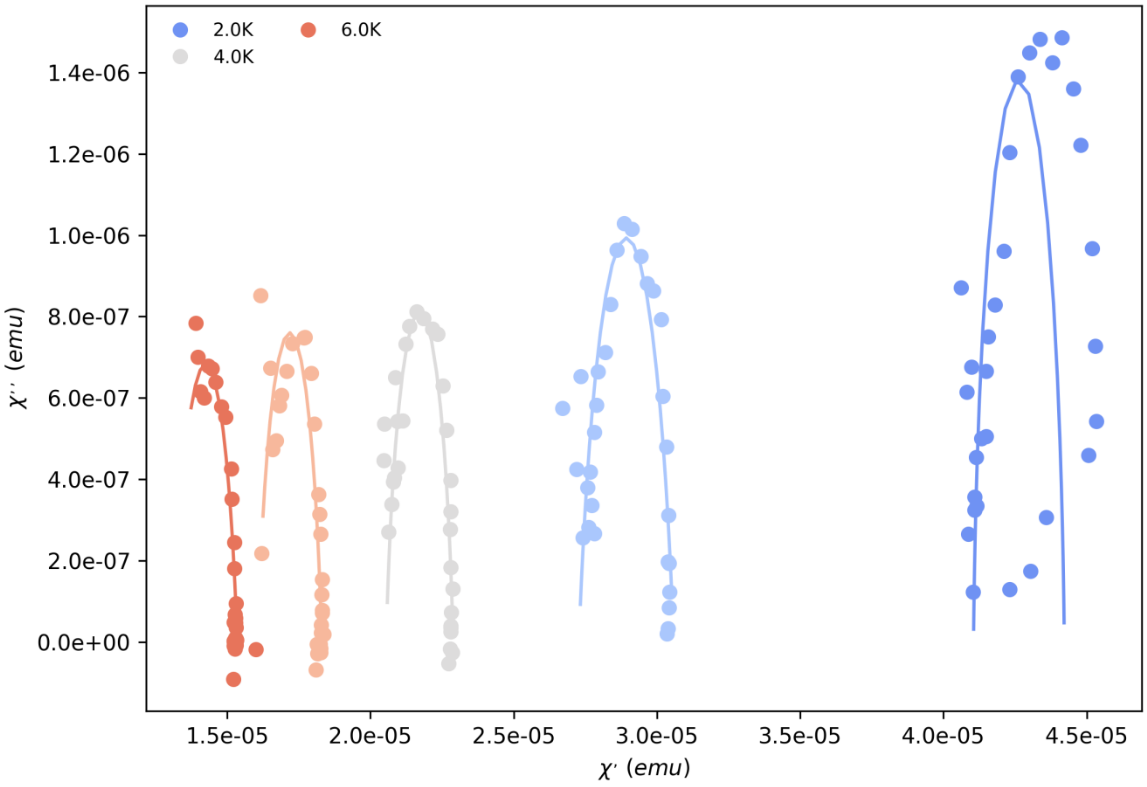

Figure S117. Cole-Cole plot for **2-Ce** in zero field. Solid lines are fits to the generalized Debye model, giving $0.09 \leq \alpha \leq 0.30$.

Table S9. Best fit parameters to the generalized Debye model for **2-Ce** in zero field.

**Temperature**	τ	χ_S	χ_T	α
2.00	1.63E-02	4.11E-05	4.42E-05	0.086
3.00	1.54E-02	2.73E-05	3.06E-05	0.312
4.00	5.76E-03	2.06E-05	2.29E-05	0.228
5.00	1.07E-03	1.61E-05	1.83E-05	0.238
6.00	2.97E-04	1.31E-05	1.54E-05	0.296

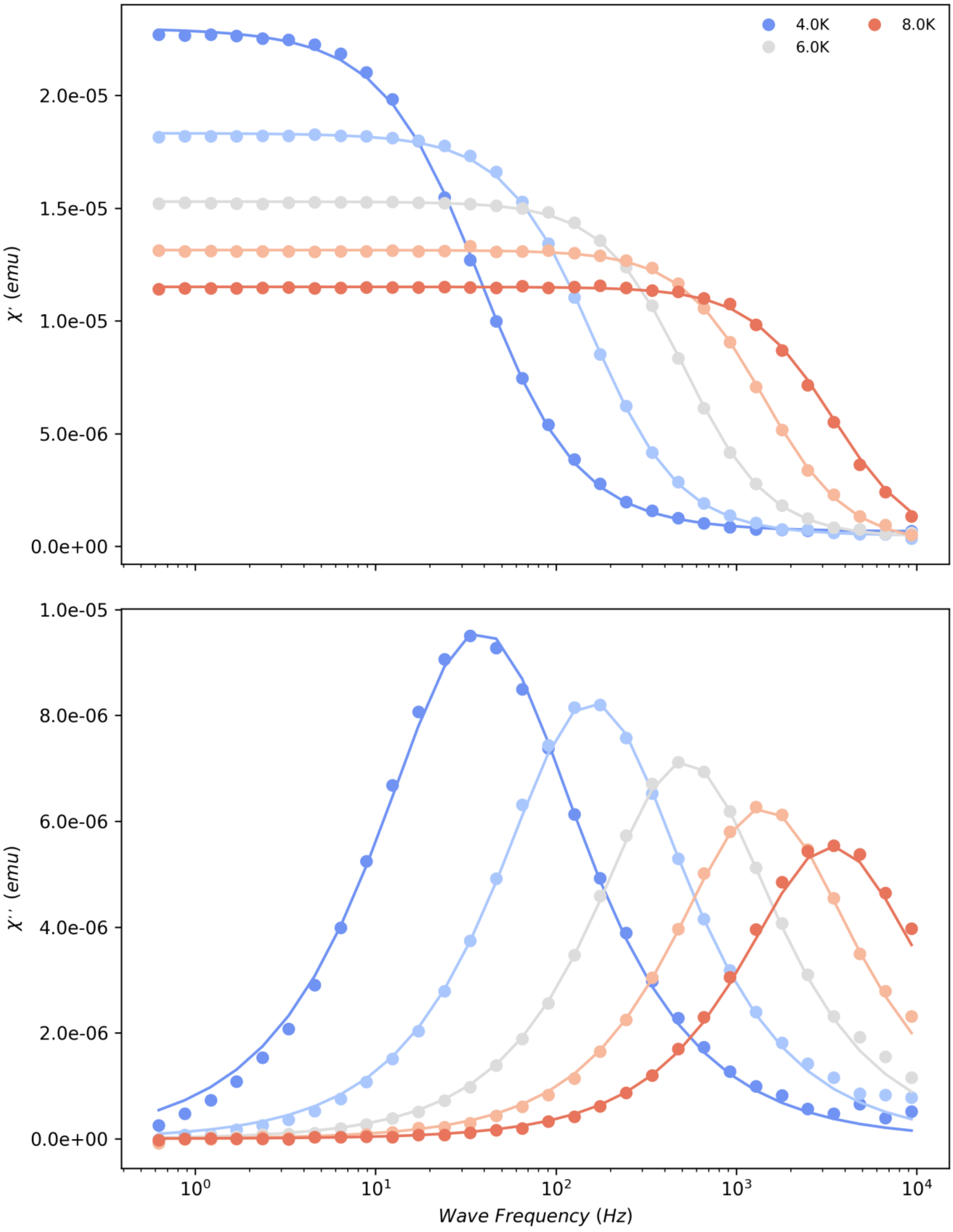

Figure S118. In-phase (top) and out-of-phase (bottom) AC susceptibilities for **2-Ce** in a 0.1 T DC field. Solid lines are fits to the generalized Debye model, giving $0.02 \leq \alpha \leq 0.09$.

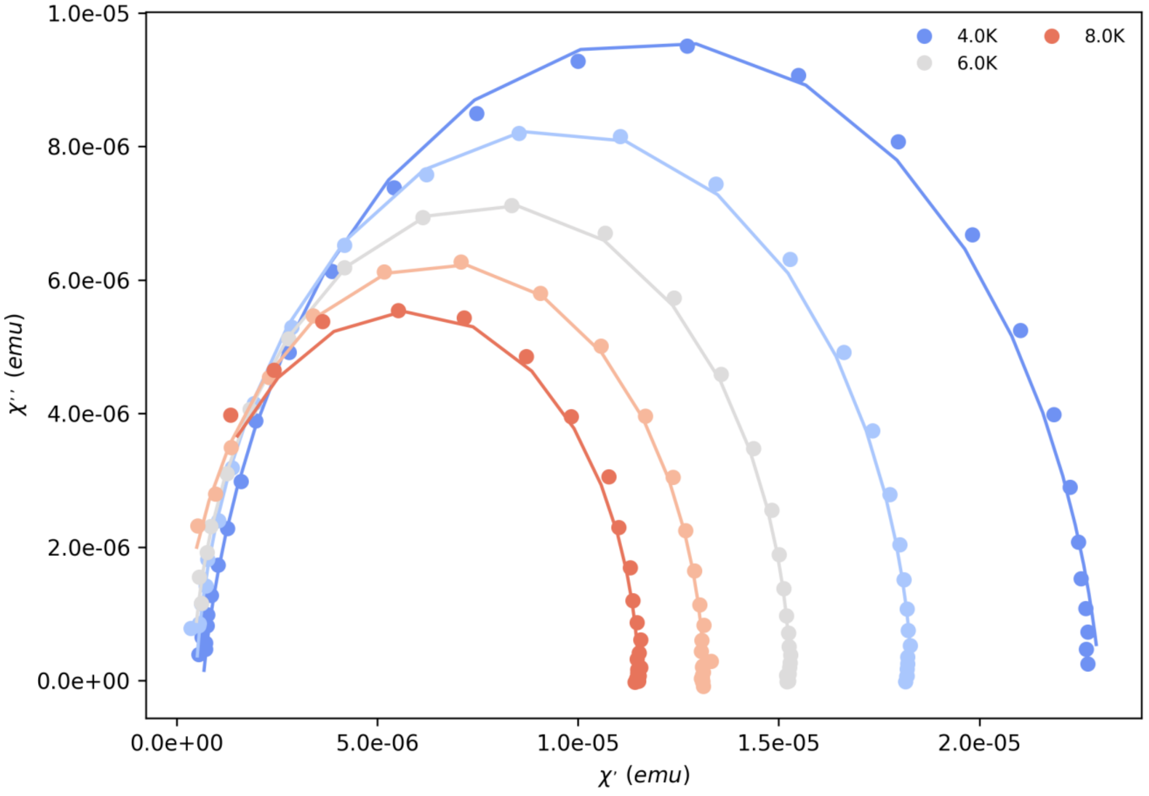

Figure S119. Cole-Cole plot for **2-Ce** in a 0.1 T DC field. Solid lines are fits to the generalized Debye model, giving 0.02 ≤ α ≤ 0.09.

Table S10. Best fit parameters to the generalized Debye model for **2-Ce** in a 0.1 T DC field.

**Temperature**	τ	χ_S	χ_T	α
4.00	2.63E-02	6.50E-07	2.30E-05	0.0973
5.00	6.31E-03	4.70E-07	1.83E-05	0.0499
6.00	1.92E-03	4.00E-07	1.53E-05	0.0271
7.00	7.08E-04	9.00E-08	1.32E-05	0.0275
8.00	2.95E-04	0.00E+00	1.15E-05	0.0255

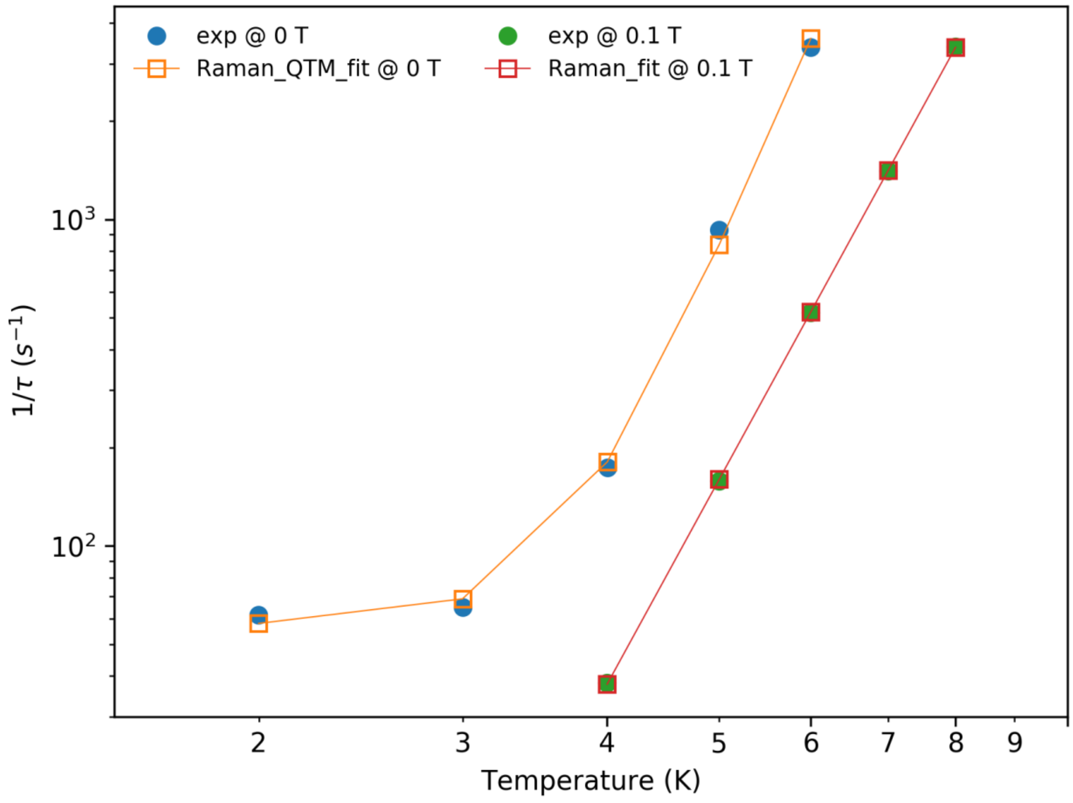

Figure S120. Fitting of relaxation rates for **2-Ce**.

Table S11. Best fit parameters to the relaxation rates for **2-Ce**.

**Model**	***H*** **(T)**	***C*** **($s^{-1}K^{-n}$)**	***n***	τ_{QTM}
$\tau^{-1} = CT^n + \tau_{QTM}^{-1}$	0	1.28E-3	8.28	1.74E-2
$\tau^{-1} = CT^n$	0.1	4.75E-3	6.48	-

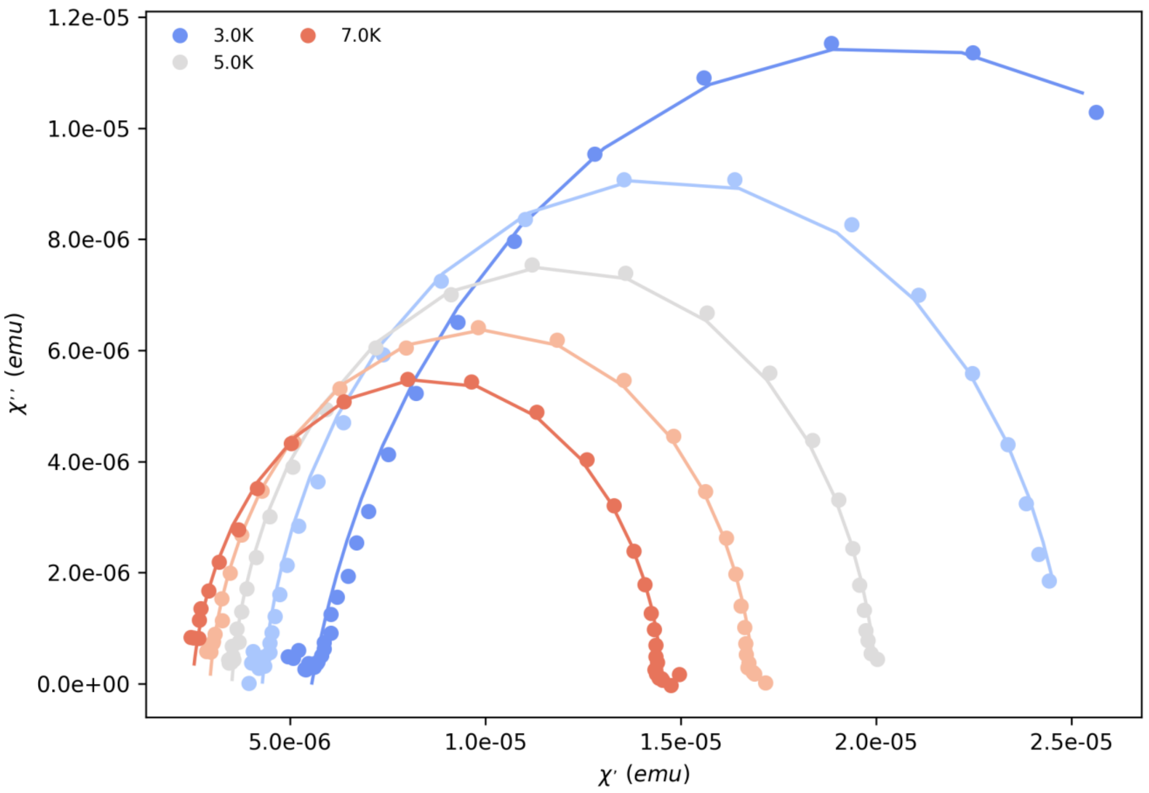

Figure S121. Cole-Cole plot for **1-Nd** in a 0.1 T DC field. Solid lines are fits to the generalized Debye model, giving 0.02 ≤ α ≤ 0.09.

Table S12. Best fit parameters to the generalized Debye model for **1-Nd** in a 0.1 T DC field.

**Temperature**	τ	χ_S	χ_T	α
3.00	9.43E-01	5.55E-06	3.50E-05	0.1569
4.00	1.25E-01	4.29E-06	2.49E-05	0.0811
5.00	3.18E-02	3.51E-06	2.00E-05	0.0604
6.00	1.10E-02	2.94E-06	1.69E-05	0.0568
7.00	4.45E-03	2.50E-06	1.45E-05	0.0589

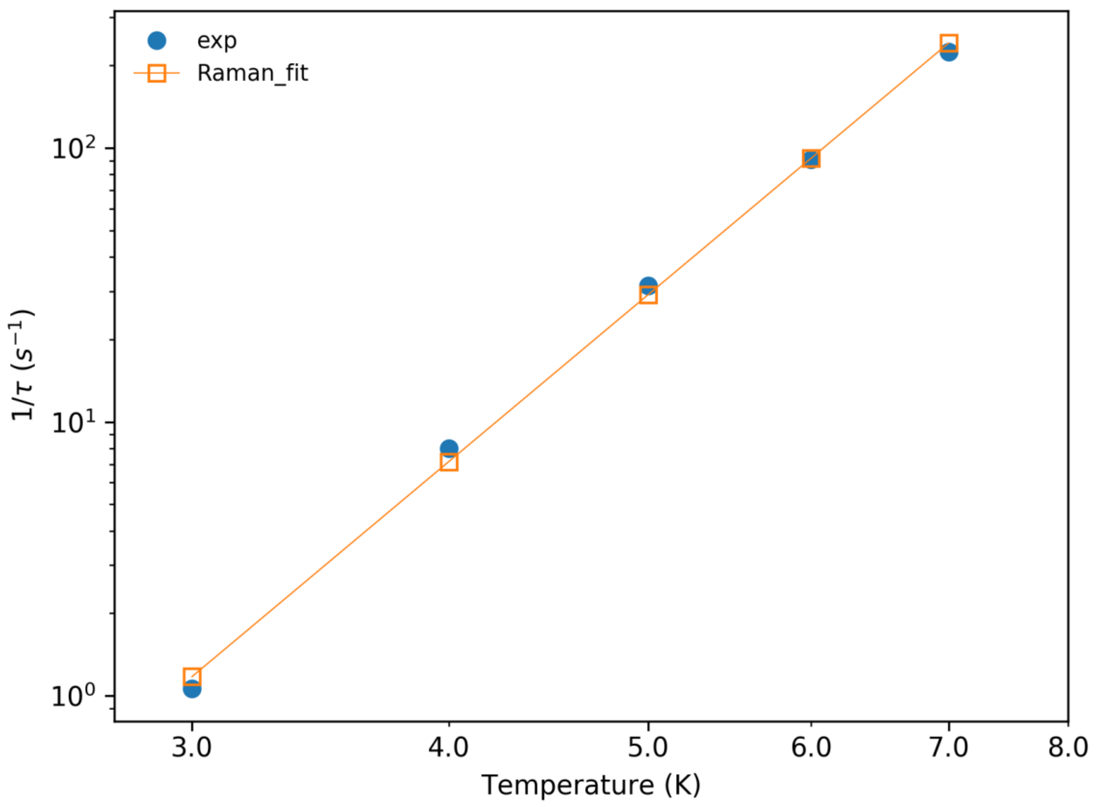

Figure S122. Fitting of relaxation rates for **1-Nd** in a 0.1 T DC field.

Table S13. Best fit parameters to the relaxation rates for **1-Nd** in a 0.1 T DC field.

**Model**	***C*** **($s^{-1}K^{-n}$)**	***n***
$\tau^{-1} = CT^n$	1.17E-3	6.29

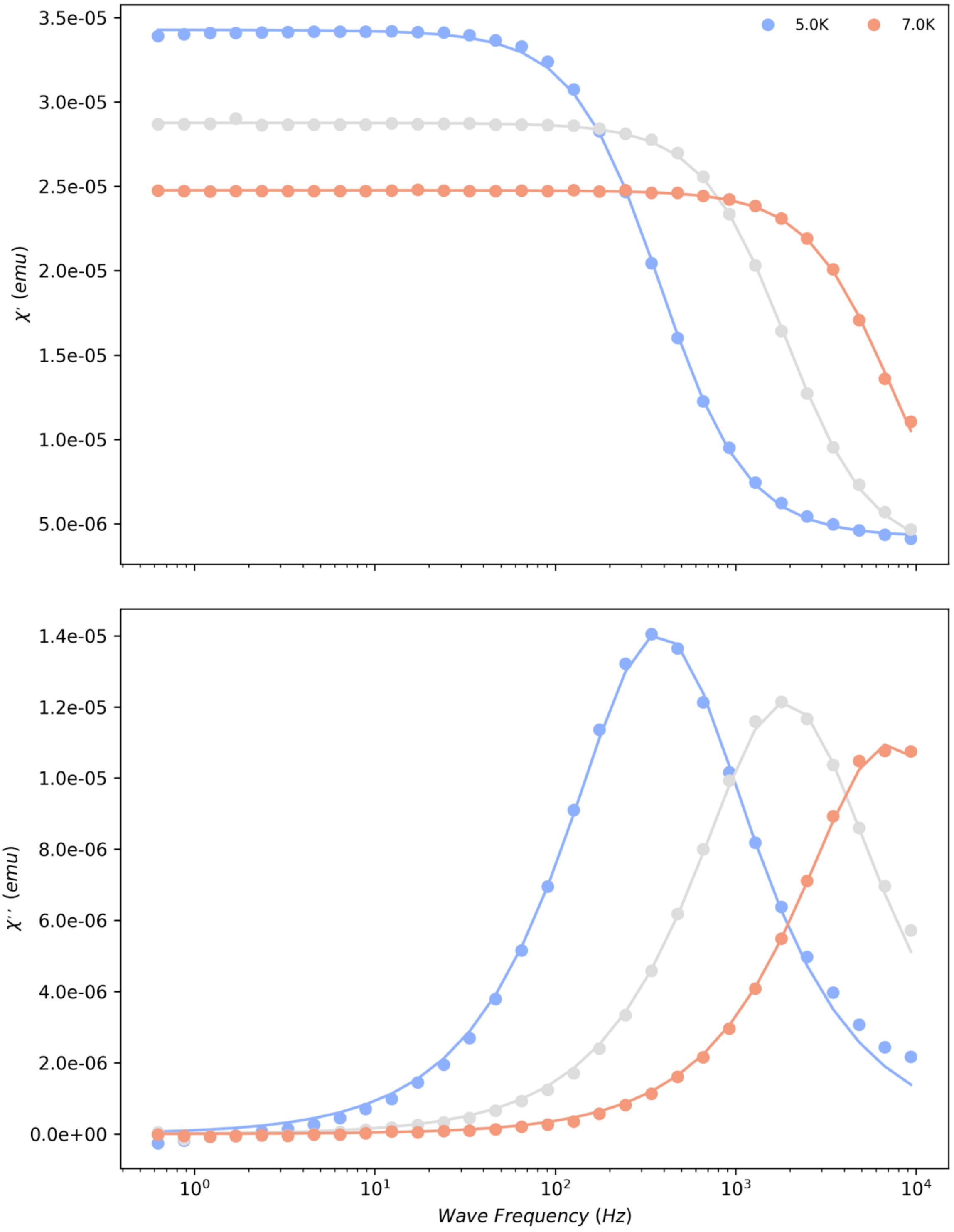

Figure S123. In-phase (top) and out-of-phase (bottom) AC susceptibilities for **2-Nd** in a 0.1 T DC field. Solid lines are fits to the generalized Debye model, giving $0.03 \leq \alpha \leq 0.04$.

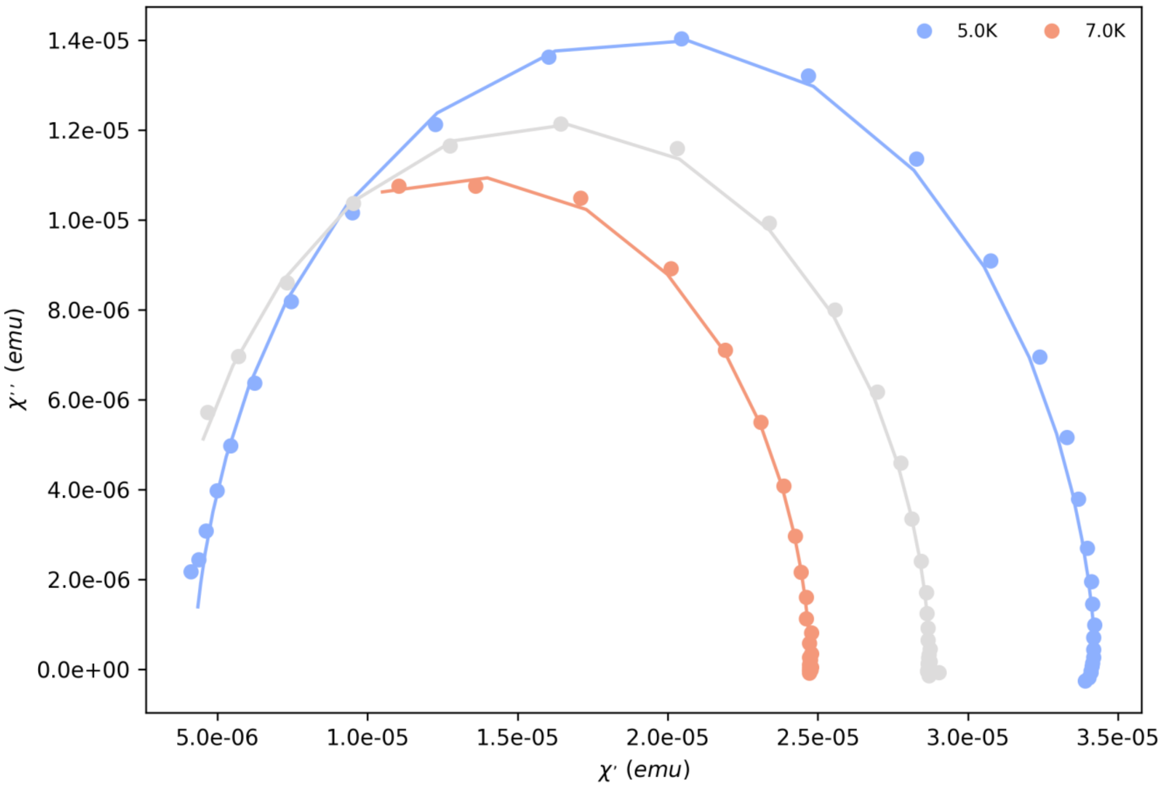

Figure S124. Cole-Cole plot for **2-Nd** in a 0.1 T DC field. Solid lines are fits to the generalized Debye model, giving 0.03 ≤ α ≤ 0.04.

Table S14. Best fit parameters to the generalized Debye model for **2-Nd** in a 0.1 T DC field.

**Temperature**	$\boldsymbol{\tau}$	χ_S	χ_T	$\boldsymbol{\alpha}$
5.00	2.64E-03	4.19E-06	3.43E-05	0.0430
6.00	5.27E-04	3.16E-06	2.88E-05	0.0336
7.00	1.39E-04	1.75E-06	2.48E-05	0.0309

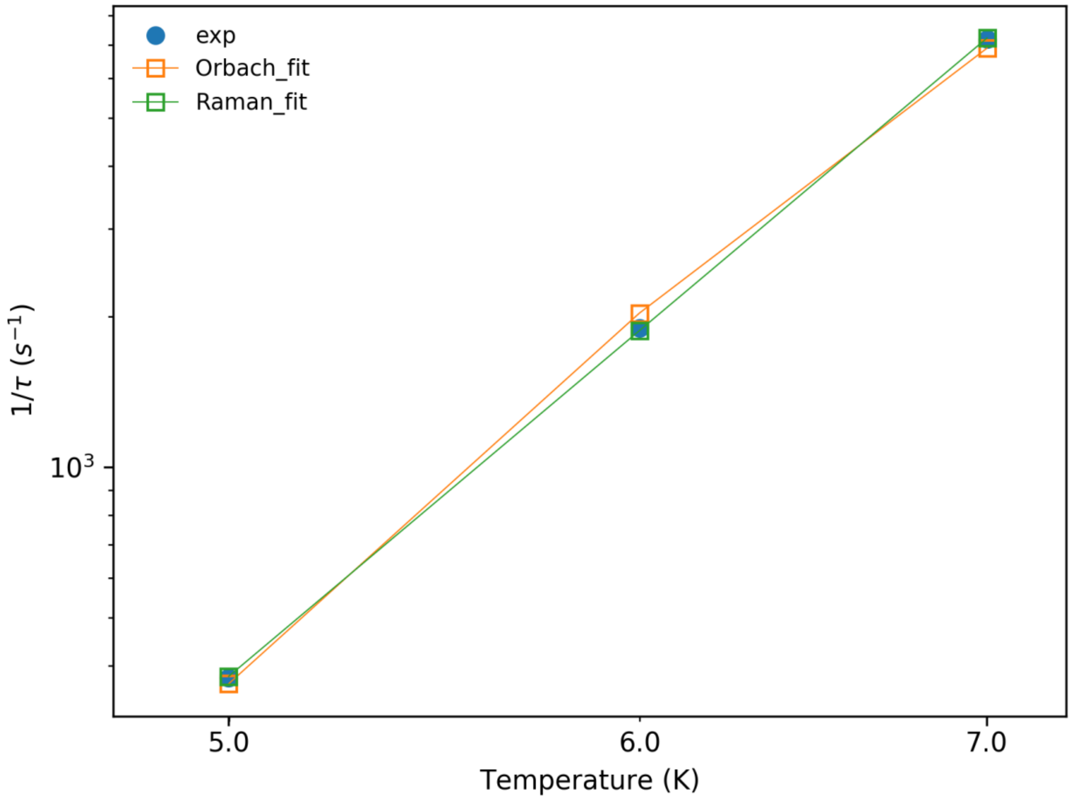

Figure S125. Fitting of relaxation rates for **2-Nd** in a 0.1 T DC field.

Table S15. Best fit parameters to the relaxation rates for **2-Nd** in a 0.1 T DC field.

**Model**	τ_0 **(s)**	U_{eff} **(cm**$^{-1}$**)**	C **(s**$^{-1}$**K**$^{-n}$**)**	n
$\tau^{-1} = \tau_0^{-1} e^{\left(-U_{eff}/_{kT}\right)}$	9.64E-8	51.2	-	-
$\tau^{-1} = CT^n$	-	-	3.00E-4	8.74

10. EPR spectra

X-band spectra were recorded with a Bruker EMX spectrometer fitted with a Super High Q X-band resonator. Polycrystalline samples of the Kramers ions (Ce, Nd and Sm) were sealed in quartz Q-band EPR tubes under an inert atmosphere; we crushed all samples as best we could without decomposing the sample, but we note that some effects due to polycrystallinity remain in the spectra below. We observed field-induced orientation effects in some of the samples, and therefore we have immobilized these with eicosane (following a similar procedure to that described for the SQUID samples) to prevent orientation effects. The presence of a very sharp resonance at g = 2.00 is attributed to an impurity in the quartz EPR tubes, and serves as an internal reference for comparing relative intensities. EPR spectra are simulated with PHI[8] using an isotropic frequency-space Gaussian linewidth in all cases.

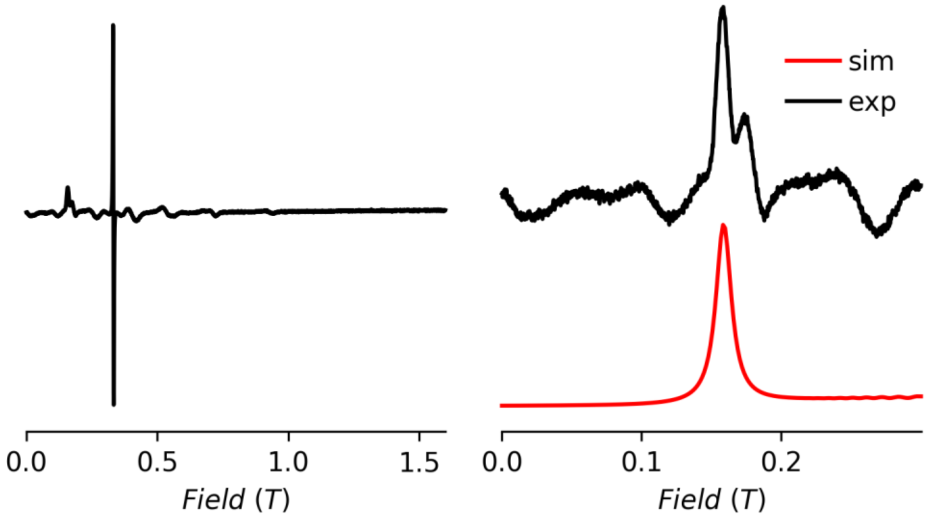

Figure S126. X-band EPR for **1-Ce** at 9.375 GHz and 10 K without eicosane, recorded with 20 dB attenuation and 4 G modulation. Simulated with S = 1/2, g_1 = g_2 = 0.1, g_3 = 4.22, linewidth = 0.70 GHz. The wiggly form of the spectrum is due to polycrystallinity and very anisotropic g-values. Sharp signal at g = 2 is an impurity in the tube.

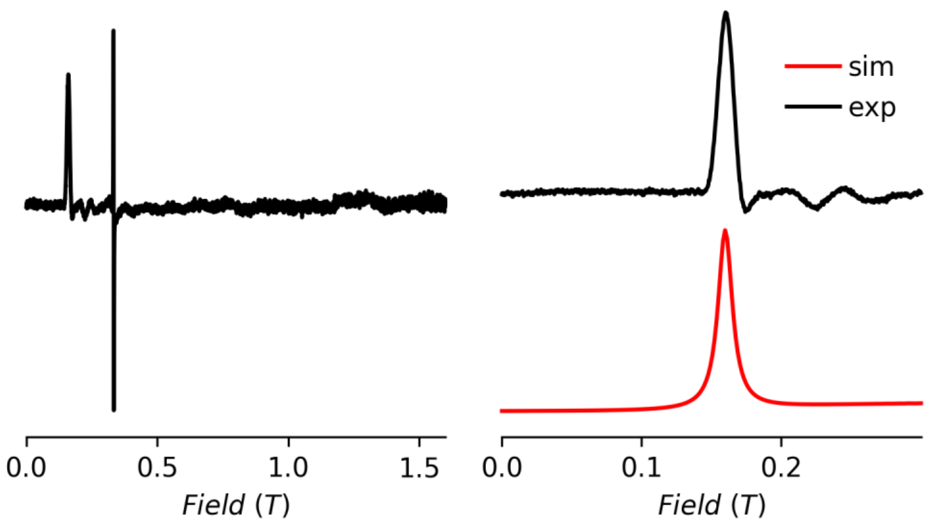

Figure S127. X-band EPR for **2-Ce** at 9.377 GHz and 5.5 K immobilised with eicosane, recorded with 20 dB attenuation and 4 G modulation. Simulated with S = 1/2, $g_1 = g_2 = 0.1$, $g_3 = 4.19$, linewidth = 0.70 GHz. Sharp signal at $g = 2$ is an impurity in the tube.

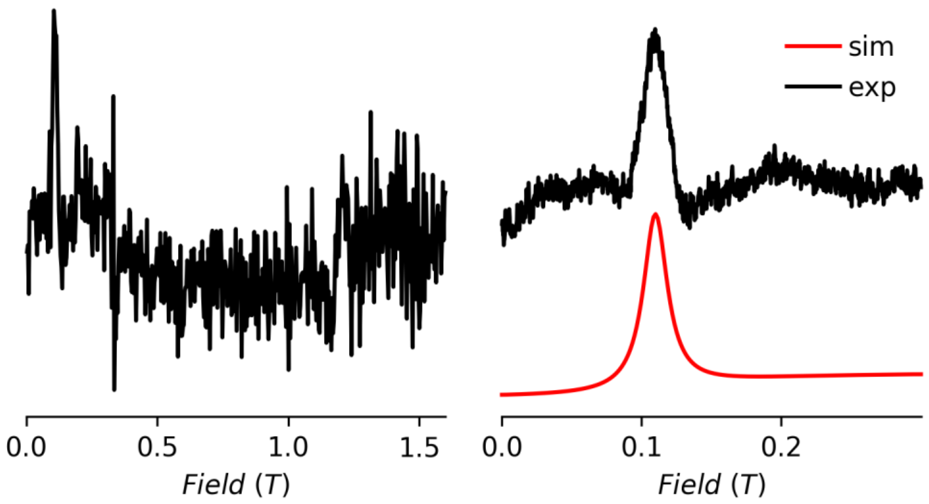

Figure S128. X-band EPR for **2-Nd** at 9.377 GHz and 5.5 K immobilised with eicosane, recorded with 20 dB attenuation and 4 G modulation. Simulated with S = 1/2, $g_1 = g_2 = 0.1$, $g_3 = 6.10$, linewidth = 1.77 GHz.

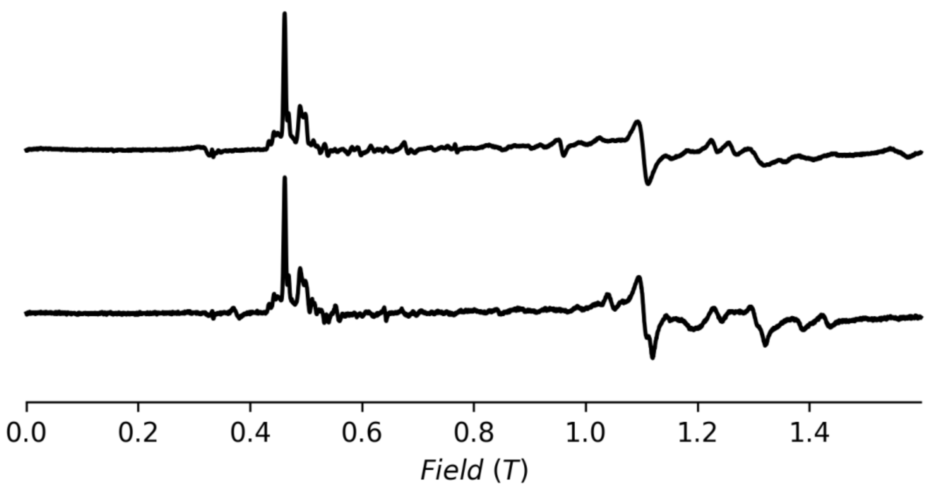

Figure S129. X-band EPR for **1-Sm** at 9.375 GHz and 6 K immobilised with eicosane, recorded with 20 dB attenuation and 4 G modulation, at two different sample orientations. Resonances at ~0.45 T are the parallel features of the anisotropic doublet corresponding to *g* ~ 1.5. Features at higher fields are due to polycrystallinity in the sample. Spectrum has been background corrected with an empty tube blank.

11. CASSCF-SO electronic structure

We used MOLCAS 8.0[9] to perform CASSCF-SO calculations of the **1-Ln** and **2-Ln** complexes in order to determine their electronic structures. We employed the molecular geometries of the metal-containing molecules from single crystal XRD structure with no optimization, taking the largest disorder component only. If the $[B(C_6F_5)_4]^-$ counter ion was in contact with the cation, it was also included in the calculations. Basis sets from ANO-RCC library[10,11] were employed with VTZP quality for Ln atoms, VDZP quality for the cyclopentadienyl C atoms and the equatorial chloride atom (**2-Ln** only), and VDZ quality for all remaining atoms, employing the second-order DKH transformation. Cholesky decomposition of the two-electron integrals with a threshold of 10^{-8} was performed to save disk space and reduce computational demand. The molecular orbitals (MOs) were optimized in state-averaged CASSCF calculations, where the number of 4f electrons in the active space of the seven f orbitals, the number of roots of each spin multiplicity are indicated in Table S16. Most of the resulting states were mixed together by spin-orbit coupling (Table S16), and these spin-orbit wavefunctions were decomposed into a CF Hamiltonian (below), and the magnetic susceptibility calculated (see Magnetism and EPR spectroscopy) using SINGLE_ANISO.[12]

Table S16. Computational details used for the CASSCF-SO calculations.

Ion	4f electrons	Spin multiplicity	Roots	States mixed by spin-orbit coupling
Ce^{III}	1	2	7	7
Pr^{III}	2	3, 1	21, 28	21, 28
Nd^{III}	3	4, 2	35, 112	35, 112
Sm^{III}	5	6, 4, 2	21, 224, 490	21, 128, 130

Table S17. Electronic structure of **1-Ce** calculated with CASSCF-SO at the solid-state geometry in zero-field, quantised along the g_3 direction of the ground doublet.

**Energy (cm^{-1})**	**Energy (K)**	$\boldsymbol{g_1}$	$\boldsymbol{g_2}$	$\boldsymbol{g_3}$	**angle (deg)**	**Wavefunction**	$\langle J_z \rangle$
0.0	0.0	0.05	0.10	4.16	-	$100\%\lvert\pm 5/2\rangle$	± 2.50
786.6	1131.7	0.48	0.52	2.21	2.74	$93\%\lvert\pm 3/2\rangle + 5\%\lvert\mp 3/2\rangle + 2\%\lvert\pm 1/2\rangle$	± 1.30
1577.1	2269.1	1.82	3.00	0.84	2.33	$94\%\lvert\pm 1/2\rangle + 4\%\lvert\mp 1/2\rangle + 2\%\lvert\pm 3/2\rangle$	± 0.43

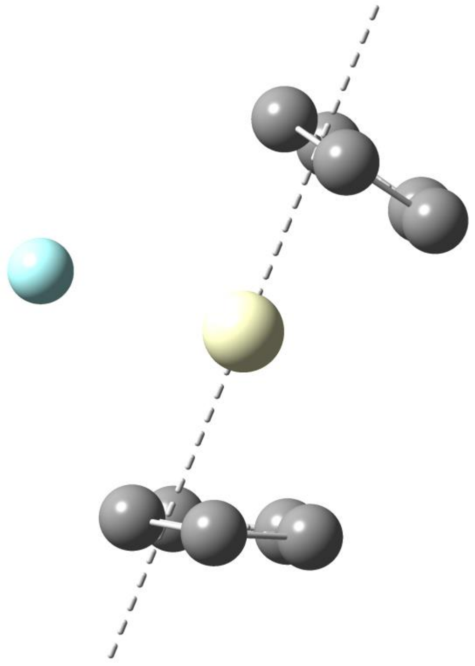

Figure S130. Representation of the g_3 direction in the molecular frame for **1-Ce**. *t*Bu groups, H-atoms and all of the $[B(C_6F_5)_4]^-$ except the closest F-atom are omitted for clarity.

Table S18. Electronic structure of **1-Ce** calculated with CASSCF-SO at the solid-state geometry without counterion in zero-field, quantised along the g_3 direction of the ground doublet.

Energy (cm^{-1})	Energy (K)	g_1	g_2	g_3	angle (deg)	Wavefunction	$<J_z>$
0.0	0.0	0.01	0.01	4.16	-	$100\%|\pm 5/2\rangle$	± 2.50
935.8	1346.4	0.01	0.15	2.25	1.72	$68\%|\pm 3/2\rangle + 32\%|\mp 3/2\rangle$	± 0.55
1784.1	2566.9	2.32	2.50	0.91	3.00	$96\%|\pm 1/2\rangle + 4\%|\mp 1/2\rangle$	± 0.46

Table S19. Electronic structure of **2-Ce** calculated with CASSCF-SO at the solid-state geometry in zero-field, quantised along the g_3 direction of the ground doublet.

Energy (cm^{-1})	Energy (K)	g_1	g_2	g_3	angle (deg)	Wavefunction	$<J_z>$
0.0	0.0	0.10	0.14	4.09	-	$97\%|\pm 5/2\rangle + 3\%|\mp 1/2\rangle$	± 2.45
352.6	507.3	1.66	1.69	1.85	67.34	$78\%|\pm 1/2\rangle + 19\%|\mp 1/2\rangle + 2\%|\mp 3/2\rangle$	± 1.03
1141.6	1642.5	0.33	0.52	4.19	88.77	$27\%|\pm 1/2\rangle + 51\%|\mp 1/2\rangle + 14\%|\pm 3/2\rangle + 6\%|\mp 3/2\rangle$	± 0.01

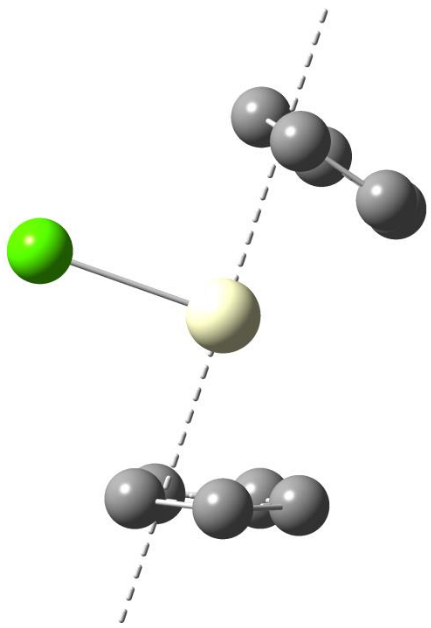

Figure S131. Representation of the ground state g_3 direction in the molecular frame for **2-Ce**. *t*Bu groups and H-atoms omitted for clarity.

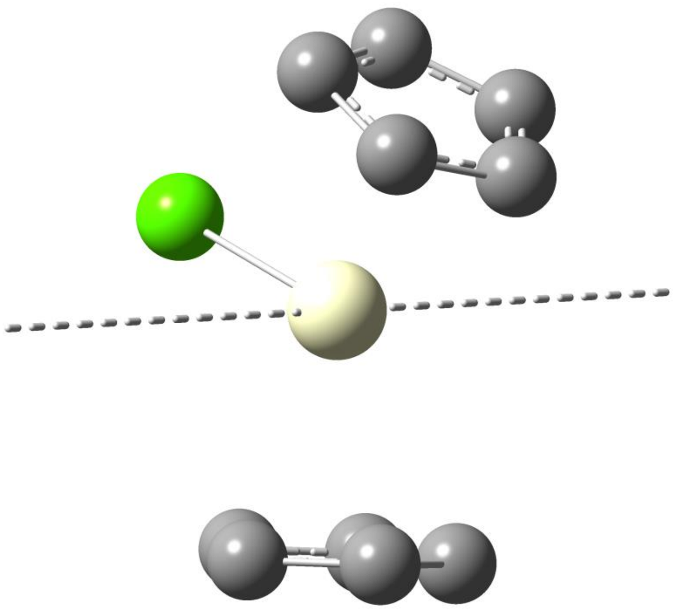

Figure S132. Representation of g_3 direction of the most excited Kramers doublet in the molecular frame for **2-Ce**. *t*Bu groups and H-atoms omitted for clarity.

Table S20. Electronic structure of **1-Pr** calculated with CASSCF-SO at the solid-state geometry in zero-field, quantised along the g_3 direction of the ground doublet.

**Energy (cm^{-1})**	**Energy (K)**	g_3	**angle (deg)**	**Wavefunction**	$<J_z>$
0.00	0.00	6.18	-	50%|−4⟩ + 50%|+4⟩	0.00
0.31	0.44			50%|−4⟩ + 50%|+4⟩	0.00
852.05	1225.91	4.76	10.2	49%|−3⟩ + 49%|+3⟩	0.00
863.29	1242.07			48%|−3⟩ + 48%|+3⟩ + 2%|−2⟩ + 2%|+2⟩	0.00
1050.72	1511.75	3.28	20.8	48%|−2⟩ + 48%|+2⟩ + 1%|−4⟩ + 1%|+4⟩	0.00
1054.69	1517.46			47%|−2⟩ + 47%|+2⟩ + 2%|−3⟩ + 2%|+3⟩	0.00
1210.68	1741.89	-	-	49%|−1⟩ + 49%|+1⟩ + 2%|0⟩	0.00
1377.28	1981.58	-	-	49%|−1⟩ + 49%|+1⟩ + 1%|−2⟩ + 1%|+2⟩	0.00
1403.77	2019.70	-	-	97%|0⟩	0.00

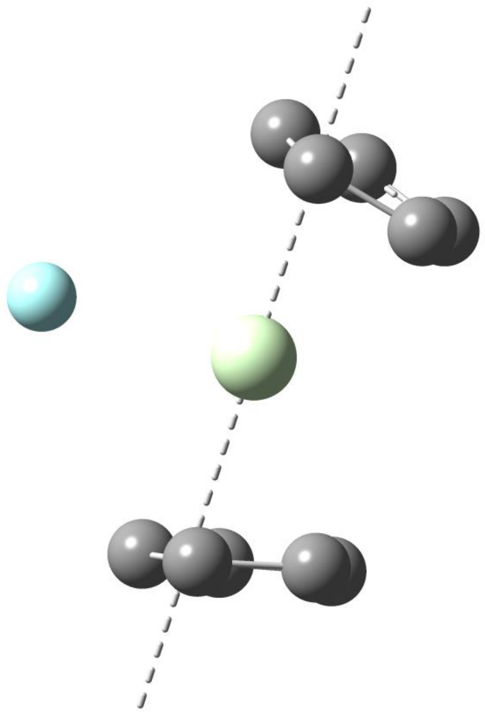

Figure S133. Representation of g_3 direction in the molecular frame for **1-Pr**. *t*Bu groups and H-atoms except the closest F-atom are omitted for clarity.

Table S21. Electronic structure of **1-Pr** calculated with PHI using the CFPs from CASSCF-SO at the solid-state geometry in a 0.1 T applied-field, quantised along the g_3 direction of the ground doublet.

**Energy (cm^{-1})**	**Energy (K)**	**Wavefunction**	$<J_z>$
0.00	0.00	$65\%\|-4\rangle + 34\%\|+4\rangle$	-1.25
0.95	1.36	$34\%\|-4\rangle + 65\%\|+4\rangle$	1.25
852.20	1226.12	$50\%\|-3\rangle + 48\%\|+3\rangle$	-0.07
861.30	1239.21	$47\%\|-3\rangle + 49\%\|+3\rangle$	0.07
1055.00	1517.90	$49\%\|-2\rangle + 47\%\|+2\rangle$	-0.05
1061.00	1526.53	$47\%\|-2\rangle + 49\%\|+2\rangle$	0.05
1192.00	1715.01	$49\%\|-1\rangle + 49\%\|+1\rangle + 1\%\|0\rangle$	0.00
1373.00	1975.43	$49\%\|-1\rangle + 49\%\|+1\rangle$	0.00
1415.00	2035.86	$98\%\|0\rangle$	0.00

Table S22. Electronic structure of **1-Pr** calculated with CASSCF-SO at the solid-state geometry without counterion in zero-field, quantised along the g_3 direction of the ground doublet.

**Energy (cm^{-1})**	**Energy (K)**	g_3	**angle (deg)**	**Wavefunction**	$<J_z>$
0.00	0.00	6.20	--	$50\%\|-4\rangle + 50\%\|+4\rangle$	0.00
0.14	0.21			$50\%\|-4\rangle + 50\%\|+4\rangle$	0.00
933.82	1343.56	4.68	7.15	$49\%\|-3\rangle + 49\%\|+3\rangle$	0.00
936.89	1347.97			$48\%\|-3\rangle + 48\%\|+3\rangle + 1\%\|-2\rangle + 1\%\|+2\rangle$	0.00
1133.70	1631.13	3.19	20.58	$48\%\|-2\rangle + 48\%\|+2\rangle + 1\%\|-4\rangle + 1\%\|+4\rangle$	0.00
1156.01	1663.23			$47\%\|-2\rangle + 47\%\|+2\rangle + 2\%\|-3\rangle + 2\%\|+3\rangle$	0.00
1431.24	2059.22	1.54	2.56	$49\%\|-1\rangle + 49\%\|+1\rangle + 2\%\|0\rangle$	0.00
1464.14	2106.56			$49\%\|-1\rangle + 49\%\|+1\rangle + 1\%\|-2\rangle + 1\%\|+2\rangle$	0.00
1560.86	2245.72			$95\%\|0\rangle$	0.00

Table S23. Electronic structure of **2-Pr** calculated with CASSCF-SO at the solid-state geometry in zero-field, quantised along the g_3 direction of the ground doublet.

**Energy (cm^{-1})**	**Energy (K)**	g_3	**Wavefunction**	$\langle J_z \rangle$
0.00	0.00	5.87	43%$\lvert -4\rangle$ + 43%$\lvert +4\rangle$ + 6%$\lvert -2\rangle$ + 6%$\lvert +2\rangle$	0.00
17.56	25.26		46%$\lvert -4\rangle$ + 46%$\lvert +4\rangle$ + 4%$\lvert -2\rangle$ + 4%$\lvert +2\rangle$	0.00
433.31	623.43	-	24%$\lvert -3\rangle$ + 24%$\lvert +3\rangle$ + 25%$\lvert -1\rangle$ + 25%$\lvert +1\rangle$	0.00
540.06	777.02	-	26%$\lvert -2\rangle$ + 26%$\lvert +2\rangle$ + 33%$\lvert 0\rangle$ + 6%$\lvert -4\rangle$ + 6%$\lvert +4\rangle$	0.00
567.53	816.55	-	47%$\lvert -3\rangle$ + 47%$\lvert +3\rangle$ + 1%$\lvert -4\rangle$ + 1%$\lvert +4\rangle$	0.00
740.98	1066.09	-	26%$\lvert -3\rangle$ + 26%$\lvert +3\rangle$ + 23%$\lvert -1\rangle$ + 23%$\lvert +1\rangle$	0.00
843.44	1213.52	-	46%$\lvert -2\rangle$ + 46%$\lvert +2\rangle$	0.00
1159.04	1667.58	-	64%$\lvert 0\rangle$ + 17%$\lvert -2\rangle$ + 17%$\lvert +2\rangle$	0.00
1186.10	1706.52	-	49%$\lvert -1\rangle$ + 49%$\lvert +1\rangle$	0.00

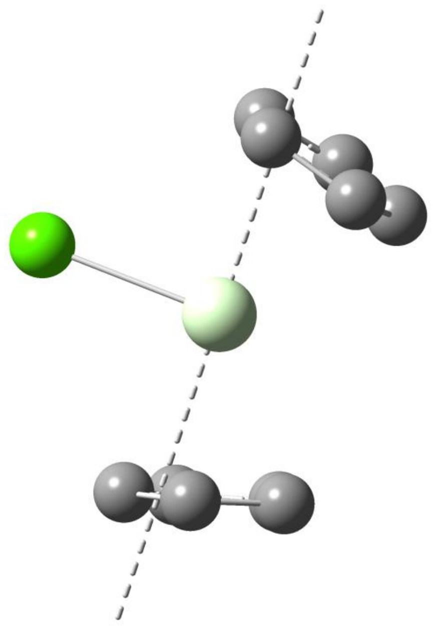

Figure S134. Representation of g_3 direction in the molecular frame for **2-Pr**. *t*Bu groups and H-atoms omitted for clarity.

Table S24. Electronic structure of **2-Pr** calculated with PHI using the CFPs from CASSCF-SO at the solid-state geometry in a 0.1 T applied-field, quantised along the g_3 direction of the ground doublet.

Energy (cm^{-1})	Energy (K)	Wavefunction	$<J_z>$
0.00	0.00	$43\%\|-4\rangle + 43\%\|+4\rangle + 6\%\|-2\rangle + 6\%\|+2\rangle$	-0.06
17.44	25.09	$46\%\|-4\rangle + 46\%\|+4\rangle + 4\%\|-2\rangle + 4\%\|+2\rangle$	0.06
425.10	611.62	$24\%\|-3\rangle + 24\%\|+3\rangle + 25\%\|-1\rangle + 25\%\|+1\rangle$	-0.00
546.80	786.72	$26\%\|-2\rangle + 26\%\|+2\rangle + 33\%\|0\rangle + 6\%\|-4\rangle + 6\%\|+4\rangle$	-0.00
565.50	813.62	$47\%\|-3\rangle + 47\%\|+3\rangle + 1\%\|-4\rangle + 1\%\|+4\rangle$	0.00
735.60	1058.36	$26\%\|-3\rangle + 26\%\|+3\rangle + 23\%\|-1\rangle + 23\%\|+1\rangle$	0.00
845.90	1217.05	$46\%\|-2\rangle + 46\%\|+2\rangle$	0.00
1159.00	1667.53	$64\%\|0\rangle + 17\%\|-2\rangle + 17\%\|+2\rangle$	0.00
1184.00	1703.50	$49\%\|-1\rangle + 49\%\|+1\rangle$	0.00

Table S25. Electronic structure of **1-Nd** calculated with CASSCF-SO at the solid-state geometry in zero-field, quantised along the g_3 direction of the ground doublet.

Energy (cm^{-1})	Energy (K)	g_1	g_2	g_3	angle (deg)	Wavefunction	$<J_z>$
0.00	0.00	0.02	0.02	6.32	-	$86\%\|\pm 9/2\rangle + 9\%\|\pm 7/2\rangle + 4\%\|\pm 5/2\rangle$	$\pm$ 4.27
131.06	188.56	0.11	0.14	5.01	27.66	$86\%\|\pm 7/2\rangle + 9\%\|\pm 9/2\rangle + 4\%\|\pm 3/2\rangle$	$\pm$ 3.50
423.82	609.78	1.08	1.52	3.28	81.69	$81\%\|\pm 1/2\rangle + 11\%\|\pm 5/2\rangle + 4\%\|\mp 3/2\rangle + 3\%\|\pm 3/2\rangle$	$\pm$ 0.70
537.03	772.66	1.29	1.55	4.70	71.28	$60\%\|\pm 5/2\rangle + 30\%\|\mp 3/2\rangle + 3\%\|\pm 1/2\rangle + 2\%\|\pm 9/2\rangle$	$\pm$ 1.03
626.54	901.45	0.47	0.67	4.64	85.42	$49\%\|\mp 3/2\rangle + 22\%\|\pm 5/2\rangle + 12\%\|\pm 1/2\rangle + 11\%\|\pm 3/2\rangle +$	$\pm$ 0.00

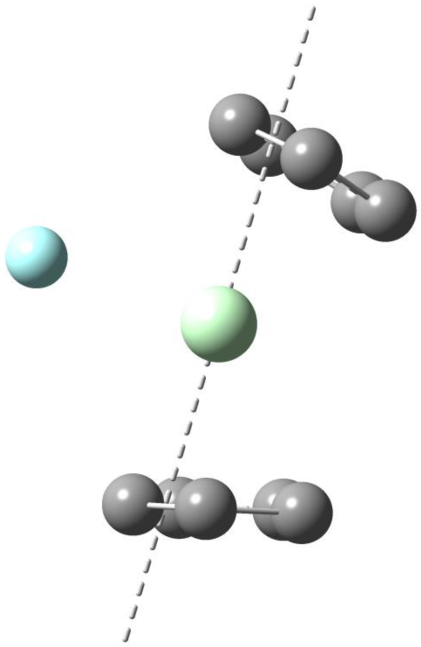

Figure S135. Representation of the ground state g_3 direction in the molecular frame for **1-Nd**. *t*Bu groups, H-atoms and all of the $[B(C_6F_5)_4]^-$ except the closest F-atom are omitted for clarity.

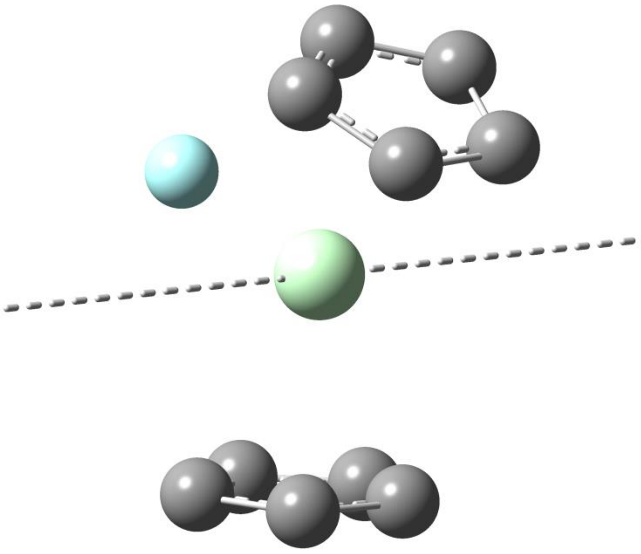

Figure S136. Representation of g_3 direction of the most excited Kramers doublet in the molecular frame for **1-Nd**. *t*Bu groups, H-atoms and all of the $[B(C_6F_5)_4]^-$ except the closest F-atom are omitted for clarity.

Table S26. Electronic structure of **1-Nd** calculated with CASSCF-SO at the solid-state geometry without the counterion in zero-field, quantised along the g_3 direction of the ground doublet.

Energy (cm^{-1})	Energy (K)	g_1	g_2	g_3	angle (deg)	Wavefunction	$<J_z>$
0.00	0.00	0.01	0.01	6.37	-	$86\%\|\pm 9/2\rangle + 9\%\|\pm 7/2\rangle + 3\%\|\pm 5/2\rangle$	$\pm$ 4.30
130.29	187.45	0.01	0.04	5.04	27.53	$89\%\|\pm 7/2\rangle + 9\%\|\pm 9/2\rangle + 2\%\|\pm 3/2\rangle$	$\pm$ 3.55
464.14	667.79	0.44	0.68	4.71	78.31	$83\%\|\mp 1/2\rangle + 13\%\|\pm 3/2\rangle$	$\pm$ 0.24
587.54	845.33	1.84	2.56	2.85	28.11	$80\%\|\pm 5/2\rangle + 13\%\|\mp 3/2\rangle + 3\%\|\pm 9/2\rangle$	$\pm$ 1.90
671.90	966.70	0.97	1.17	4.10	85.07	$44\%\|\mp 3/2\rangle + 27\%\|\pm 3/2\rangle + 8\%\|\pm 1/2\rangle + 7\%\|\mp 5/2\rangle + 6\%\|\pm 5/2\rangle + 6\%\|\mp 1/2\rangle +$	$\pm$ 0.31

Table S27. Electronic structure of **2-Nd** calculated with CASSCF-SO at the solid-state geometry in zero-field, quantised along the g_3 direction of the ground doublet.

Energy (cm^{-1})	Energy (K)	g_1	g_2	g_3	angle (deg)	Wavefunction	$<J_z>$
0.00	0.00	0.03	0.23	6.00	-	$74\%\|\pm 9/2\rangle + 16\%\|\pm 7/2\rangle + 5\%\|\pm 3/2\rangle + 3\%\|\pm 5/2\rangle$	$\pm$ 4.02
71.91	103.46	0.51	1.99	4.45	37.16	$50\%\|\pm 7/2\rangle + 17\%\|\pm 9/2\rangle + 16\%\|\pm 3/2\rangle + 4\%\|\mp 3/2\rangle + 4\%\|\mp 7/2\rangle$	$\pm$ 2.59
211.71	304.59	0.29	1.74	2.55	61.83	$51\%\|\pm 1/2\rangle + 32\%\|\pm 5/2\rangle + 8\%\|\pm 9/2\rangle + 6\%\|\mp 7/2\rangle$	$\pm$ 1.21
455.43	655.26	0.25	0.30	6.22	58.89	$24\%\|\pm 3/2\rangle + 23\%\|\pm 5/2\rangle + 15\%\|\pm 1/2\rangle + 14\%\|\mp 1/2\rangle + 11\%\|\pm 7/2\rangle$	$\pm$ 0.95
485.36	698.32	0.06	1.21	5.23	88.96	$32\%\|\mp 3/2\rangle + 28\%\|\pm 5/2\rangle + 18\%\|\pm 1/2\rangle + 7\%\|\pm 3/2\rangle +$	$\pm$ 0.15

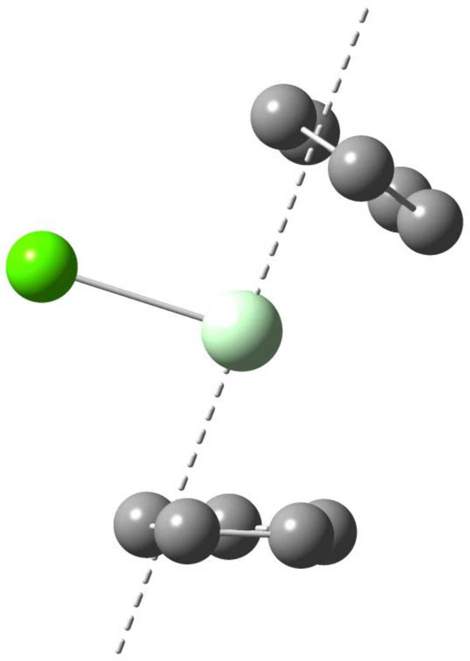

Figure S137. Representation of g_3 direction in the molecular frame for **2-Nd**. *t*Bu groups and H-atoms omitted for clarity.

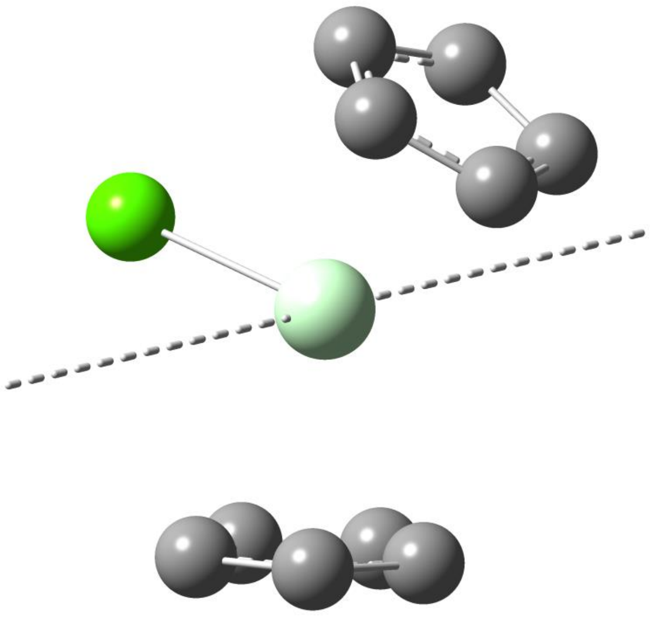

Figure S138. Representation of g_3 direction of the most excited Kramers doublet in the molecular frame for **2-Nd**. *t*Bu groups and H-atoms omitted for clarity.

Table S28. Electronic structure of **1-Sm** calculated with CASSCF-SO at the solid-state geometry in zero-field, quantised along the g_3 direction of the ground doublet.

Energy (cm^{-1})	Energy (K)	g_1	g_2	g_3	angle (deg)	Wavefunction	$\langle J_z \rangle$
0.00	0	0.18	0.38	1.08	-	$100\%\lvert\pm 1/2\rangle$	$\pm$ 0.50
308.69	444.13	0.12	0.13	2.57	2.26	$97\%\lvert\pm 3/2\rangle + 2\%\lvert\mp 3/2\rangle$	$\pm$ 1.43
905.10	1302.23	0.06	0.07	4.04	5.11	$99\%\lvert\pm 5/2\rangle + 1\%\lvert\pm 3/2\rangle$	$\pm$ 2.49

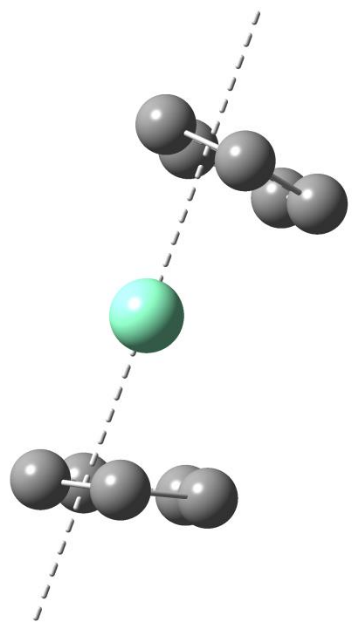

Figure S139. Representation of g_3 direction in the molecular frame for **1-Sm**. *t*Bu groups and H-atoms omitted for clarity.

Table S29. Electronic structure of **2-Sm** calculated with CASSCF-SO at the solid-state geometry in zero-field, quantised along the g_3 direction of the ground doublet.

**Energy (cm^{-1})**	**Energy (K)**	g_1	g_2	g_3	**angle (deg)**	**Wavefunction**	$<J_z>$
0.00	0.00	0.07	0.10	0.71	--	$51\%\|\pm 1/2\rangle$ + $35\%\|\mp 3/2\rangle$ + $7\%\|\pm 5/2\rangle$ + $2\%\|\mp 1/2\rangle$	$\pm$ 0.11
448.53	645.33	0.09	0.45	0.60	88.80	$61\%\|\mp 3/2\rangle$ + $26\%\|\pm 1/2\rangle$ + $14\%\|\pm 5/2\rangle$	$\pm$ 0.44
787.71	1133.34	0.21	0.39	2.21	89.51	$74\%\|\pm 5/2\rangle$ + $18\%\|\pm 1/2\rangle$ + $5\%\|\mp 5/2\rangle$	$\pm$ 1.80

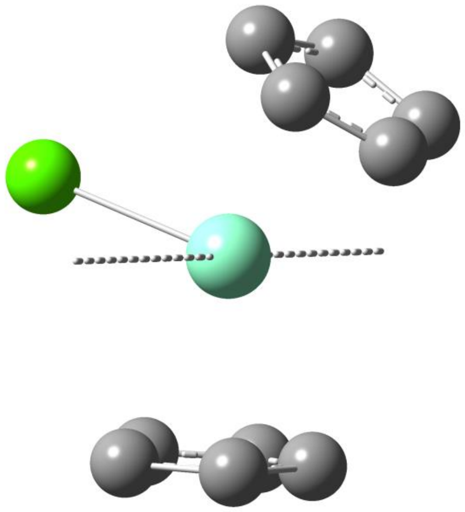

Figure S140. Representation of g_3 direction in the molecular frame for **2-Sm**. *t*Bu groups and H-atoms omitted for clarity.

12. References

[1] *CrysAlisPRO*, Version 39.27b; Agilent Technologies: Yarnton, U.K., 2017.

[2] G. M. Sheldrick, *Acta Crystallogr. Sect. A.* **2008**, *64*, 112–122.

[3] G. M. Sheldrick, *Acta Crystallogr. Sect. C* **2015**, *71*, 3–8.

[4] O. V. Dolomanov, L. J. Bourhis, R. J. Gildea, J. A. K. Howard, H. Puschmann, *J. Appl. Crystallogr*. **2009**, *42*, 339–341.

[5] L. J. Farrugia, *J. Appl. Crystallogr*. **2012**, *45*, 849–854.

[6] *POV-Ray*, Persistence of Vision Raytracer Pty. Ltd.: Williamstown, Australia, **2013**.

[7] G. A. Bain, J. F. Berry, *J. Chem. Ed*. **2008**, *85*, 532–536.

[8] N. F. Chilton, R. P. Anderson, D. L. Turner, A. Soncini, K. S. Murray, *J. Comput. Chem*. **2013**, *34*, 1164–1175.

[9] F. Aquilante, J. Autschbach, R. K. Carlson, L. F. Chibotaru, M. G. Delcey, L. De Vico, I. Fernandez Galván, N. Ferré, L. M. Frutos, L. Gagliardi, M. Garavelli, A. Giussani, C. E. Hoyer, G. Manni, H. Lischka, D. Ma, P. Å. Malmqvist, T. Müller, A. Nenov, M. Olivucci, T. B. Pedersen, D. Peng, F. Plasser, B. Pritchard, M. Reiher, I. Rivalta, I. Schapiro, J. Segarra-Martí, M. Stenrup, D. G. Truhlar, L. Ungur, A. Valentini, S. Vancoillie, V. Veryazov, V. P. Vysotskiy, O. Weingart, F. Zapata, R. Lindh, *J. Comput. Chem*. **2016**, *37,* 506–541.

[10] B. O. Roos, R. Lindh, P.-Å. Malmqvist, V. Veryazov, P.-O. Widmark, *J. Phys. Chem. A*. **2004**, *108*, 2851–2858.

[11] B. O. Roos, R. Lindh, P.-Å. Malmqvist, V. Veryazov, P.-O. Widmark, *J. Phys. Chem. A*. **2005**, *109*, 6575–6579.

[12] L. Ungur, L. F. Chibotaru, *Chem. Eur. J*. **2017**, *23*, 3708–3718.
